# How useful are registered birth statistics for health and social policy? A global systematic assessment of the availability and quality of birth registration data

**DOI:** 10.1186/s12963-018-0180-6

**Published:** 2018-12-27

**Authors:** David E. Phillips, Tim Adair, Alan D. Lopez

**Affiliations:** 1 Institute for Health Metrics and Evaluation, University of Washington, Seattle, USA; 2 Melbourne School of Population and Global Health, University of Melbourne, Melbourne, Australia

**Keywords:** Civil registration, Vital statistics, Birth certificates, Data quality

## Abstract

**Background:**

The registration and certification of births has a wide array of individual and societal benefits. While near-universal in some parts of the world, birth registration is less common in many low- and middle-income countries, and the quality of vital statistics vary. We assembled publicly available birth registration records for as many countries as possible into a novel global birth registration database, and we present a systematic assessment of available data.

**Methods:**

We obtained 4918 country-years of data from 145 countries covering the period 1948–2015. We compared these to existing estimates of total births to assess completeness of public data and adapted existing methods to evaluate the quality and timeliness of the data.

**Results:**

Since 1980, approximately one billion births were registered and shared in public databases. Compared to estimates of fertility, this represents only 40.0% of total births in the peak year, 2011. Approximately 74 million births (53.1%) per year occur in countries whose systems do not systematically register them and release the aggregate records. Considering data quality, timeliness, and completeness in country-years where data are available, only about 12 million births per year (8.6%) occur in countries with high-performing registration systems.

**Conclusions:**

This analysis highlights the gaps in available data. Our objective and low-cost approach to assessing the performance of birth registration systems can be helpful to monitor country progress, and to help national and international policymakers set targets for strengthening birth registration systems.

**Electronic supplementary material:**

The online version of this article (10.1186/s12963-018-0180-6) contains supplementary material, which is available to authorized users.

## Background

The registration and certification of births, while a near-universal practice in some parts of the world, is far less common in many low- and middle-income countries (LMICs) [1]. Birth registration has a wide array of individual and societal benefits [2], including the identification and facilitation of legal entitlements [1], citizenship and voting rights [3], social security benefits, social inclusion [4], access to health and education services [5], security benefits in times of crisis [6], and proof of age [3]. So fundamental is birth registration to legal identity that it has frequently been described as a basic human right [7–9]. Additionally, reliable birth registration, compiled and consolidated within a national civil registration and vital statistics (CRVS) system, should be the primary data source for fertility statistics [10]. Such data are necessary to track (often rapid) changes in fertility levels and patterns, to monitor and evaluate family planning programs, to provide the denominator for an array of key maternal and child mortality indicators [11], to project future population size and structure [12], and to inform planning for future health, education, and other social services. Their fundamental and comprehensive importance for a nation’s health and social development underlies calls for universal birth registration, as reflected in the Sustainable Development Goal 16.9 that aims, by 2030, to provide legal identity for all, including birth registration [13].

As interest in universal birth registration continues to grow, it will become increasingly important for countries and development partners alike to understand the performance of birth registration systems and in particular, to have some objective basis to determine whether these systems are ‘fit-for purpose’, as described above. Yet, despite their fundamental importance, the global status of birth registration is not well understood. While multiple studies have described, assessed, and monitored the global landscape of death registration [14–16], to our knowledge no comparable evaluations of birth registration systems exist. Some partial assessments to guide policy, however, have been undertaken. UNICEF, for example, has estimated that 71% of all children younger than 5 years have had their birth registered [17]. However, this estimate is based on self-reported survey responses that may be biased, especially as data from UNICEF show considerable discrepancies in some countries between reported birth registration and evidence of a birth certificate. For example, for only 10% of births in Rwanda that are reported to be “registered” can the family provide the birth certificate [18]. Moreover, the UNICEF approach does not include information on children who have died, especially neonatal deaths, for whom birth registration is often overlooked [10].

One reason why there has been no systematic assessment of birth registration data and systems could be the absence of a comprehensive and properly maintained and used global database. While agencies such as the World Health Organization annually aggregate and disseminate cause of death statistics based on death registration data from over 150 countries around the world, birth registration data are made public only through information provided by countries to the United Nations Statistical Division via an annual questionnaire, or through country-specific channels (e.g., national statistical offices) and other decentralized sources such as the Human Fertility Collection [19, 20].

To objectively assess the quality of birth registration data, and thus (indirectly) the performance of birth registration systems, it is first necessary to define the essential elements of data quality. While a fundamental measure of the quality of birth registration data is the completeness of registration, i.e., the percentage of all births that occur in a given year that are registered, there is other specific information about the newborn, the mother or the family that is, or should be, routinely collected for each birth and provided along with the birth certificate. Much of this information is likely to be of central importance for public health and demographic purposes, and hence reflects the utility of birth registration data. These characteristics include:

- age of the mother, to understand the age patterns of fertility and to calculate the total fertility rate, the most common summary measure of fertility levels in a population;
- sex of the newborn, to monitor the sex ratio at birth, also as an indicator of sex preferences in fertility [21];
- birth order of the child, to understand fertility behavior (such as stopping behavior and progression patterns from one parity to the next); and
- birthweight, given its critical role for the survival of the newborn [22].

An objective, reliable, and descriptive low-cost approach to assessing the performance of birth registration systems would enable countries to monitor progress in developing their birth registration and reporting systems, by facilitating international goal-setting, facilitating monitoring of development goals, and assisting in the global efforts to improve birth registration that are already underway by identifying specific aspects of data quality or availability that require attention [23]. This paper advances efforts to improve the monitoring of global birth registration in a number of ways. First, we present the results of what we believe is the first systematic effort to assemble publicly available birth registration records for as many countries as possible into a global birth registration database, similar to what WHO maintains for death registration and causes of death. Second, we present a systematic assessment of birth registration data quality around the world. We do so by adapting an existing framework used to assess the quality and utility of death registration statistics, known as the Vital Statistics Performance Index (VSPI) [15], to the context of birth registration. We expect that the birth registration database, and our findings and framework for assessing its utility, will help enable the measurement and tracking of performance metrics, especially between countries, and thus will be of immediate use by both countries and development partners to facilitate monitoring of progress with global and national development goals.

## Methods

### Data

We have systematically compiled a global database^1^ on birth registration statistics, based on 4918 country-years of data from 145 countries covering the period 1948–2015 (Table 1). For each country-year, the number of registered live births^2^ specified by age of mother, sex of newborn, birth order, and birthweight were compiled, where available. These variables are all recommended core topics to be collected for vital statistics purposes in national civil registration systems as specified in the *UN Principles and Recommendations for a Vital Statistics System* [21]. The primary source of data was the United Nations Statistical Division (UNSD) database, which provides birth registration data reported by countries in standardized tables in the *Demographic Yearbook* questionnaire [19, 24]. Because this database covers only a subset of countries likely to have functional birth registration systems, additional data were collected from Eurostat and directly from national statistical offices and ministry of health databases (Table 1). It is important to note that these are the data that are publicly available. Most, if not all, countries are likely to have some form of a birth registration system, but in many countries these data are not published. For example, there are many countries reported by UNICEF as having birth registration data as reported in surveys, but which cannot be found in the UNSD database or country statistical office publications [18].

**Table 1:** Data availability

Region	Country	Years with data^a^
Eastern Europe/Central Asia	Albania	1948, 1950–1967, 1969–1971, 1979–2013
North Africa/Middle East	Algeria	1964–1965, 1978–1980, 1985–1986
East Asia/Pacific	American Samoa	1952–1969, 1971–1973, 1976, 1982, 1984–2014
High Income	Andorra	2002–2012
Latin America/Caribbean	Antigua and Barbuda	1972–1975, 1977–1986, 1993, 1995
High Income	Argentina	1960–1966, 1968–1970, 1979–2014
Eastern Europe/Central Asia	Armenia	1982–1994, 1996–2000, 2002–2004, 2006–2009, 2014
Latin America/Caribbean	Aruba	1993–1995, 1997–2015
High Income	Australia	1948–2014, 2010–2015 [32]
High Income	Austria	1951–2015
Eastern Europe/Central Asia	Azerbaijan	1982–2004, 2006–2010, 2012–2014
Latin America/Caribbean	Bahamas	1968–1977, 1990–1992, 1996
North Africa/Middle East	Bahrain	1977–2014
Latin America/Caribbean	Barbados	1954–1980, 1982–1987, 1990–1991, 2005–2007
Eastern Europe/Central Asia	Belarus	1969–1973, 1986–1999, 2002–2014
High Income	Belgium	1947–1970, 1972–1983, 1986–1987, 1989–2015
Latin America/Caribbean	Bermuda	1962–1965, 1975–1989, 2006–2015
Eastern Europe/Central Asia	Bosnia and Herzegovina	1989–1991, 1996–2010, 2012
Latin America/Caribbean	Brazil	1994–1999, 2000–2015 [33]
High Income	Brunei Darussalam	1969–1974, 1976, 1978, 1981–1992, 1996–2002, 2006–2008, 2011–2014
Eastern Europe/Central Asia	Bulgaria	1949–1990, 1992–2014
Sub-Saharan Africa	Cabo Verde	1979–1985, 1990
High Income	Canada	1948–2009, 2010–2014 [34]
Latin America/Caribbean	Cayman Islands	1981–1983, 1986–1995, 2009, 2011–2014
High Income	Chile	1948–2003, 2005–2014, 1997–1999, 2005–2014 [35]
Latin America/Caribbean	Colombia	1998–2014 [36]
East Asia/Pacific	Cook Islands	1971–1977, 1979–1982
Latin America/Caribbean	Costa Rica	1953–1974, 1976–1991, 1994–1997, 1999–2014
Eastern Europe/Central Asia	Croatia	1988–2014
Latin America/Caribbean	Cuba	1965–1971, 1976–1989, 1991, 1993–2014
Latin America/Caribbean	Curaçao	2009–2015
High Income	Cyprus	1948–2014
Eastern Europe/Central Asia	Czech Republic	1991–2014
High Income	Denmark	1948–1966, 1968–2015
Latin America/Caribbean	Dominica	1960, 1966, 1969, 1985–1989, 2005–2006
Latin America/Caribbean	Ecuador	1992–2007, 2009–2010 [37]
North Africa/Middle East	Egypt	1965–1999, 2006–2012
Latin America/Caribbean	El Salvador	1948–2004, 2005–2007, 2010, 2012
Eastern Europe/Central Asia	Estonia	1986–2015
High Income	Faeroe Islands	1951–1966, 1968–1987, 1989, 2005–2007
East Asia/Pacific	Fiji	1948–1987, 2004, 2008
High Income	Finland	1948–2015
High Income	France	1948–1972, 1974–2009, 2011–2014, 2015 [38]
Latin America/Caribbean	French Guiana	1951–1970, 1972–1976, 1984–1985, 1996, 1998–2003, 2005–2007
East Asia/Pacific	French Polynesia	1968
Eastern Europe/Central Asia	Georgia	1989, 1992, 1994–1997, 1999–2015
High Income	Germany	1991–1997, 1999–2015
High Income	Greece	1956–1985, 1990–2015
High Income	Greenland	1952–1965, 1967–1986
Latin America/Caribbean	Grenada	1951–1969, 1978, 1997, 2000
Latin America/Caribbean	Guadeloupe	1950–1967, 1969–1970, 1975, 1978–1980, 1984–1986, 1991, 1999–2003
East Asia/Pacific	Guam	1949–1986, 1988–1992, 1999, 2001–2004, 2015
Latin America/Caribbean	Guatemala	1948–1973, 1975–1979, 1981–1999, 2006, 2009–2014 [39]
Latin America/Caribbean	Guyana	1954–1956, 1960–1961, 1967–1972,
East Asia/Pacific	Hong Kong	1969–2014
Eastern Europe/Central Asia	Hungary	1948–2015
High Income	Iceland	1948–2015
South Asia	India	2011–2015 [40]
North Africa/Middle East	Iran	2011–2013
High Income	Ireland	1955–2015
High Income	Isle of Man	1955–1961
High Income	Israel	1953–2015
High Income	Italy	1948–1964, 1973, 1980–1997, 1999–2015
Latin America/Caribbean	Jamaica	1948–1964, 1977–1984, 1986–1989,1995–1996, 2000–2004, 2016
High Income	Japan	1948–2010, 2012–2014, 2011, 2015 [41]
North Africa/Middle East	Jordan	1969–1979, 2000–2015 [42]
Eastern Europe/Central Asia	Kazakhstan	1987–2008, 2012–2013
Eastern Europe/Central Asia	Kosovo	2002–2003, 2005, 2008, 2011
North Africa/Middle East	Kuwait	1963–1970, 1972, 1987, 1991–2014
Eastern Europe/Central Asia	Kyrgyzstan	1980, 1982–2015
Eastern Europe/Central Asia	Latvia	1986–2015
North Africa/Middle East	Libya	1972–1977, 1981, 1996, 2000, 2002
High Income	Liechtenstein	1965–1966, 1968, 1978–1983, 1986, 1987, 1993, 2003–2014
Eastern Europe/Central Asia	Lithuania	1970–1977, 1985–2015
High Income	Luxembourg	1948–2014, 2015 [38]
East Asia/Pacific	Macao	1955–2015
East Asia/Pacific	Malaysia	1990–1997, 2001–2009, 2011–2015
East Asia/Pacific	Maldives	1996, 1999–2014
Sub–Saharan Africa	Mali	1897
High Income	Malta	1957–1990, 1992–2015
Latin America/Caribbean	Martinique	1950–1970, 1972–1976, 1984–1992, 1999–2003, 2005–2007
East Asia/Pacific	Mauritius	1990–2003, 2005–2015
Latin America/Caribbean	Mexico	1985–2015 [43]
Eastern Europe/Central Asia	Moldova	1987–1992, 1995–1996, 1998–2014
Eastern Europe/Central Asia	Mongolia	1980, 1990, 1994–2010, 2012–2015
Eastern Europe/Central Asia	Montenegro	1980, 1990, 2000, 2003–2009
Latin America/Caribbean	Montserrat	1982–1986, 1994–1999, 2010–2014
North Africa/Middle East	Morocco	1990–1991, 1993, 1995–2001
East Asia/Pacific	Nauru	1965–1968, 2009–2011
High Income	Netherlands	1948–2014, 2015 [38]
East Asia/Pacific	New Caledonia	1962–1968, 1970–1985, 1987, 1990–1994, 1996–2003, 2005–2007, 2010, 2012
High Income	New Zealand	1962–2015
East Asia/Pacific	Niue	1957–1962, 2009
East Asia/Pacific	Norfolk Island	1948–1972, 1974–1976, 1978–1981, 1983–1984, 1988,
High Income	Norway	1948–2014, 2015 [38]
North Africa/Middle East	Oman	2006–2015 [44]
East Asia/Pacific	Palau	1989–2005
Latin America/Caribbean	Panama	1950, 1952–2000, 2002–2003, 2005–2015
Latin America/Caribbean	Peru	2013–2015 [45]
East Asia/Pacific	Philippines	1990–1993, 1997–2007, 2009–2015
Eastern Europe/Central Asia	Poland	1950–2015
High Income	Portugal	1948–2015
Latin America/Caribbean	Puerto Rico	1948–1962, 1964–1985, 1987–1994, 1996–2000, 2002–2009, 2012–2015
North Africa/Middle East	Qatar	1985–1994, 1996–2010, 2012–2013
High Income	South Korea	1993–2014
East Asia/Pacific	Reunion	1950–1970, 1980, 1982–1986, 1989, 1993–1997, 2002–2003, 2005–2007
Eastern Europe/Central Asia	Romania	1955, 1957–2014, 2015 [38]
Eastern Europe/Central Asia	Russia	1960,1965, 1970, 1975, 1980–1989, 1991–2004, 2006–2011, 2013, 2014 [38]
High Income	Saint Pierre and Miquelon	1948–1952, 1959, 1963–1964, 1967, 1969, 1973–1977
Latin America/Caribbean	Saint Vincent and the Grenadines	1952–1956, 1960–1964, 1977–1984, 1986, 1988, 1992–1994, 1996–2005, 2008–2009, 2013–2014
Latin America/Caribbean	Saint Kitts and Nevis	1956–1972, 1974–1991, 1993–1996
Latin America/Caribbean	St Lucia	1953–1961, 1963, 1975, 1978–1986, 1994–2002, 2004–2005
East Asia/Pacific	Samoa	1993
High Income	San Marino	1960–1989, 1992–1995, 1997–2004, 2011–2014
Sub-Saharan Africa	Sao Tome and Principe	1958, 1974–1979
Eastern Europe/Central Asia	Serbia	2000–2015
East Asia/Pacific	Seychelles	1982,1984–1985, 1990, 1992–1993, 1995–1996, 2004–2015
High Income	Singapore	1948–2015
Eastern Europe/Central Asia	Slovakia	1988–1995, 1997–2015
Eastern Europe/Central Asia	Slovenia	1987–2015
Sub-Saharan Africa	South Africa	1998–2015 [46]
High Income	Spain	1948–1983, 1985–2014, 2015 [38]
East Asia/Pacific	Sri Lanka	1952–1969, 1977–1989, 1991, 1995–1996, 2001, 2006–2010
Latin America/Caribbean	Suriname	1980–1986, 1988–2007, 2012–2014
High Income	Sweden	1948–2014, 2015 [38]
High Income	Switzerland	1948–1982, 1984–2014, 2015 [38]
East Asia/Pacific	Taiwan	1982–2014 [47]
Eastern Europe/Central Asia	Tajikistan	1989–1994, 2001–2003
Eastern Europe/Central Asia	TFYR of Macedonia	1989–2015
East Asia/Pacific	Thailand	1991–1992, 1994, 1997
Sub-Saharan Africa	Tonga	1990, 1993–2000, 2002–2003
Latin America/Caribbean	Trinidad and Tobago	1992–1995, 1997, 2002, 2004–2006, 2008–2009
North Africa/Middle East	Tunisia	1960, 1965–1972, 1974, 1977–1980, 1985–1989, 1992–1995, 1998, 2006–2007, 2011
North Africa/Middle East	Turkey	2009–2015
Eastern Europe/Central Asia	Turkmenistan	1989
Latin America/Caribbean	Turks and Caicos Islands	2001–2005
Eastern Europe/Central Asia	Ukraine	1969–1971, 1973–1975, 1987–1996, 1998, 2001–2004, 2006–2008, 2010–2012, 2014–2015
High Income	United Kingdom	1982–2004, 2007–2014, 2015 [38]
High Income	United States	1948–1989, 1991, 1993–2002, 2003–2015 [48]
Latin America/Caribbean	United States Virgin Islands	1948–1962, 1964–1967, 1969–1972, 1977–1997
High Income	Uruguay	1949–1954, 1963, 1977–1989, 1993, 1996–1997, 1999–2007, 2012–2014 [49]
Eastern Europe/Central Asia	Uzbekistan	1989, 1993–1997, 1999–2000, 2005–2015
Latin America/Caribbean	Venezuela	1990–1991, 1996, 1998–2002, 2005–2007, 2009–2015
East Asia/Pacific	Wallis and Futuna Islands	1969, 1996–2008
Eastern Europe/Central Asia	Yugoslavia	1994–1995

^a^Unless otherwise specified with a citation, the source for data is [19] UN Statistics Division. UNSD Demographic Statistics [Internet]. United Nations; 2017. Available from: http://data.un.org

### Assessment of completeness

In order to assess birth registration completeness, we relied on existing annual estimates of total births produced by the United Nations Population Division [25]. We use these estimates as a measure of the total number of live births that occur each year in a country, recognizing that they are subject to methodological and empirical uncertainty. They are, however, the only estimates of the total numbers of births occurring in countries currently available. The observed number of births reported for each country were divided by estimates of the total number of live born in each country-year.

The resulting figures thus represent a measure of completeness of birth registration data *in the public domain*. We assume that in a country-year which has made birth registration data available, the data include all registered births for that year, and therefore can be used to assess registration completeness. In country-years where no data are available, we are unable to draw conclusions about registration completeness.

### Vital statistics performance index

To evaluate the *utility* of vital statistics with respect to their accuracy in addition to their completeness and availability, we adapted methods defined by Phillips et al. 2014 [15]. In their study, six empirical indicators were used to create a summary index of death registration data utility known as the VSPI. Comparably, we defined four indicators of *data quality*: the proportion of registered births with unspecified maternal age, the proportion of registered births with unspecified newborn sex, the proportion of registered births with unspecified birthweight, and the proportion of registered births with unspecified live birth order. Following the VSPI framework, we included two additional components of system performance which, together with the four components of data quality mentioned above define a summary of the overall accuracy of birth registration data.

These indicators of performance were selected on the basis of their suitability for assessing the policy relevance of demographic and fertility statistics (as described above), their availability in many data systems, and their inclusion in global recommendations for vital registration systems [21]. In doing so, we implicitly assume that complete, accurate, and recent information about maternal age, newborn sex, birthweight, and live birth order are useful to describe about the distribution and trends of fertility, and can summarize the overall accuracy of data used to represent those fertility trends.

As detailed in Phillips et al. 2014 [15], simulation techniques were used to combine the six indicators into a composite index. The purpose of the simulation is to assess the distortion in observed fertility trends as compared to the true underlying trends associated with different levels of the above indicators. As an example, if a certain proportion of births are reported with an unknown sex, the simulation approach measures the accuracy of sex ratios in the observed data as compared to the sex ratio of the population from which the data were derived. Each other indicator’s accuracy was assessed using a separate, relevant objective function. Maternal age was evaluated using the fraction of births in each age group (less than 15 years of age, 15–19, 20–24, 25–29, 30–34, 35–39, 40–44, and greater than or equal to 45 years of age). Birthweight was evaluated using the fraction of births in each birthweight category (less than 2500 g, 2500–3499, and greater than 3500 g). Birth order was evaluated using the proportion of births in each sibship size (0, 1, 2, or 3 or more livebirth siblings). Like the VSPI framework, we used the population-level accuracy formula defined by Murray et al. 2011 to assess the similarity between observed fractions and that of the underlying simulated population [26].

We used the above-mentioned estimates of birth counts as the population for the simulation [25]. Because these estimates are not disaggregated by sex, birthweight, and live birth order of the newborn, and because no other global estimates are as well, to our knowledge, we developed an approach to disaggregating them based on available data. We combined publicly-available survey data as direct measures of the fraction of births in each birth group (age, sex, birthweight, and birth order). These data included 211 Demographic and Health Surveys from 73 countries and the UK Understanding Society Longitudinal Household Study [27, 28]. We used regression techniques (see Additional file 1 for details) to estimate the fraction of births by birth group from the survey data. Modeled birth fractions were multiplied by the UN estimates of births by country, year, and maternal age to disaggregate them, leaving the total unchanged.

Using these estimates of birth counts as a population of simulated births, we drew a weighted sample of birth certificates in order to simulate progressively less-than-complete registration. Observed patterns of missing data from the birth registration database described above were used as empirical probabilities for weighted sampling. Finally, we computed observed proportions of missing data among the actual data, and simulation results were used to assess the accuracy of those observed proportions.

The separate indicators of data quality were then combined by taking the product of accuracy measures from the simulation. Following Phillips et al. 2014 [15], an exponential smoothing algorithm was applied to the product in order to measure the component of overall utility related to the timeliness of the data. Further details on the exact computation of the VSPI has been described elsewhere [15].

The result of this simulation and smoothing procedure is a single index of the policy utility of birth registration statistics for a given population in a given year, simultaneously capturing data availability, quality, completeness, and timeliness, which we will term VSPI-B. This index quantifies the extent to which registered and available birth data are accurate in reflecting the underlying demographic profile of births in the country.

## Results

We analyzed 2680 country-years of data, from 109 countries spanning 1980 to 2015. We found 51 countries with greater than or equal to 30 years of available data since 1980, 75 countries with greater than or equal to 20 years of available data, and 11 countries with less than or equal to five years. Available data came from 32 high-income countries, 29 countries from Eastern Europe or Central Asia, 20 countries from Latin America and the Caribbean, 12 countries from North Africa and the Middle East, 11 countries from East Asia and the Pacific, and four countries from Sub-Saharan Africa (Table 1). Notably, several populous countries (e.g., China, Bangladesh and Pakistan) did not have any birth registration data publicly-available for analysis.

**Fig. 1:**
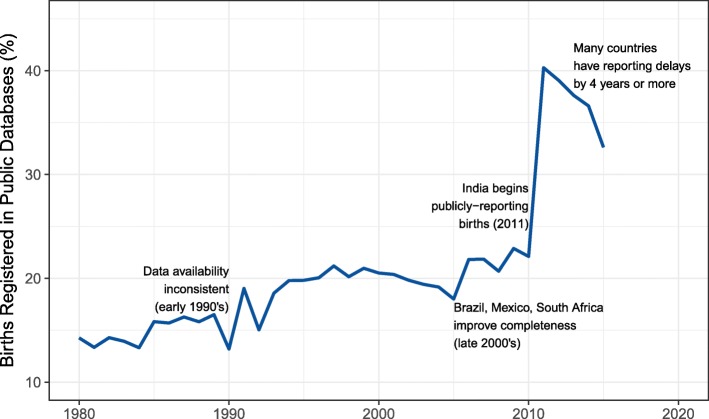
Percentage of global births registered in publicly-available data

The data we were able to gather represented approximately 27.9 million births per year on average, ranging from 16.8 million births recorded in 1981 to 55.3 million births recorded in 2011, for a total of 1.01 billion births registered since 1980. The available data represented only 20.8% of the estimated total number of birth worldwide. This figure varied from 13.2% in 1990 to 40.0% in 2011, the most recent year for which data was available for most of the reporting countries. In 2015, the most recent year for which data were available, global availability was estimated as 32.6%. The most notable change in global registration completeness occurred in 2011, when India began publicly reporting data. Figure 1 displays the global percentage of births registered based on publicly-available data over time.

Based on their most recent year with available data, 83 countries had estimated completeness greater than 90%, 19 countries had estimated completeness between 80 and 90%, five countries had estimated completeness between 50 and 80%, and two countries had estimated completeness below 50%. Completeness estimates for the most recent year for each country with available data are shown in Table 2. Additional file 2 displays the time series of completeness for each country.

**Table 2:** Birth registration completeness in most recent year by country

Country	Year	Completeness (%)
Albania	2013	90
Algeria	1986	90
Antigua and Barbuda	1995	97
Argentina	2014	100
Armenia	2014	100
Australia	2014	95
Austria	2015	100
Azerbaijan	2014	85
Bahamas	1996	100
Bahrain	2014	99
Barbados	2007	100
Belarus	2014	100
Belgium	2015	94
Bosnia and Herzegovina	2012	93
Brazil	2015	99
Brunei Darussalam	2014	100
Bulgaria	2014	100
Canada	2014	100
Chile	2014	100
Colombia	2014	90
Costa Rica	2014	100
Croatia	2014	95
Cuba	2014	100
Cyprus	2014	71
Czech Republic	2014	100
Denmark	2015	100
Ecuador	2010	88
Egypt	2012	100
El Salvador	2012	100
Estonia	2015	100
Fiji	2008	91
Finland	2015	98
France	2015	100
Georgia	2015	100
Germany	2015	100
Greece	2015	100
Grenada	2000	96
Guatemala	2014	88
Hong Kong	2014	81
Hungary	2015	100
Iceland	2015	97
India	2015	92
Iran	2013	100
Ireland	2015	91
Israel	2015	100
Italy	2015	100
Jamaica	2006	84
Japan	2015	99
Jordan	2015	93
Kazakhstan	2013	100
Kuwait	2014	80
Kyrgyzstan	2015	94
Latvia	2015	100
Libya	2002	90
Lithuania	2015	100
Luxembourg	2015	96
Macao	2015	89
Macedonia	2015	97
Malaysia	2015	100
Maldives	2014	91
Mali	1987	95
Malta	2015	100
Mauritius	2015	95
Mexico	2015	87
Moldova	2014	89
Mongolia	2015	100
Montenegro	2009	100
Morocco	2001	89
Netherlands	2015	98
New Zealand	2015	100
Norway	2015	100
Oman	2015	85
Panama	2014	100
Peru	2015	85
Philippines	2015	74
Poland	2015	95
Portugal	2015	100
Puerto Rico	2015	73
Qatar	2013	93
Romania	2015	100
Russia	2014	99
Saint Vincent and the Grenadines	2014	100
Samoa	1993	37
Serbia	2015	73
Seychelles	2015	97
Singapore	2015	83
Slovakia	2015	99
Slovenia	2015	92
South Africa	2015	83
South Korea	2014	93
Spain	2015	100
Sri Lanka	2010	100
Suriname	2014	100
Sweden	2015	97
Switzerland	2015	100
Taiwan	2014	100
Tajikistan	2003	49
Thailand	1997	95
Tonga	2003	97
Trinidad and Tobago	2009	89
Tunisia	2011	100
Turkey	2015	100
Turkmenistan	1989	96
Ukraine	2015	85
United Kingdom	2015	96
United States	2015	100
Uruguay	2014	100
Uzbekistan	2015	100
Venezuela	2015	100

Among the indicators of data quality, most country-years reported births by maternal age and the newborn’s sex (95.3 and 73.4% of country-years respectively), when data were available. Fewer countries reported births by live birth order and birthweight, with 55.6 and 51.1%, respectively, of country years containing these indicators. Among countries which did report each indicator, some missing values were observed as well. The indicator with the highest proportion missing was birth weight, with 2.6% of births with unknown birth weight. Maternal age and live birth order had fewer missing values: 1.0% each. Births without a recorded sex were very rare, occurring in only 0.05% of cases. Additional file 2 displays the level of each indicator over time by country.

**Fig. 2:**
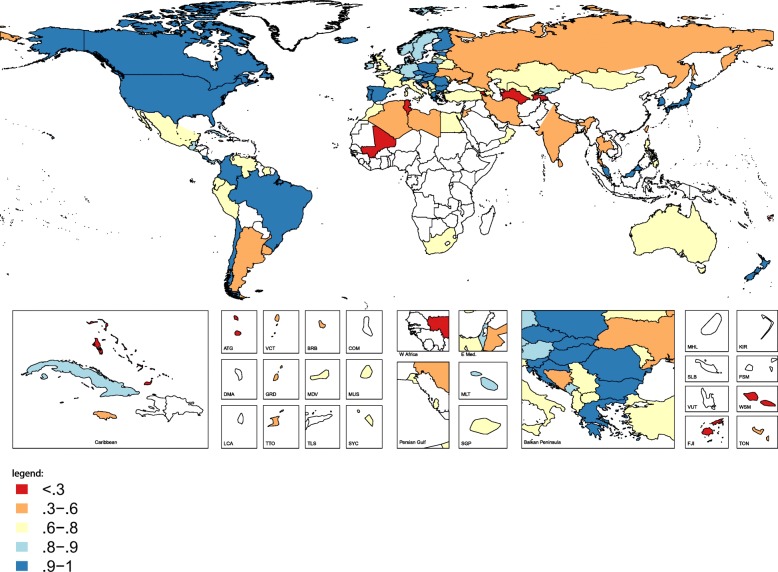
Vital statistics performance index (most recent year with available data)

Combining completeness, quality, and timeliness, Fig. 2 displays the VSPI-B scores for each country for their most recent year with available data. 26 countries had VSPI-B scores in the highest category, between 0.9 and 1. These countries include many high- and middle-income countries with high completeness, and are generally countries which report births by all four data quality indicators. 17 countries had VSPI-B scores in the range 0.8–0.9. These countries also typically included mostly high- and middle-income countries, and were characterized by high completeness but sporadic reporting of the four data quality indicators. 38 countries were in the range 0.6–0.8. Spanning high-, middle-, and lower-middle income countries, these countries’ VSPI-B scores were driven by a mixture of lower completeness and lack of reporting of one or more data quality indicator. 19 countries had VSPI-B scores in the 0.3–0.6 range, characterized by either lower completeness, erratic availability of data, and/or lack of reporting on multiple data quality indicators (i.e., only reporting births by mother’s age or newborn’s sex, but not the others). Finally, nine countries had VSPI-B scores that were lower than 0.3. These countries typically had only few years with available data, low completeness, and/or lack of reporting of multiple indicators of data quality. Additional file 2 displays the time series of VSPI-B scores for each country.

The results from the simulation indicate that, all else being equal, the completeness indicator has the highest weight in the VSPI-B. This is evidenced by Additional file 3, which displays the simulated accuracy associated with each indicator at varying levels among simulated samples. At high levels, all five indicators have generally similar accuracy (i.e., similar influence on the VSPI-B score), but at lower levels the indicators have quite different values. This is the result of different empirical simulation probabilities to inform the weighted samples.

## Discussion

This paper presents, for the first time to our knowledge, a systematic assessment of the availability and quality of data reported by birth registration systems worldwide. As Mikkelsen et al. 2015 [16] argue, vital registration data quality can be assumed to be an accurate reflection of the performance of the registration system itself. In assessing birth registration data quality, we demonstrate each country’s progress toward strengthening birth registration through an adaptation of the Vital Statistics Performance Index. We also present estimates of the country-level completeness of birth registration based on available data. This assessment is based on the largest database of its kind, containing records of over one billion births since 1980 by country, year, maternal age, sex, birthweight, and live birth order.

Although over 100 countries had at least one data point, the availability of data remains low in many parts of the world. Our assessment of birth registration availability and completeness, where available, demonstrates that sharing of birth registration data is surprisingly low compared with death registration, although it does appear to be increasing. Only around 33% of births worldwide in 2015 were registered with the aggregate records made publicly available. This (and even the 2011 peak of 40% availability) is considerably less than for deaths, where 55–60% of global deaths are now registered in publicly available data systems. Further, we found considerable variance between birth reporting systems in that some countries report births by maternal age, sex, birthweight, and live birth order, while others exclude some or all of this information.

This description of birth registration completeness in country-years where it is possible to assess is in stark contrast to other assessments, particularly UNICEF’s State of the World’s Children report [17]. In their most recent such report, completeness estimates are much higher than those presented here, even in country-years with data available to assess. For example, the available data from the Philippines in 2015 represent only 74% of the estimated births according to our assessment, but the UNICEF report estimates 90% completeness. Notable examples of large discrepancies in other parts of the world include Peru (85% completeness according to 2015 data, as compared with 98% according to UNICEF), and Serbia (73% as compared to 99%). Other countries, such as South Africa, are closer, but still different (83% as compared to 85%). The reasons for the discrepancies are likely twofold. First, the alternative estimates of global birth registration completeness are based on self-report in surveys, not actual records of birth certificates. Issues of recall bias, survival bias, and survey instruments that do not confirm the actual existence of the birth certificates, are likely to lead to over-estimates of completeness via this method. Second, the estimates of completeness we present reflect the accuracy of the estimated denominator data as much as they do the completeness of systems. As already noted, the model estimates of total births include uncertainty intervals within which the total births may fall. While it would have been ideal to propagate that uncertainty into our estimates of completeness, uncertainty estimates were not available to do so at the time of analysis.

Altogether, these findings imply that approximately 74 million births (53.1% of annual global births) per year occur in countries whose systems do not systematically register them and release the records publicly. Conversely, only about 12 million births per year (8.6%) occur in countries with high-performing registration systems, i.e., those with consistent data availability, high completeness and reporting by maternal age, sex, birthweight and live birth order.

The assessment of birth registration is not without limitations however. Primarily, these numbers are based on available data only. This caution is especially salient in that it renders estimates of global registration completeness impossible, as noted above. There are likely more births registered per year that do not get aggregated and reported in order for us to assess them. As such, these availability numbers should be considered as the minimum completeness, and are most useful in countries where data are public. Evidence from China, for example, suggests that about 10 million births per year are registered in the country, which would increase global birth registration completeness to close to 50% were they to be made available for analyses such as that reported here. The assessment of completeness where data are available may also be limited by the assumption that all registered births are reported when a public release is made. The assessment of data quality is also limited to the data that are available. Many countries may have low VSPI-B scores not because their registration systems are functioning poorly, but because the data aren’t released. That includes missing years, but also failure to report certain variables. For example, it is rare to fail to record the sex of a child on their birth certificates, but many countries have not made such information publicly available. Without further data to inform our assessment, it is impossible to distinguish the reasons for lack of reporting. Additionally, some of the details of the VSPI-B simulations are subject to limitations. Principal among them is the fact that the simulations and estimates of disaggregated birth counts are based, in part, on Demographic and Health Surveys and the Understanding Society Survey. With more data, these estimates may have been more accurate. Finally, it could be argued there are other means of measuring the quality of birth registration data; for example, the percentage of births that are registered late or with unspecified type of site of occurrence (e.g., hospital, home etc.). However, given the largest available source of data, the UN database, did not collect these data, we were not able to include them in our analysis.

## Conclusion

Our findings have a number of important implications and uses. First, we highlight the gaps in available data. While national policymakers may have unpublished data at their disposal, international and multinational health and development organizations are often reliant on public information of registered births, which we demonstrate are unavailable in many country-years. These findings underscore the significance of open data practices for public policy.

Second, we present an objective and low-cost approach to assess the performance of birth registration systems, wherever data are available. This can be helpful to monitor country progress and benchmark efforts to improve birth registration against national and international goals, especially in an era with significant multilateral, bilateral, and philanthropic investments in strengthening CRVS systems [23]. In addition, we present a set of metrics for completeness and overall system performance that is consistent between countries and over time. As such, these results may be useful for international goal-setting.

An important outcome of this work should be to highlight both the importance of birth registration as a source of fertility statistics and the limitations of the available data. National and subnational governments require routine and timely birth registration data for a range of purposes, not least of which is to lessen reliance on costly sample surveys such as the Demographic and Health Surveys that produce fertility statistics with considerable uncertainty in small areas and which can be 2–5 years out of date once available. More generally, the data generated by civil registration systems are of paramount importance to global health and development efforts, as well as for critical epidemiologic and demographic research [10, 16, 29]. Some authors have even argued that the heightened ability to design and implement effective health policy afforded by greater civil registration has led to a measurable relationship with population health outcomes [30, 31]. This will hopefully encourage stakeholders to collect, consolidate, use, and release more data, release data more promptly, and ensure they maintain a centralized, standardized system for aggregating birth registration data. Considering that birth registration is seen as a fundamental human right, and given the enormous policy relevance of timely, accurate, and complete information on fertility patterns, our findings should be taken as an urgent call for immediate, coordinated, and sustained support to countries to strengthen birth as well as death registration systems, and for greater global efforts to register births and incorporate the minimum demographic and health indicators associated with each of them.

## Additional files

Additional file 1: Further Statistical Details: Disaggregating Estimated Birth Counts by Sex, Birthweight and Live Birth Order. A two-page document describing the methods applied to estimate birth counts disaggregated by sex, birthweight and live birth order. (DOCX 16 kb)

Additional file 2: VSPI-B Estimates and their Component Indicators by Country. A figure for every country with available data, displaying the observed data, final VSPI-B estimate, and sub-plots for each of the five components of the VSPI. (PDF 600 kb)

Additional file 3: Simulated Age-Sex-Parity-Birthweight Fraction Accuracy Associated with Each Indicator. A figure displaying the results of the simulation procedure. The lines demonstrate the accuracy of simulated data in terms of the fraction of births in each birth group, as compared to the underlying population. Each line represents a different component of the VSPI-B at different simulated levels of that component. (PDF 7 kb)

## Abbreviations

CRVS: Civil registration and vital statistics
LMICs: Low- and middle-income countries
UNSD: United Nations Statistical Division
VSPI: Vital Statistics Performance Index

## Footnotes

1 Data file can be downloaded from http://ghdx.healthdata.org/record/global-birth-registration-database-1948-2015

2 The analysis is restricted to live births.
