# Supplementary material for: How useful are registered birth statistics for health and social policy? A global systematic assessment of the availability and quality of birth registration data
Source: Popul Health Metr. 2018 Dec 27;16:21. doi: 10.1186/s12963-018-0180-6 (PMC6307230; doi:10.1186/s12963-018-0180-6)
Supplement: Supplementary file 1 — Further Statistical Details: Disaggregating Estimated Birth Counts by Sex, Birthweight and Live Birth Order. A two-page document describing the methods applied to estimate birth counts disaggregated by sex, birthweight and live birth order. (DOCX 16 kb) [file 12963_2018_180_MOESM1_ESM.docx]

# Further statistical details: disaggregating estimated birth counts by sex, birthweight, and birth order

As stated in the manuscript, this study relied on existing estimates of total births by country, year, and maternal age. These estimates were downloaded from the United Nations annual publication, “World Population Prospects, 2017.”(1)

Also stated in the manuscript, the VSPI uses a simulation approach to estimate the accuracy of observed data at varying levels of quality. This simulation approach samples data from a simulation population, and compares the trends in the “observed” (sampled) data to the original population.

To define a sufficiently-detailed simulation population, we disaggregated the UN estimates by sex, birthweight, and birth order of the newborn. To do so, we combined data from 211 Demographic and Health Surveys from 73 countries and the UK Understanding Society Longitudinal Household Study.(2,3) These surveys were chosen for their high global geographic coverage, and because they report births by year, maternal age, newborn sex, newborn birthweight, and birth order.

Survey data were aggregated to compute the fraction of births in each birth group (country-year-age-sex-weight-order). In order to synthesize overlapping data, account for sampling design and sample size, and to extrapolate beyond observed data, we used regression techniques to estimate mean birth fractions from the surveys.

The regression technique we used is known as seemingly unrelated regression (SUR). This allowed us to estimate the fraction of births in each birth group simultaneously, while ensuring that the estimated fractions continue to sum to 100%. We fit the SUR model among the survey data, then used model coefficients to predict birth fractions among all country-years for which UN estimates were available. These predicted fractions were then multiplied by country-year-age-specific birth counts to disaggregate.

The SUR model was constructed to utilize all available information in the UN estimates when predicting out of sample, while still estimating an identifiable model in the survey data. We therefore used the cross-product of geographic region, year and maternal age as predictor variables for each of the 24 birth groups. The following formulae represent the regression model:

$$p1_{ijk}\sim region_{ijk}*year_{ijk}*age_{ijk}$$

$$p2_{ijk}\sim region_{ijk}*year_{ijk}*age_{ijk}$$

$$\ldots$$

$$p{24}_{ijk}\sim region_{ijk}*year_{ijk}*age_{ijk}$$

Where $p1_{ijk}$ represents the proportion of births in the first birth group (maternal age: <20, newborn sex: male, birthweight: <2500 grams, birth order: 1), indexed by country $i$, year, $j$ and maternal age, $k$. The system of equations was fit using the *systemfit* package in R (version 3.4.0).(4)

1. United Nations, Department of Economic and Social Affairs, Population Division. World Population Prospects: The 2017 Revision, Volume I: Comprehensive Tables. [Internet]. UN; 2017 [cited 2017 Nov 25]. Available from: https://esa.un.org/unpd/wpp

2. ICF International. The DHS Program: Demographic and Health Surveys [Internet]. USAID; [cited 2017 Nov 25]. Available from: https://dhsprogram.com/data/

3. University of Essex. Institute for Social and Economic Research. Understanding Society: Innovation Panel, Waves 1-8, 2008-2015. UK Data Service; 7th Edition.

4. Arne Henningsen, Jeff D. Haman. systemfit: Estimating Systems of Simultaneous Equations [Internet]. CRAN: The Comprehensive R Archive Network; 2017 [cited 2018 Feb 10]. Available from: https://cran.r-project.org/web/packages/systemfit/systemfit.pdf
