# Supplementary material for: How useful are registered birth statistics for health and social policy? A global systematic assessment of the availability and quality of birth registration data
Source: Popul Health Metr. 2018 Dec 27;16:21. doi: 10.1186/s12963-018-0180-6 (PMC6307230; doi:10.1186/s12963-018-0180-6)
Supplement: Supplementary file 2 — VSPI-B Estimates and their Component Indicators by Country. A figure for every country with available data, displaying the observed data, final VSPI-B estimate, and sub-plots for each of the five components of the VSPI. (PDF 600 kb) [file 12963_2018_180_MOESM2_ESM.pdf]

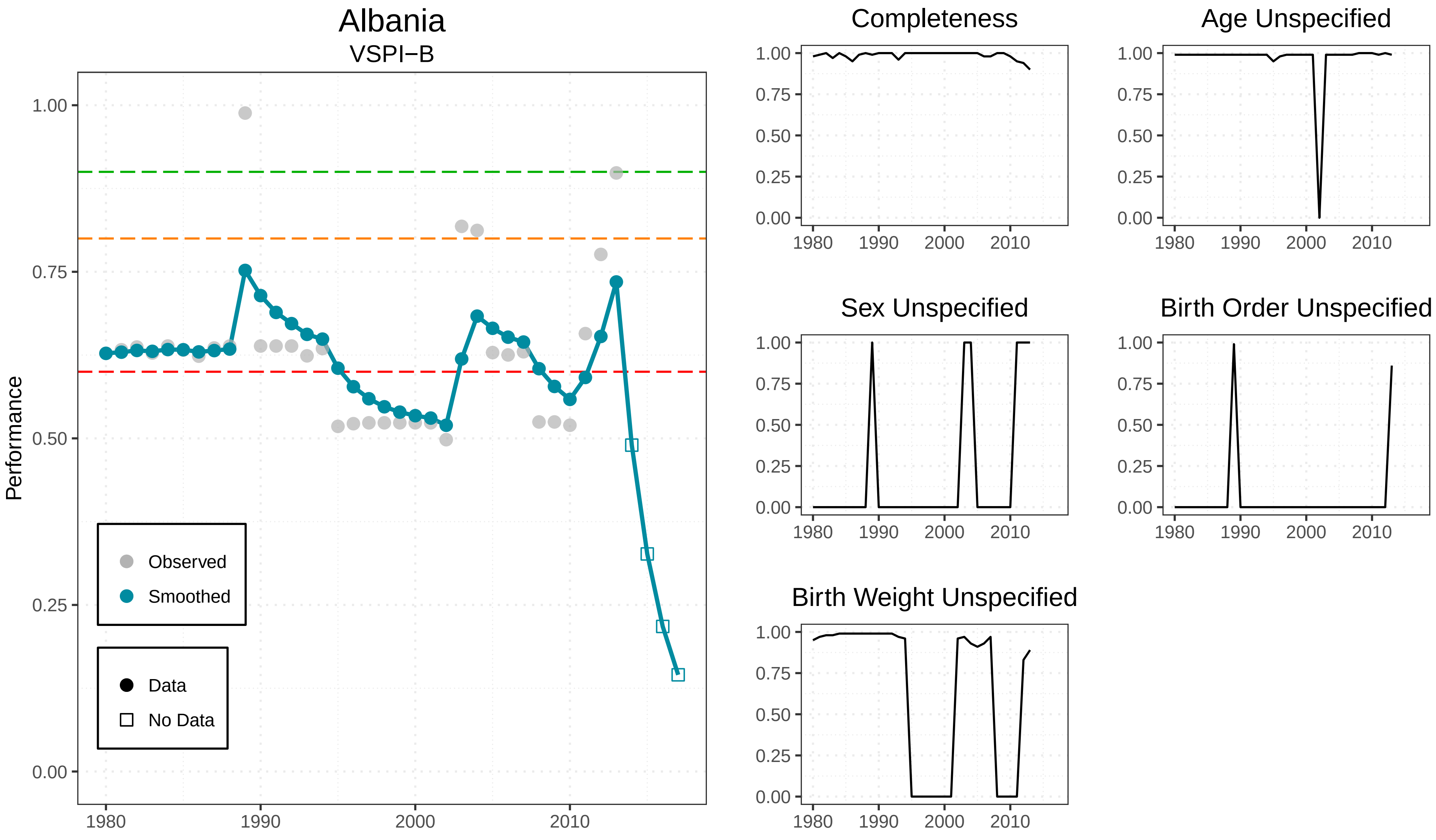

Albania
VSPI−B
Performance
1.00
0.75
0.50
0.25
0.00
1980
1990
2000
2010
Observed
Smoothed
Data
No Data
Completeness
Age Unspecified
Sex Unspecified
Birth Order Unspecified
Birth Weight Unspecified

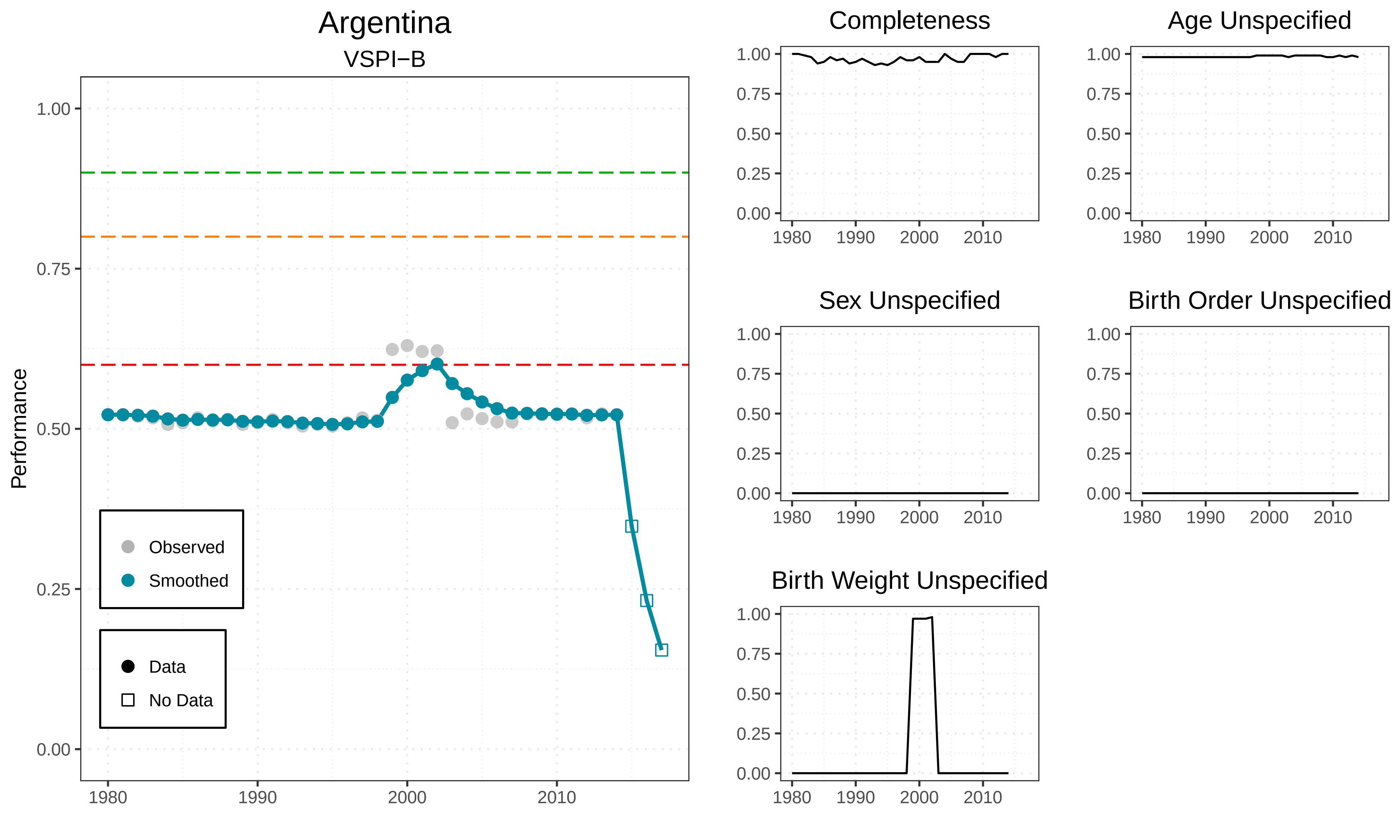

Argentina
VSPI−B
Performance
1.00
0.75
0.50
0.25
0.00
1980
1990
2000
2010
Observed
Smoothed
Data
No Data
Completeness
Age Unspecified
Sex Unspecified
Birth Order Unspecified
Birth Weight Unspecified

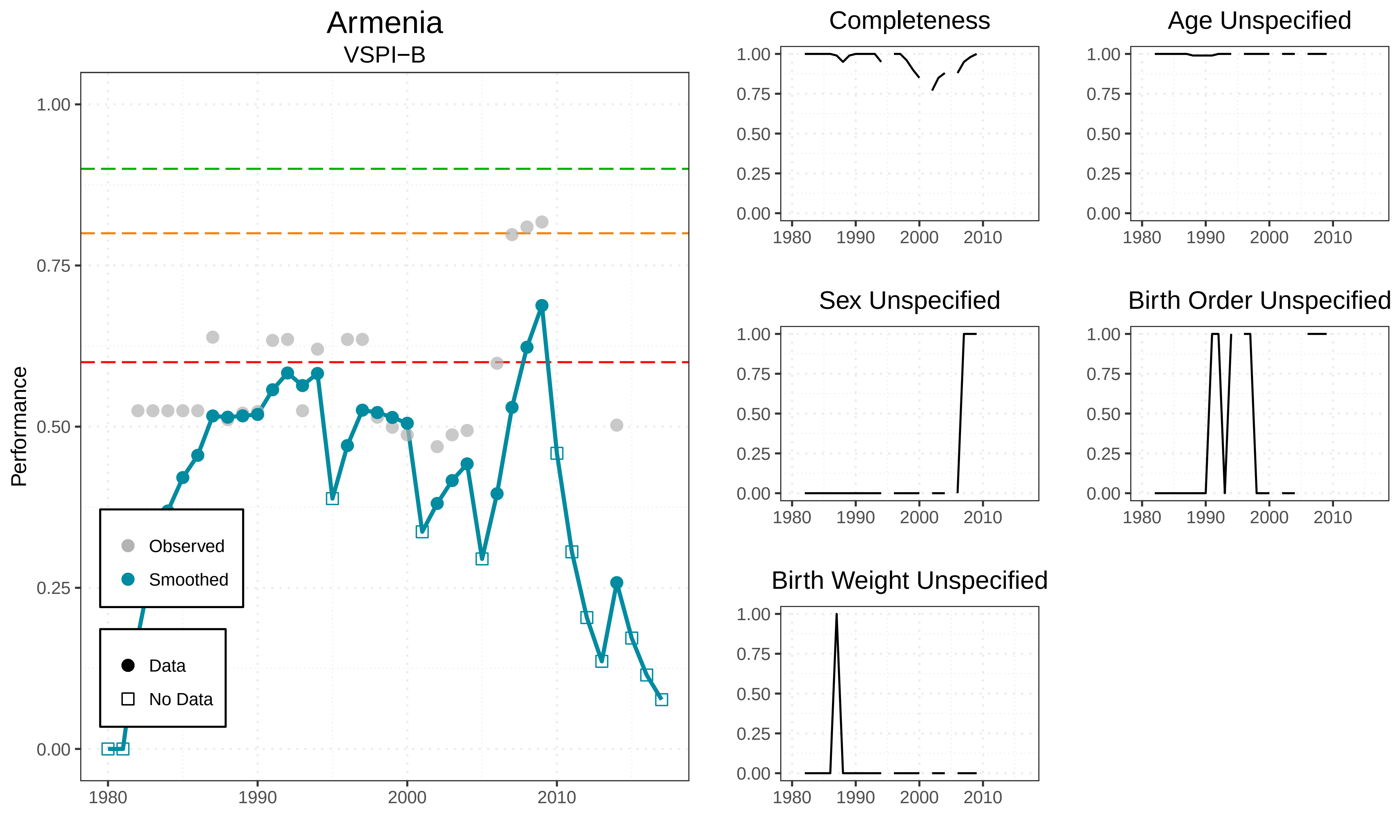

Armenia
VSPI−B
Performance
Observed
Smoothed
Data
No Data
Completeness
Age Unspecified
Sex Unspecified
Birth Order Unspecified
Birth Weight Unspecified

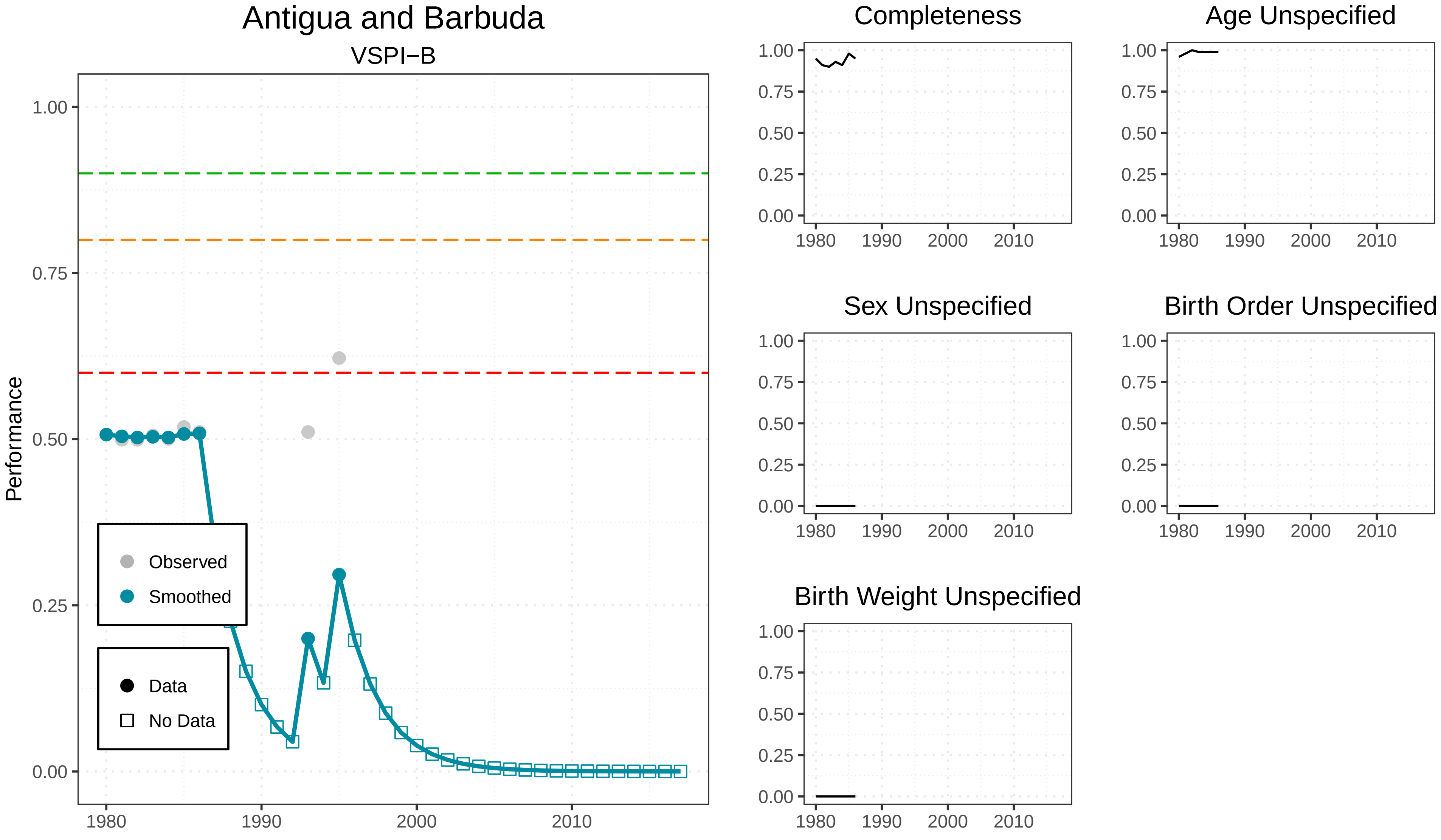

Antigua and Barbuda
VSPI−B
Performance
1.00
0.75
0.50
0.25
0.00
1980
1990
2000
2010
Observed
Smoothed
Data
No Data
Completeness
Age Unspecified
Sex Unspecified
Birth Order Unspecified
Birth Weight Unspecified

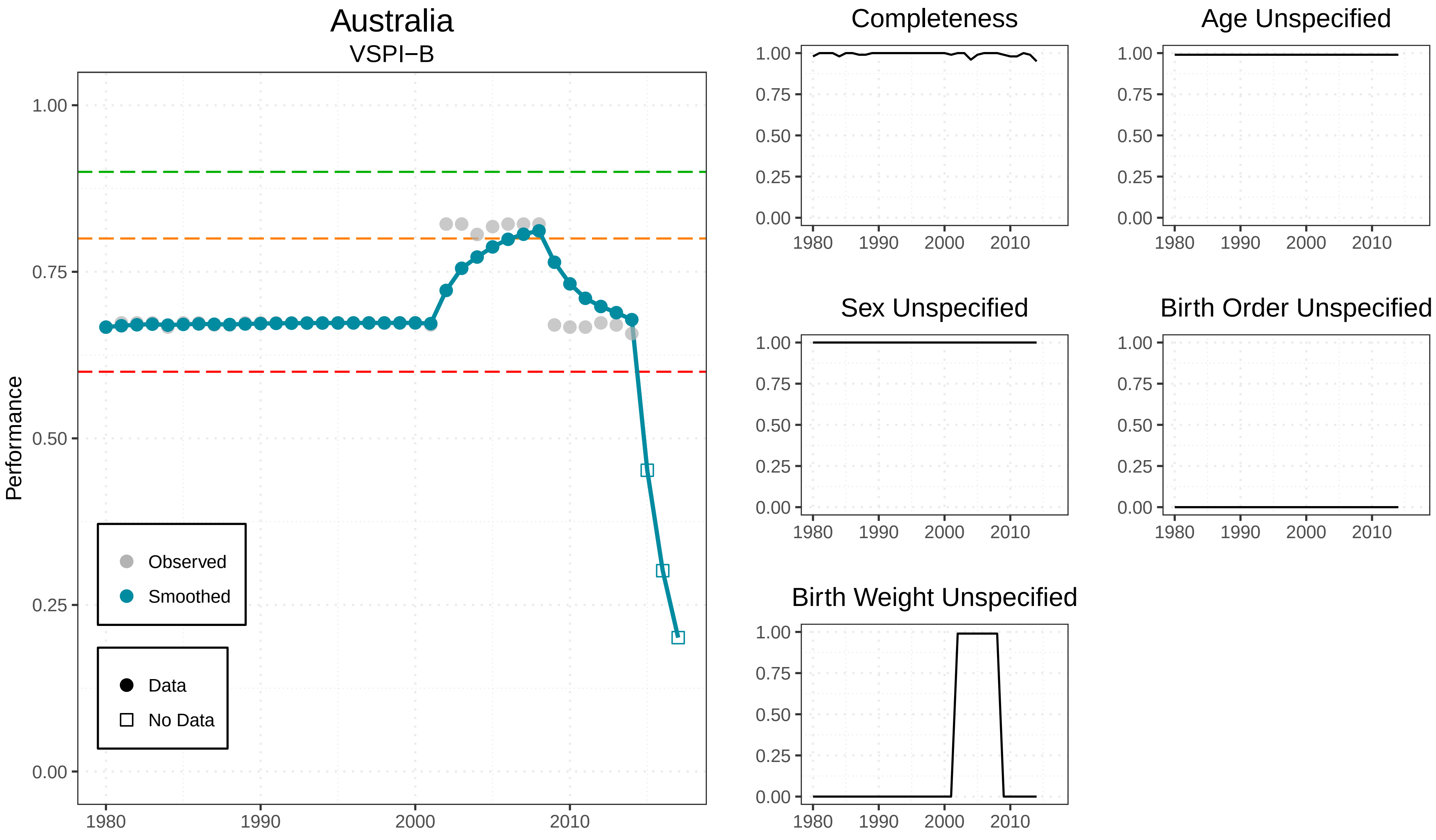
Australia
VSPI−B
Performance
1.00
0.75
0.50
0.25
0.00
1980
1990
2000
2010
Observed
Smoothed
Data
No Data
Completeness
Age Unspecified
Sex Unspecified
Birth Order Unspecified
Birth Weight Unspecified

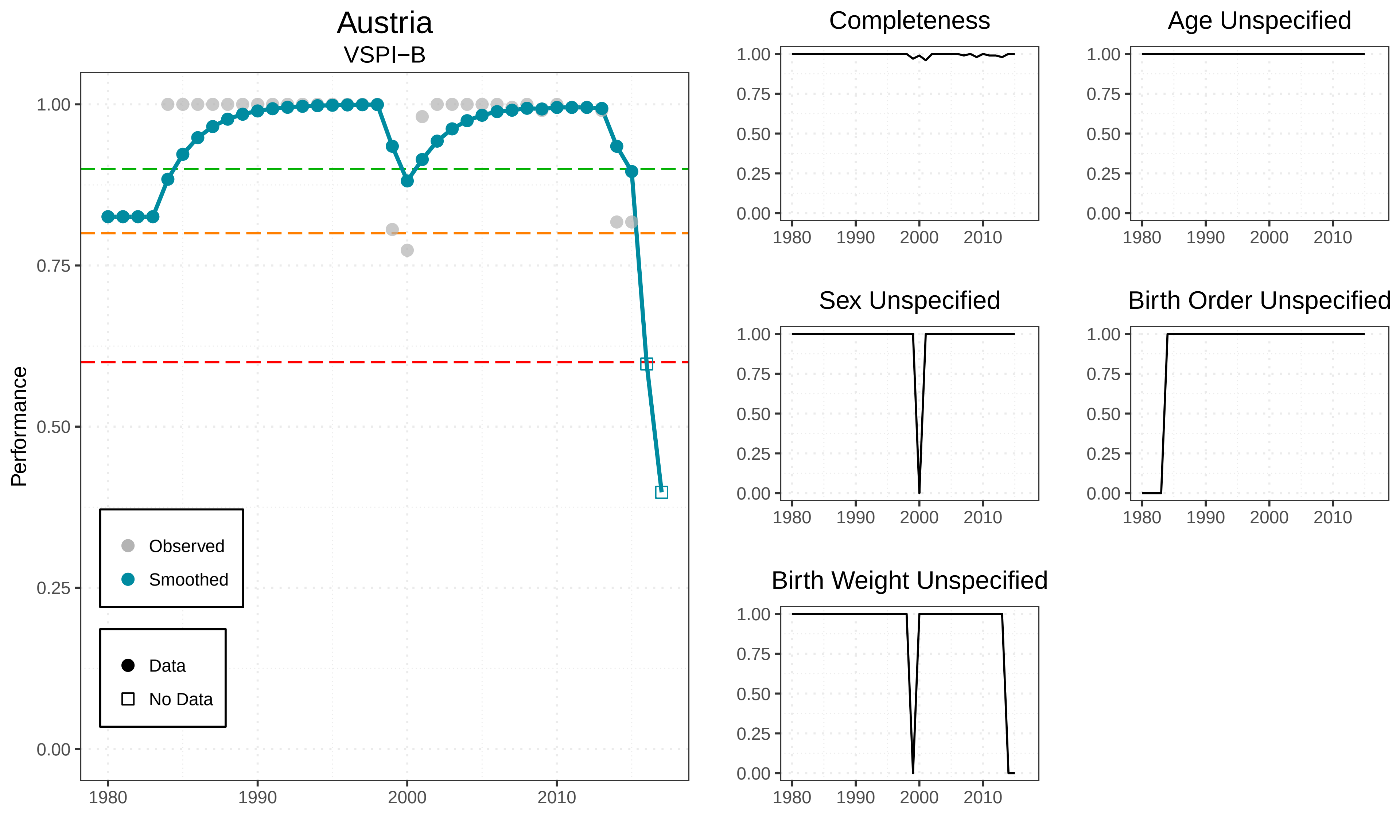

Austria
VSPI−B
Performance
1.00
0.75
0.50
0.25
0.00
1980
1990
2000
2010
Observed
Smoothed
Data
No Data
Completeness
Age Unspecified
Sex Unspecified
Birth Order Unspecified
Birth Weight Unspecified

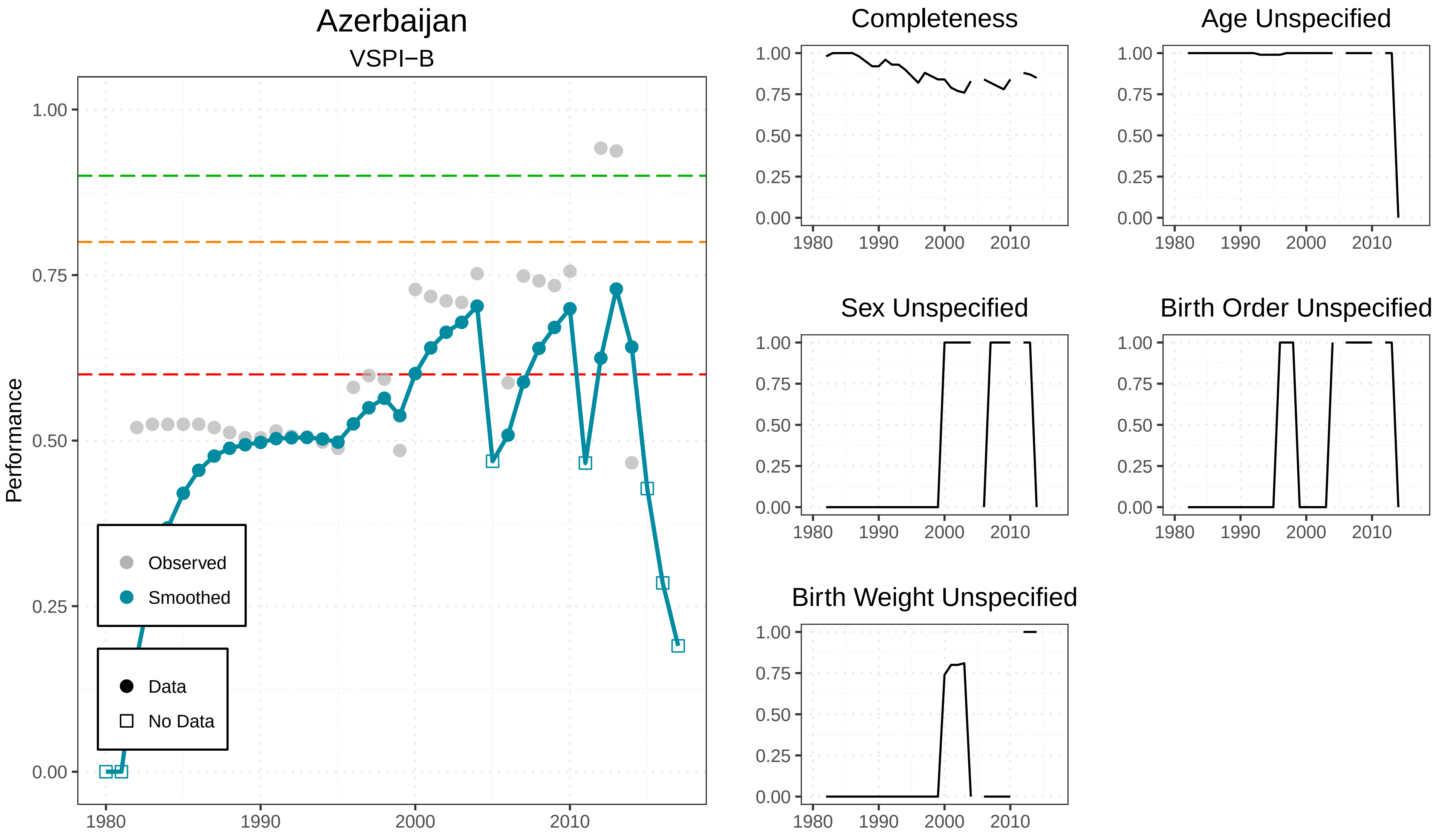

Azerbaijan
VSPI−B
Performance
Observed
Smoothed
Data
No Data
Completeness
Age Unspecified
Sex Unspecified
Birth Order Unspecified
Birth Weight Unspecified
1.00
0.75
0.50
0.25
0.00
1980
1990
2000
2010

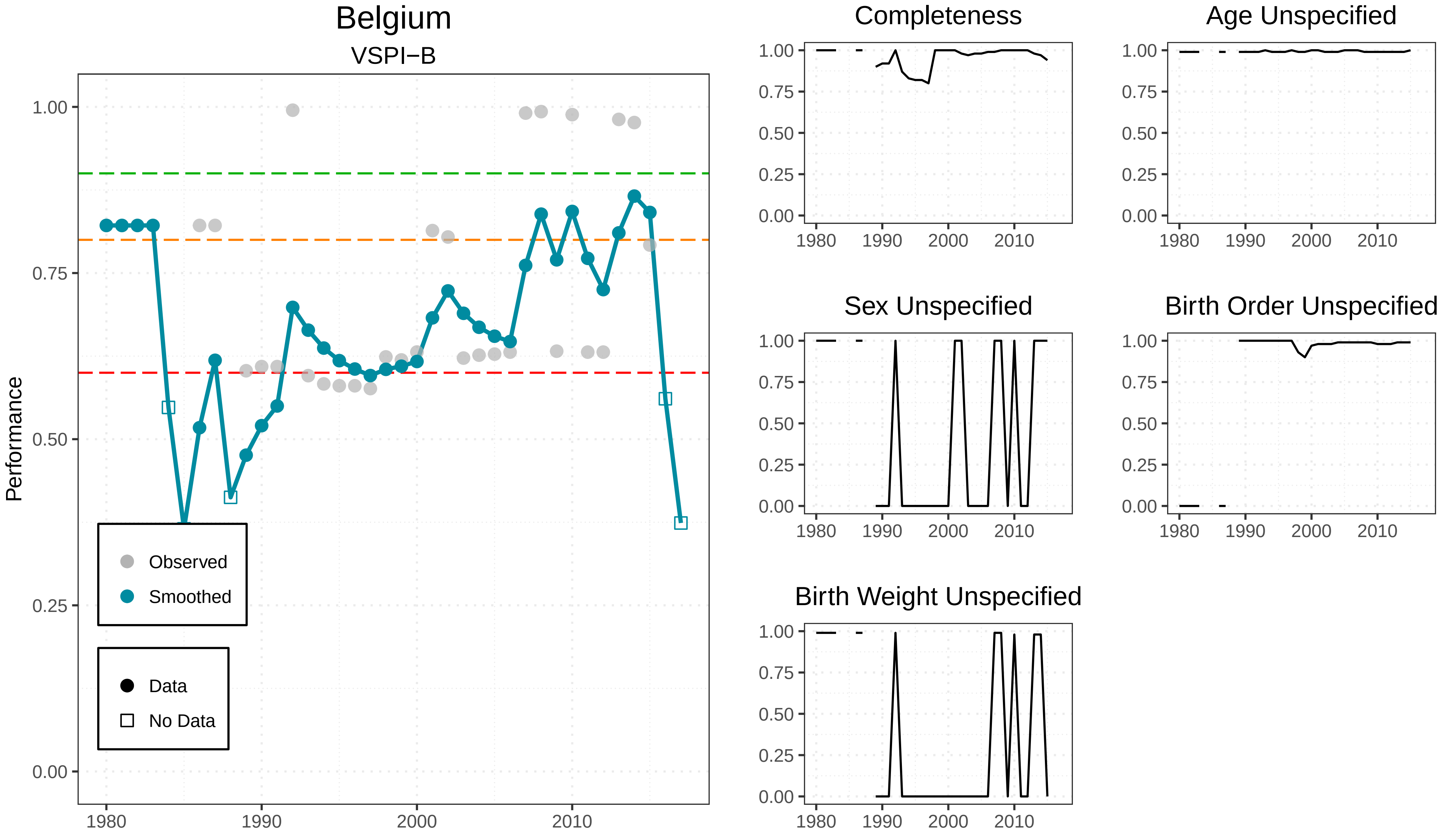

Belgium
VSPI−B
Performance
1.00
0.75
0.50
0.25
0.00
1980
1990
2000
2010
Observed
Smoothed
Data
No Data
Completeness
Age Unspecified
Sex Unspecified
Birth Order Unspecified
Birth Weight Unspecified

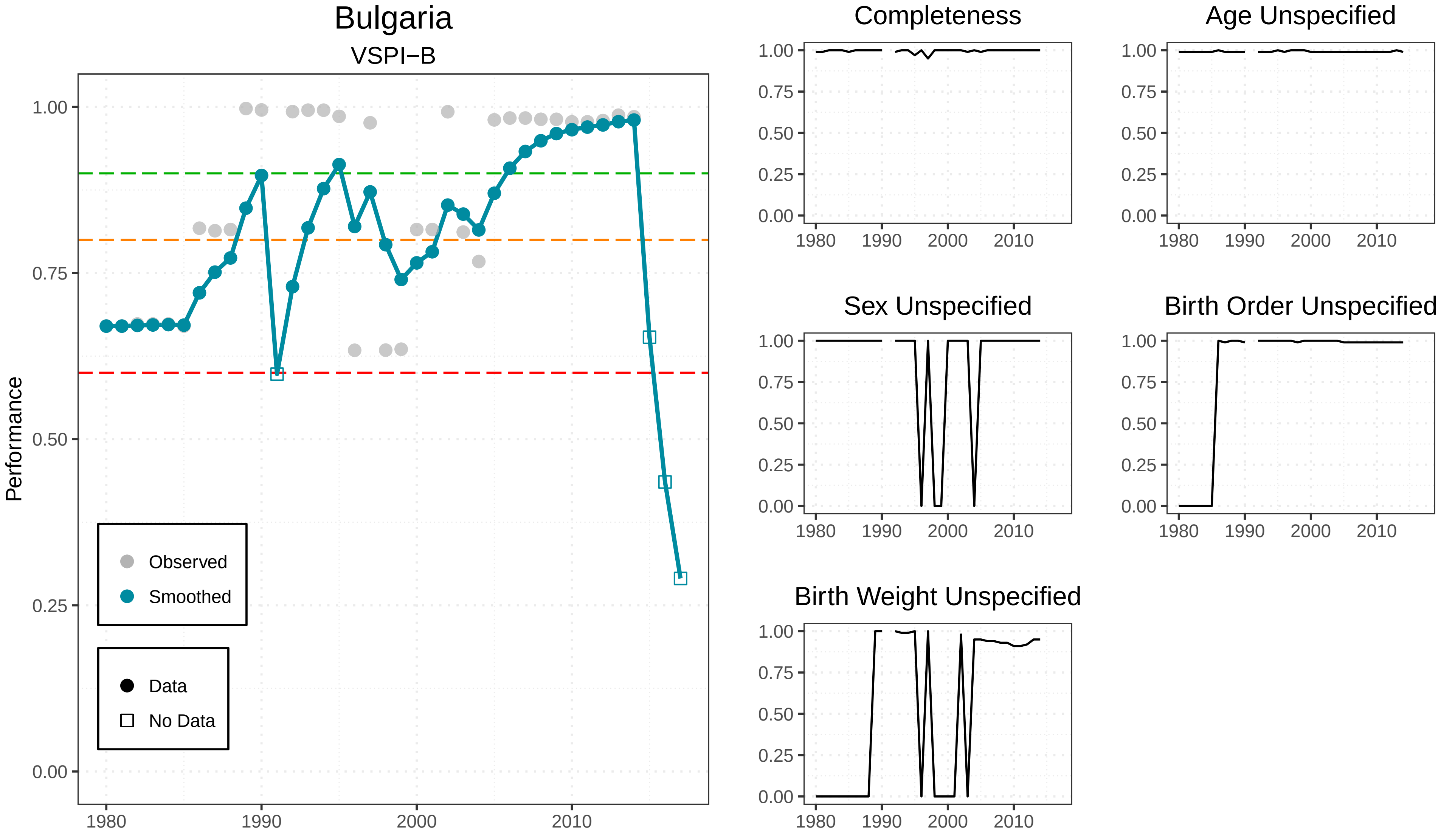

Bulgaria
VSPI−B
Performance
1.00
0.75
0.50
0.25
0.00
1980
1990
2000
2010
Observed
Smoothed
Data
No Data
Completeness
Age Unspecified
Sex Unspecified
Birth Order Unspecified
Birth Weight Unspecified

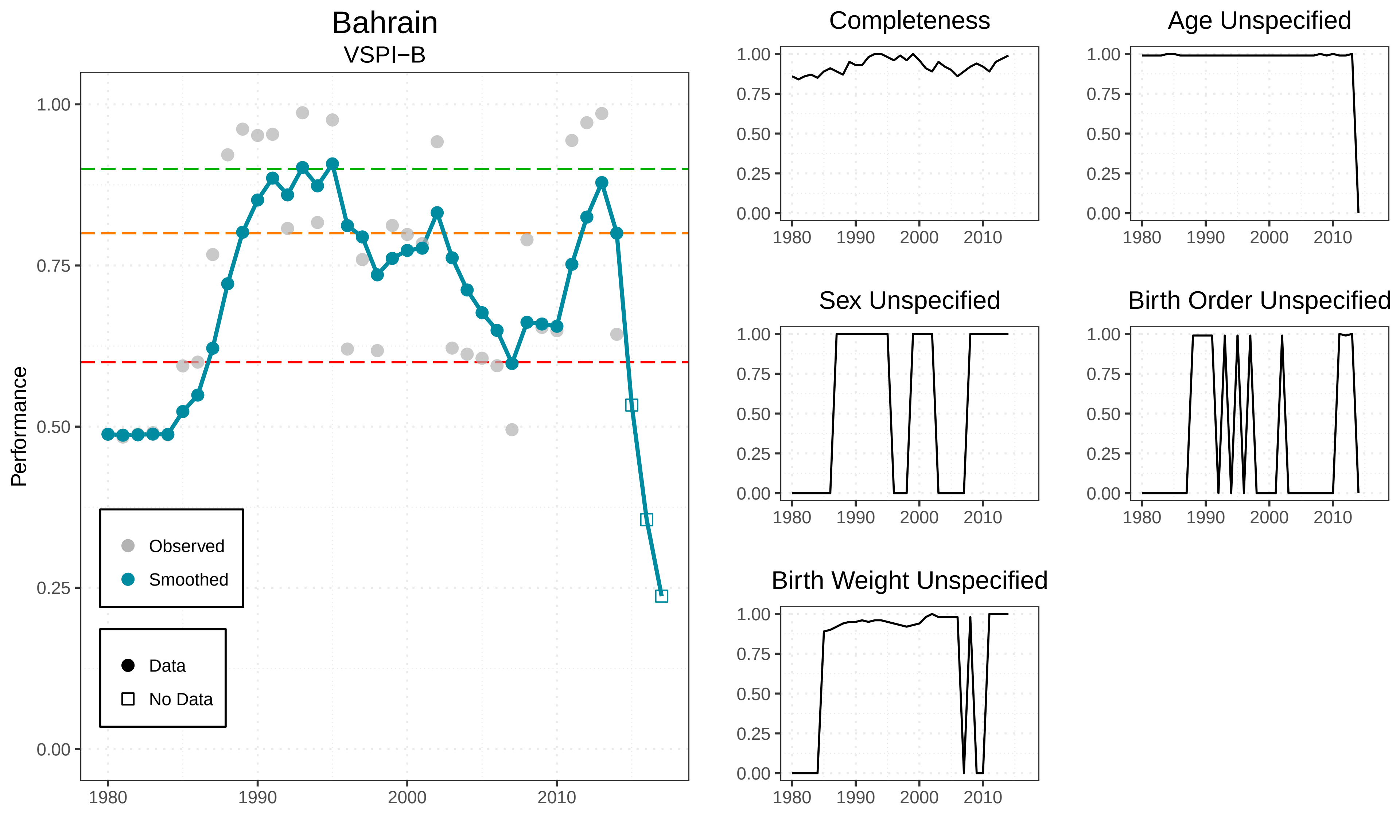

Bahrain
VSPI−B
Performance
1.00
0.75
0.50
0.25
0.00
1980
1990
2000
2010
Observed
Smoothed
Data
No Data
Completeness
Age Unspecified
Sex Unspecified
Birth Order Unspecified
Birth Weight Unspecified

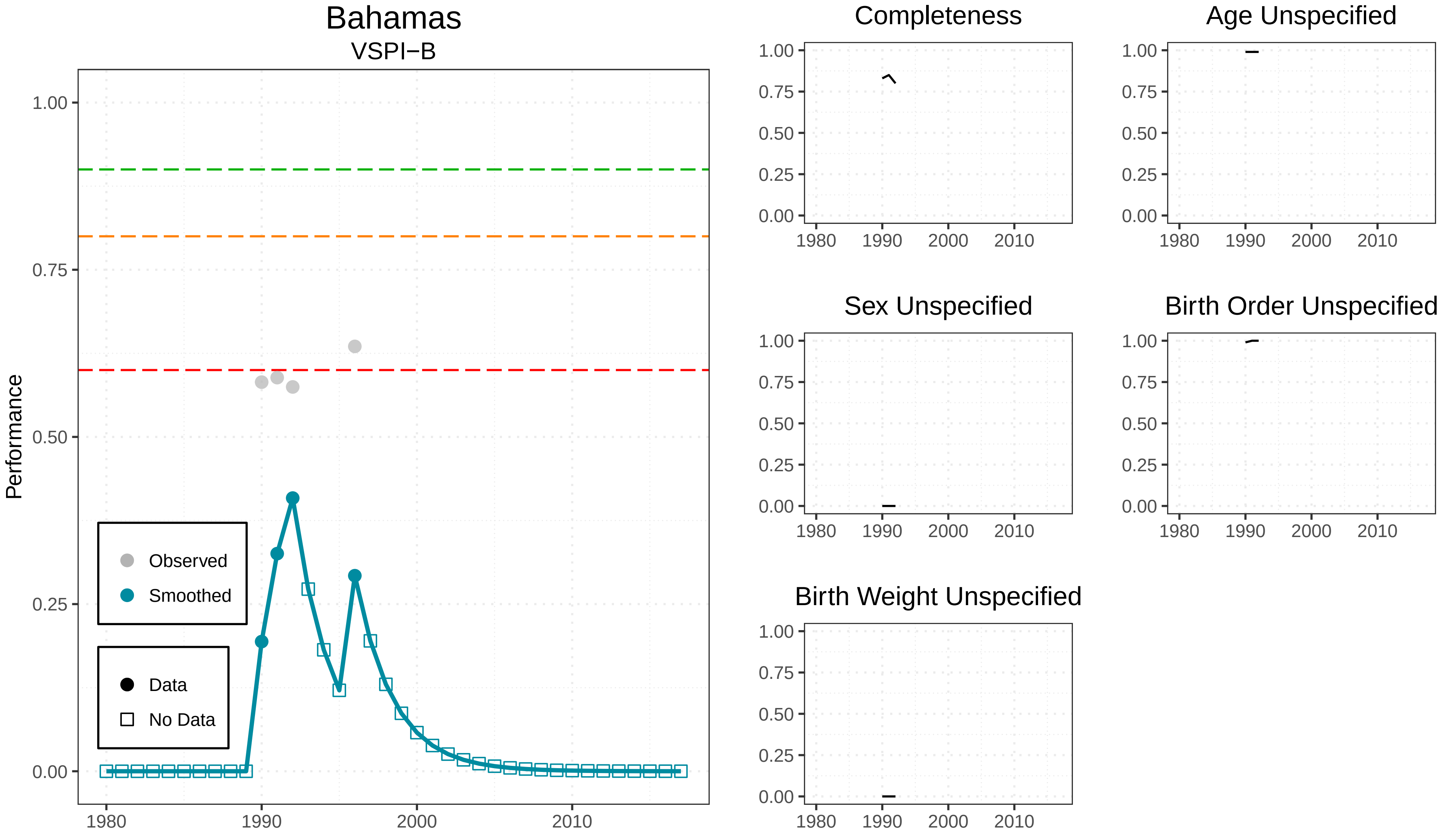
Bahamas
VSPI−B
Performance
1.00
0.75
0.50
0.25
0.00
1980
1990
2000
2010
Observed
Smoothed
Data
No Data
Completeness
Age Unspecified
Sex Unspecified
Birth Order Unspecified
Birth Weight Unspecified

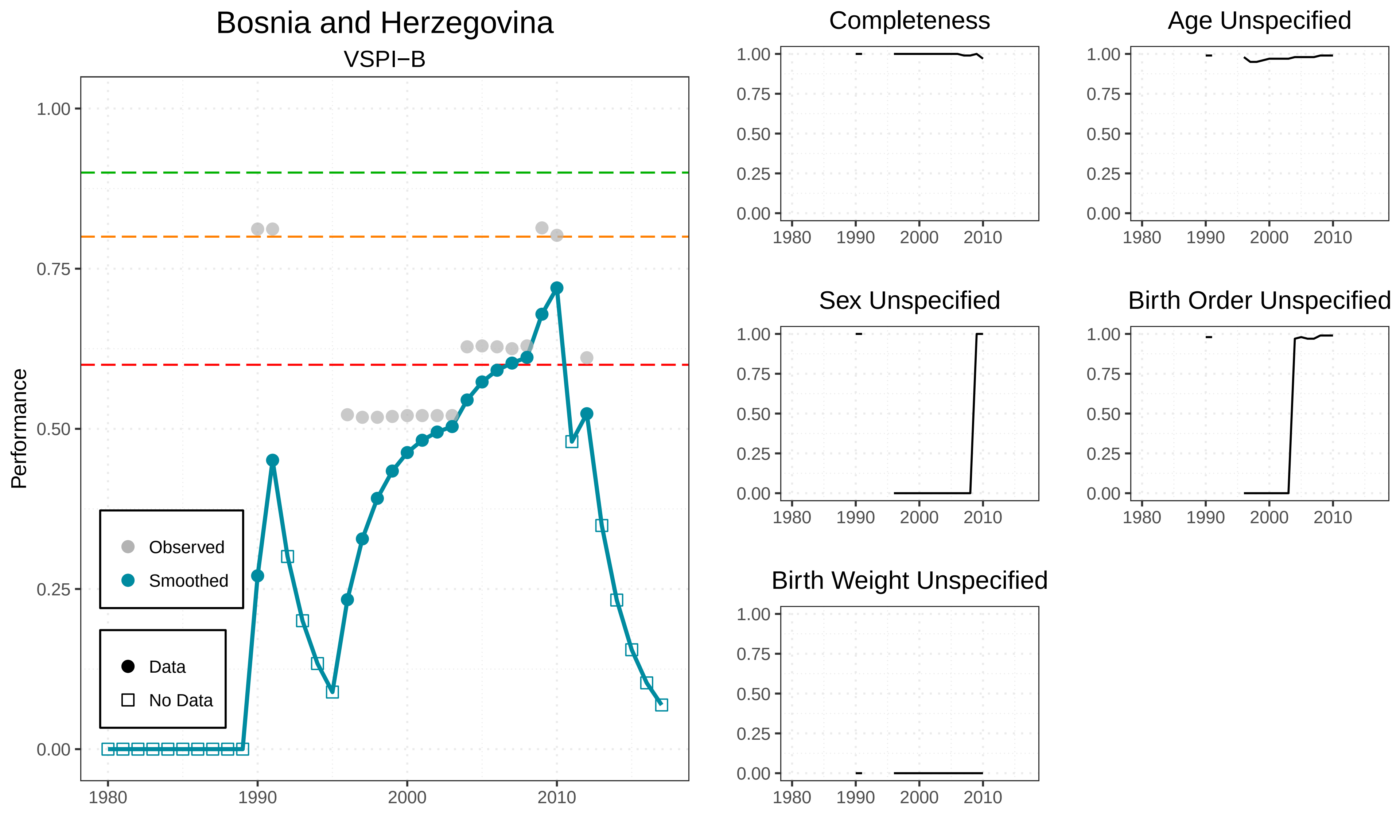

Bosnia and Herzegovina
VSPI−B
Performance
Observed
Smoothed
Data
No Data
Completeness
Age Unspecified
Sex Unspecified
Birth Order Unspecified
Birth Weight Unspecified
1.00
0.75
0.50
0.25
0.00
1980
1990
2000
2010

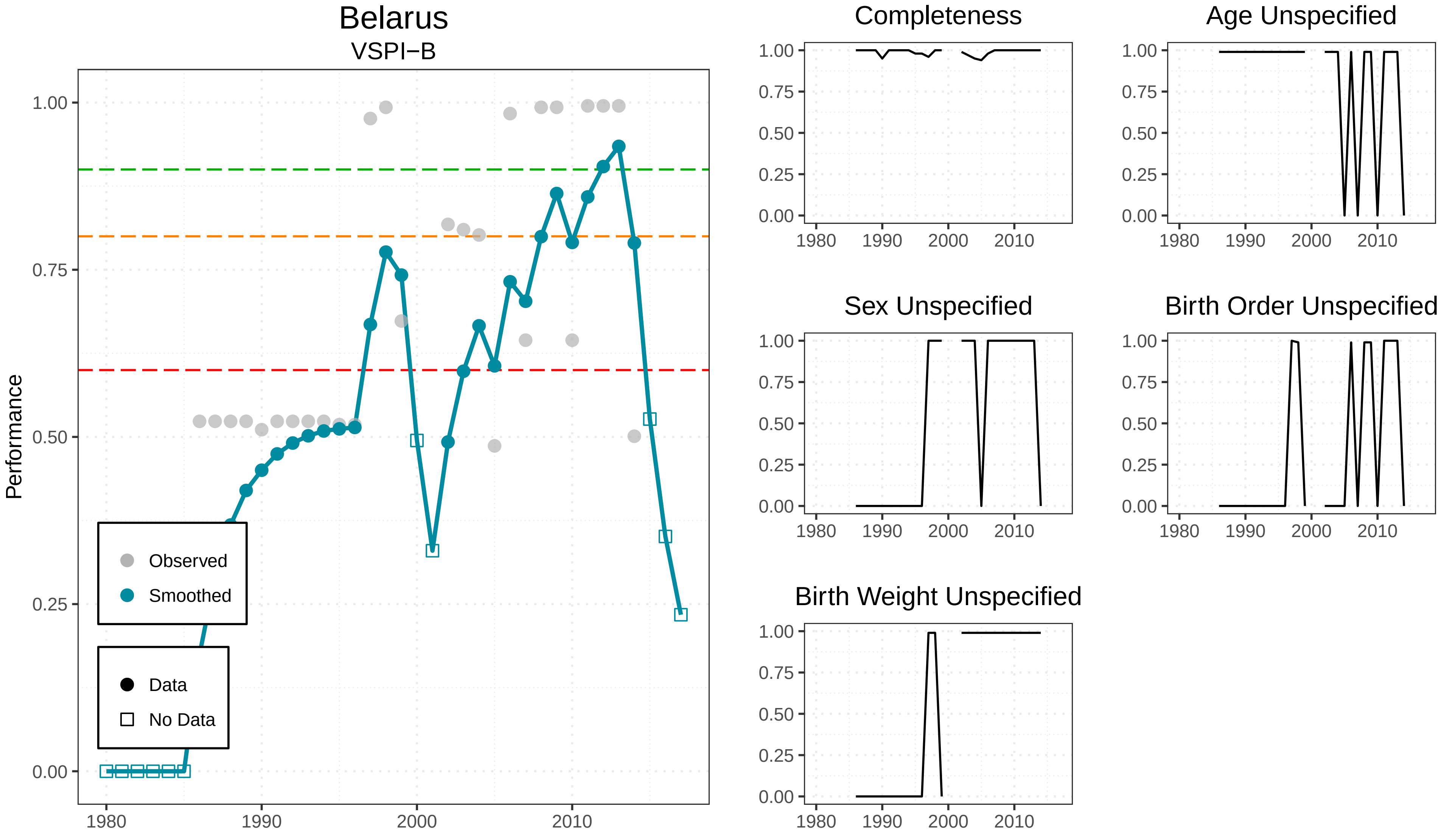

Belarus
VSPI−B
Performance
Observed
Smoothed
Data
No Data
Completeness
Age Unspecified
Sex Unspecified
Birth Order Unspecified
Birth Weight Unspecified

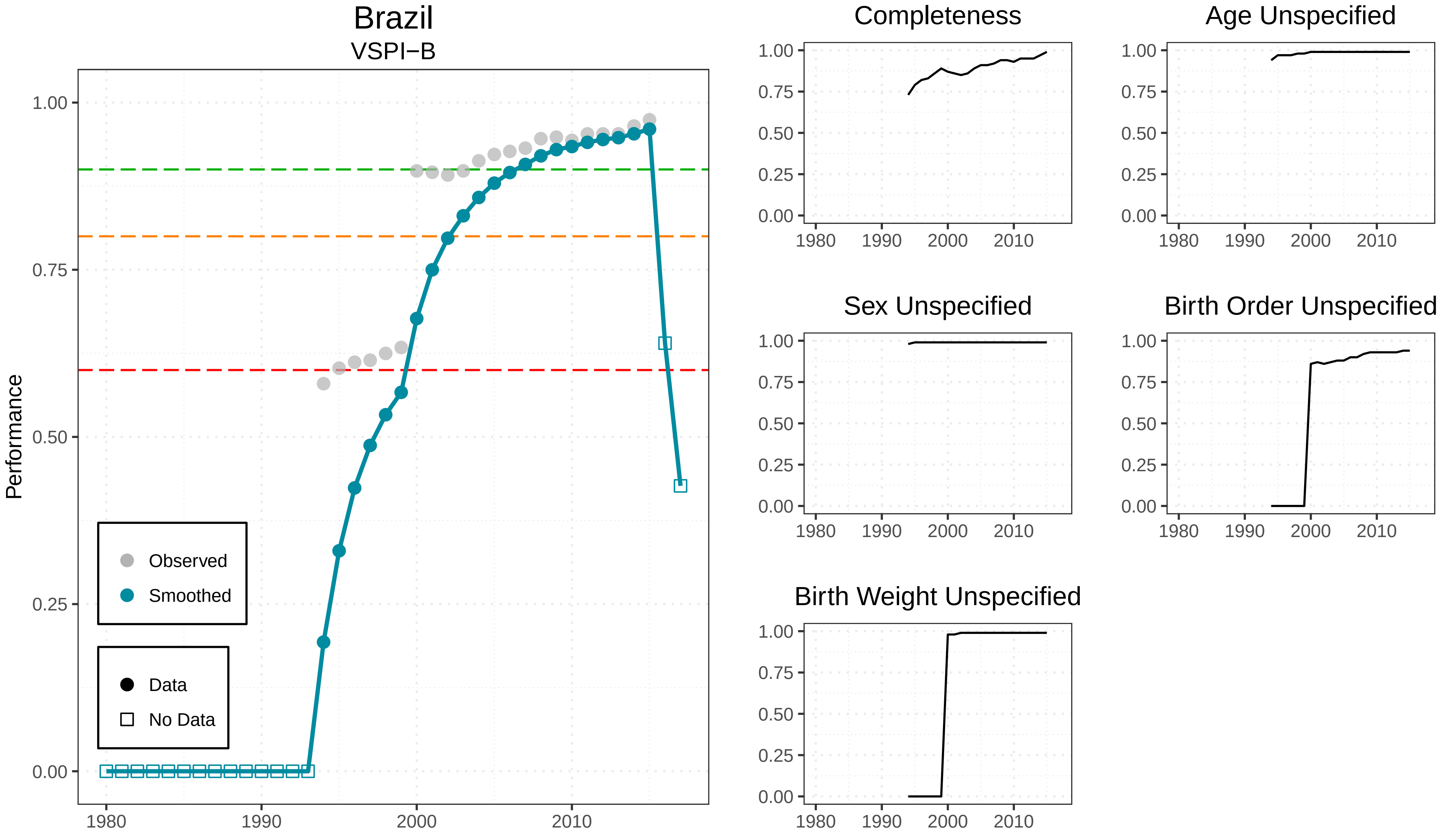

Brazil
VSPI−B
Performance
1.00
0.75
0.50
0.25
0.00
1980
1990
2000
2010
Observed
Smoothed
Data
No Data
Completeness
Age Unspecified
Sex Unspecified
Birth Order Unspecified
Birth Weight Unspecified

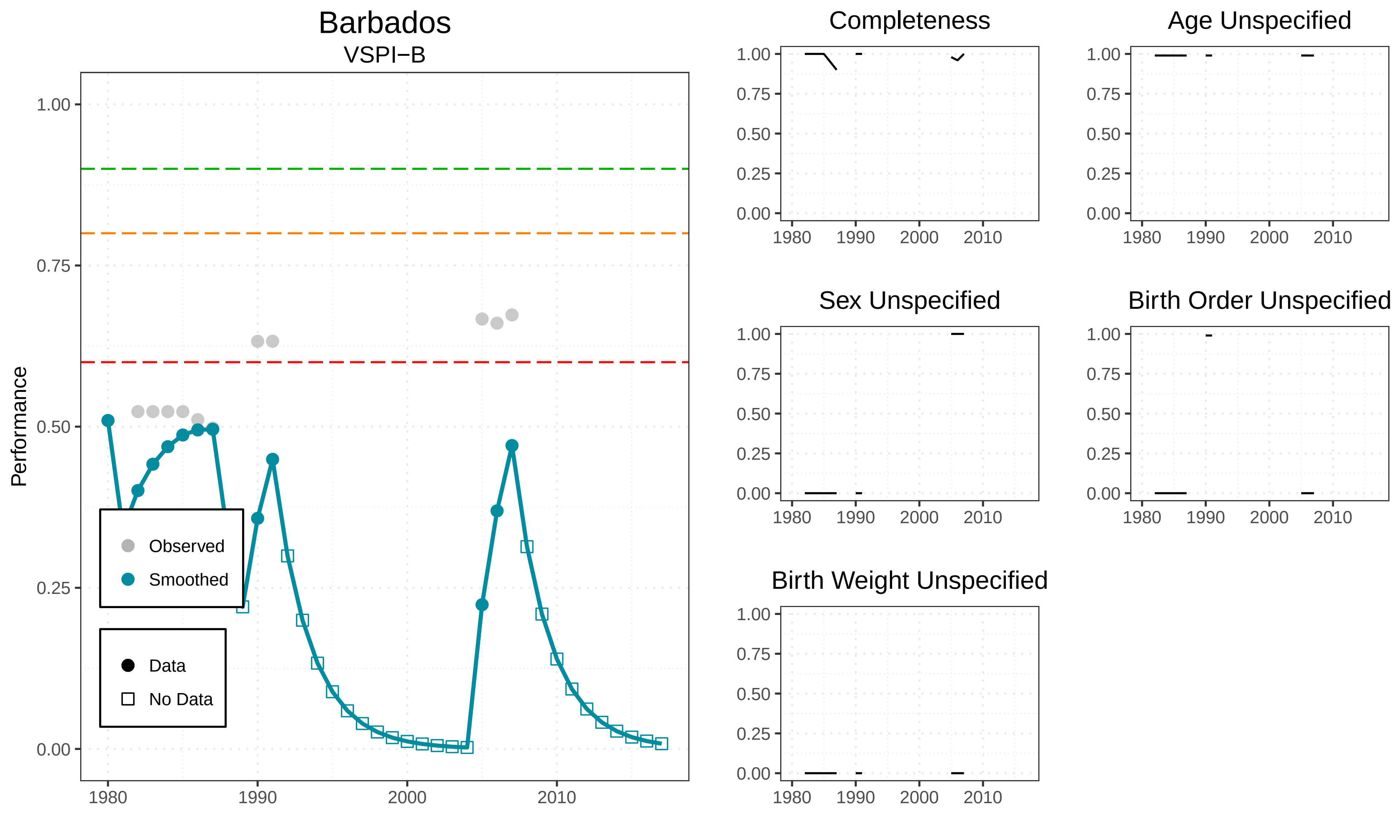

Barbados
VSPI−B
Performance
1.00
0.75
0.50
0.25
0.00
1980
1990
2000
2010
Observed
Smoothed
Data
No Data
Completeness
Age Unspecified
Sex Unspecified
Birth Order Unspecified
Birth Weight Unspecified

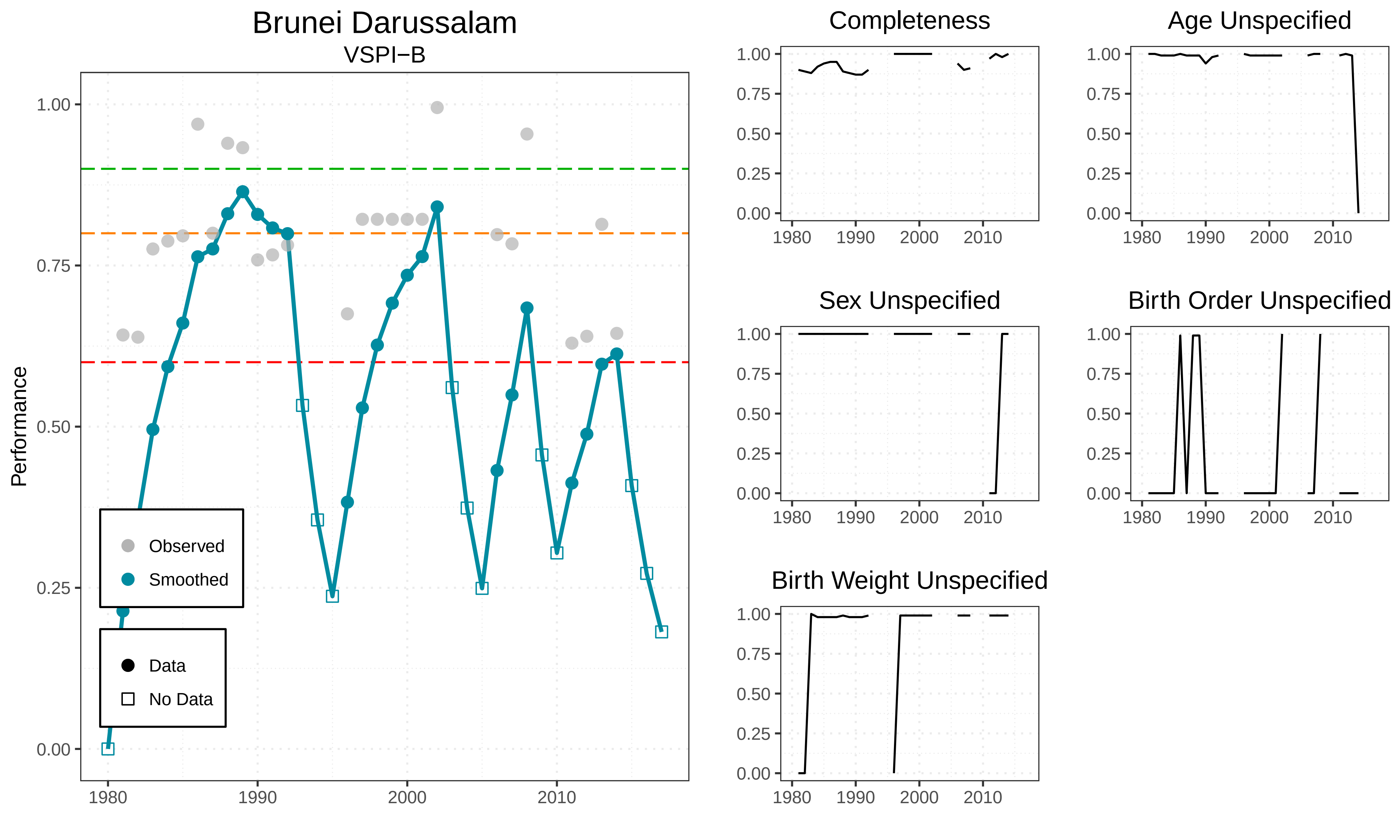

Brunei Darussalam
VSPI−B
Performance
1.00
0.75
0.50
0.25
0.00
1980
1990
2000
2010
Observed
Smoothed
Data
No Data
Completeness
Age Unspecified
Sex Unspecified
Birth Order Unspecified
Birth Weight Unspecified

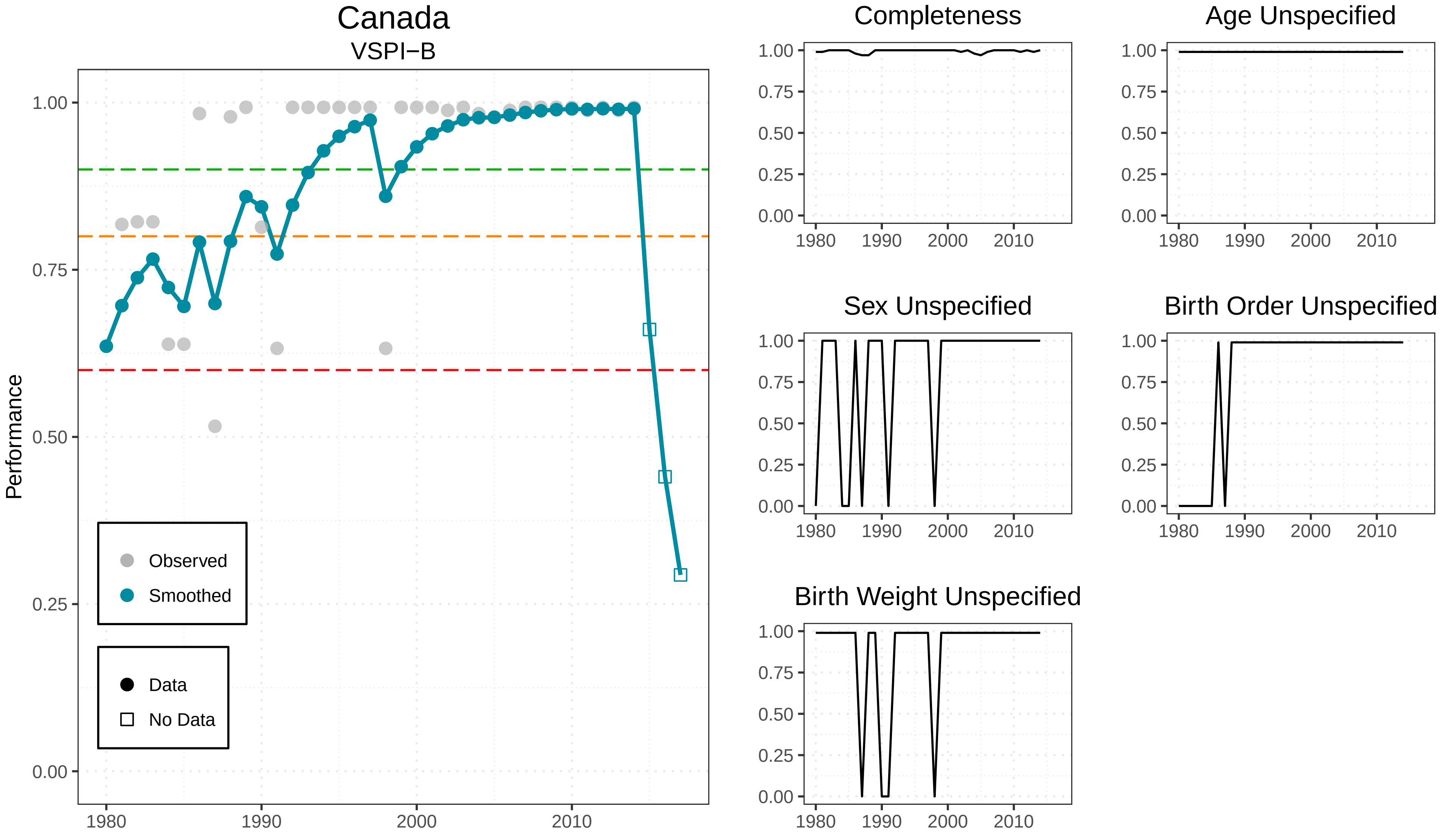
Canada
VSPI−B
Performance
Observed
Smoothed
Data
No Data
Completeness
Age Unspecified
Sex Unspecified
Birth Order Unspecified
Birth Weight Unspecified

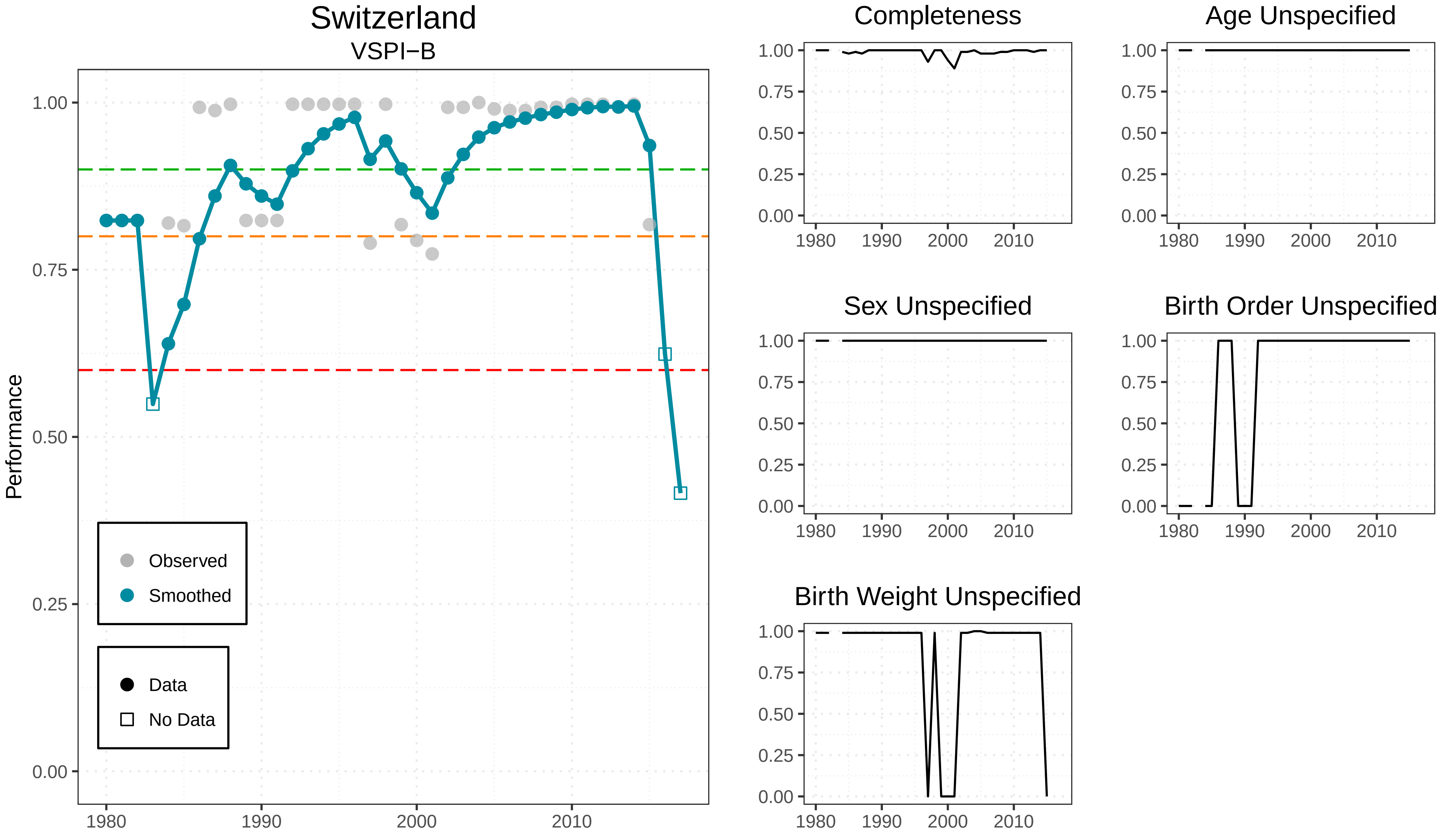

Switzerland
VSPI−B
Performance
1.00
0.75
0.50
0.25
0.00
1980
1990
2000
2010
Observed
Smoothed
Data
No Data
Completeness
Age Unspecified
Sex Unspecified
Birth Order Unspecified
Birth Weight Unspecified

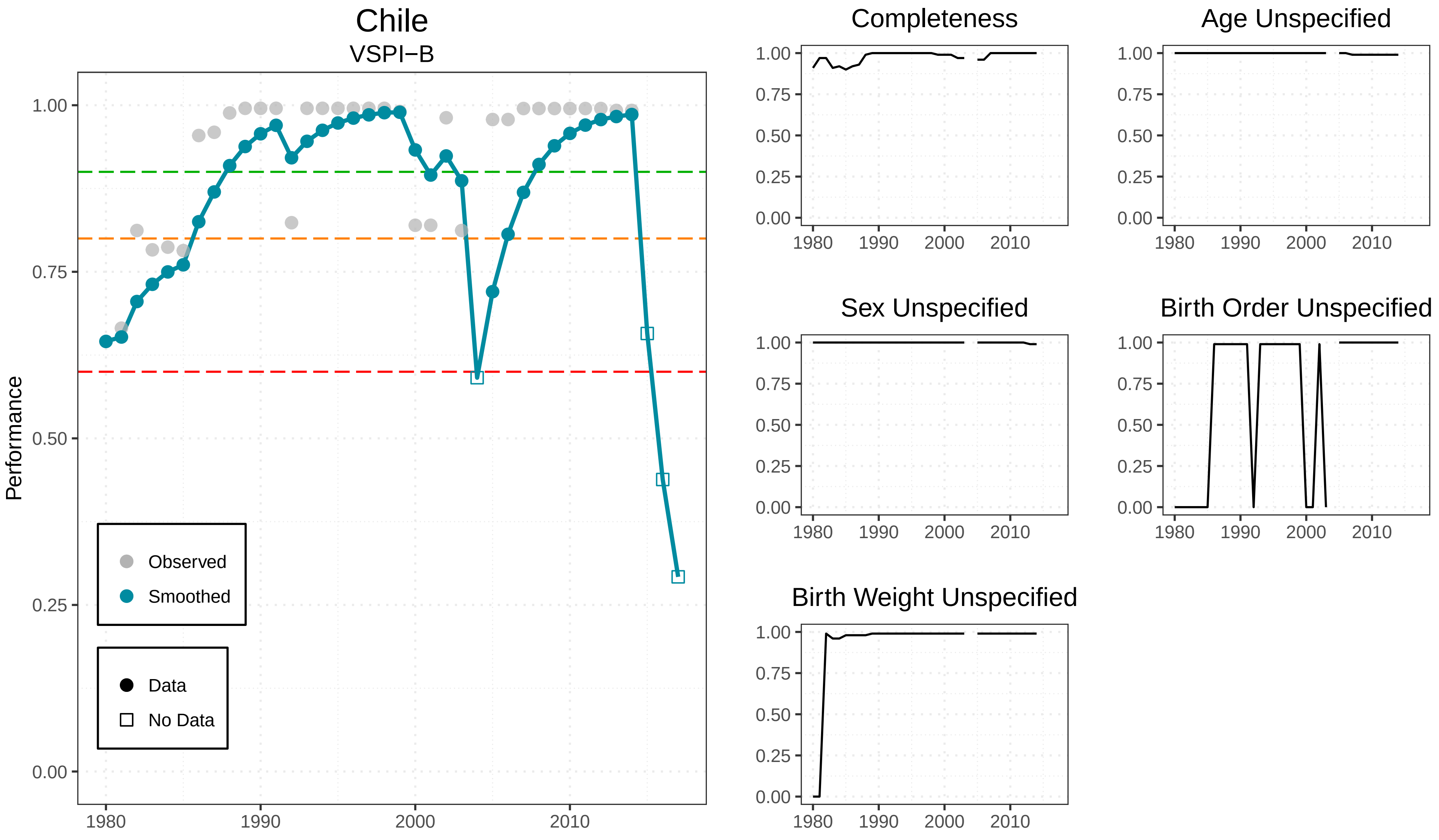

Chile
VSPI−B
Performance
1.00
0.75
0.50
0.25
0.00
1980
1990
2000
2010
Observed
Smoothed
Data
No Data
Completeness
Age Unspecified
Sex Unspecified
Birth Order Unspecified
Birth Weight Unspecified

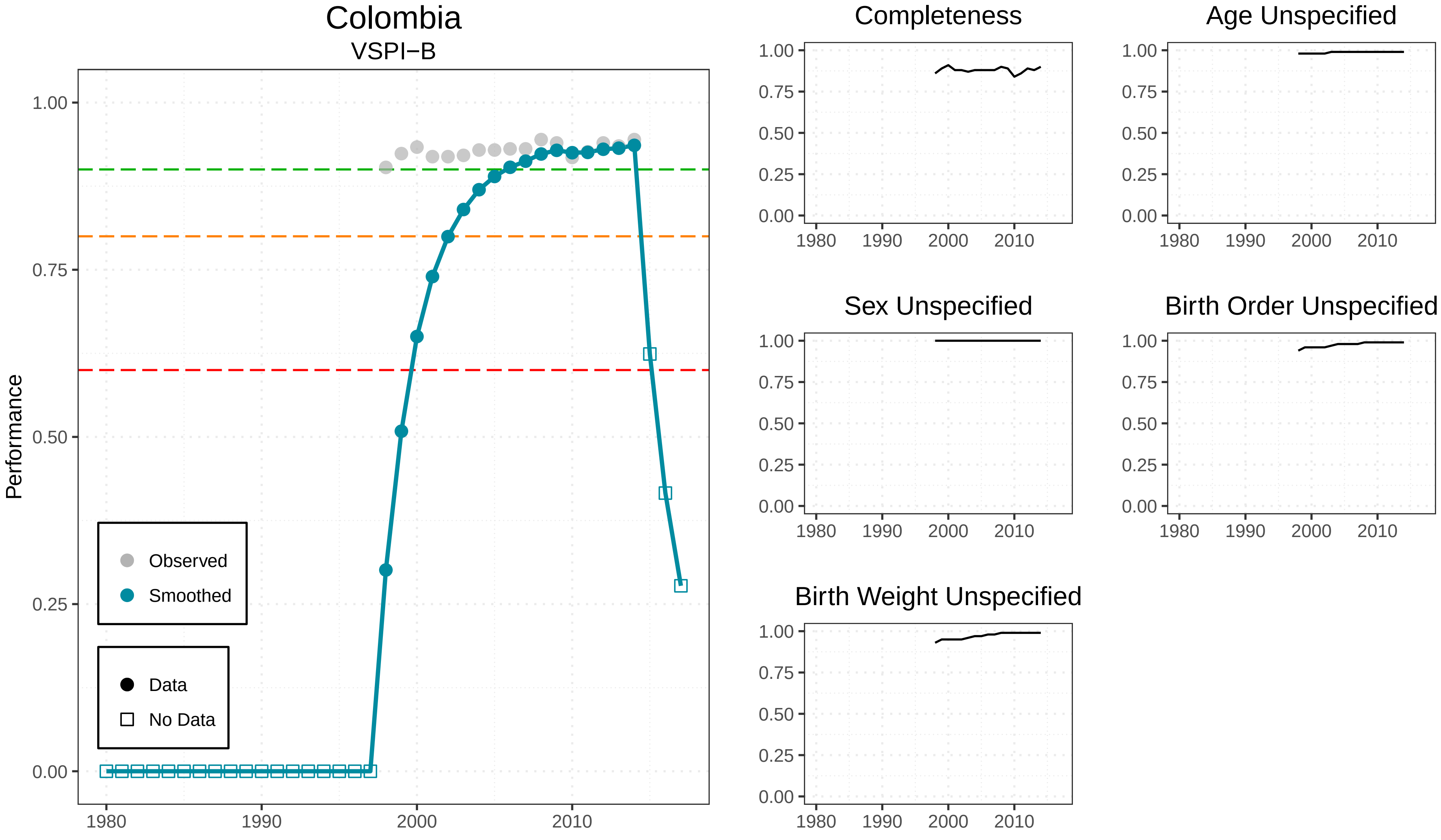

Colombia
VSPI−B
Performance
1.00
0.75
0.50
0.25
0.00
1980
1990
2000
2010
Observed
Smoothed
Data
No Data
Completeness
Age Unspecified
Sex Unspecified
Birth Order Unspecified
Birth Weight Unspecified

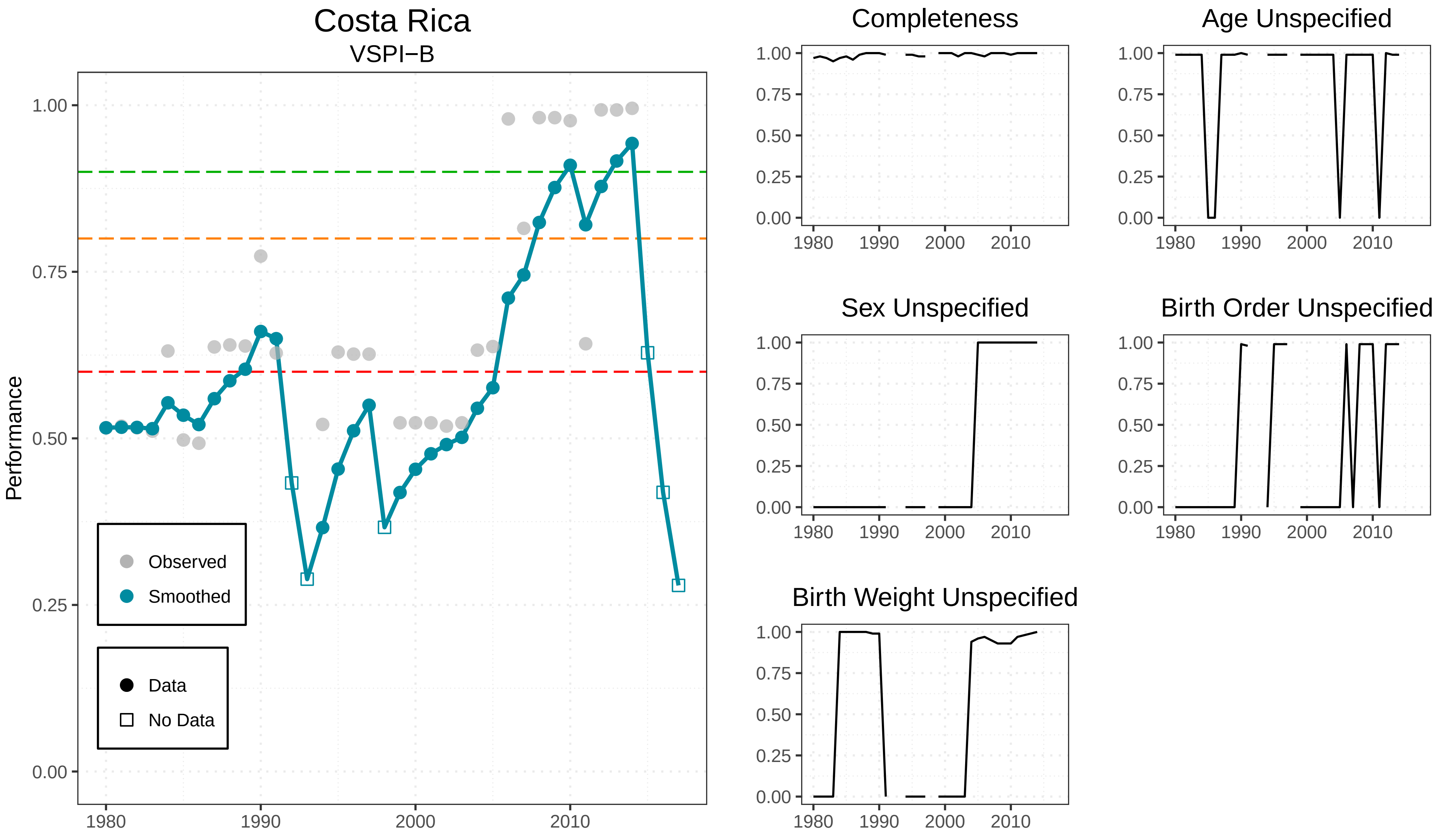
Costa Rica
VSPI−B
Performance
1.00
0.75
0.50
0.25
0.00
1980
1990
2000
2010
Observed
Smoothed
Data
No Data
Completeness
Age Unspecified
Sex Unspecified
Birth Order Unspecified
Birth Weight Unspecified

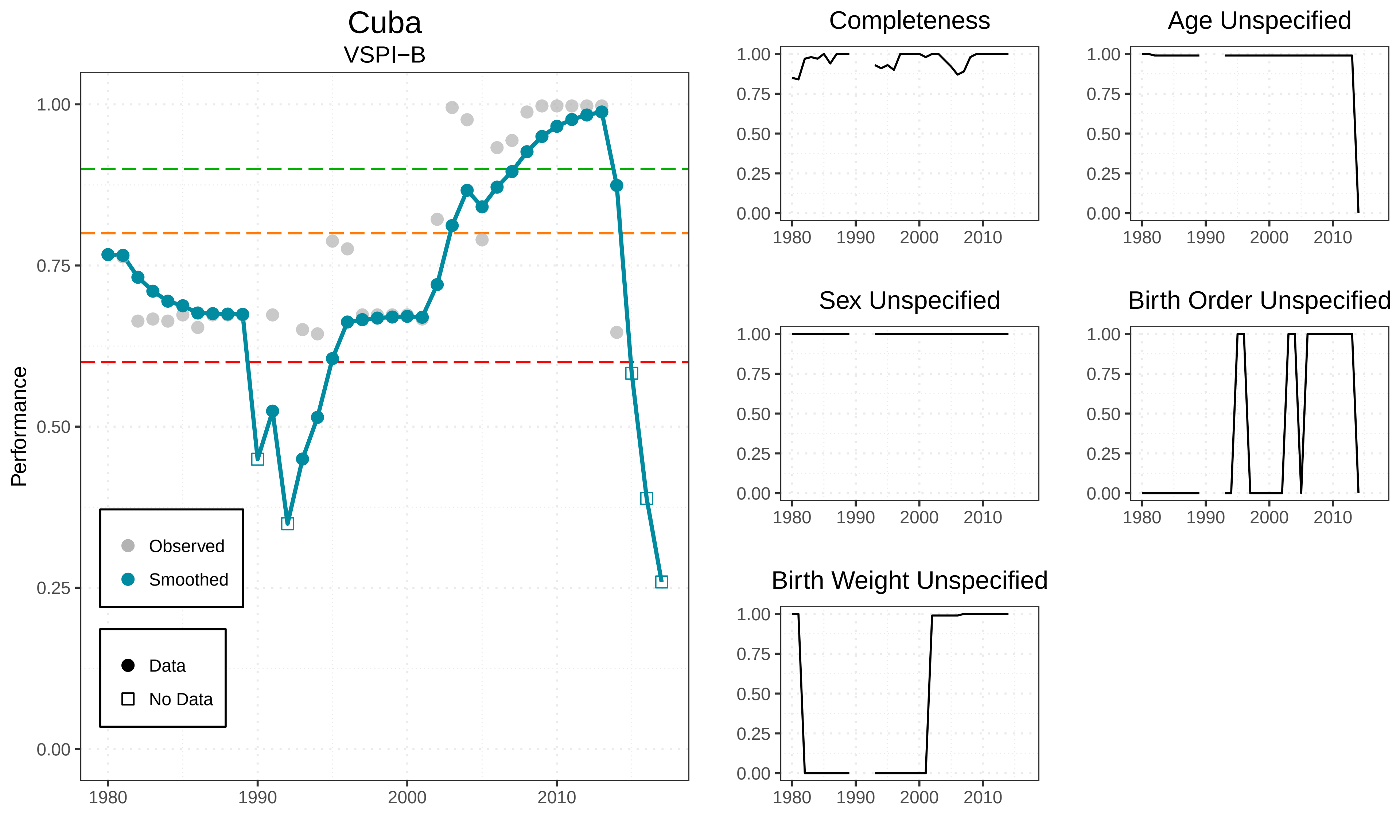

Cuba
VSPI−B
Performance
1.00
0.75
0.50
0.25
0.00
1980
1990
2000
2010
Observed
Smoothed
Data
No Data
Completeness
Age Unspecified
Sex Unspecified
Birth Order Unspecified
Birth Weight Unspecified

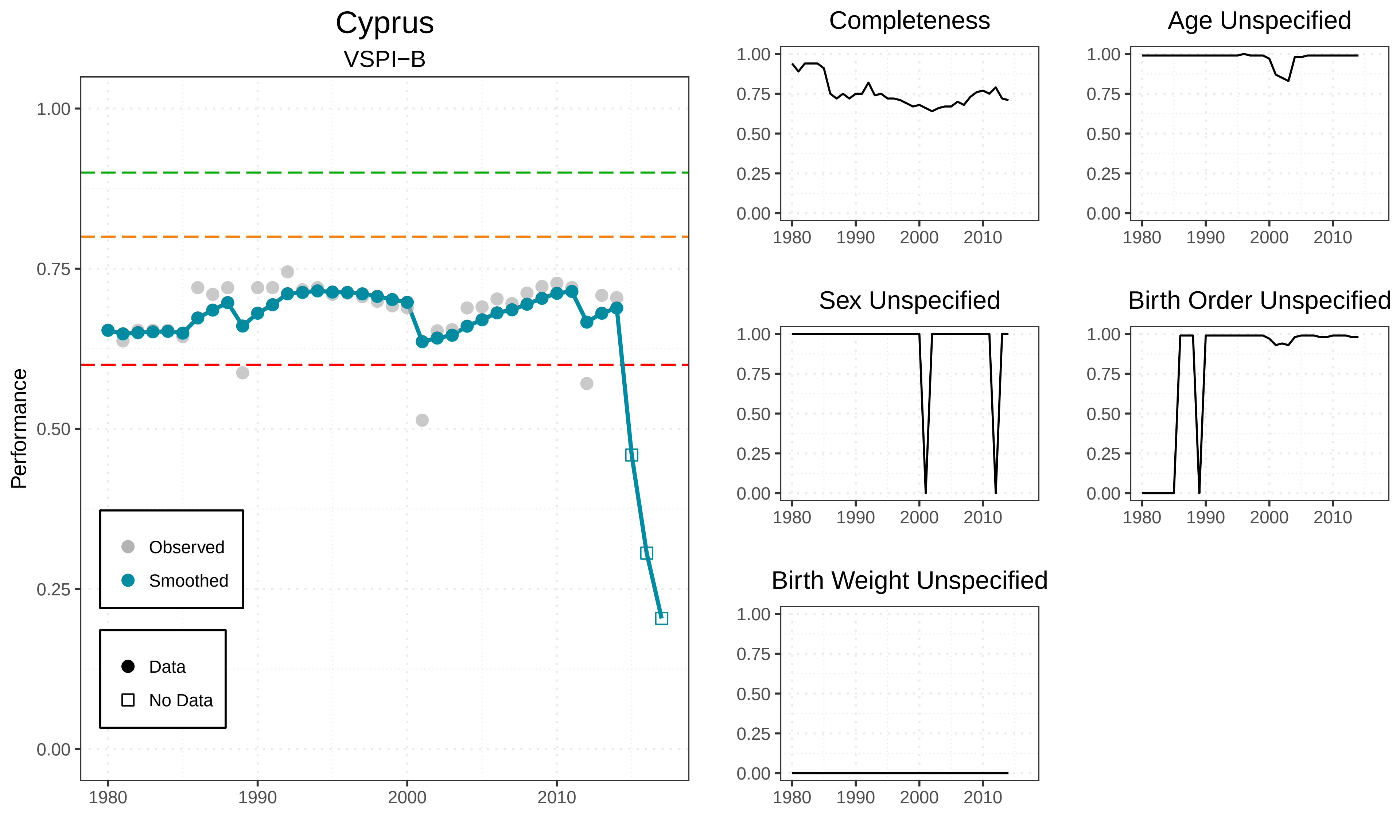
Cyprus
VSPI−B
Performance
1.00
0.75
0.50
0.25
0.00
1980
1990
2000
2010
Observed
Smoothed
Data
No Data
Completeness
Age Unspecified
Sex Unspecified
Birth Order Unspecified
Birth Weight Unspecified

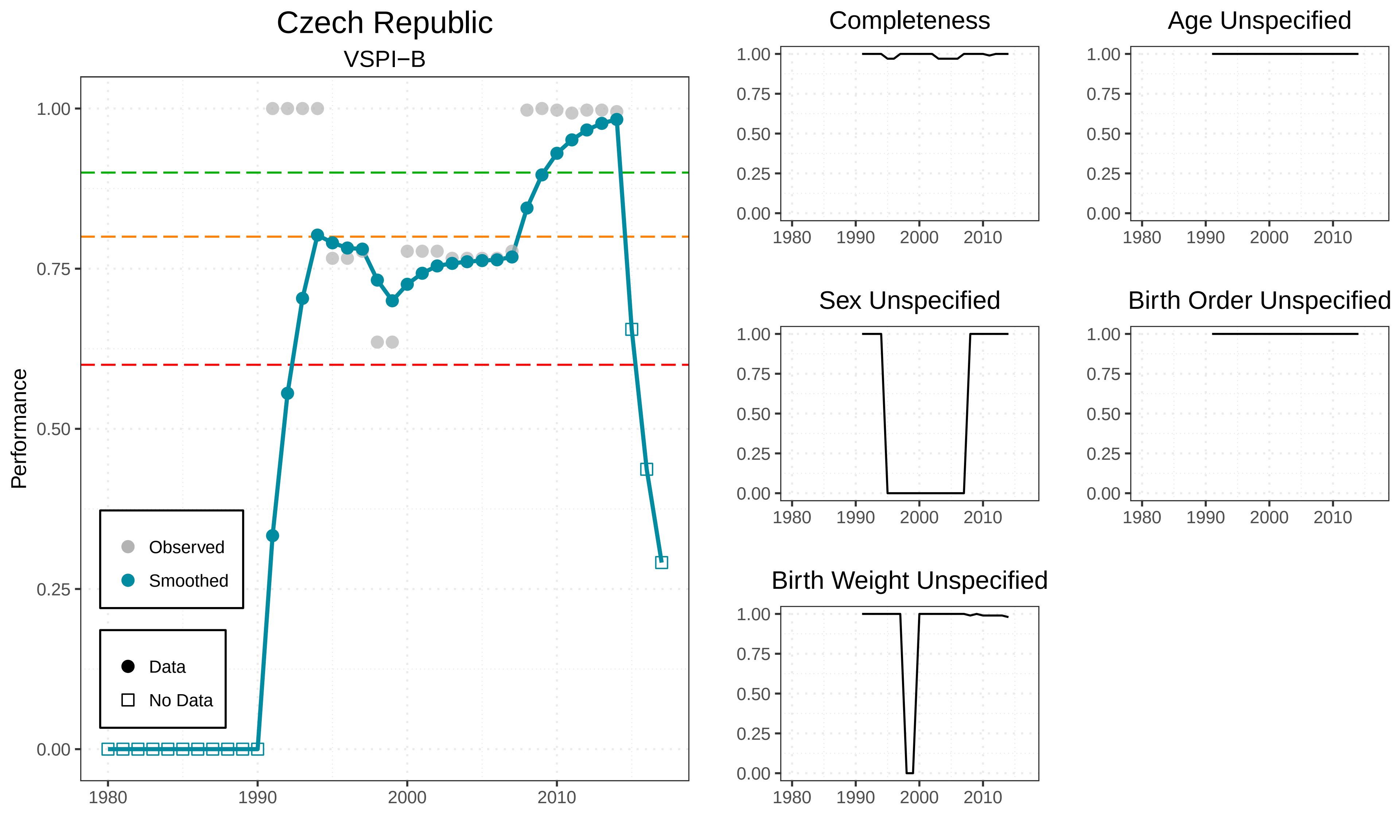

Czech Republic
VSPI−B
Performance
1.00
0.75
0.50
0.25
0.00
1980
1990
2000
2010
Observed
Smoothed
Data
No Data
Completeness
Age Unspecified
Sex Unspecified
Birth Order Unspecified
Birth Weight Unspecified

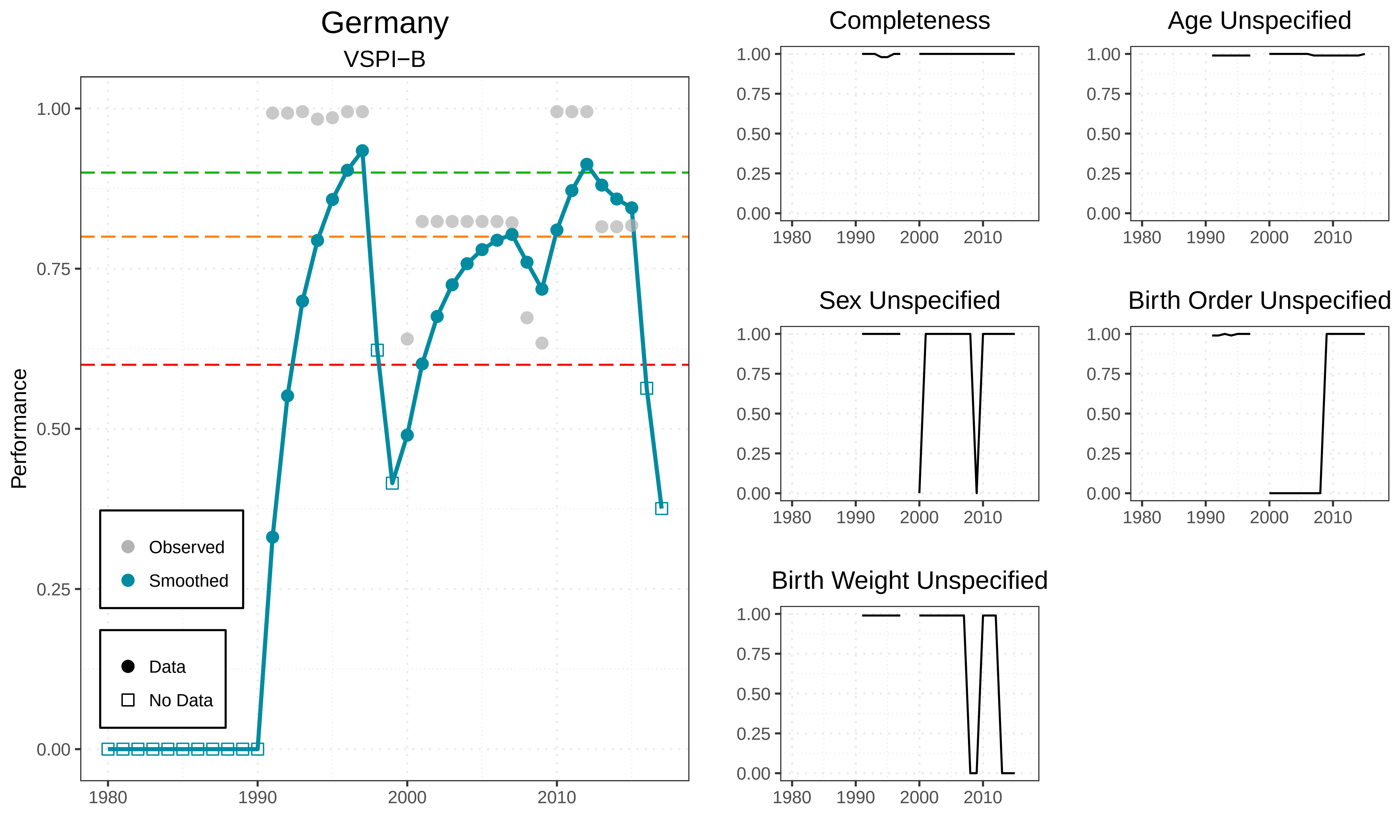
Germany
VSPI−B
Performance
Observed
Smoothed
Data
No Data
Completeness
Age Unspecified
Sex Unspecified
Birth Order Unspecified
Birth Weight Unspecified
1.00
0.75
0.50
0.25
0.00
1980
1990
2000
2010

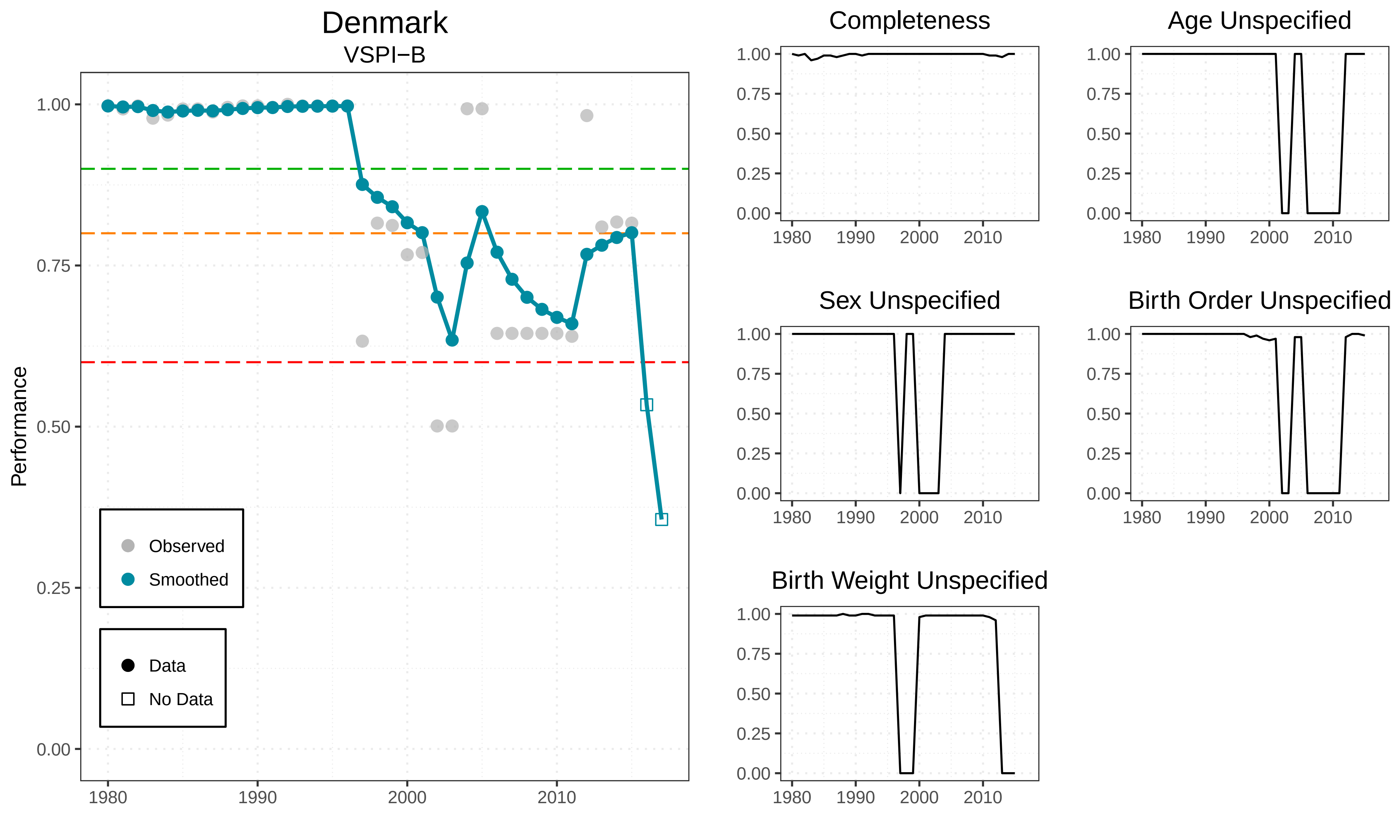
Denmark
VSPI−B
Performance
Observed
Smoothed
Data
No Data
Completeness
Age Unspecified
Sex Unspecified
Birth Order Unspecified
Birth Weight Unspecified

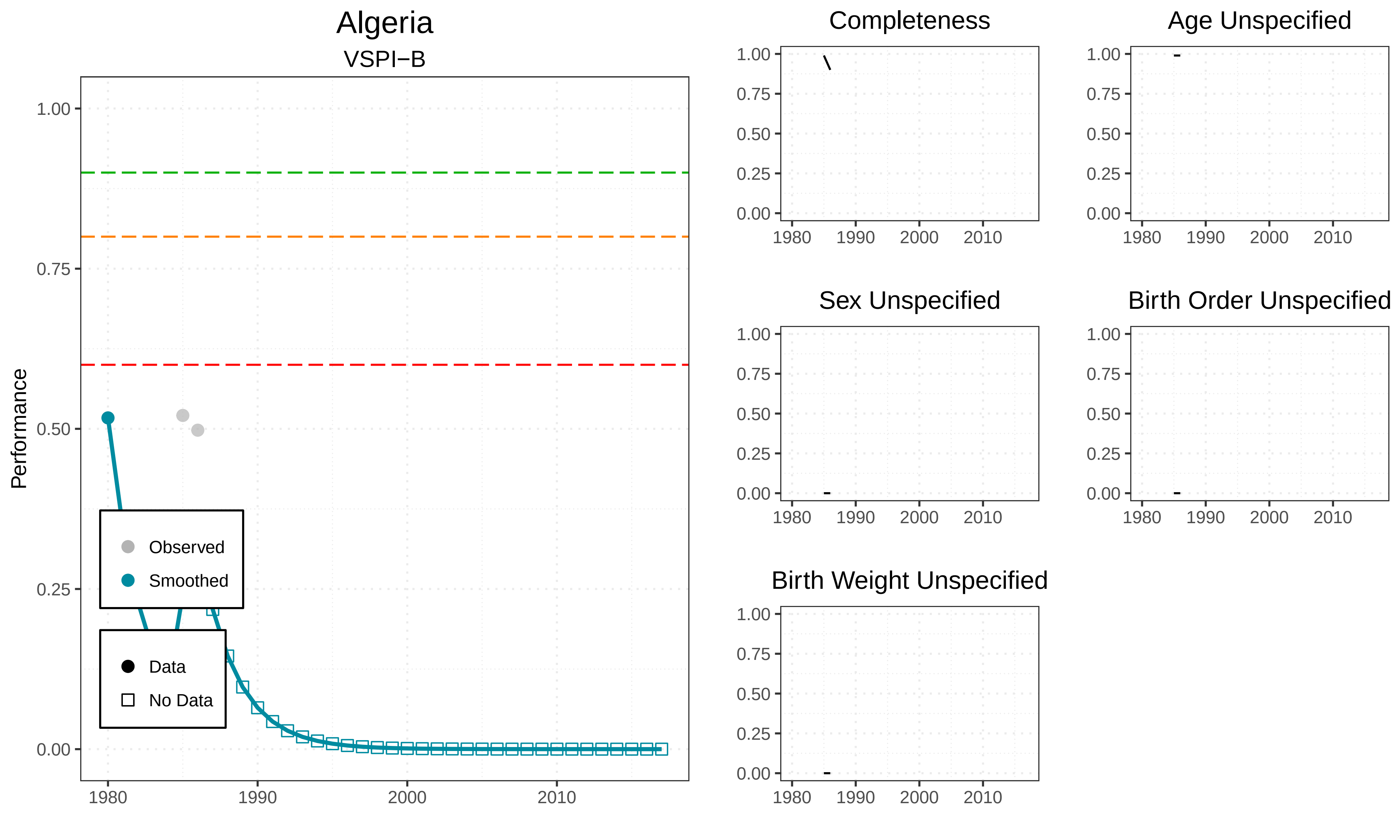
Algeria
VSPI−B
Performance
Observed
Smoothed
Data
No Data
1.00
0.75
0.50
0.25
0.00
1980
1990
2000
2010
Completeness
Age Unspecified
Sex Unspecified
Birth Order Unspecified
Birth Weight Unspecified

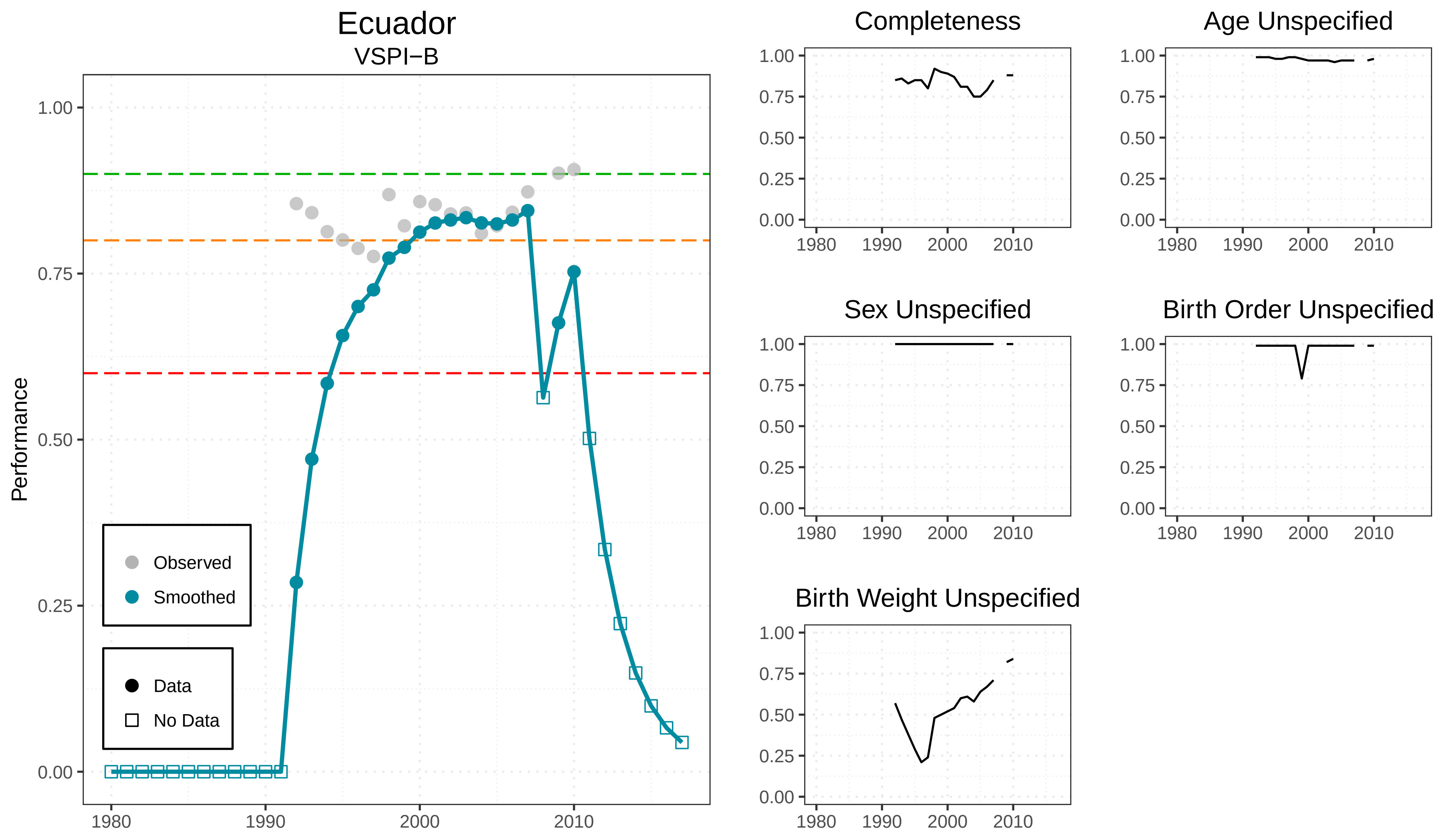

Ecuador
VSPI−B
Performance
1.00
0.75
0.50
0.25
0.00
1980
1990
2000
2010
Observed
Smoothed
Data
No Data
Completeness
Age Unspecified
Sex Unspecified
Birth Order Unspecified
Birth Weight Unspecified

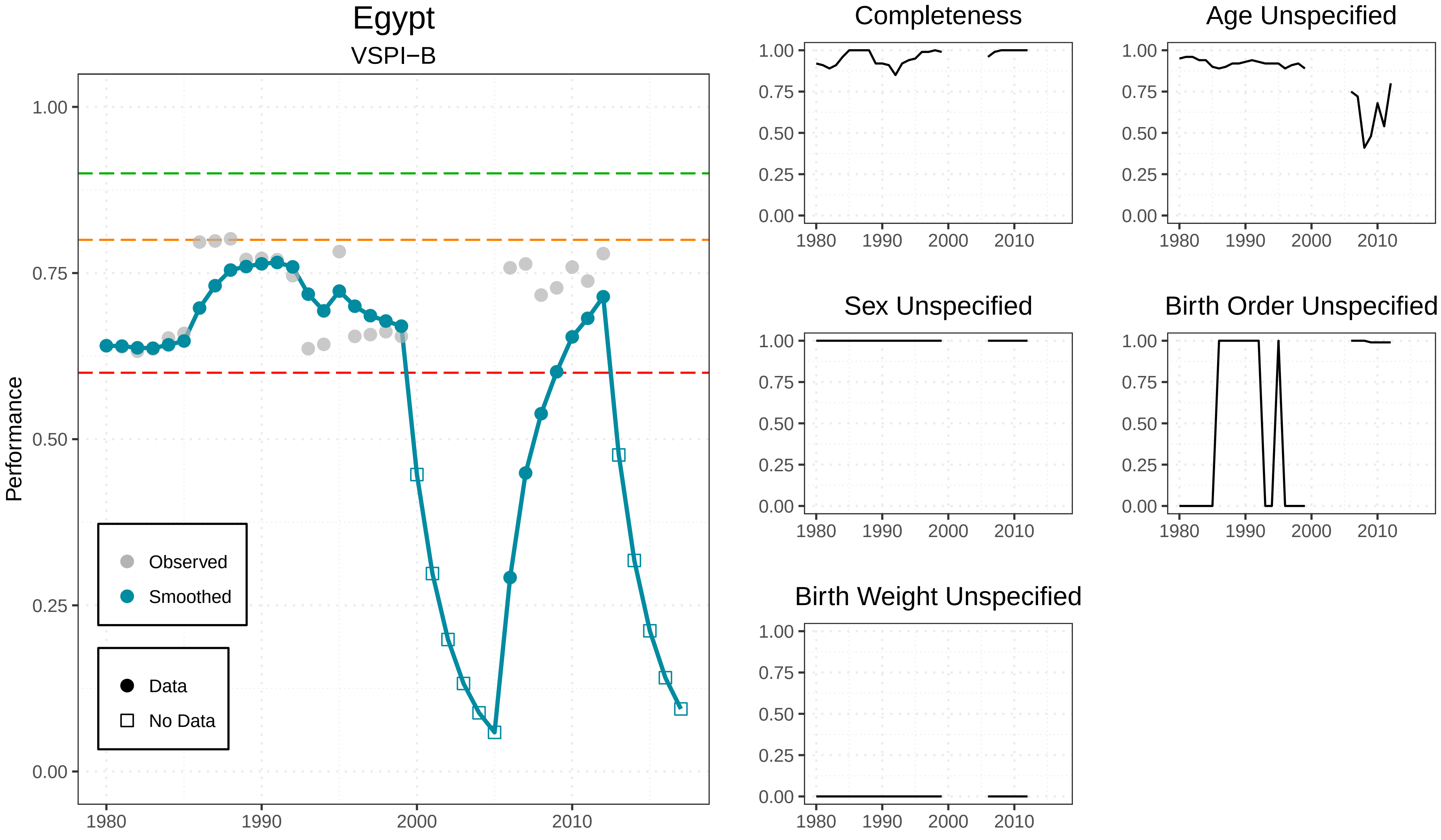

Egypt
VSPI−B
Performance
1.00
0.75
0.50
0.25
0.00
1980
1990
2000
2010
Observed
Smoothed
Data
No Data
Completeness
Age Unspecified
Sex Unspecified
Birth Order Unspecified
Birth Weight Unspecified

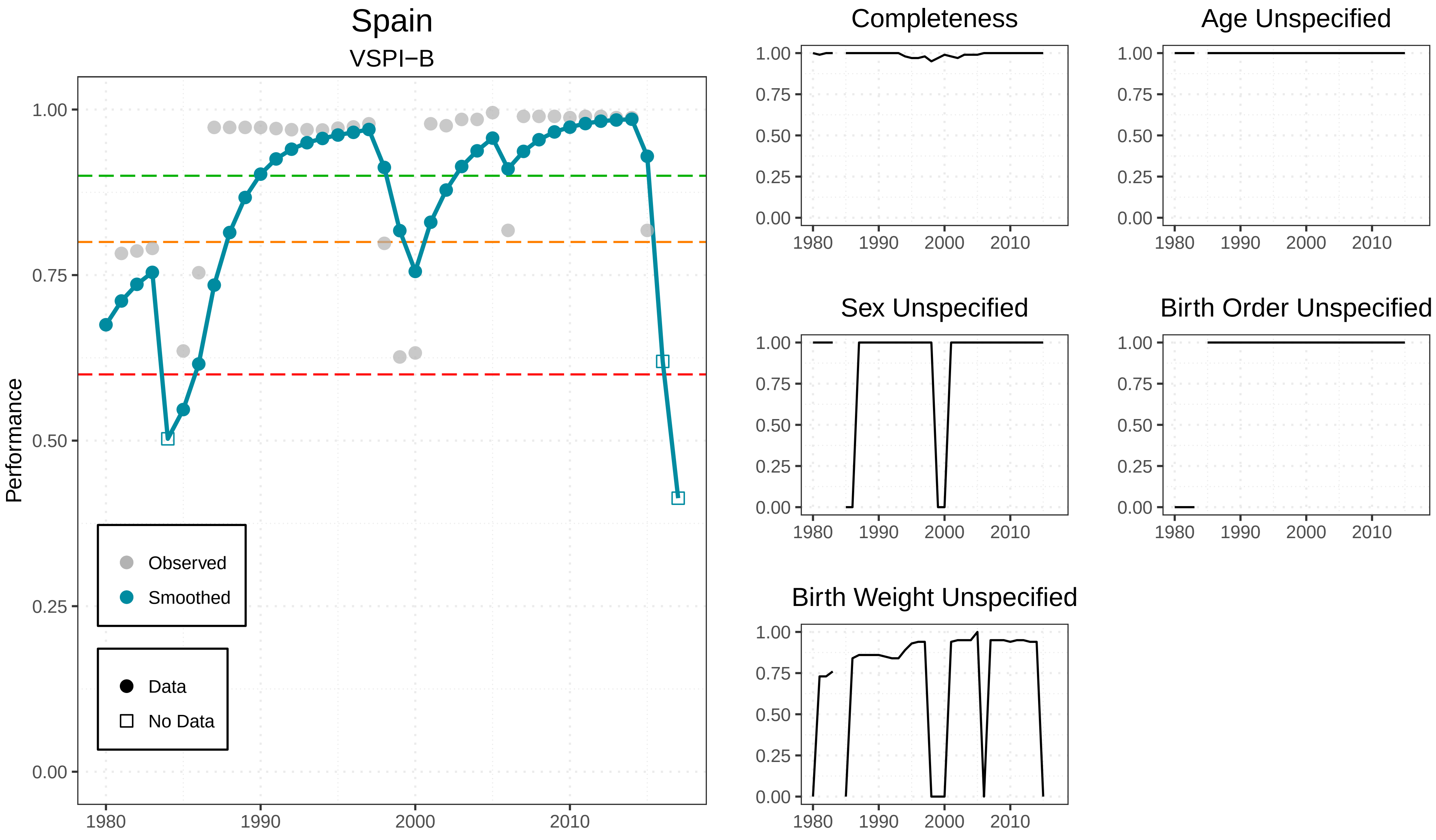

Spain
VSPI−B
Performance
Observed
Smoothed
Data
No Data
Completeness
Age Unspecified
Sex Unspecified
Birth Order Unspecified
Birth Weight Unspecified

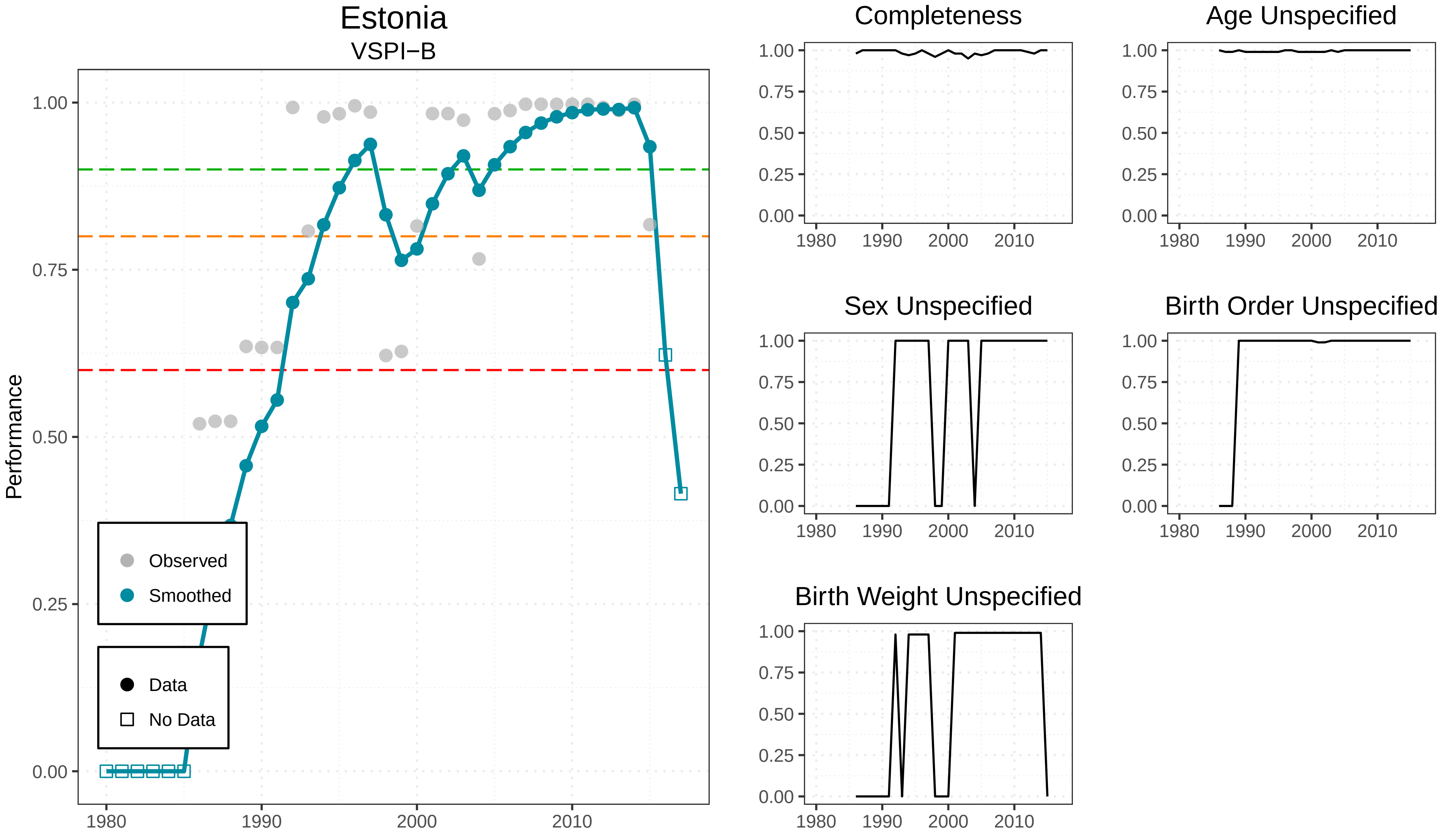

Estonia
VSPI−B
Performance
1.00
0.75
0.50
0.25
0.00
1980
1990
2000
2010
Observed
Smoothed
Data
No Data
Completeness
Age Unspecified
Sex Unspecified
Birth Order Unspecified
Birth Weight Unspecified

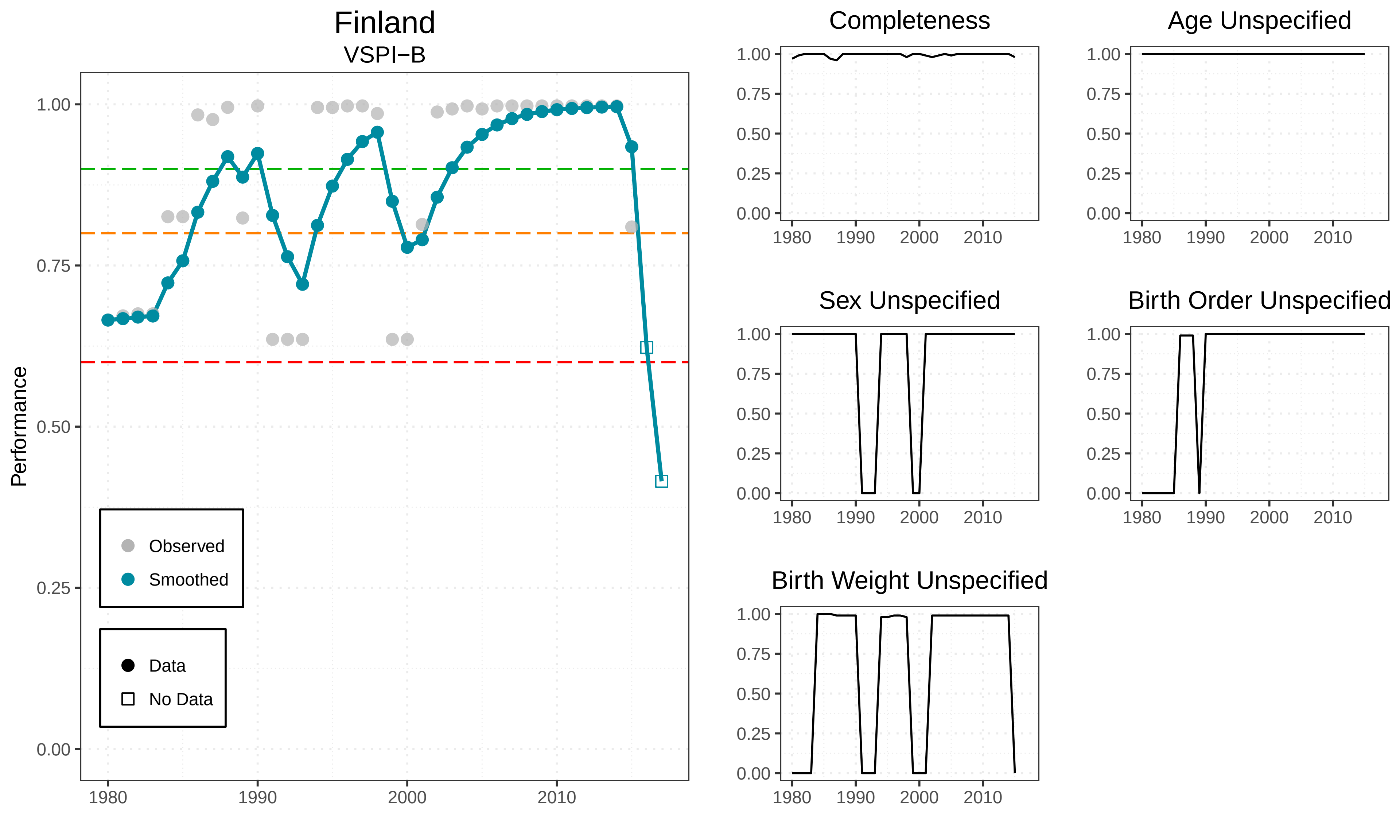

Finland
VSPI−B
Performance
1.00
0.75
0.50
0.25
0.00
1980
1990
2000
2010
Observed
Smoothed
Data
No Data
Completeness
Age Unspecified
Sex Unspecified
Birth Order Unspecified
Birth Weight Unspecified

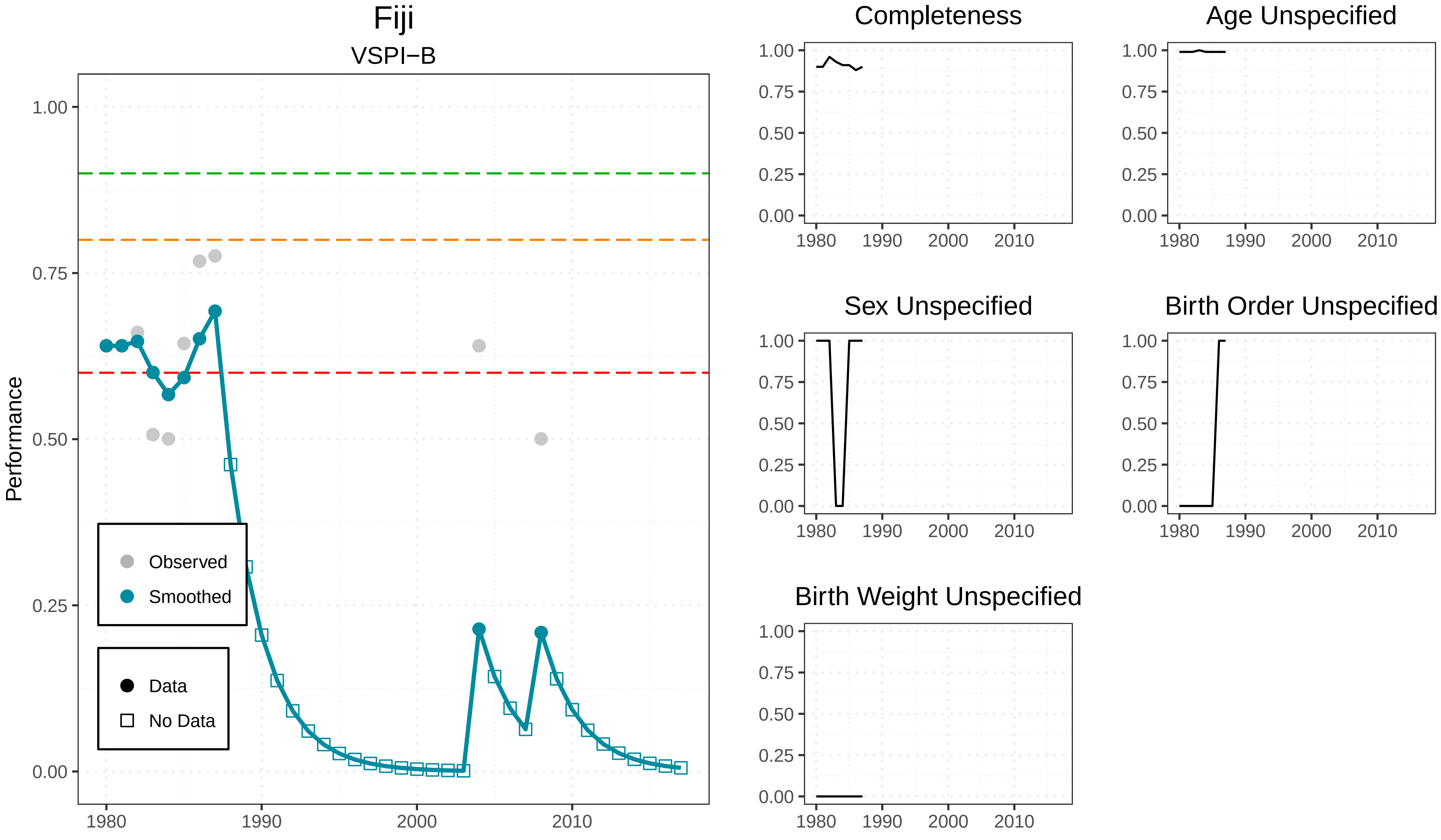
Fiji
VSPI−B
Performance
1.00
0.75
0.50
0.25
0.00
1980
1990
2000
2010
Observed
Smoothed
Data
No Data
Completeness
Age Unspecified
Sex Unspecified
Birth Order Unspecified
Birth Weight Unspecified

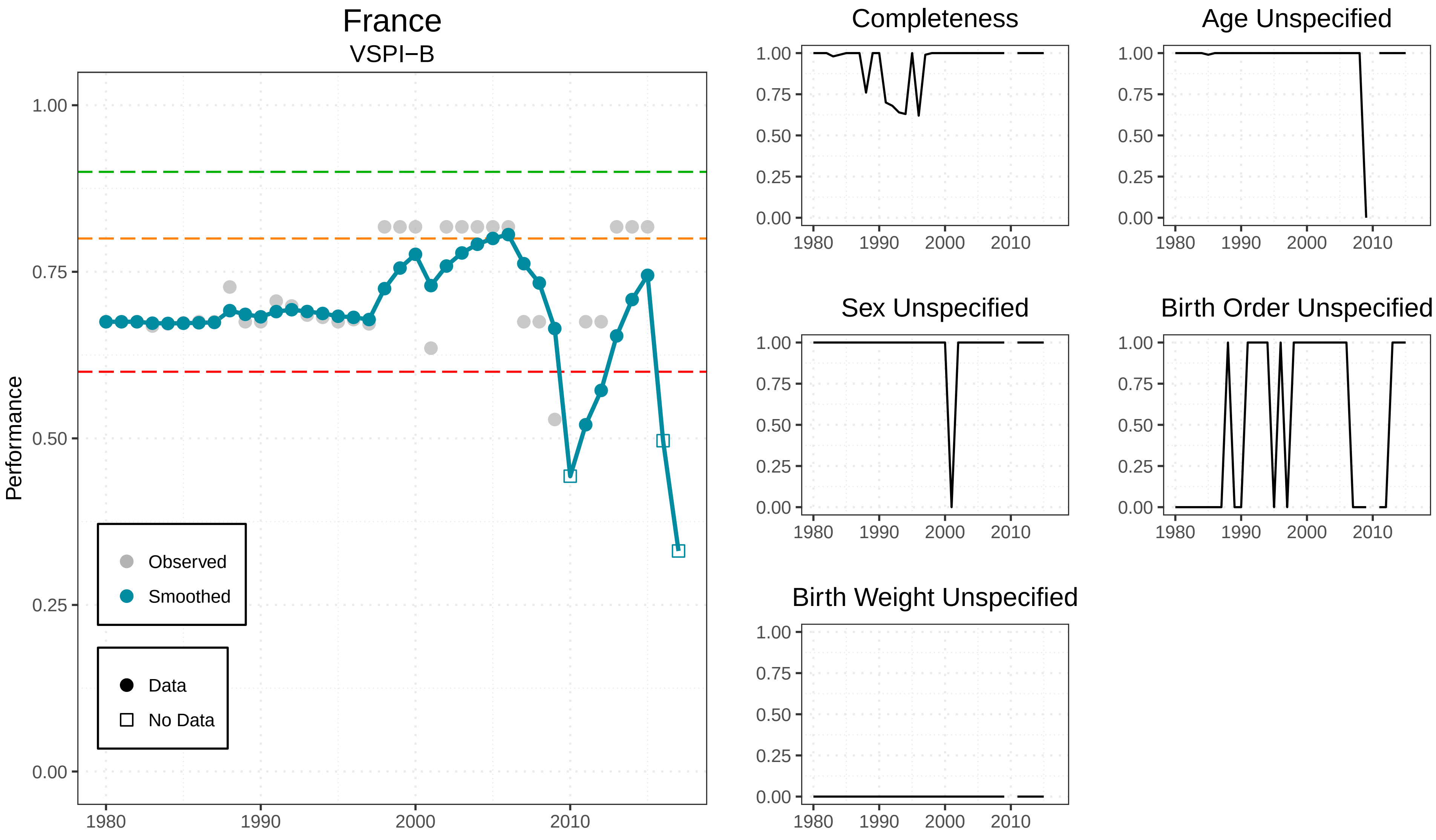

France
VSPI−B
Performance
1.00
0.75
0.50
0.25
0.00
1980
1990
2000
2010
Observed
Smoothed
Data
No Data
Completeness
Age Unspecified
Sex Unspecified
Birth Order Unspecified
Birth Weight Unspecified

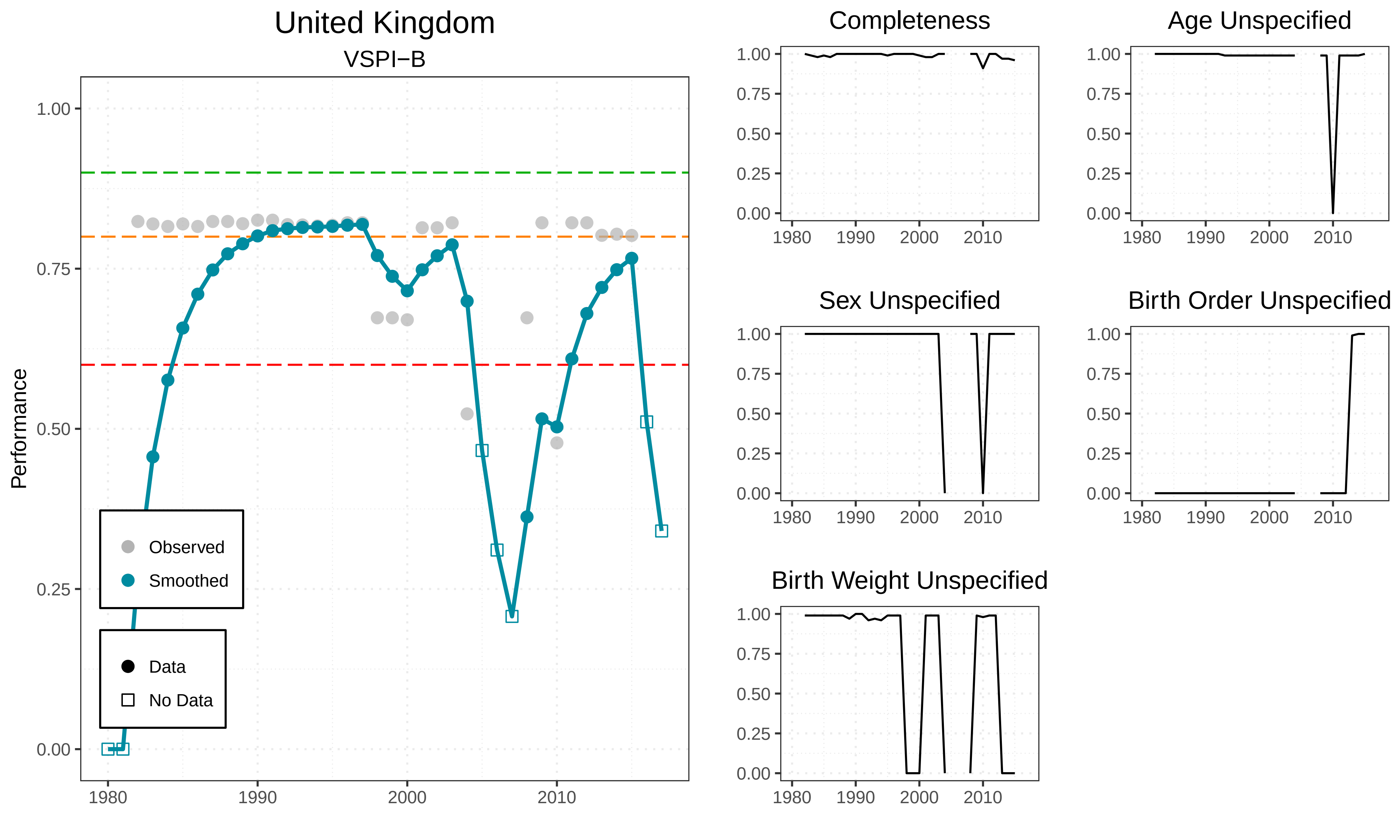
United Kingdom
VSPI−B
Performance
1.00
0.75
0.50
0.25
0.00
1980
1990
2000
2010
Observed
Smoothed
Data
No Data
Completeness
Age Unspecified
Sex Unspecified
Birth Order Unspecified
Birth Weight Unspecified

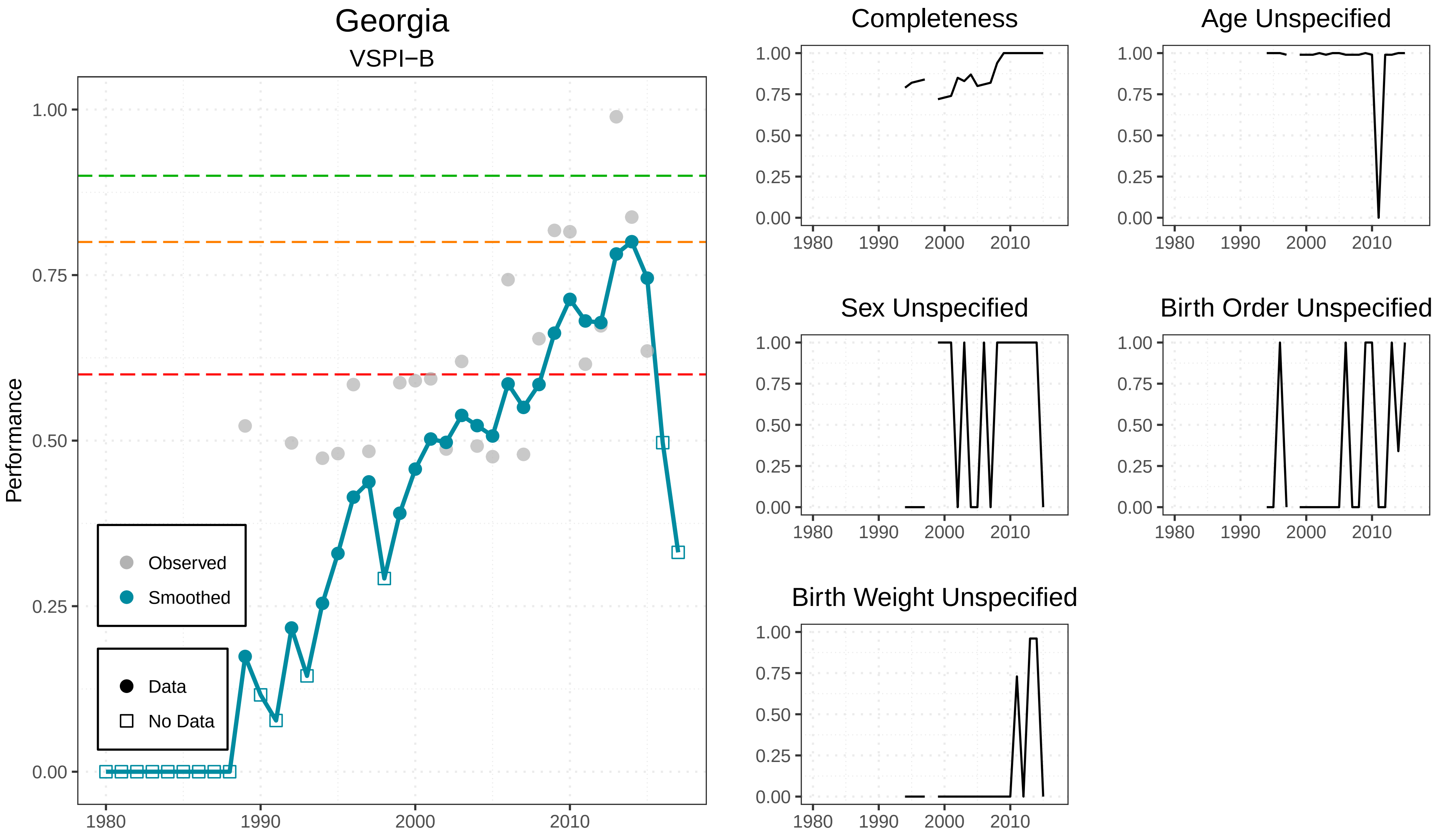
Georgia
VSPI−B
Performance
1.00
0.75
0.50
0.25
0.00
1980
1990
2000
2010
Observed
Smoothed
Data
No Data
Completeness
Age Unspecified
Sex Unspecified
Birth Order Unspecified
Birth Weight Unspecified

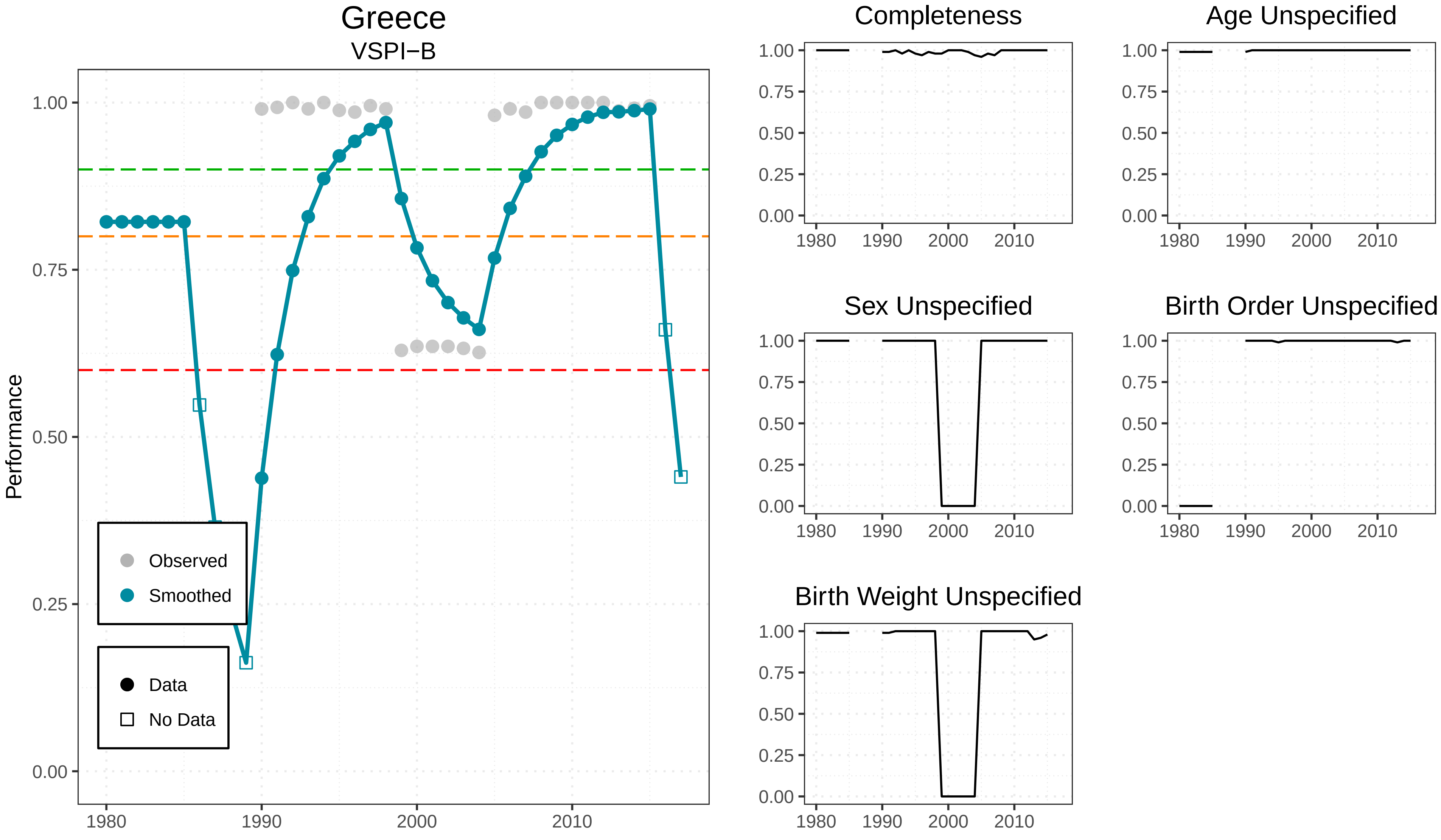
Greece
VSPI−B
Performance
1.00
0.75
0.50
0.25
0.00
1980
1990
2000
2010
Observed
Smoothed
Data
No Data
Completeness
Age Unspecified
Sex Unspecified
Birth Order Unspecified
Birth Weight Unspecified

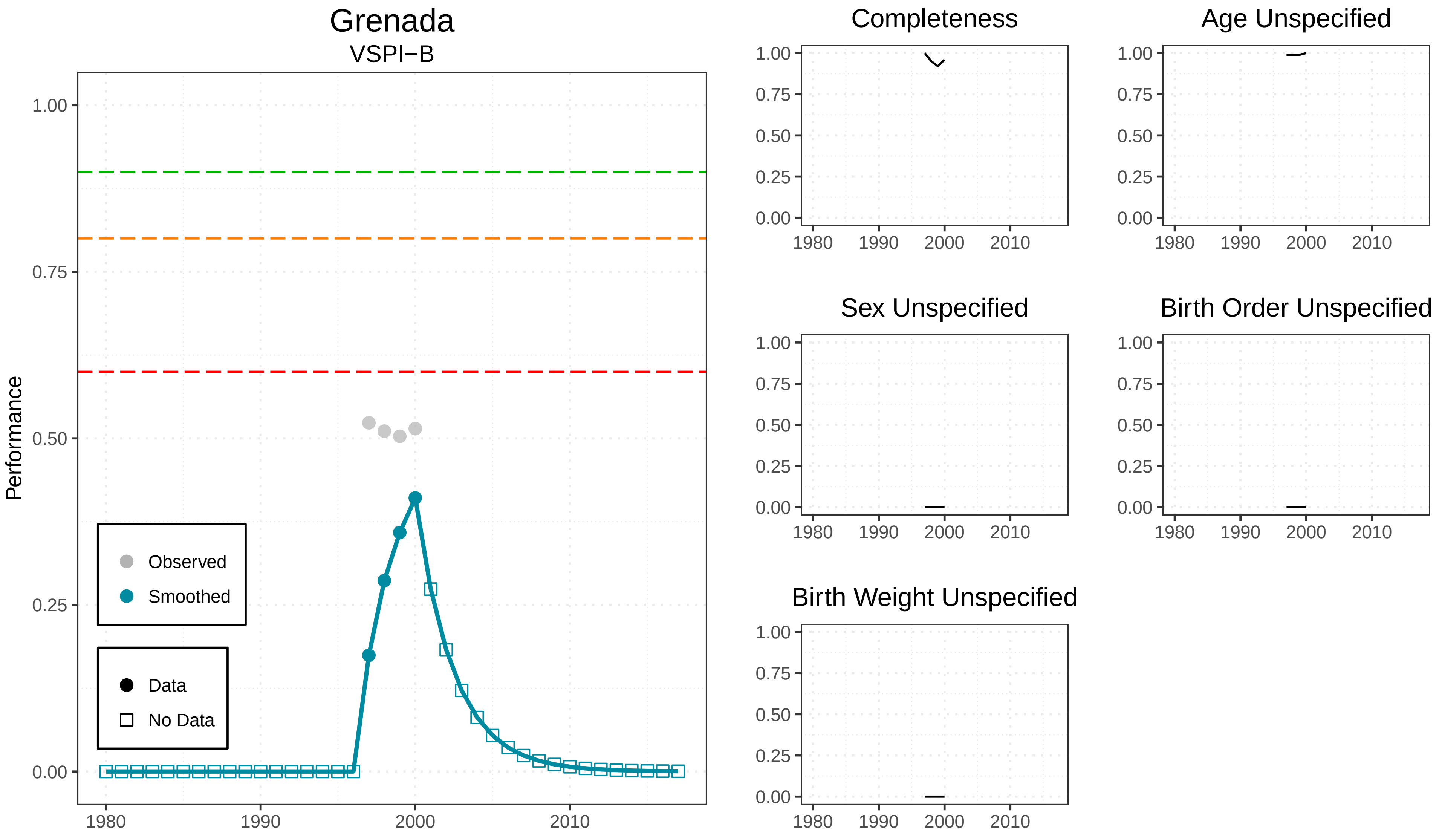
Grenada
VSPI−B
Performance
Observed
Smoothed
Data
No Data
Completeness
Age Unspecified
Sex Unspecified
Birth Order Unspecified
Birth Weight Unspecified

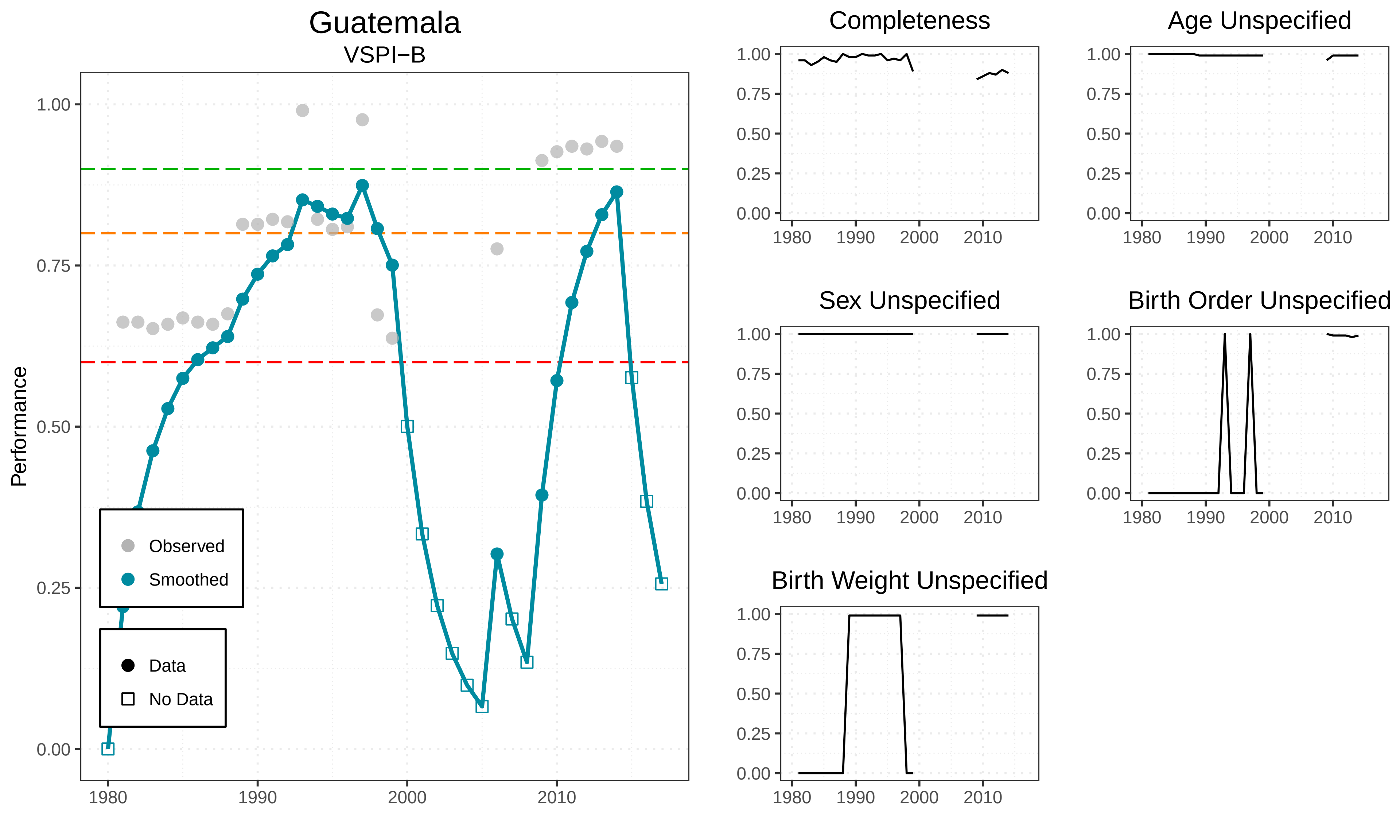
Guatemala
VSPI−B
Performance
1.00
0.75
0.50
0.25
0.00
1980
1990
2000
2010
Observed
Smoothed
Data
No Data
Completeness
Age Unspecified
Sex Unspecified
Birth Order Unspecified
Birth Weight Unspecified

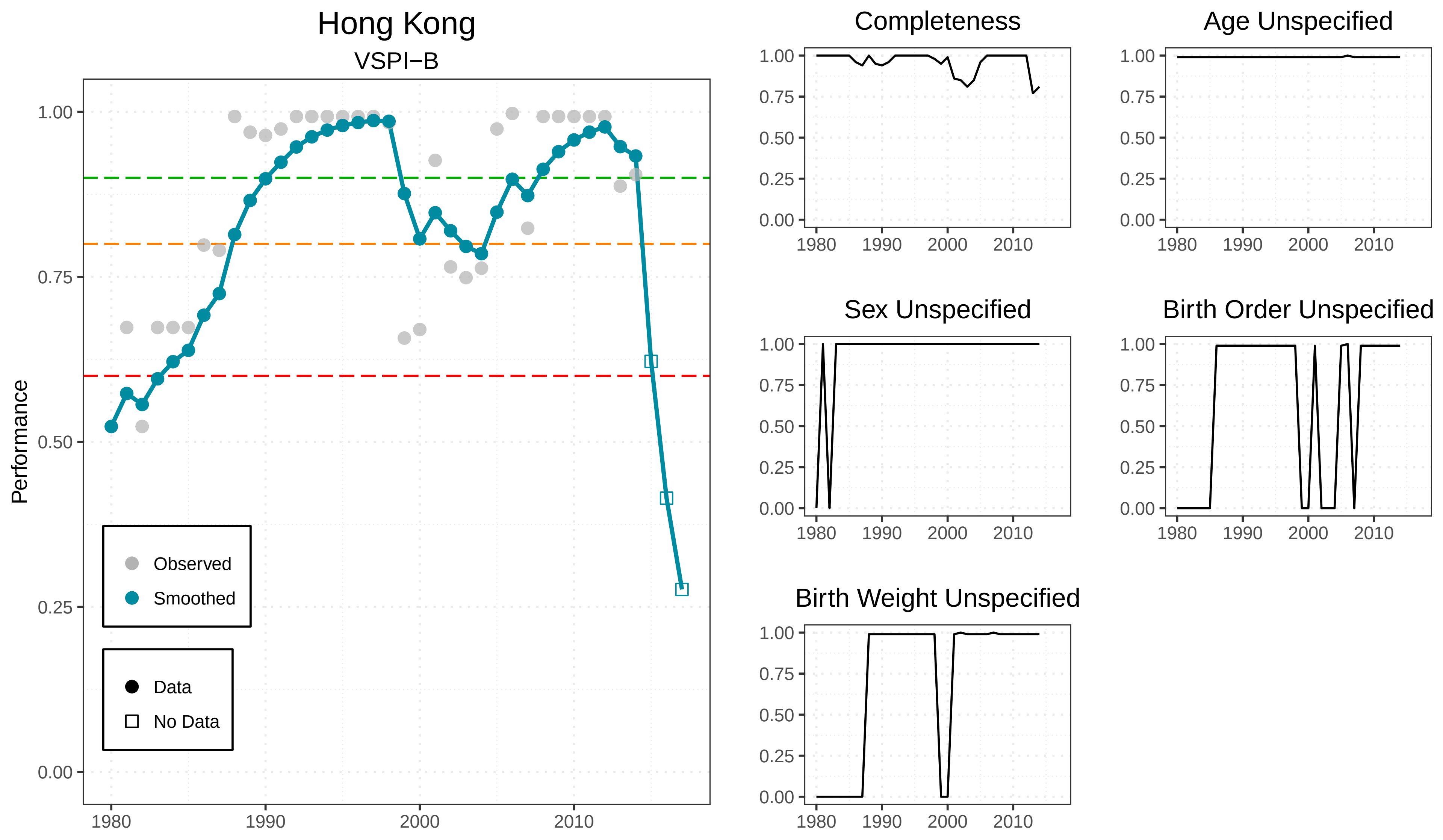

Hong Kong
VSPI−B
Performance
1.00
0.75
0.50
0.25
0.00
1980
1990
2000
2010
Observed
Smoothed
Data
No Data
Completeness
Age Unspecified
Sex Unspecified
Birth Order Unspecified
Birth Weight Unspecified

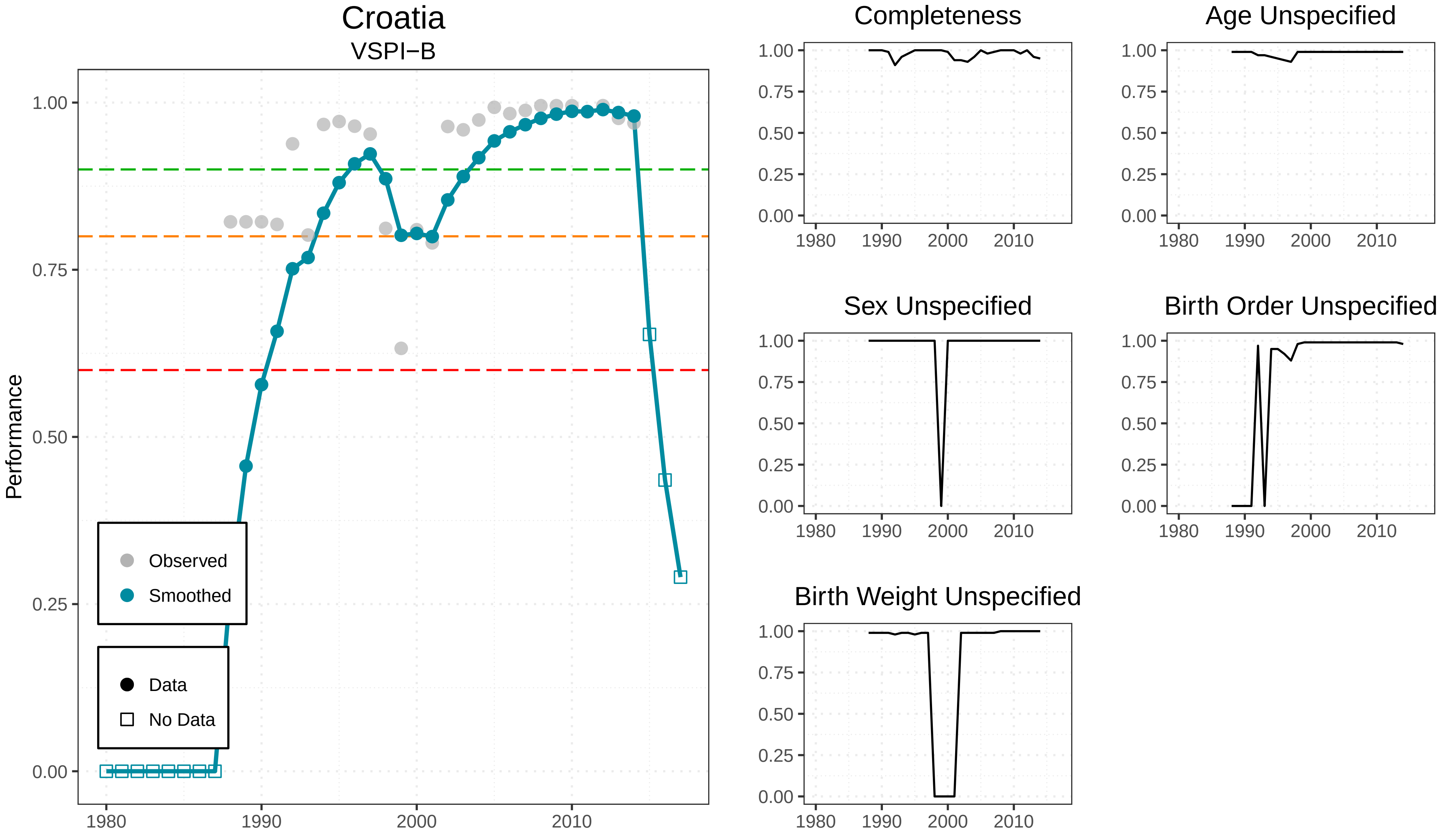
Croatia
VSPI−B
Performance
1.00
0.75
0.50
0.25
0.00
1980
1990
2000
2010
Observed
Smoothed
Data
No Data
Completeness
Age Unspecified
Sex Unspecified
Birth Order Unspecified
Birth Weight Unspecified

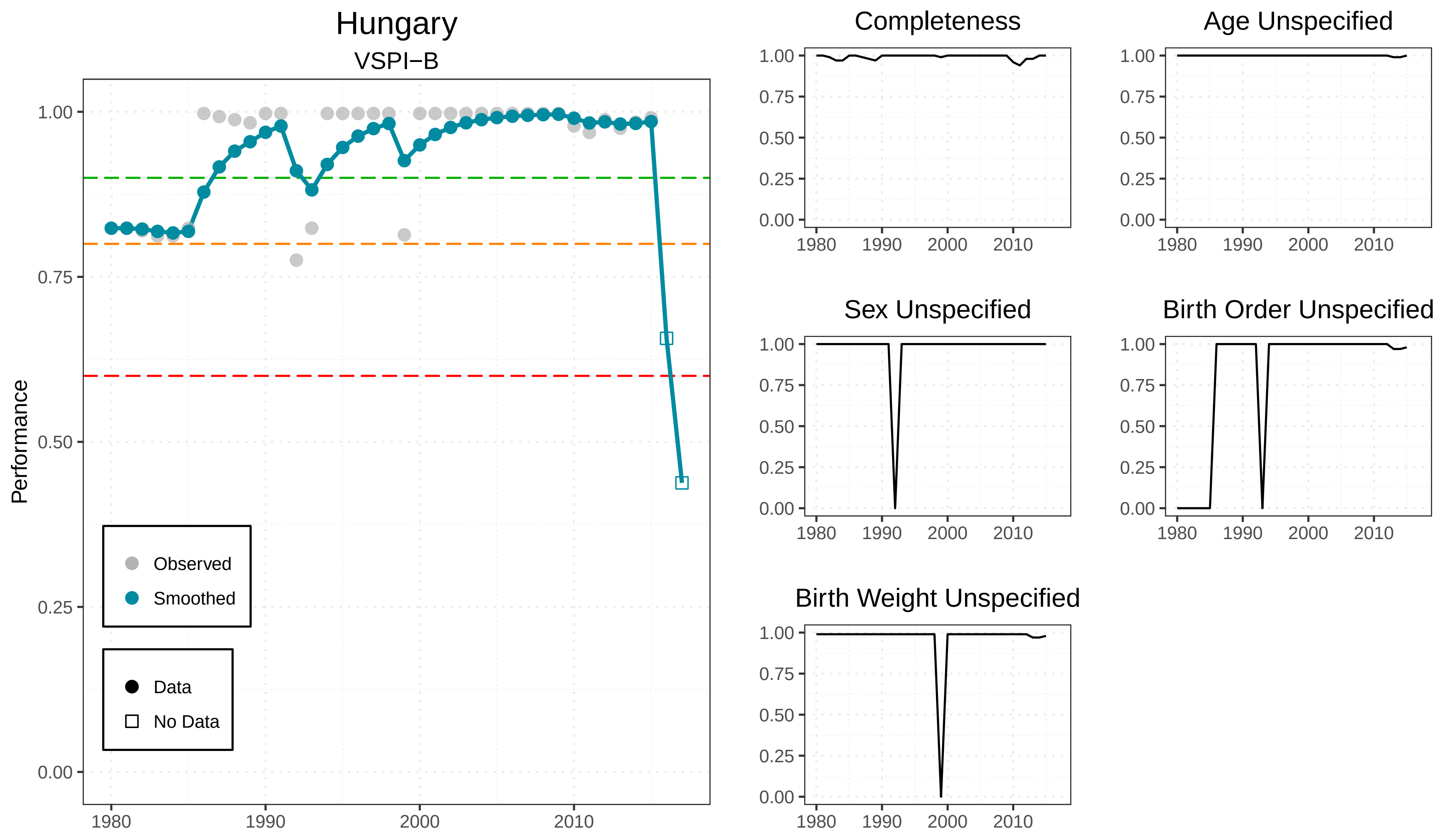

Hungary
VSPI−B
Performance
1.00
0.75
0.50
0.25
0.00
1980
1990
2000
2010
Observed
Smoothed
Data
No Data
Completeness
Age Unspecified
Sex Unspecified
Birth Order Unspecified
Birth Weight Unspecified

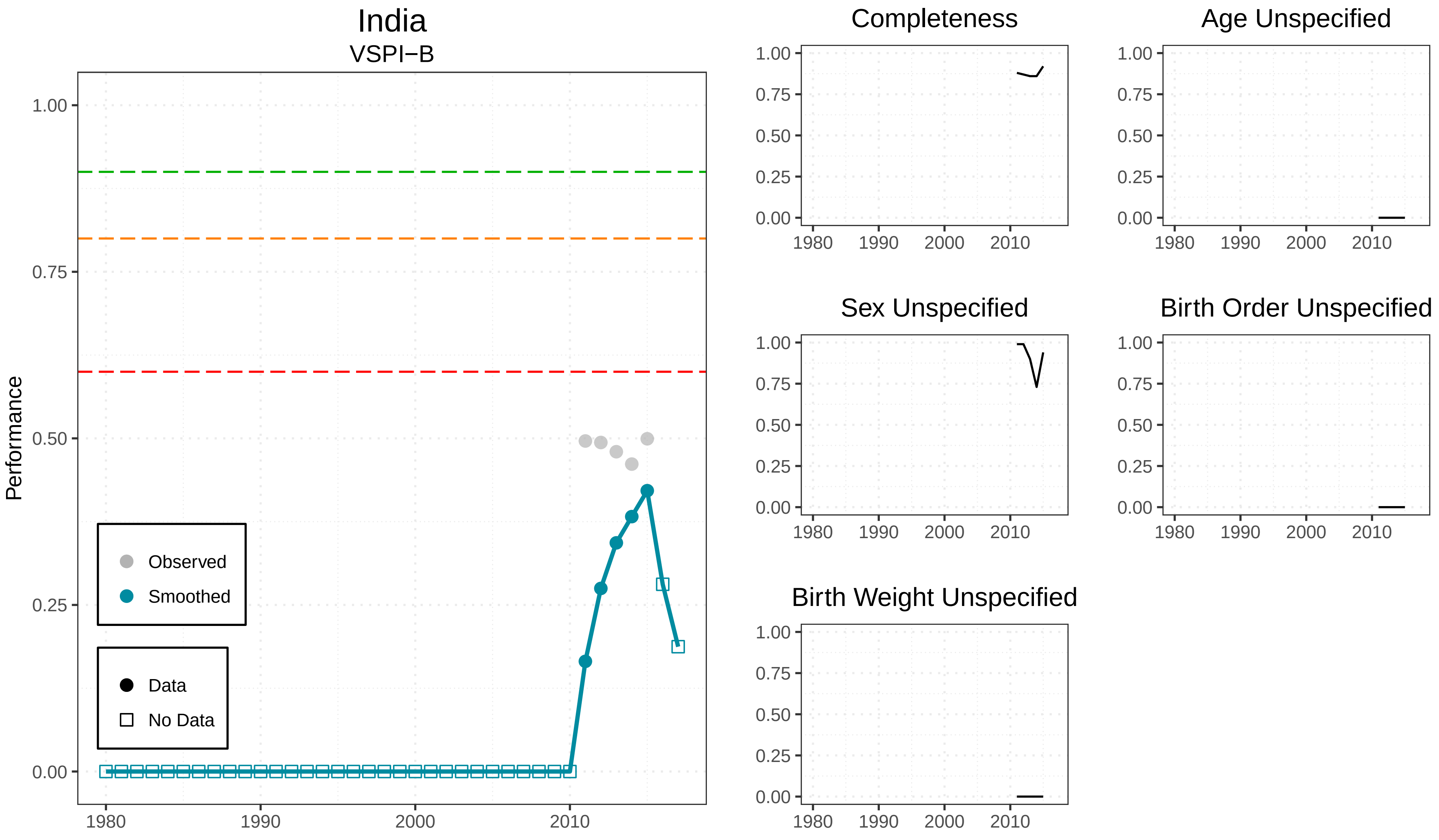
India
VSPI−B
Performance
1.00
0.75
0.50
0.25
0.00
1980
1990
2000
2010
Observed
Smoothed
Data
No Data
Completeness
Age Unspecified
Sex Unspecified
Birth Order Unspecified
Birth Weight Unspecified

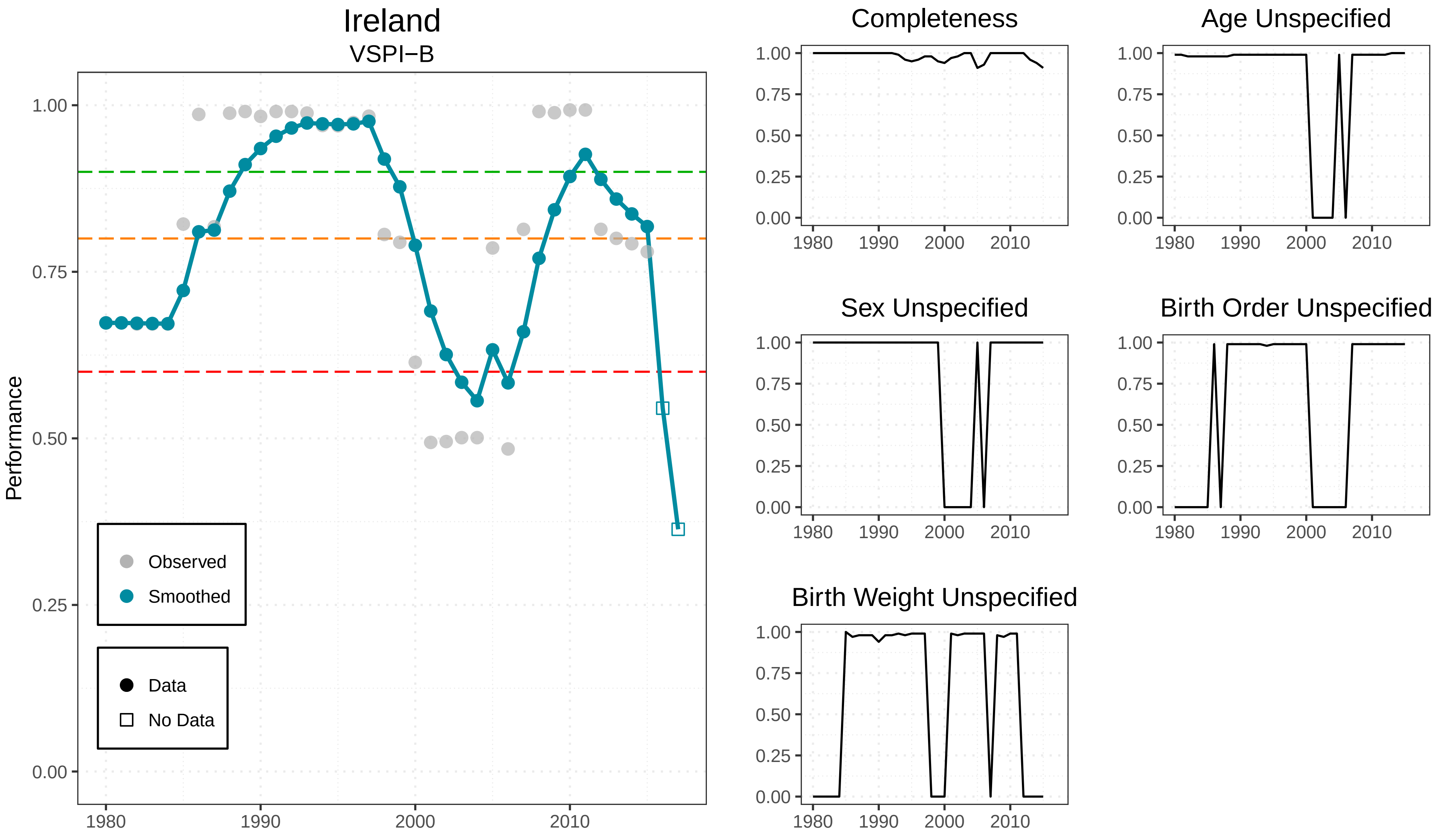

Ireland
VSPI−B
Performance
1.00
0.75
0.50
0.25
0.00
1980
1990
2000
2010
Observed
Smoothed
Data
No Data
Completeness
Age Unspecified
Sex Unspecified
Birth Order Unspecified
Birth Weight Unspecified

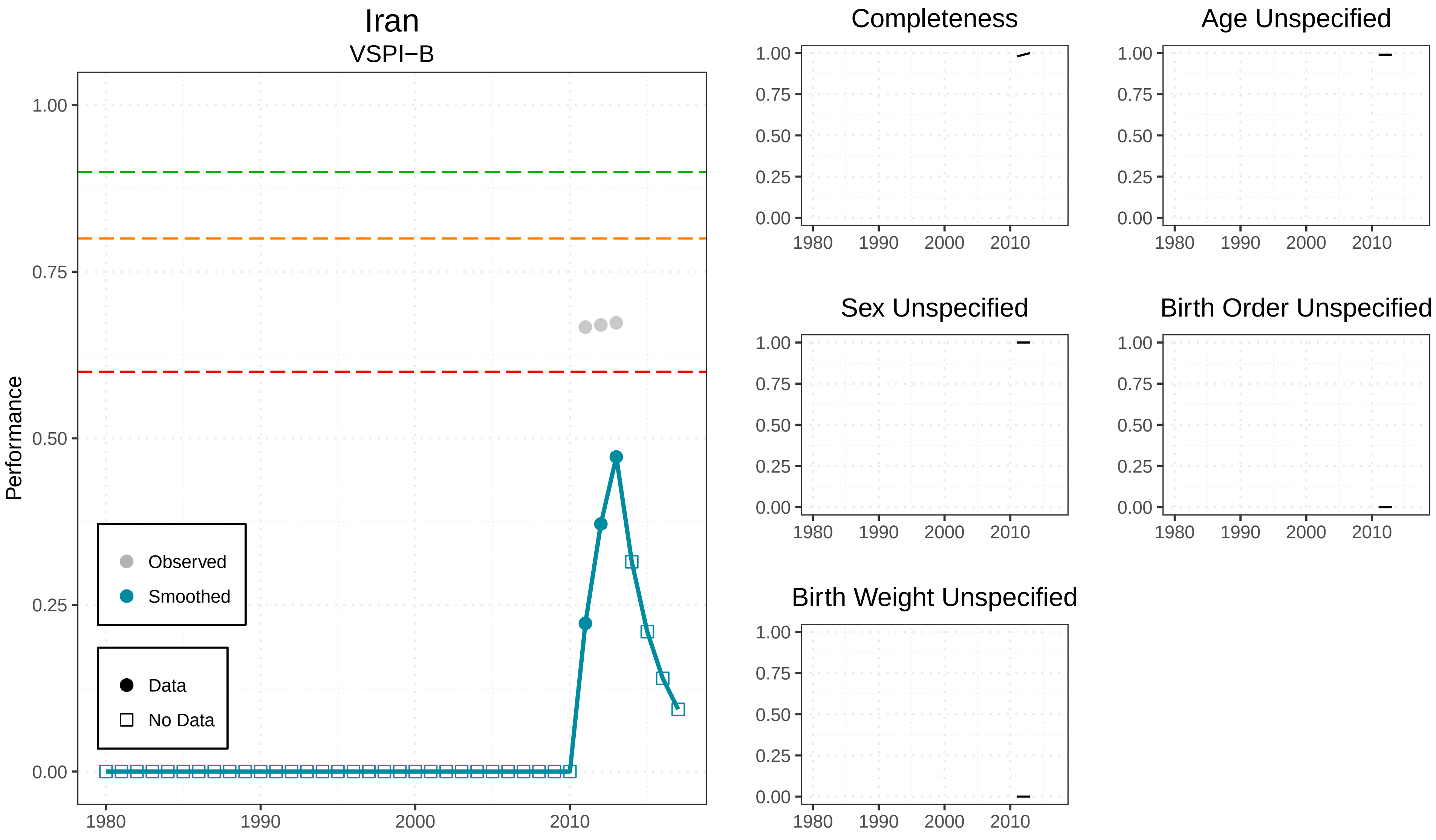

Iran
VSPI−B
Performance
1.00
0.75
0.50
0.25
0.00
1980
1990
2000
2010
Observed
Smoothed
Data
No Data
Completeness
Age Unspecified
Sex Unspecified
Birth Order Unspecified
Birth Weight Unspecified

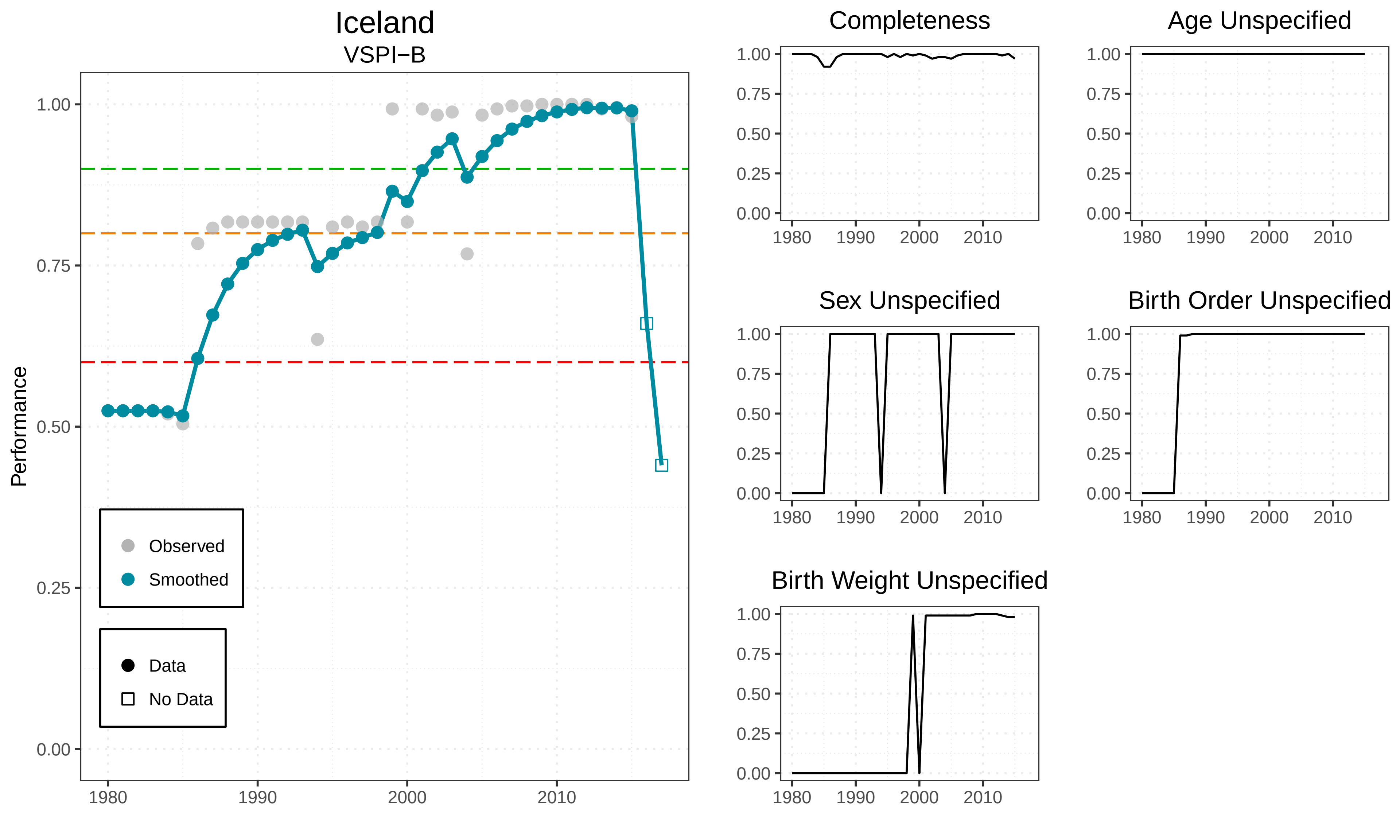

Iceland
VSPI−B
Performance
1.00
0.75
0.50
0.25
0.00
1980
1990
2000
2010
Observed
Smoothed
Data
No Data
Completeness
Age Unspecified
Sex Unspecified
Birth Order Unspecified
Birth Weight Unspecified

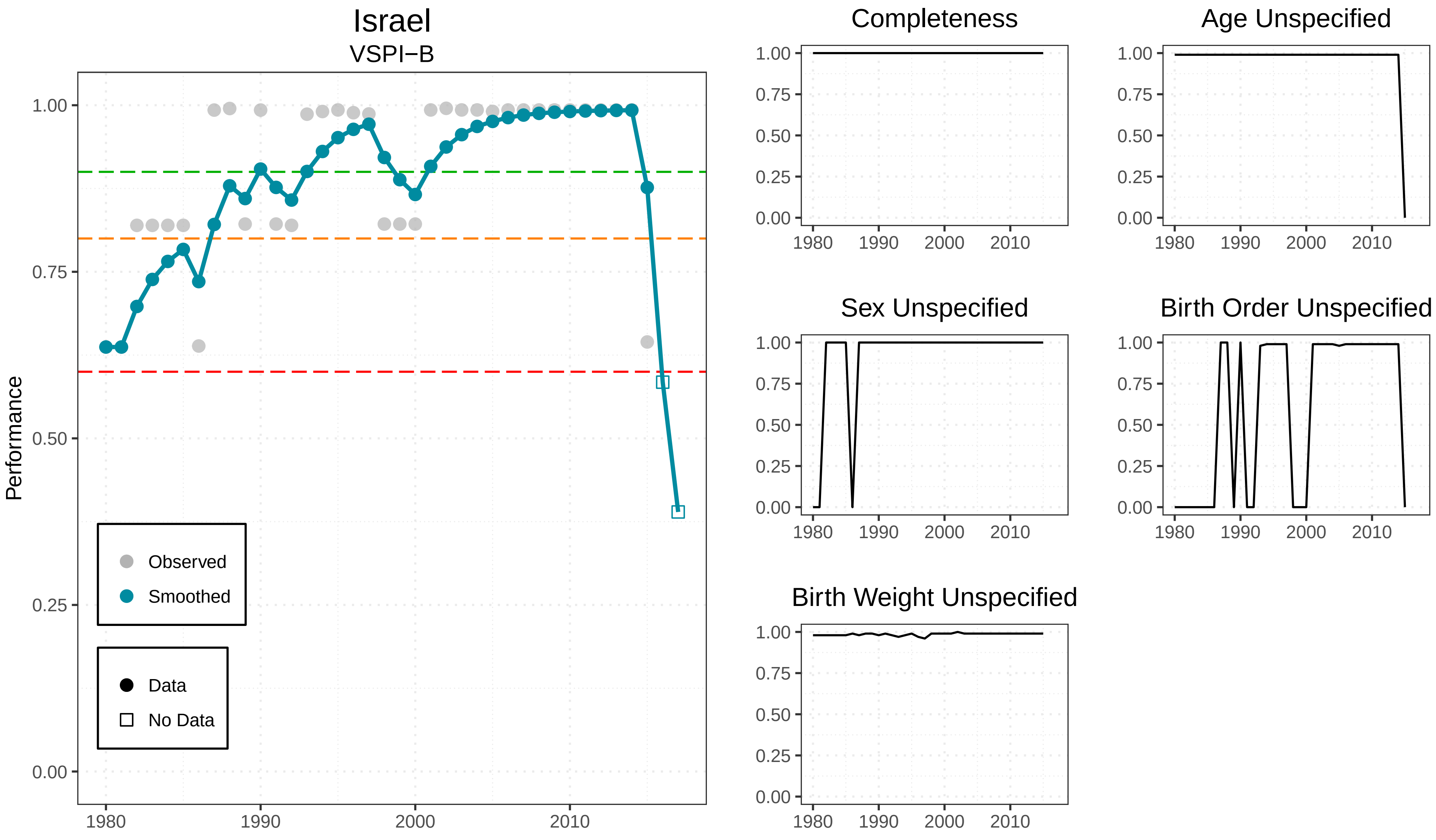

Israel
VSPI−B
Performance
1.00
0.75
0.50
0.25
0.00
1980
1990
2000
2010
Observed
Smoothed
Data
No Data
Completeness
Age Unspecified
Sex Unspecified
Birth Order Unspecified
Birth Weight Unspecified

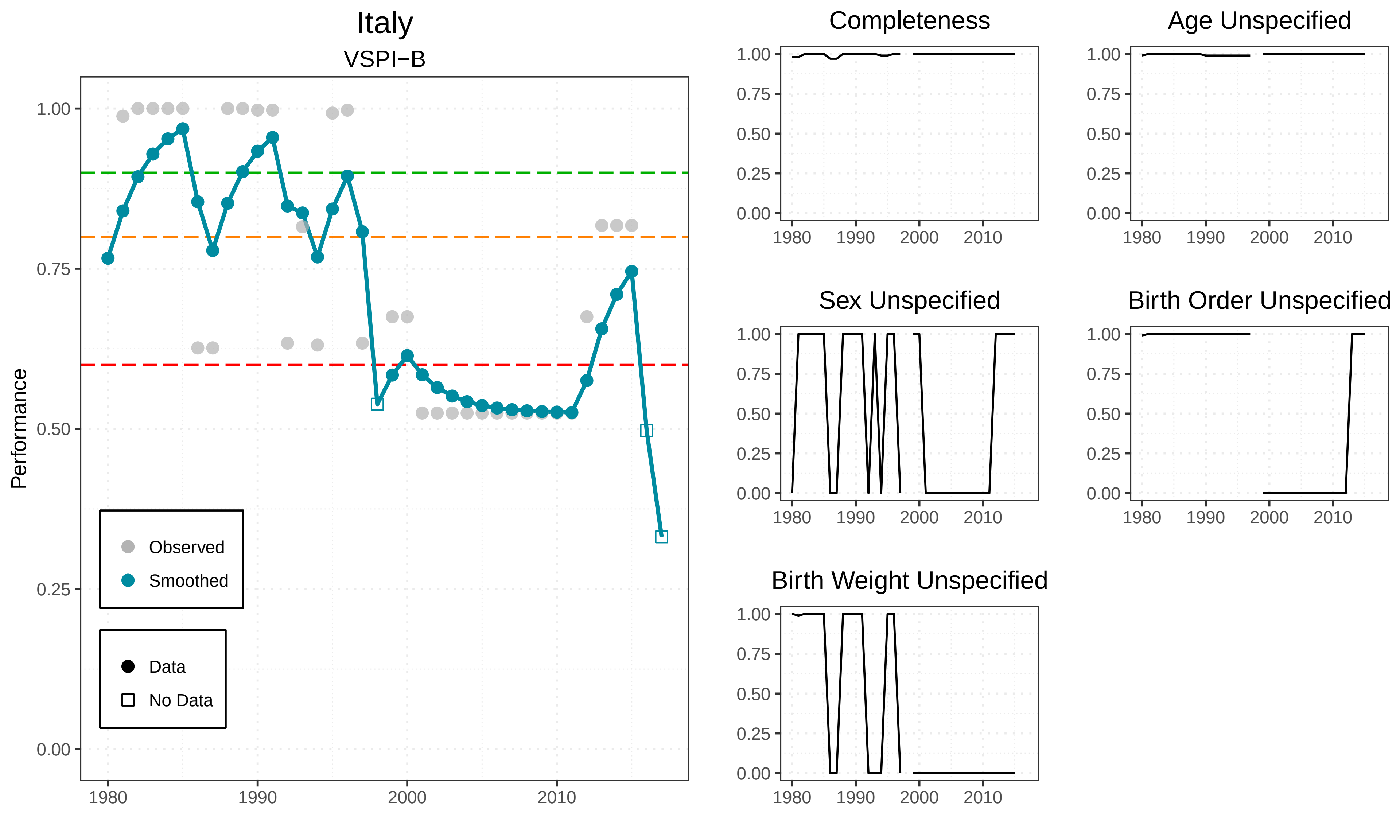

Italy
VSPI−B
Performance
Observed
Smoothed
Data
No Data
Completeness
Age Unspecified
Sex Unspecified
Birth Order Unspecified
Birth Weight Unspecified

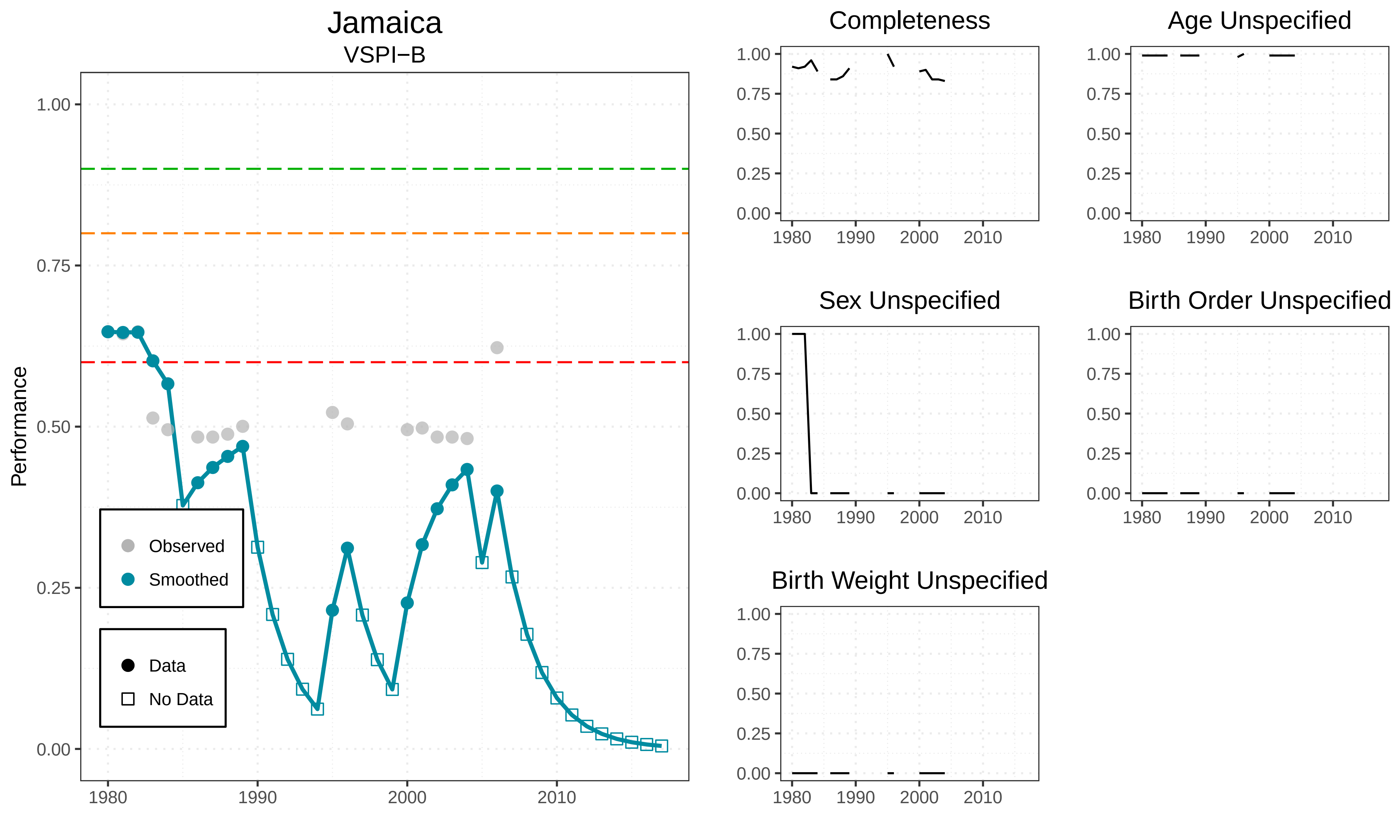

Jamaica
VSPI−B
Performance
1.00
0.75
0.50
0.25
0.00
1980
1990
2000
2010
Observed
Smoothed
Data
No Data
Completeness
Age Unspecified
Sex Unspecified
Birth Order Unspecified
Birth Weight Unspecified

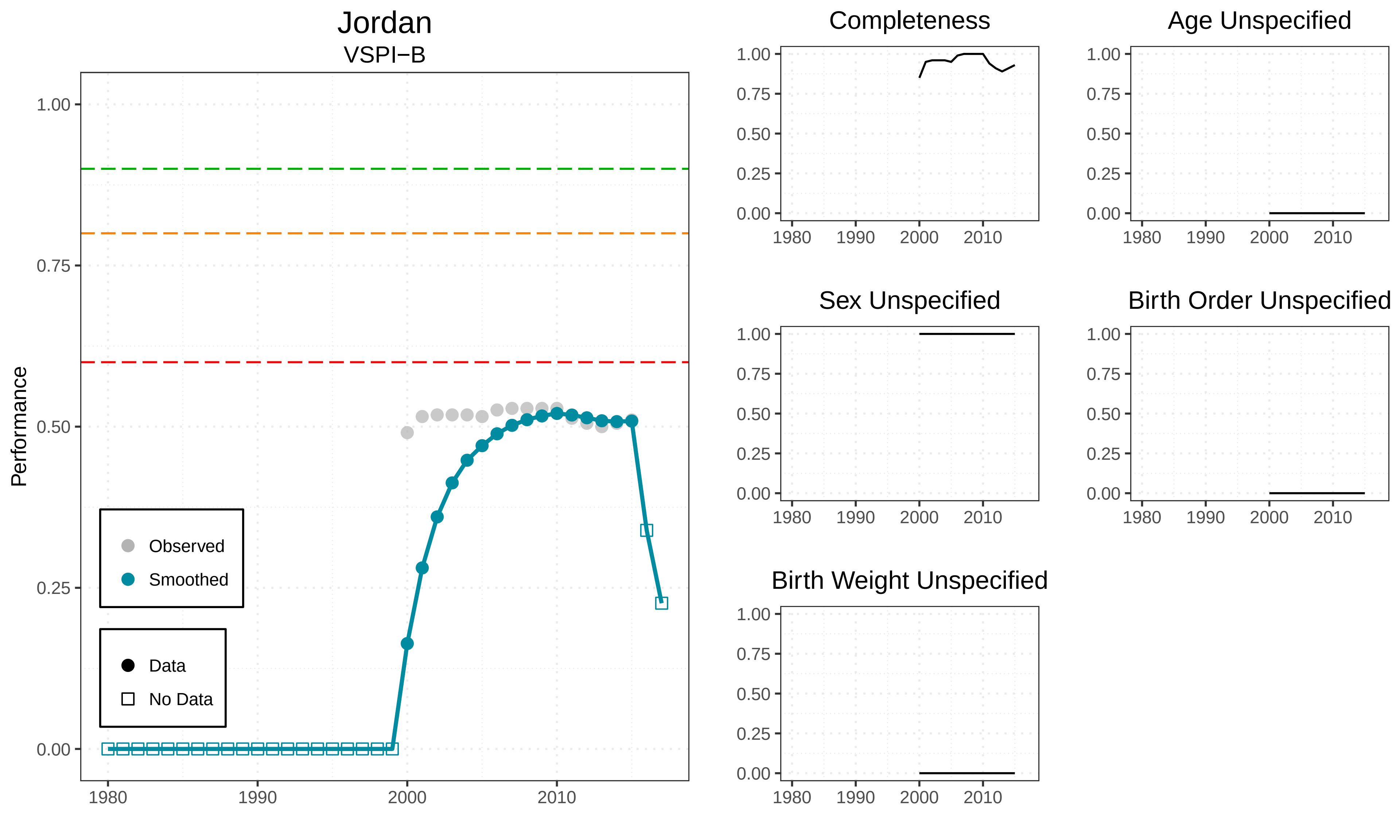
Jordan
VSPI−B
Performance
Observed
Smoothed
Data
No Data
Completeness
Age Unspecified
Sex Unspecified
Birth Order Unspecified
Birth Weight Unspecified
1.00
0.75
0.50
0.25
0.00
1980
1990
2000
2010

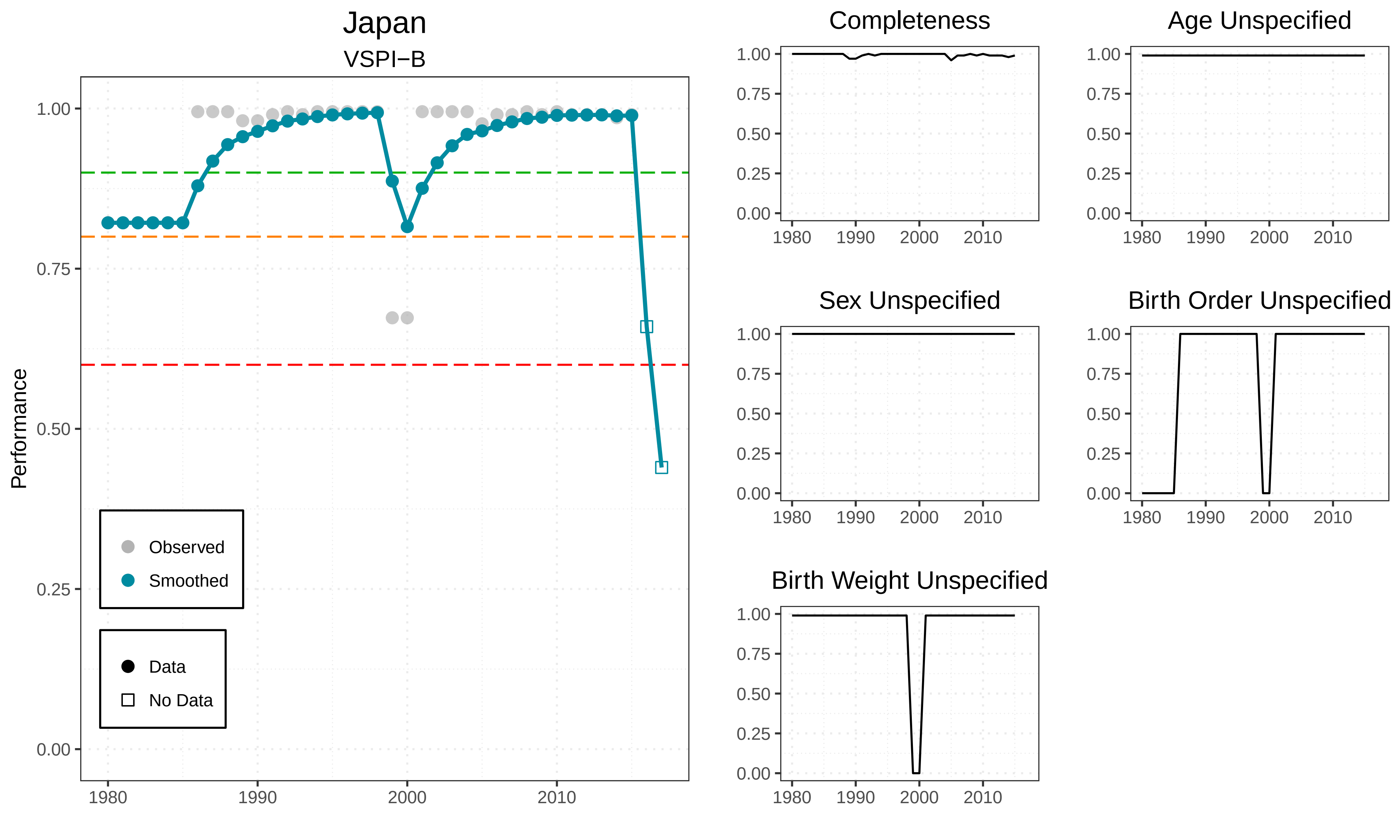
Japan
VSPI−B
Performance
1.00
0.75
0.50
0.25
0.00
1980
1990
2000
2010
Observed
Smoothed
Data
No Data
Completeness
Age Unspecified
Sex Unspecified
Birth Order Unspecified
Birth Weight Unspecified

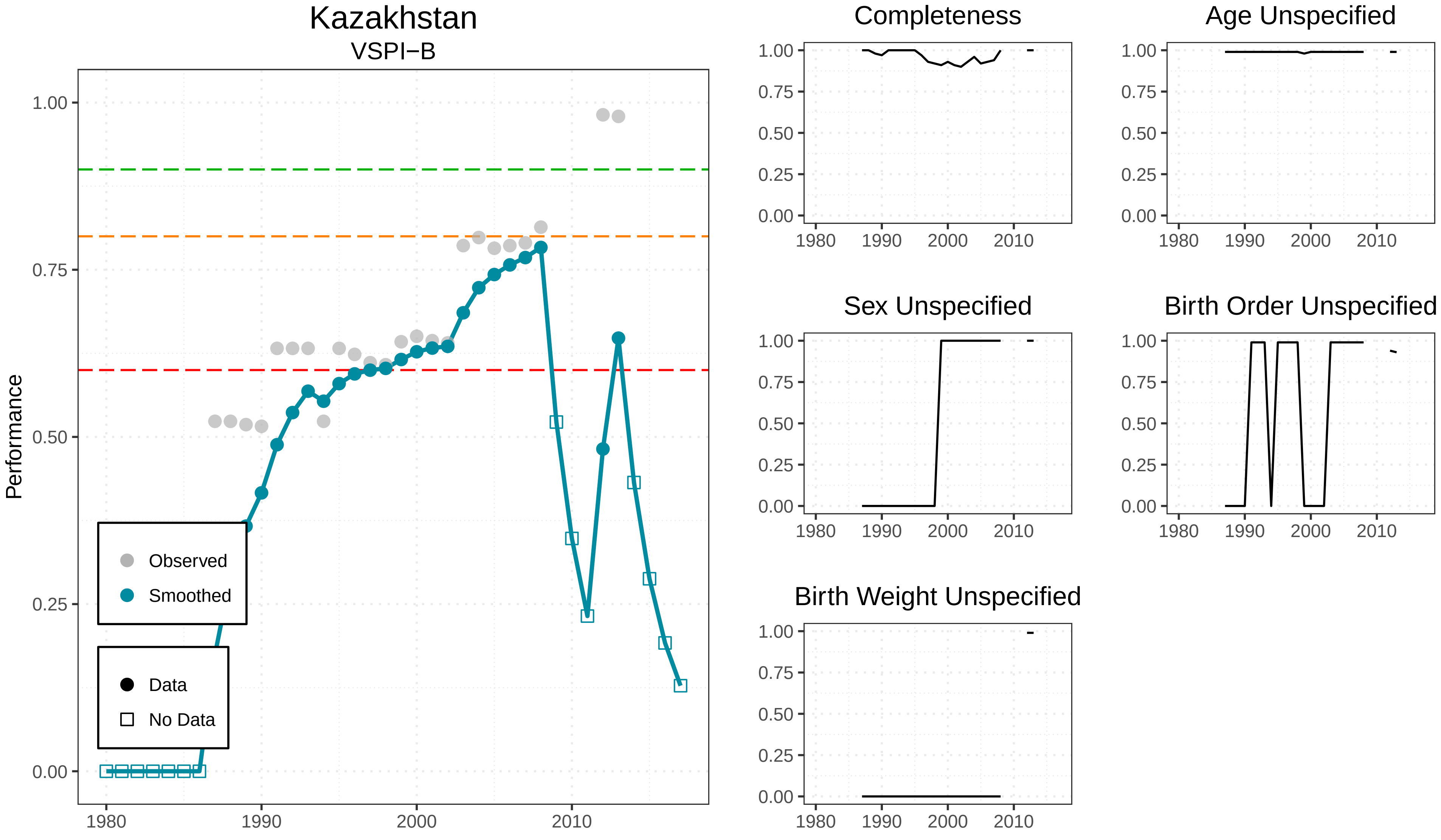
Kazakhstan
VSPI−B
Performance
1.00
0.75
0.50
0.25
0.00
1980
1990
2000
2010
Observed
Smoothed
Data
No Data
Completeness
Age Unspecified
Sex Unspecified
Birth Order Unspecified
Birth Weight Unspecified

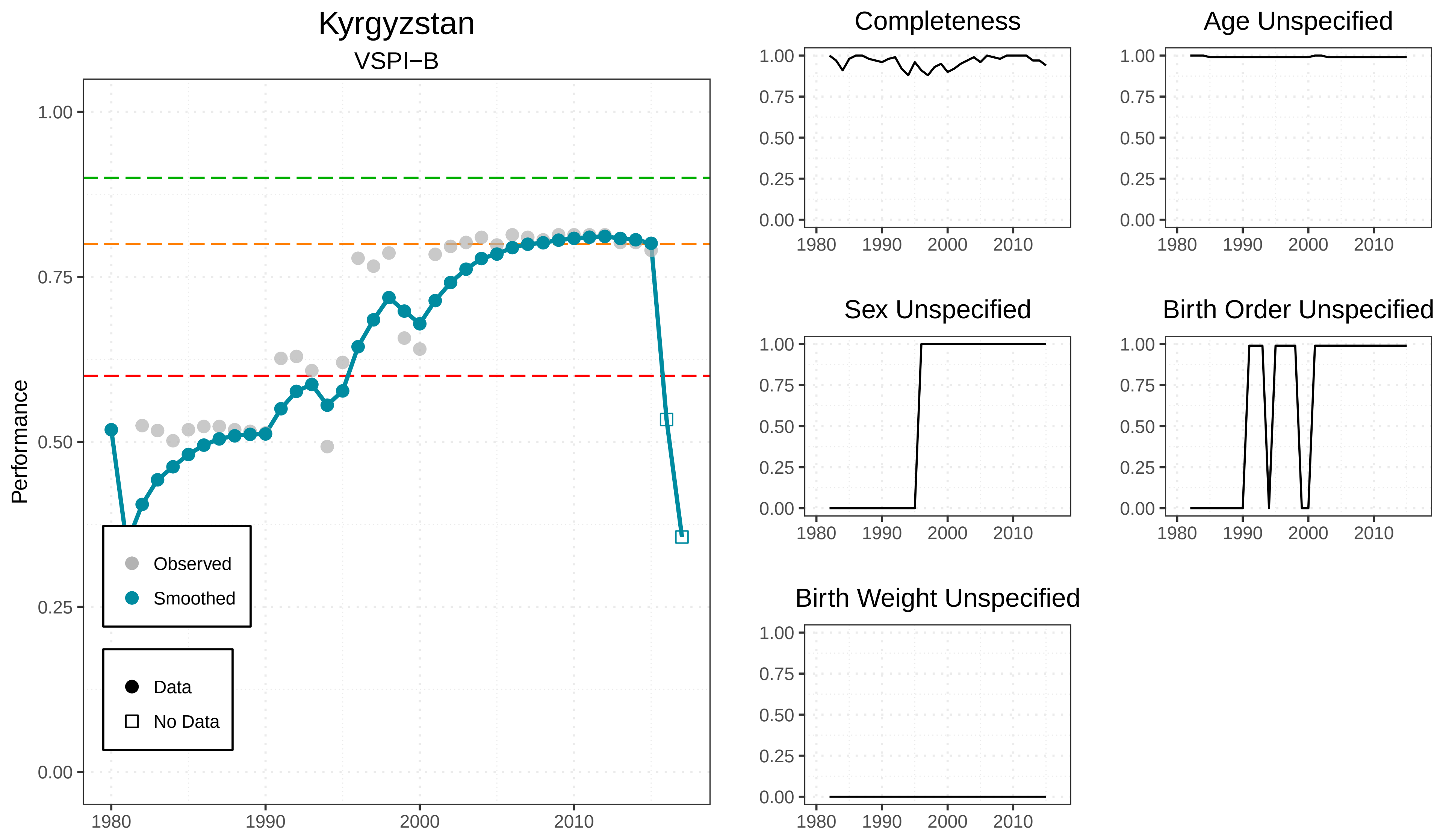

Kyrgyzstan
VSPI−B
Performance
1.00
0.75
0.50
0.25
0.00
1980
1990
2000
2010
Observed
Smoothed
Data
No Data
Completeness
Age Unspecified
Sex Unspecified
Birth Order Unspecified
Birth Weight Unspecified

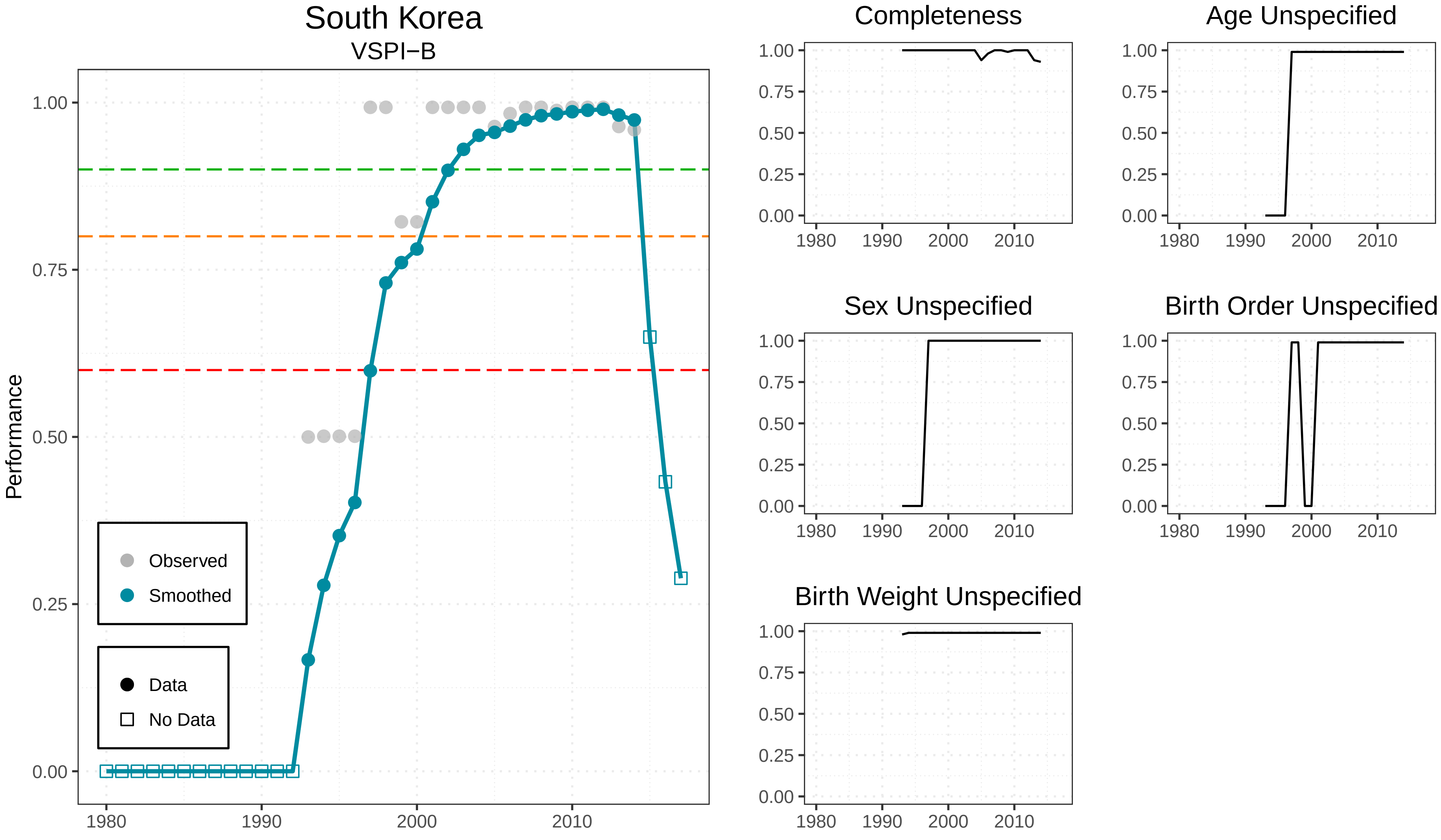

South Korea
VSPI−B
Performance
1.00
0.75
0.50
0.25
0.00
1980
1990
2000
2010
Observed
Smoothed
Data
No Data
Completeness
Age Unspecified
Sex Unspecified
Birth Order Unspecified
Birth Weight Unspecified

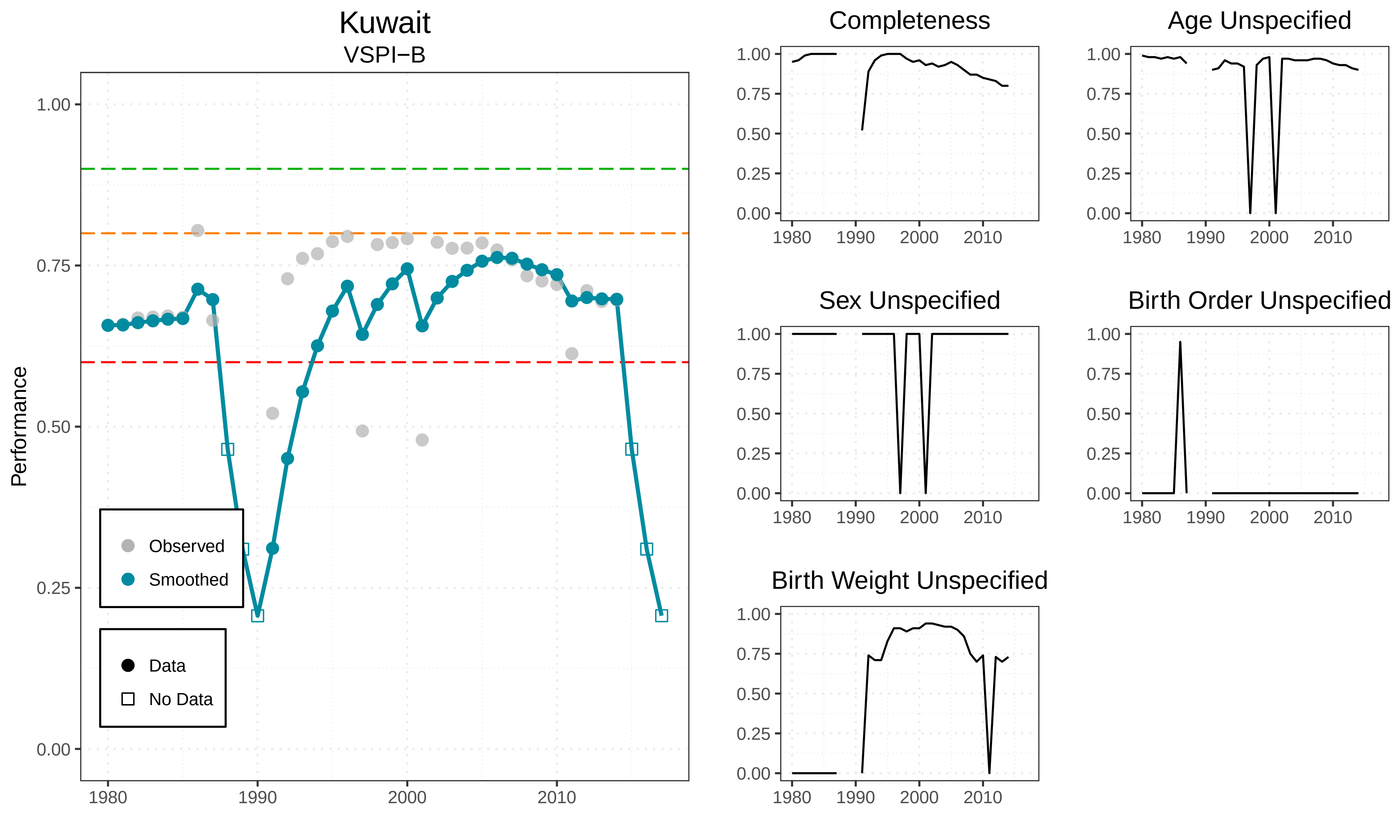

Kuwait
VSPI−B
Performance
1.00
0.75
0.50
0.25
0.00
1980
1990
2000
2010
Observed
Smoothed
Data
No Data
Completeness
Age Unspecified
Sex Unspecified
Birth Order Unspecified
Birth Weight Unspecified

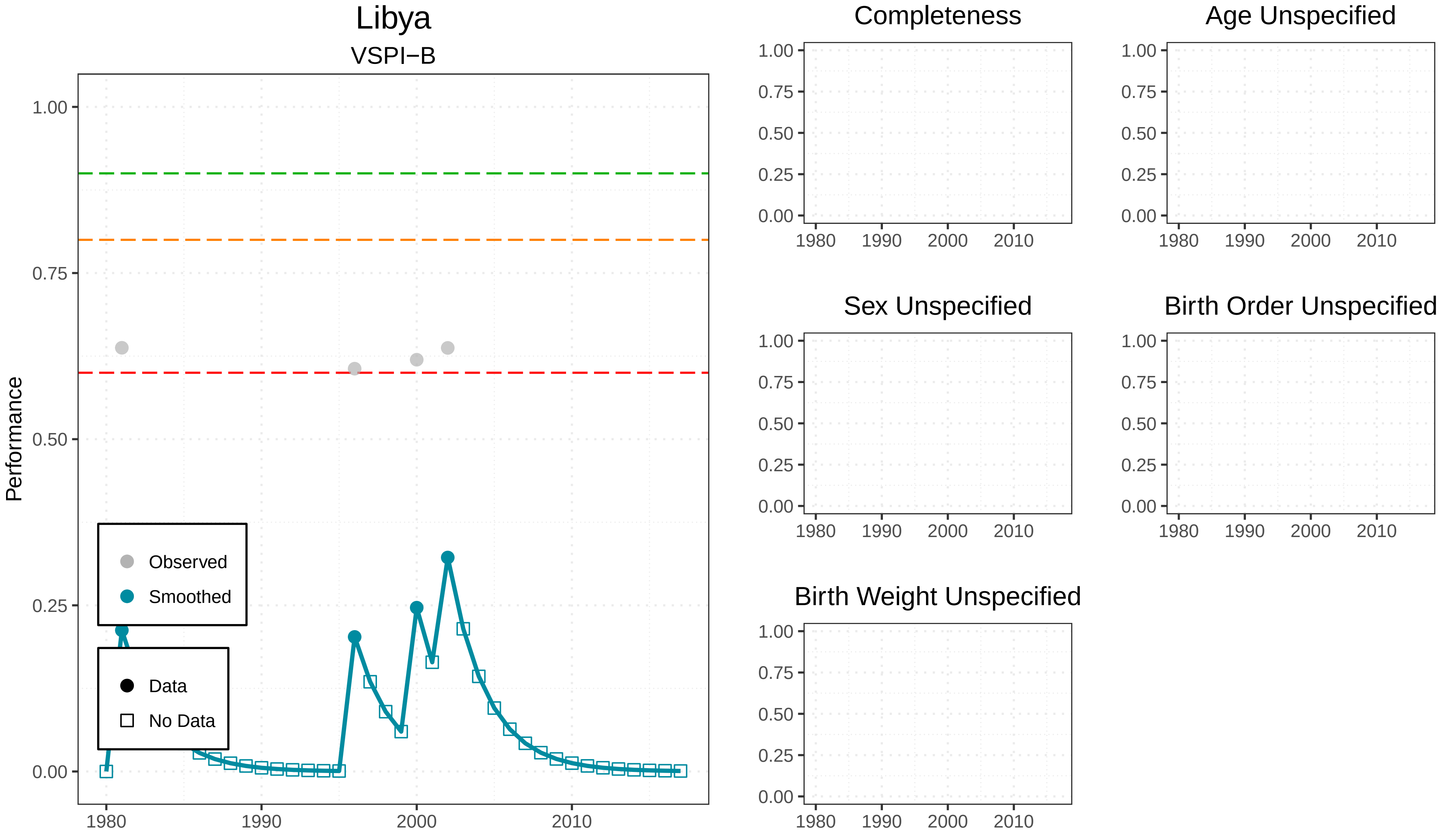

Libya
VSPI−B
Performance
Observed
Smoothed
Data
No Data
Completeness
Age Unspecified
Sex Unspecified
Birth Order Unspecified
Birth Weight Unspecified

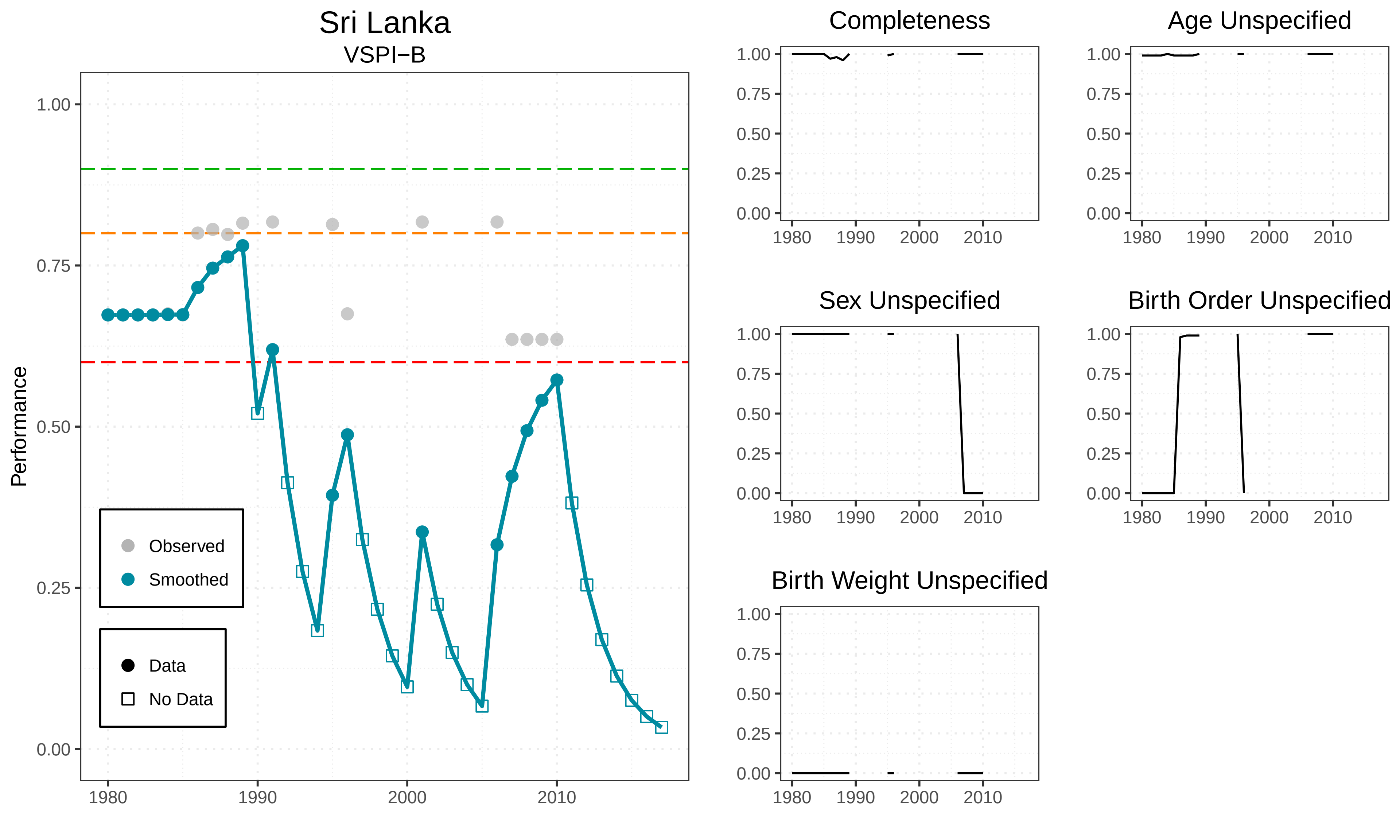

Sri Lanka
VSPI−B
Performance
1.00
0.75
0.50
0.25
0.00
1980
1990
2000
2010
Observed
Smoothed
Data
No Data
Completeness
Age Unspecified
Sex Unspecified
Birth Order Unspecified
Birth Weight Unspecified

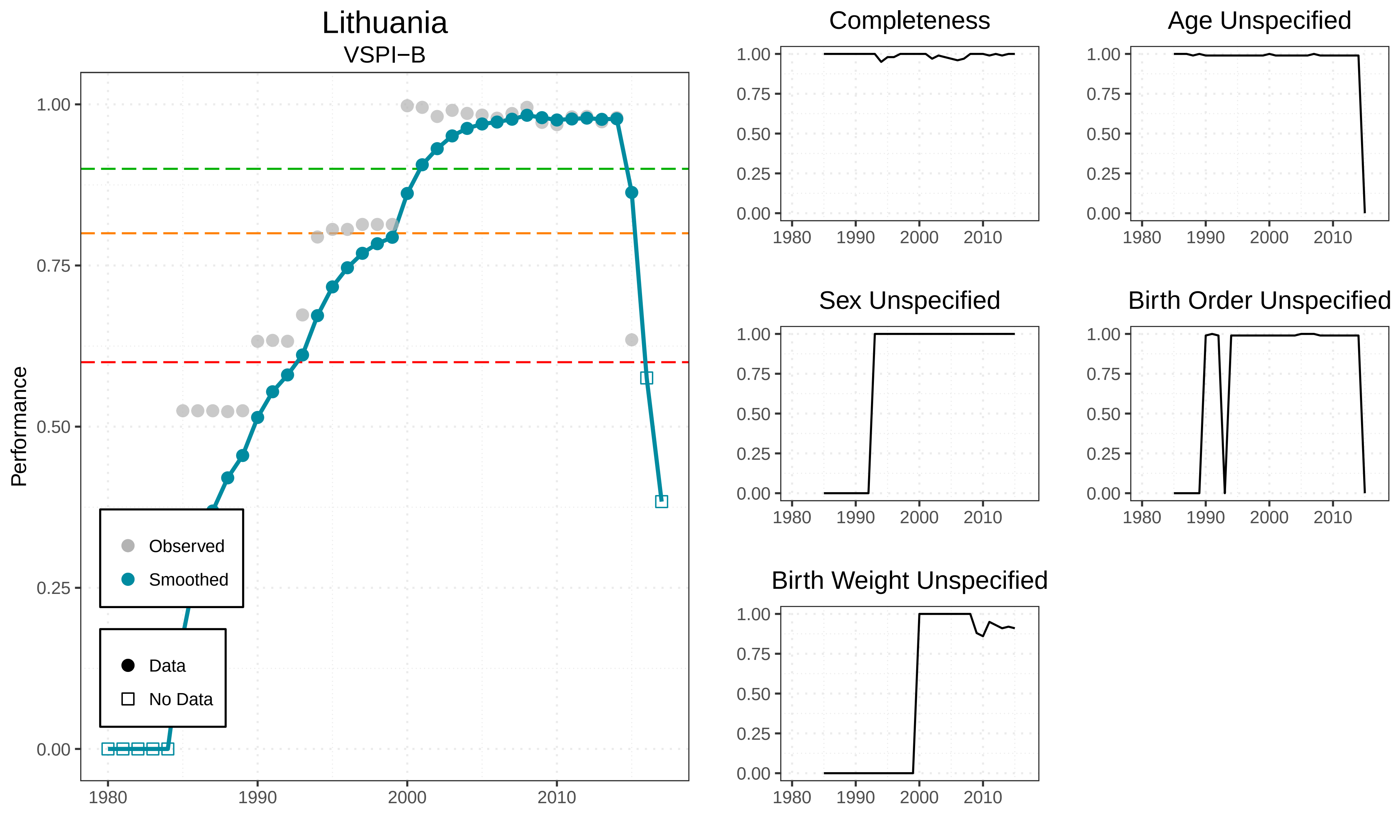

Lithuania
VSPI−B
Performance
Observed
Smoothed
Data
No Data
1.00
0.75
0.50
0.25
0.00
1980
1990
2000
2010
Completeness
Age Unspecified
Sex Unspecified
Birth Order Unspecified
Birth Weight Unspecified

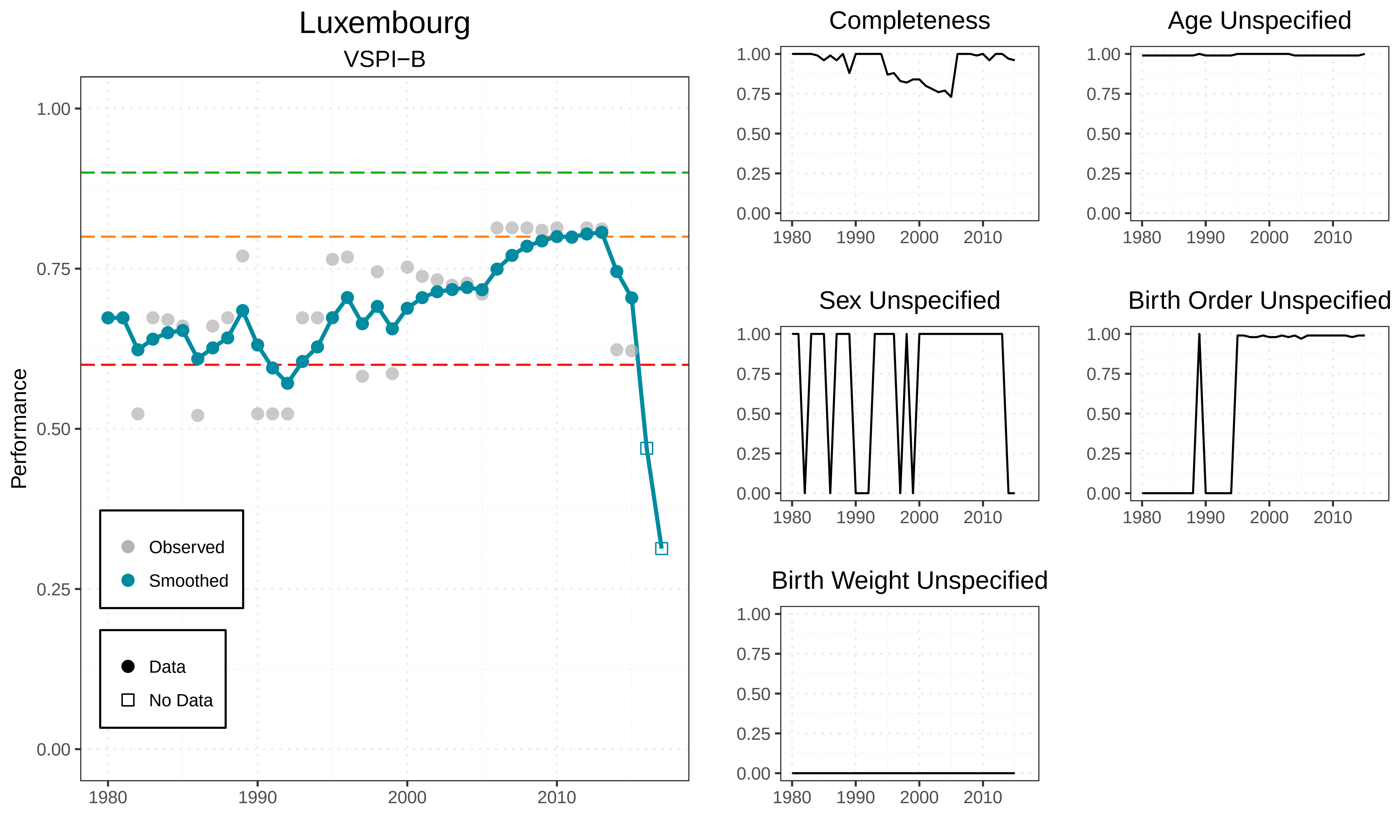

Luxembourg
VSPI−B
Performance
1.00
0.75
0.50
0.25
0.00
1980
1990
2000
2010
Observed
Smoothed
Data
No Data
Completeness
Age Unspecified
Sex Unspecified
Birth Order Unspecified
Birth Weight Unspecified

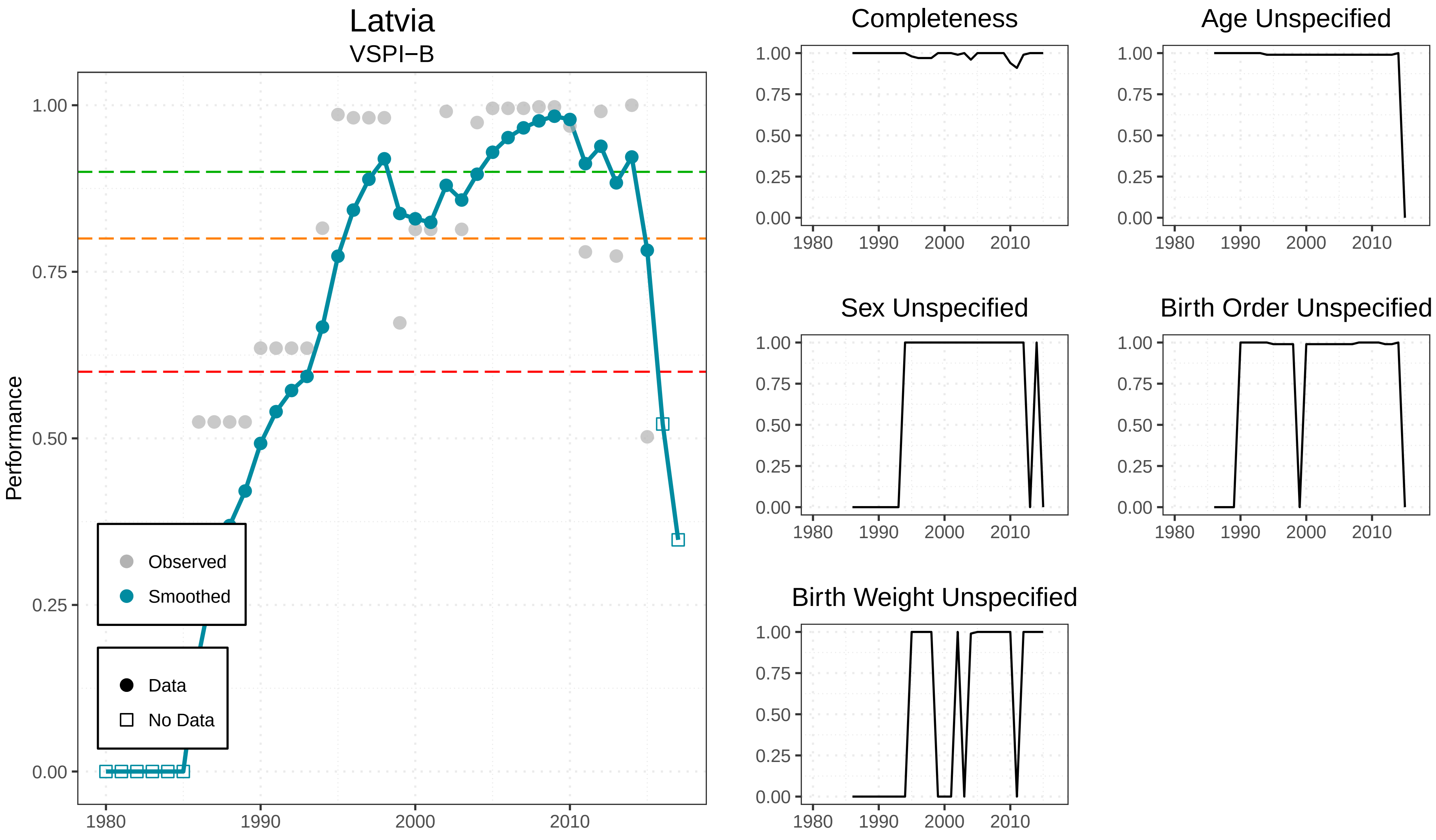

Latvia
VSPI−B
Performance
1.00
0.75
0.50
0.25
0.00
1980
1990
2000
2010
Observed
Smoothed
Data
No Data
Completeness
Age Unspecified
Sex Unspecified
Birth Order Unspecified
Birth Weight Unspecified

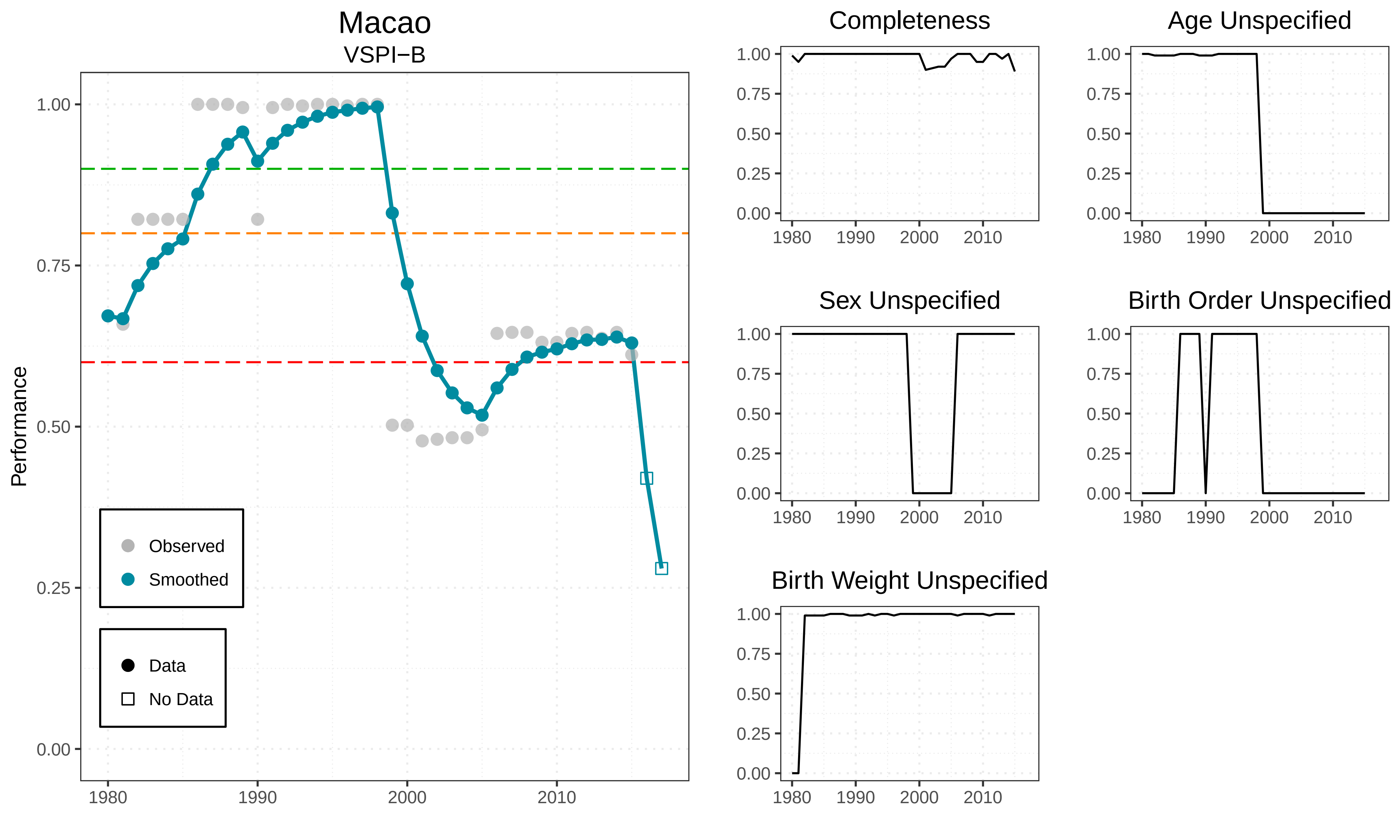

Macao
VSPI−B
Performance
Observed
Smoothed
Data
No Data
Completeness
Age Unspecified
Sex Unspecified
Birth Order Unspecified
Birth Weight Unspecified
1.00
0.75
0.50
0.25
0.00
1980
1990
2000
2010

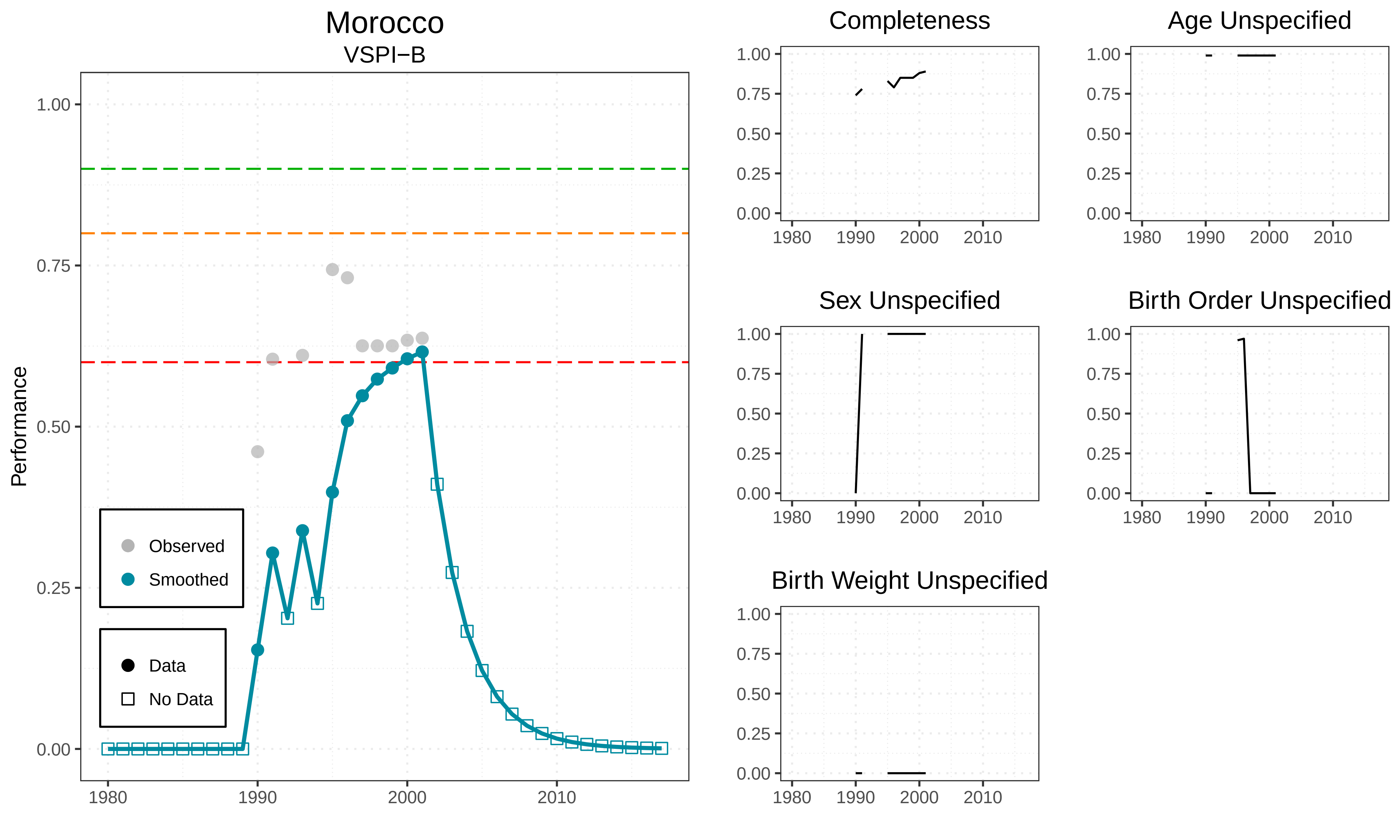

Morocco
VSPI−B
Performance
1.00
0.75
0.50
0.25
0.00
1980
1990
2000
2010
Observed
Smoothed
Data
No Data
Completeness
Age Unspecified
Sex Unspecified
Birth Order Unspecified
Birth Weight Unspecified

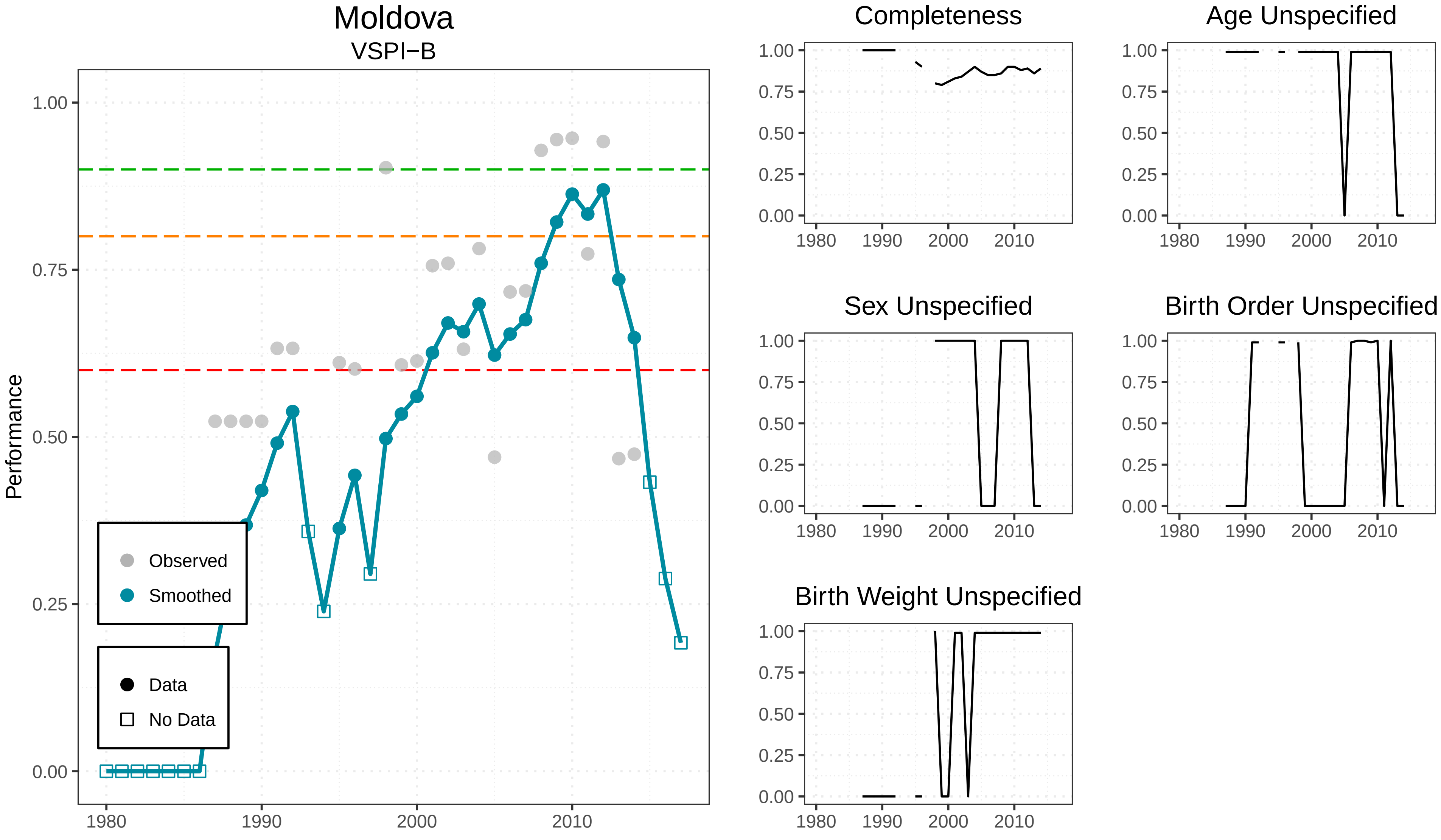

Moldova
VSPI−B
Performance
1.00
0.75
0.50
0.25
0.00
1980
1990
2000
2010
Observed
Smoothed
Data
No Data
Completeness
Age Unspecified
Sex Unspecified
Birth Order Unspecified
Birth Weight Unspecified

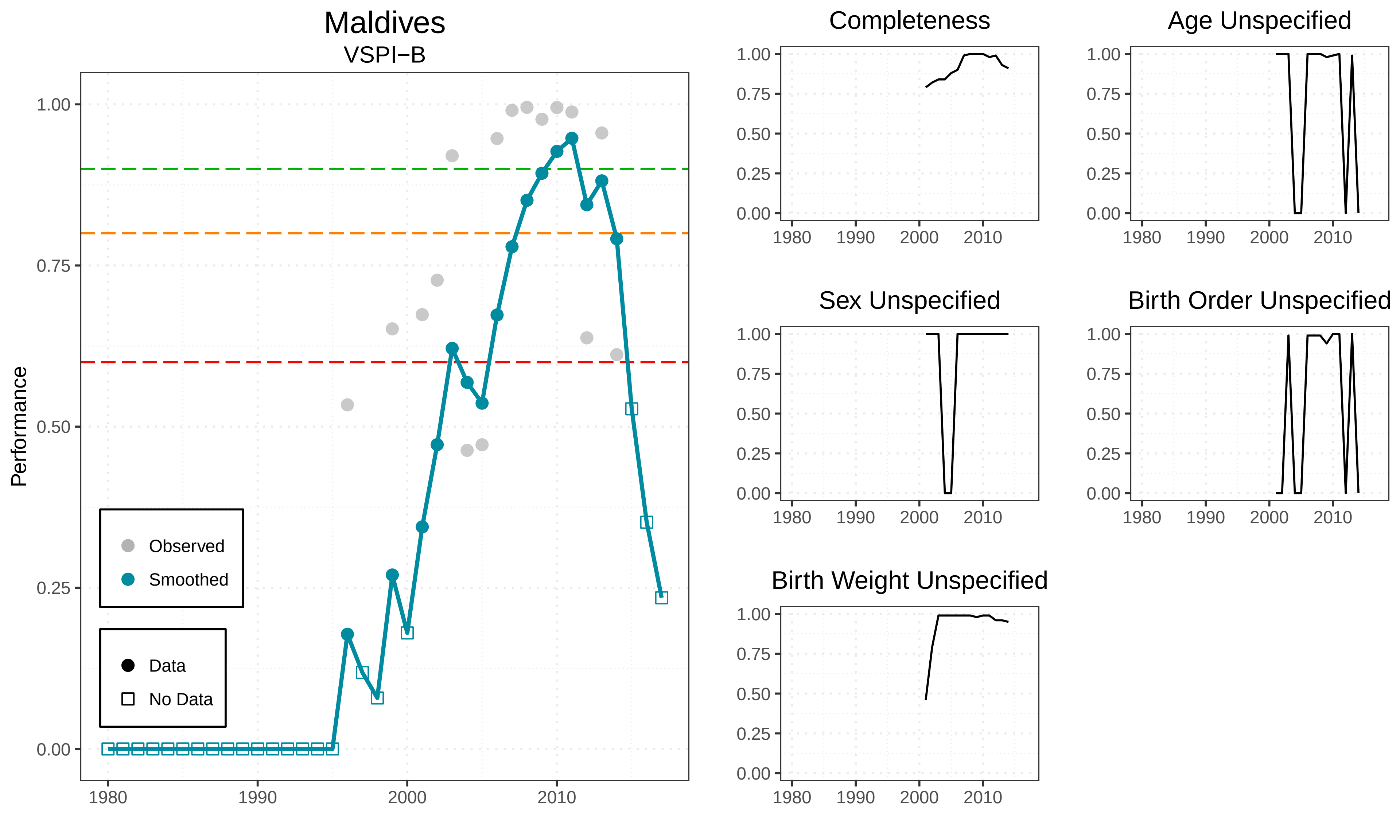

Maldives
VSPI−B
Performance
1.00
0.75
0.50
0.25
0.00
1980
1990
2000
2010
Observed
Smoothed
Data
No Data
Completeness
Age Unspecified
Sex Unspecified
Birth Order Unspecified
Birth Weight Unspecified

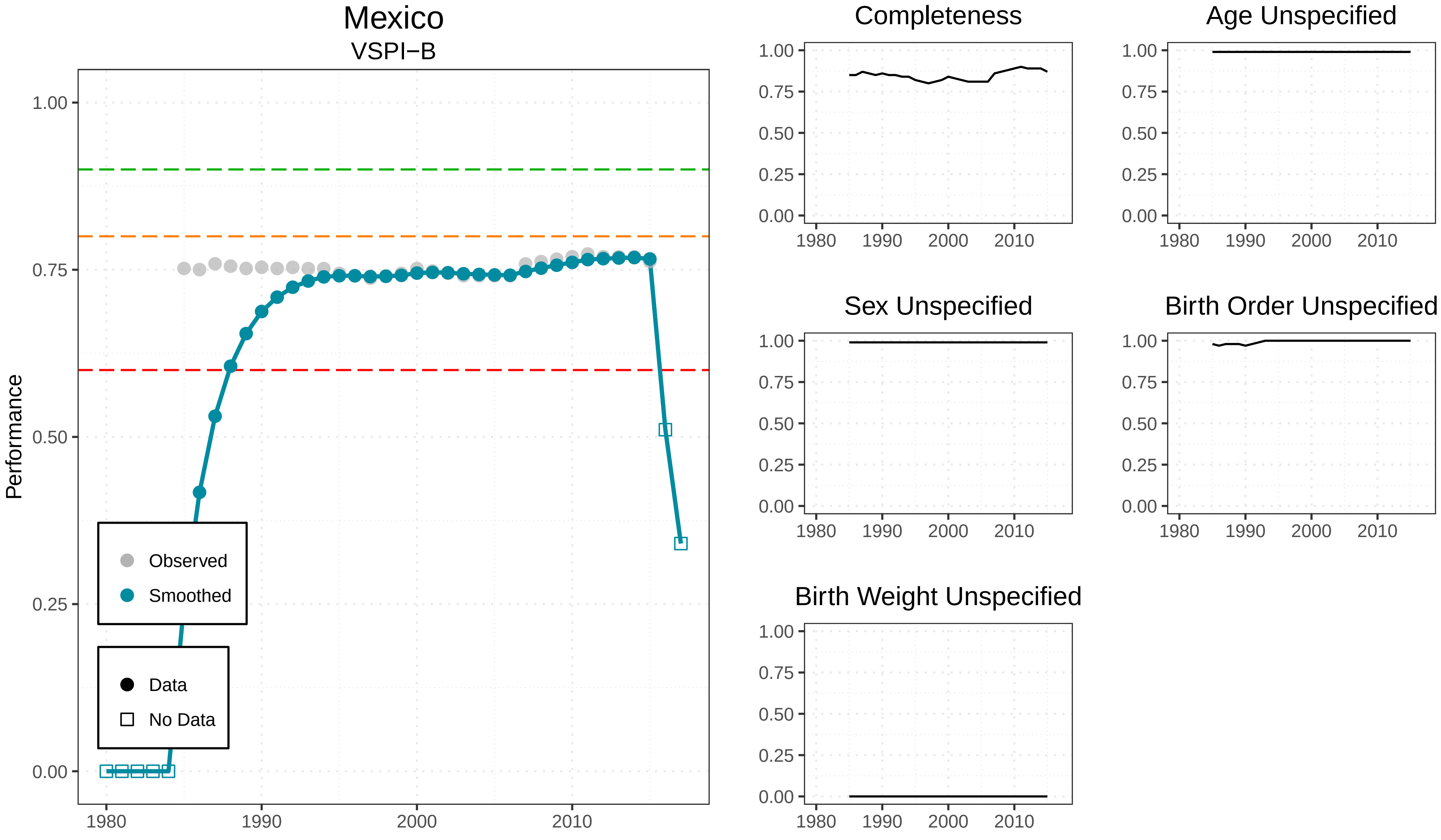

Mexico
VSPI−B
Performance
1.00
0.75
0.50
0.25
0.00
1980
1990
2000
2010
Observed
Smoothed
Data
No Data
Completeness
Age Unspecified
Sex Unspecified
Birth Order Unspecified
Birth Weight Unspecified

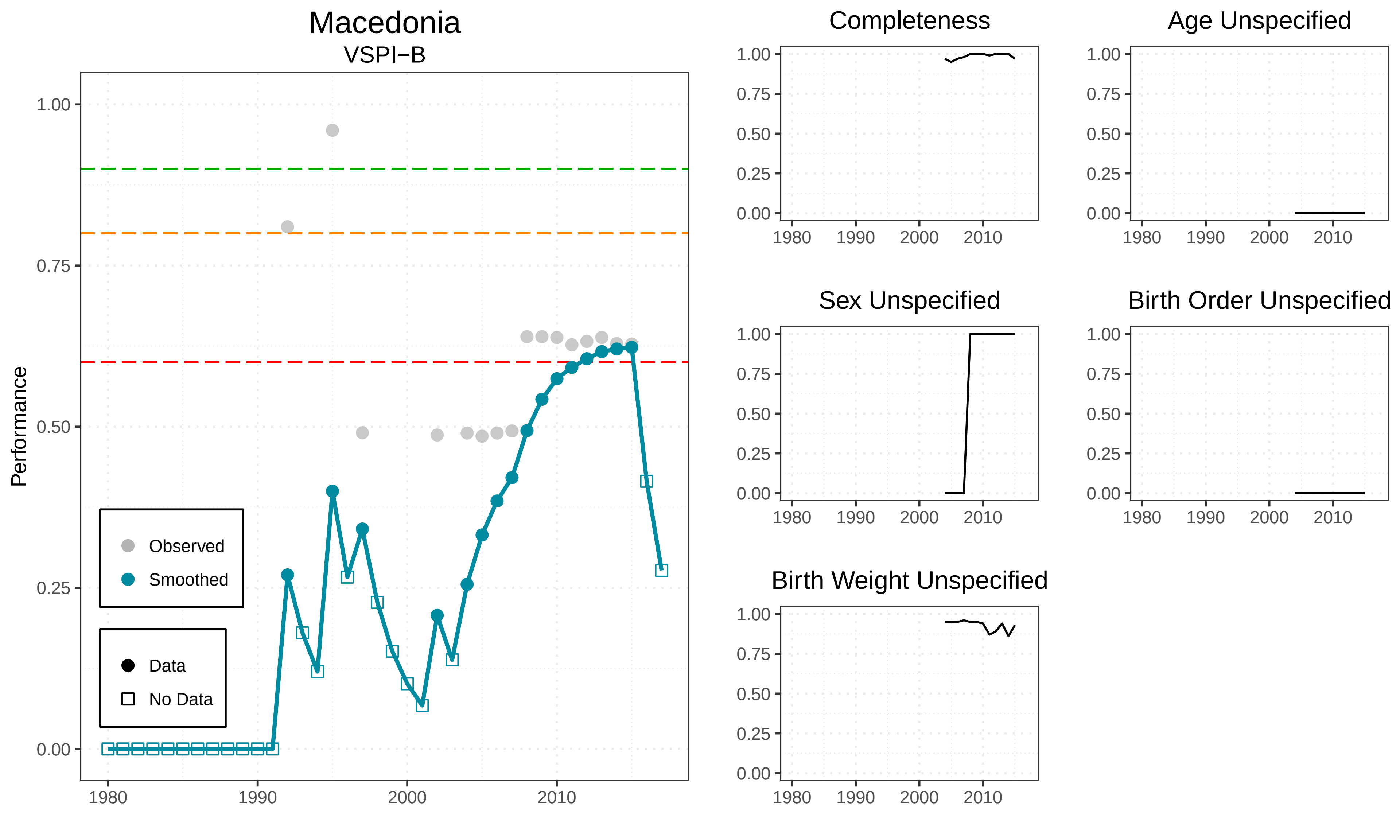

Macedonia
VSPI−B
Performance
1.00
0.75
0.50
0.25
0.00
1980
1990
2000
2010
Observed
Smoothed
Data
No Data
Completeness
Age Unspecified
Sex Unspecified
Birth Order Unspecified
Birth Weight Unspecified

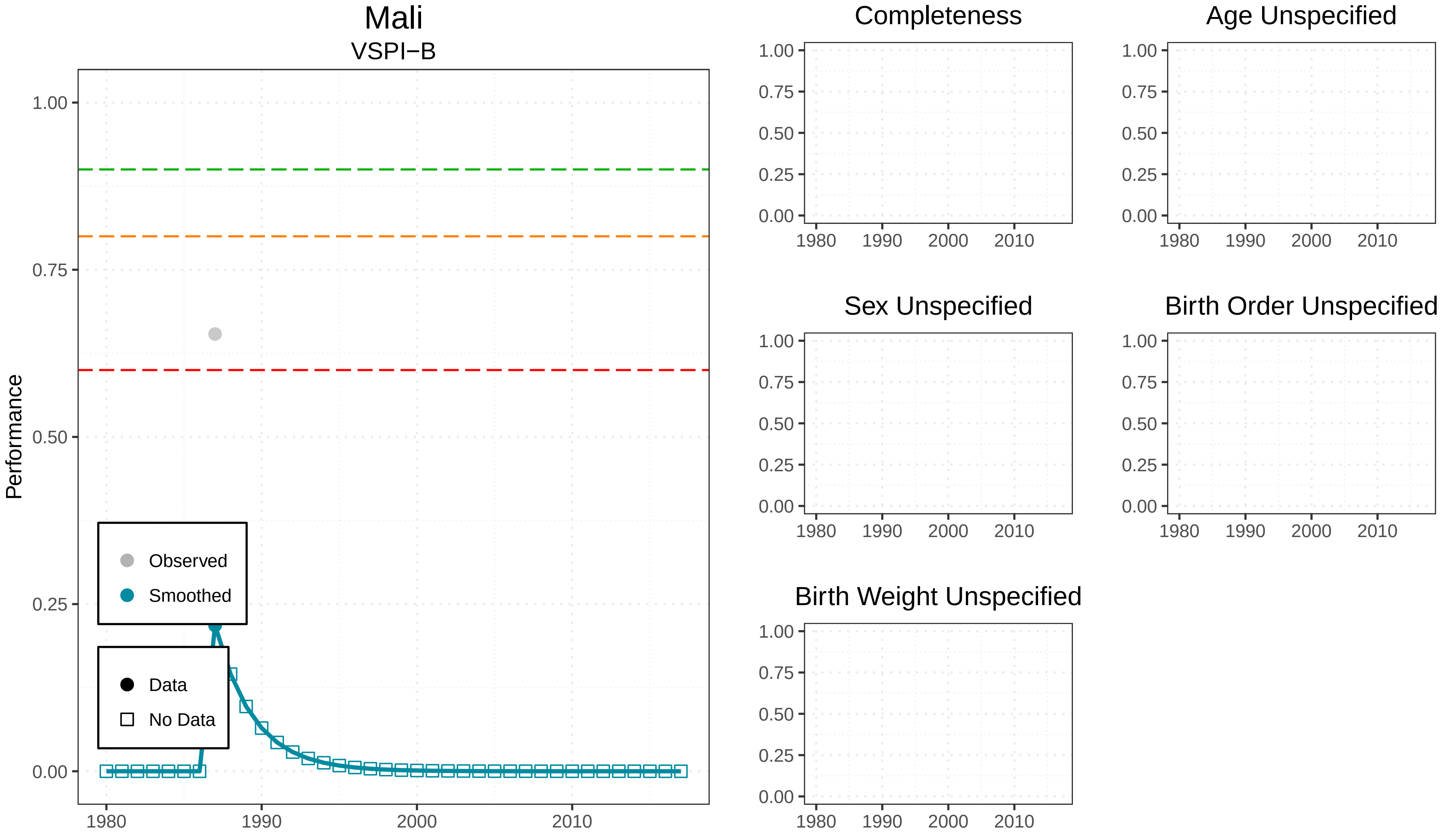

Mali
VSPI−B
Performance
1.00
0.75
0.50
0.25
0.00
1980
1990
2000
2010
Observed
Smoothed
Data
No Data
Completeness
Age Unspecified
Sex Unspecified
Birth Order Unspecified
Birth Weight Unspecified

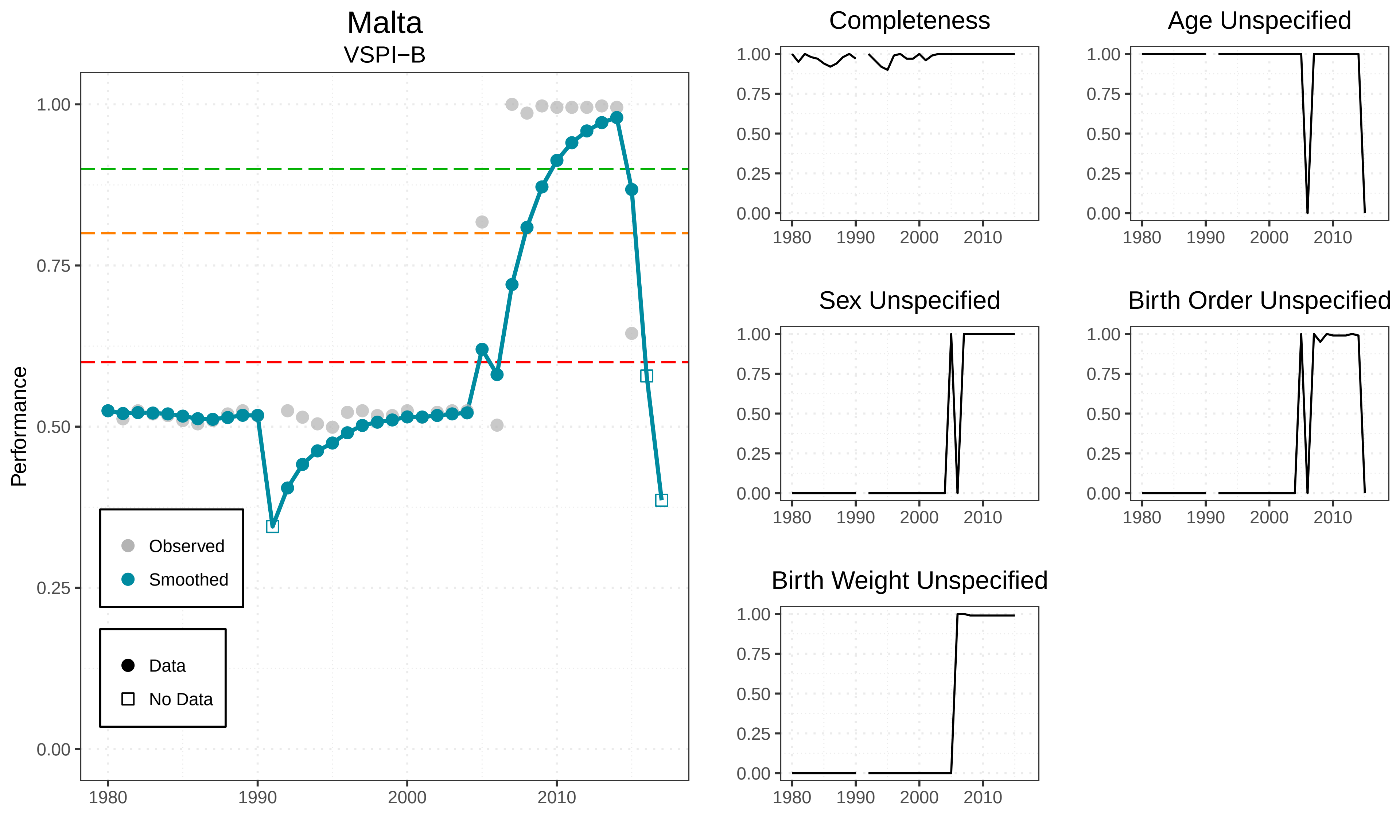

Malta
VSPI−B
Performance
1.00
0.75
0.50
0.25
0.00
1980
1990
2000
2010
Observed
Smoothed
Data
No Data
Completeness
Age Unspecified
Sex Unspecified
Birth Order Unspecified
Birth Weight Unspecified

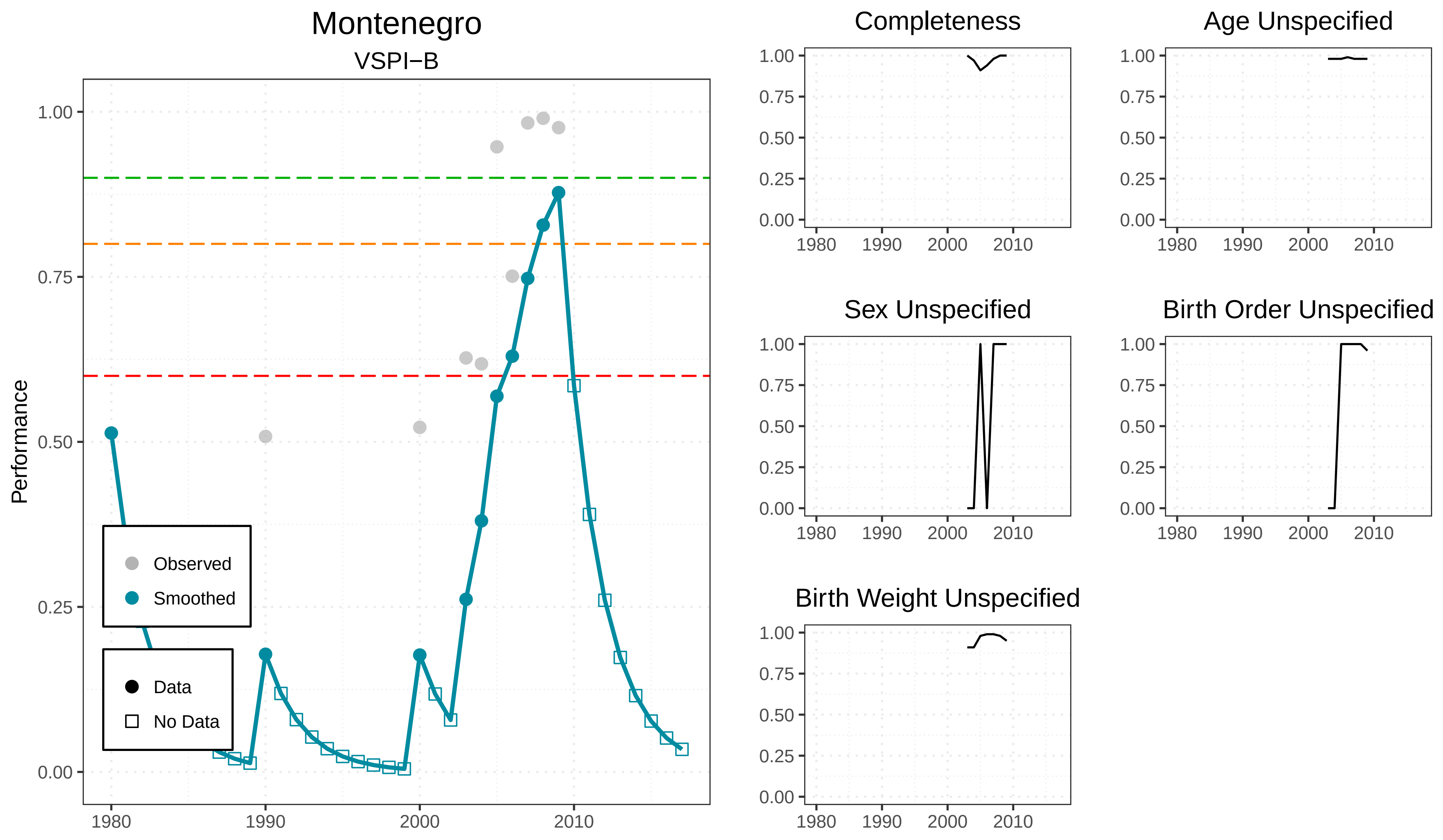

Montenegro
VSPI−B
Performance
Observed
Smoothed
Data
No Data
Completeness
Age Unspecified
Sex Unspecified
Birth Order Unspecified
Birth Weight Unspecified

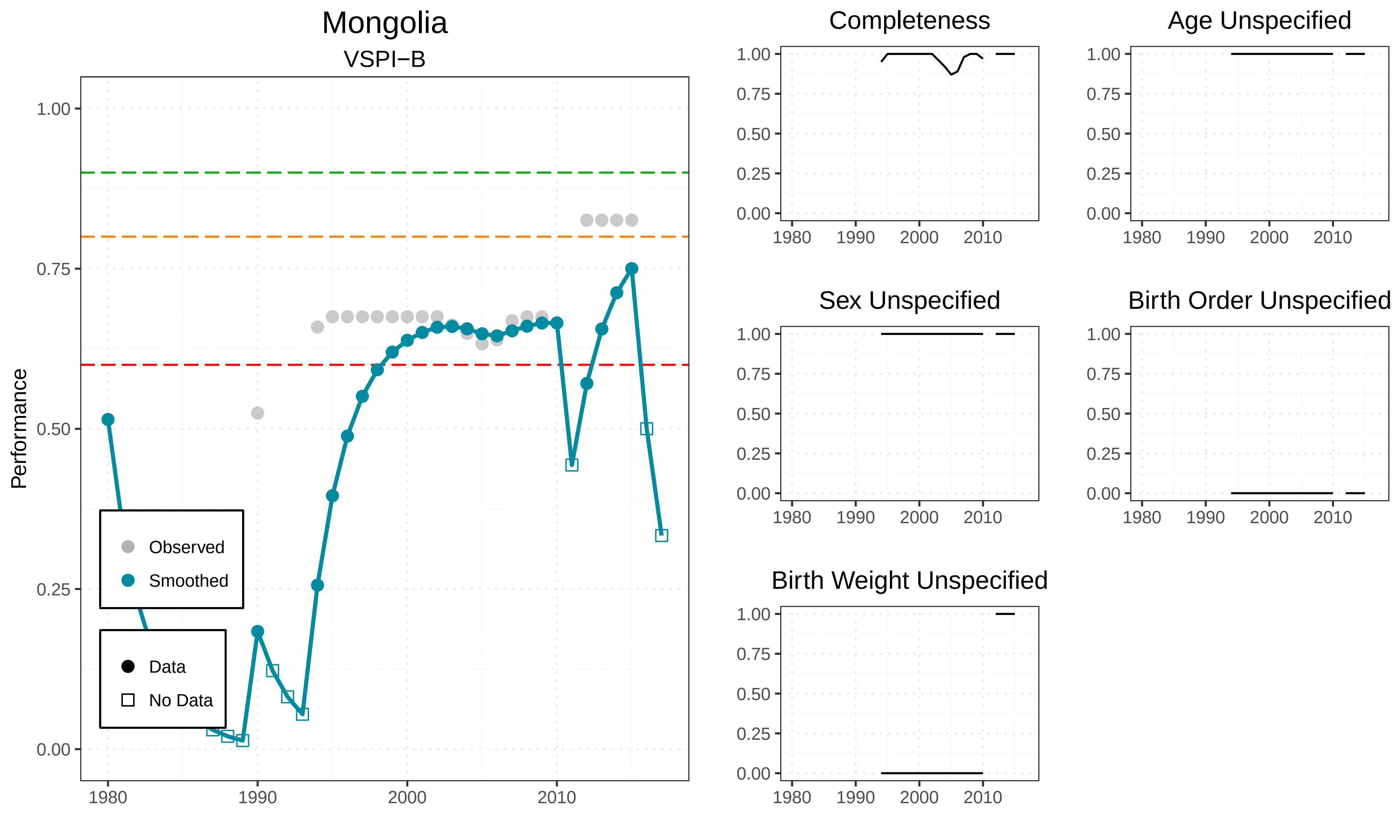

Mongolia
VSPI−B
Performance
1.00
0.75
0.50
0.25
0.00
1980
1990
2000
2010
Observed
Smoothed
Data
No Data
Completeness
Age Unspecified
Sex Unspecified
Birth Order Unspecified
Birth Weight Unspecified

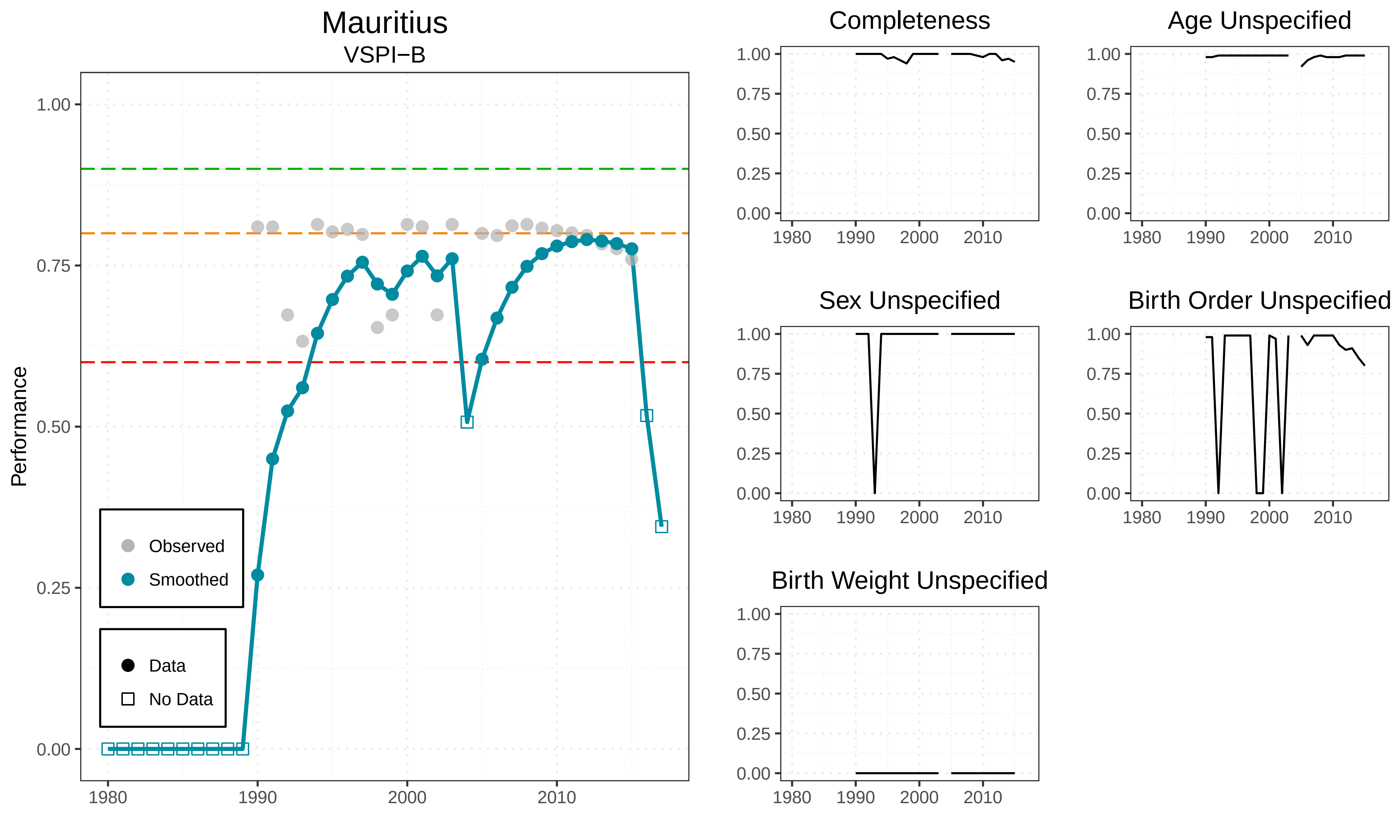
Mauritius
VSPI−B
Performance
Observed
Smoothed
Data
No Data
Completeness
Age Unspecified
Sex Unspecified
Birth Order Unspecified
Birth Weight Unspecified

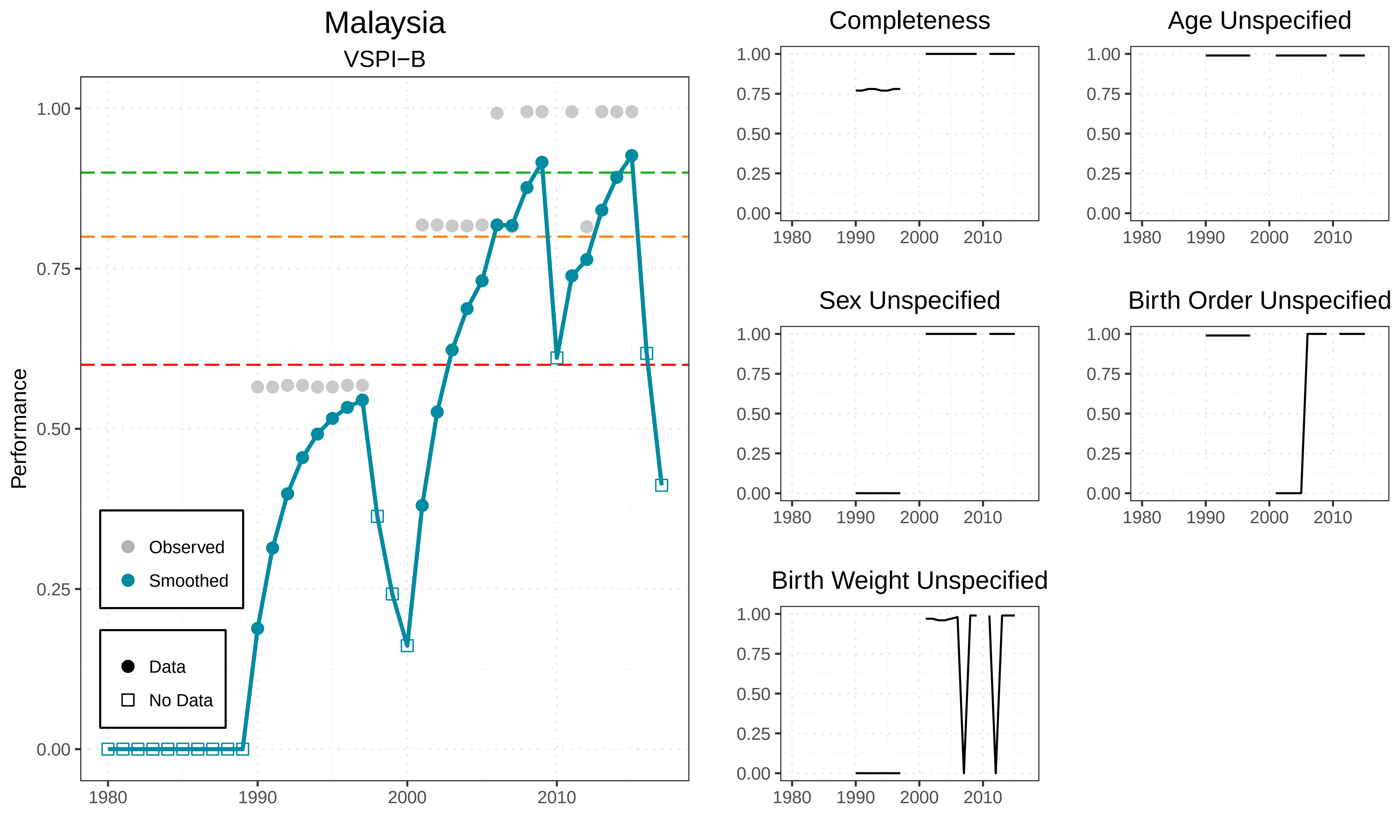
Malaysia
VSPI−B
Performance
Observed
Smoothed
Data
No Data
Completeness
Age Unspecified
Sex Unspecified
Birth Order Unspecified
Birth Weight Unspecified
1.00
0.75
0.50
0.25
0.00
1980
1990
2000
2010

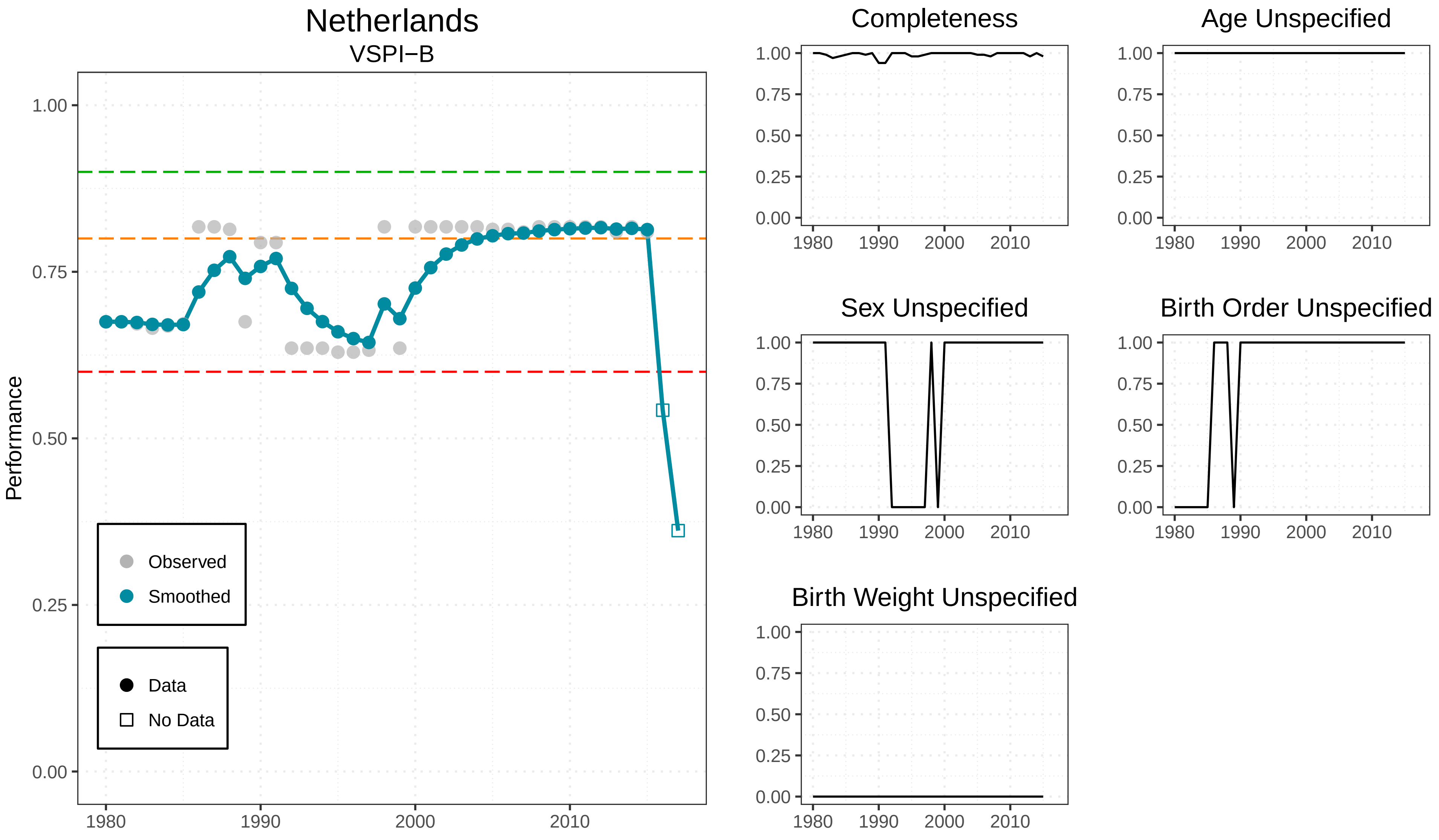

Netherlands
VSPI−B
Performance
1.00
0.75
0.50
0.25
0.00
1980
1990
2000
2010
Observed
Smoothed
Data
No Data
Completeness
Age Unspecified
Sex Unspecified
Birth Order Unspecified
Birth Weight Unspecified

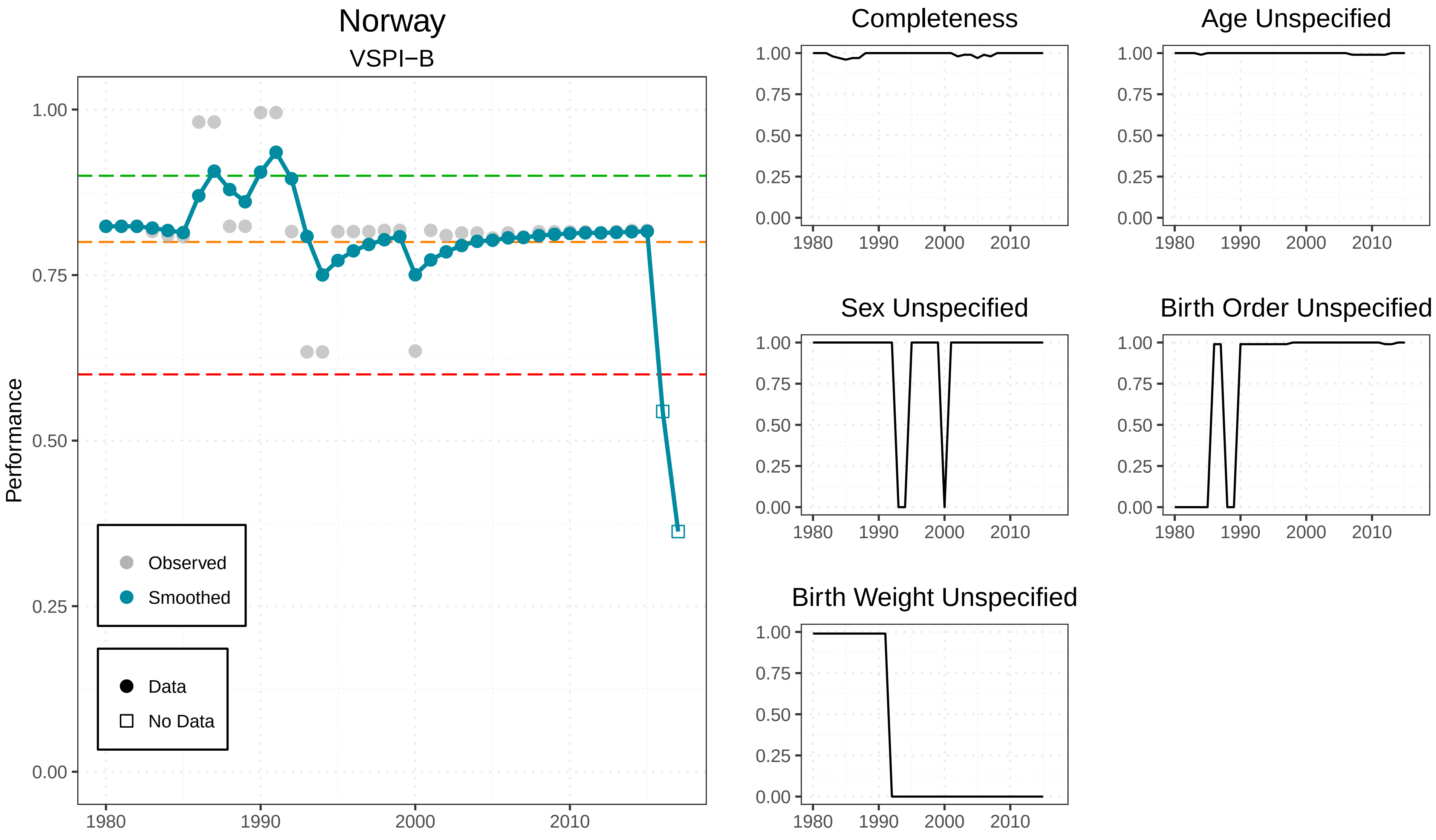
Norway
VSPI−B
Performance
1.00
0.75
0.50
0.25
0.00
1980
1990
2000
2010
Observed
Smoothed
Data
No Data
Completeness
Age Unspecified
Sex Unspecified
Birth Order Unspecified
Birth Weight Unspecified

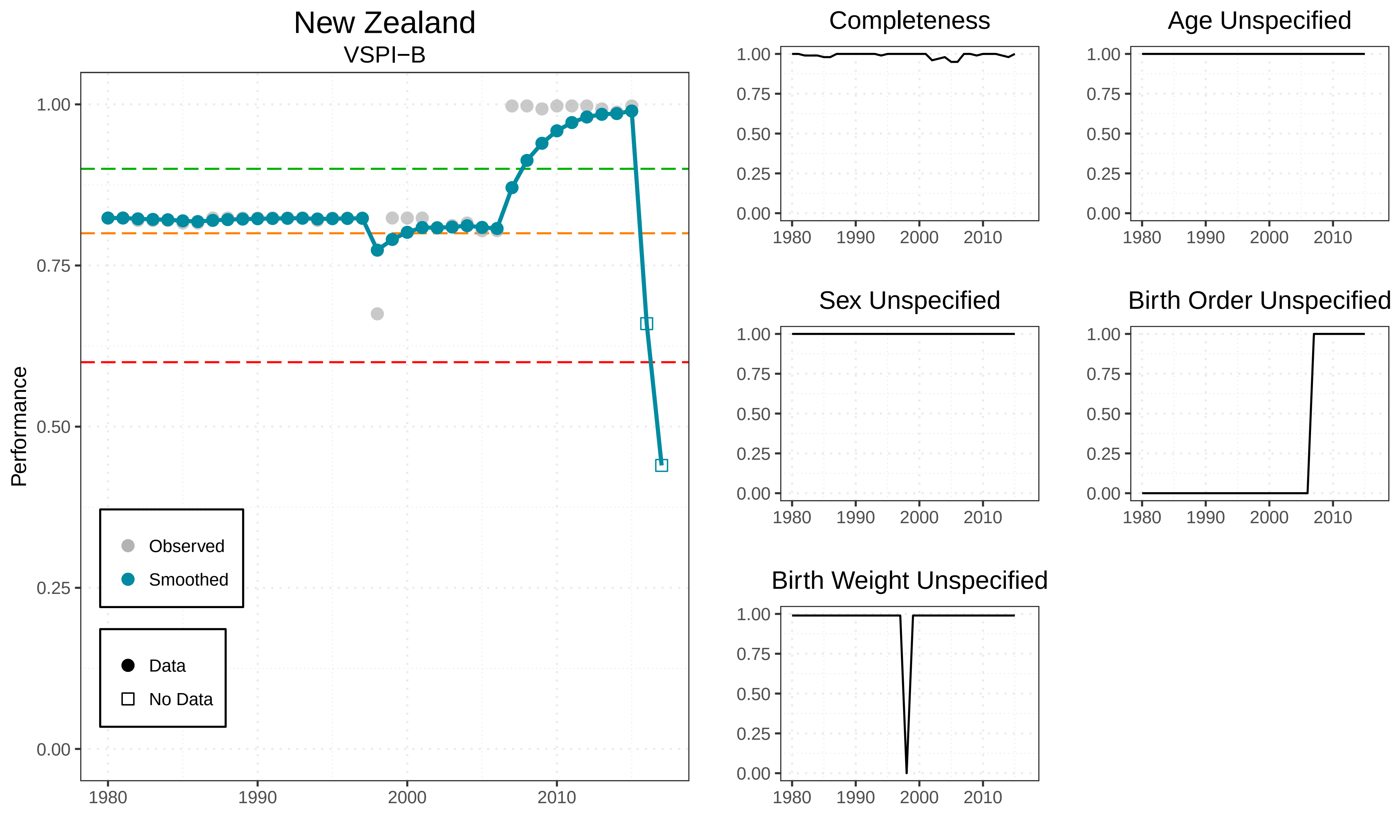
New Zealand
VSPI−B
Performance
Observed
Smoothed
Data
No Data
Completeness
Age Unspecified
Sex Unspecified
Birth Order Unspecified
Birth Weight Unspecified
1.00
0.75
0.50
0.25
0.00
1980
1990
2000
2010

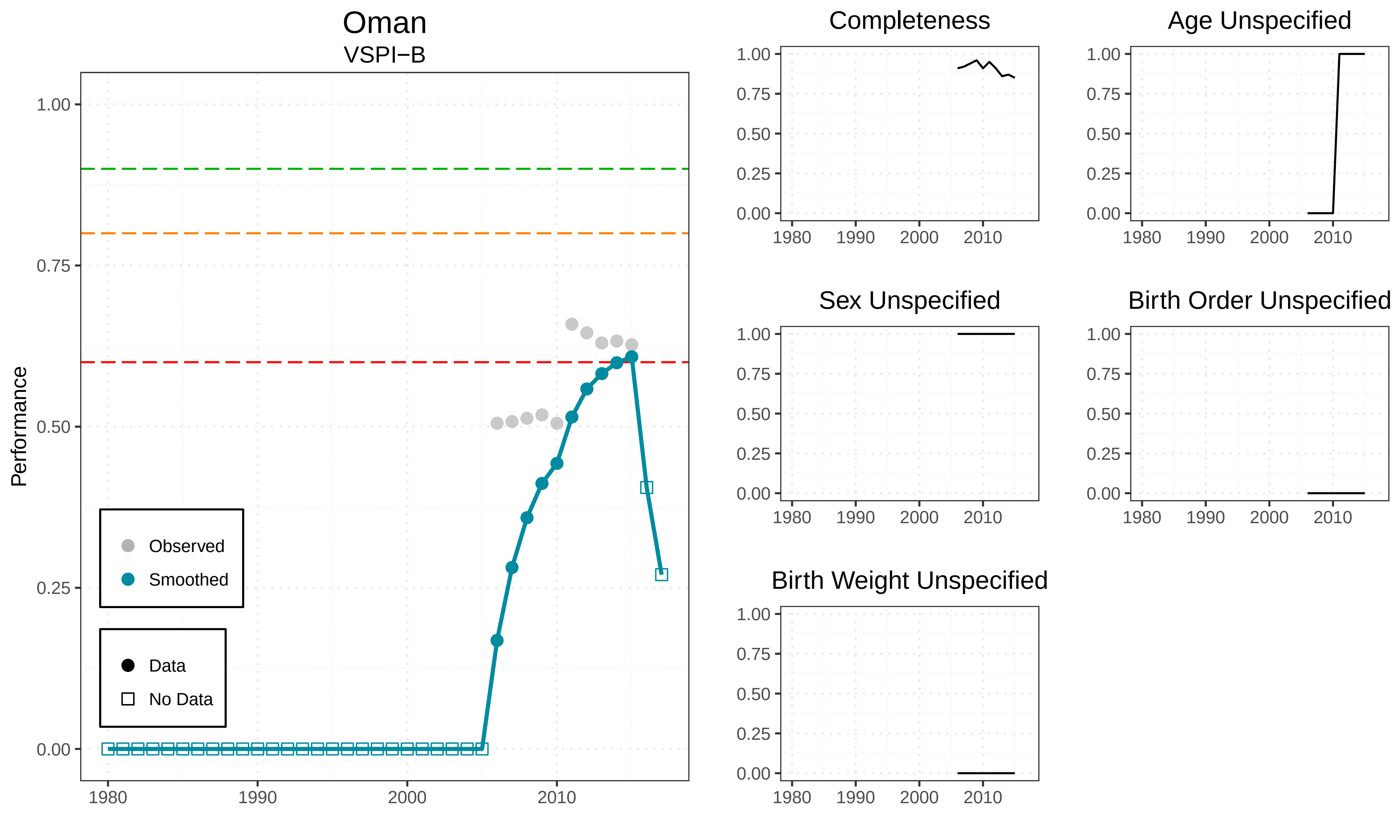
Oman
VSPI−B
Performance
Observed
Smoothed
Data
No Data
Completeness
Age Unspecified
Sex Unspecified
Birth Order Unspecified
Birth Weight Unspecified

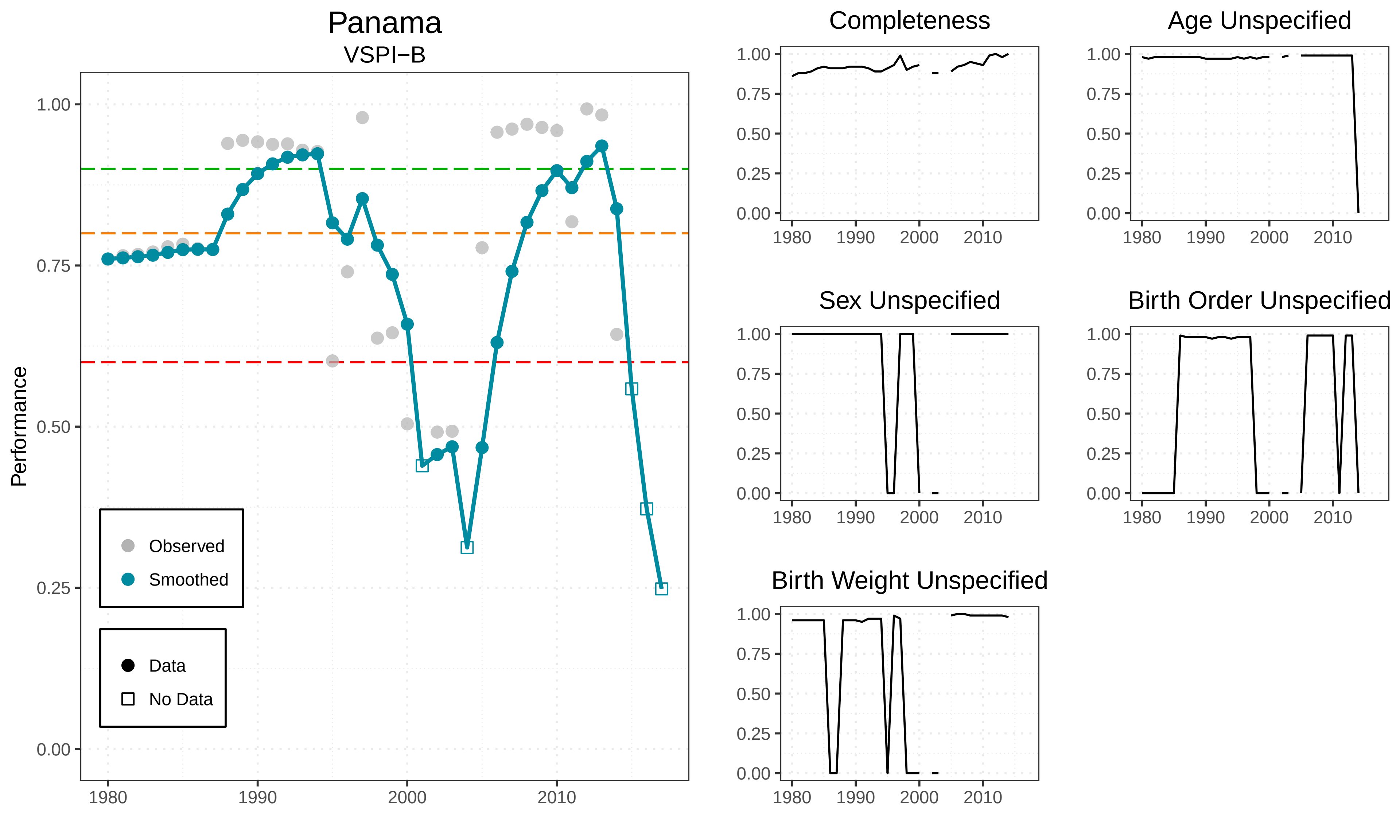

Panama
VSPI−B
Performance
1.00
0.75
0.50
0.25
0.00
1980
1990
2000
2010
Observed
Smoothed
Data
No Data
Completeness
Age Unspecified
Sex Unspecified
Birth Order Unspecified
Birth Weight Unspecified

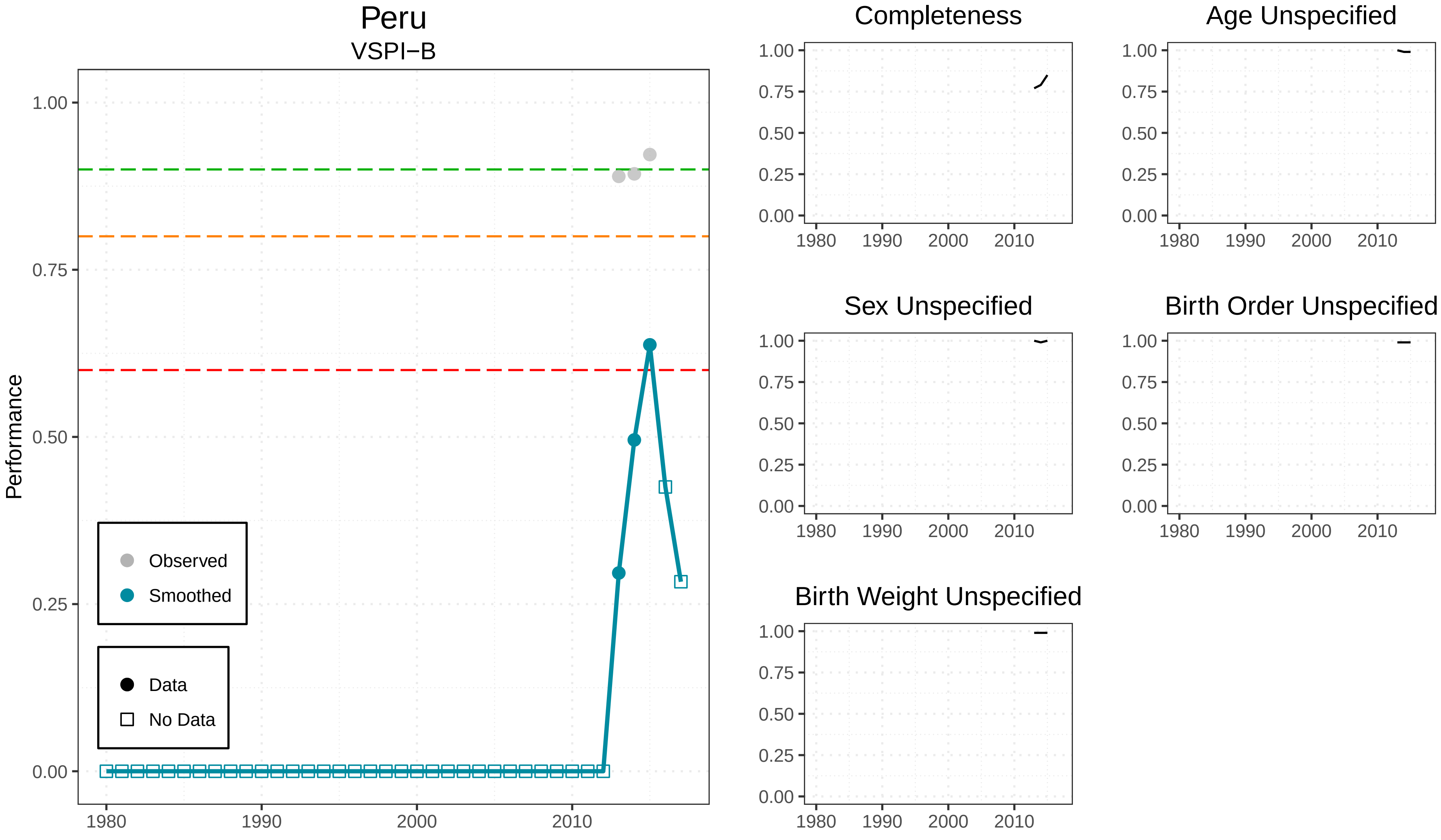

Peru
VSPI−B
Performance
1.00
0.75
0.50
0.25
0.00
1980
1990
2000
2010
Observed
Smoothed
Data
No Data
Completeness
Age Unspecified
Sex Unspecified
Birth Order Unspecified
Birth Weight Unspecified

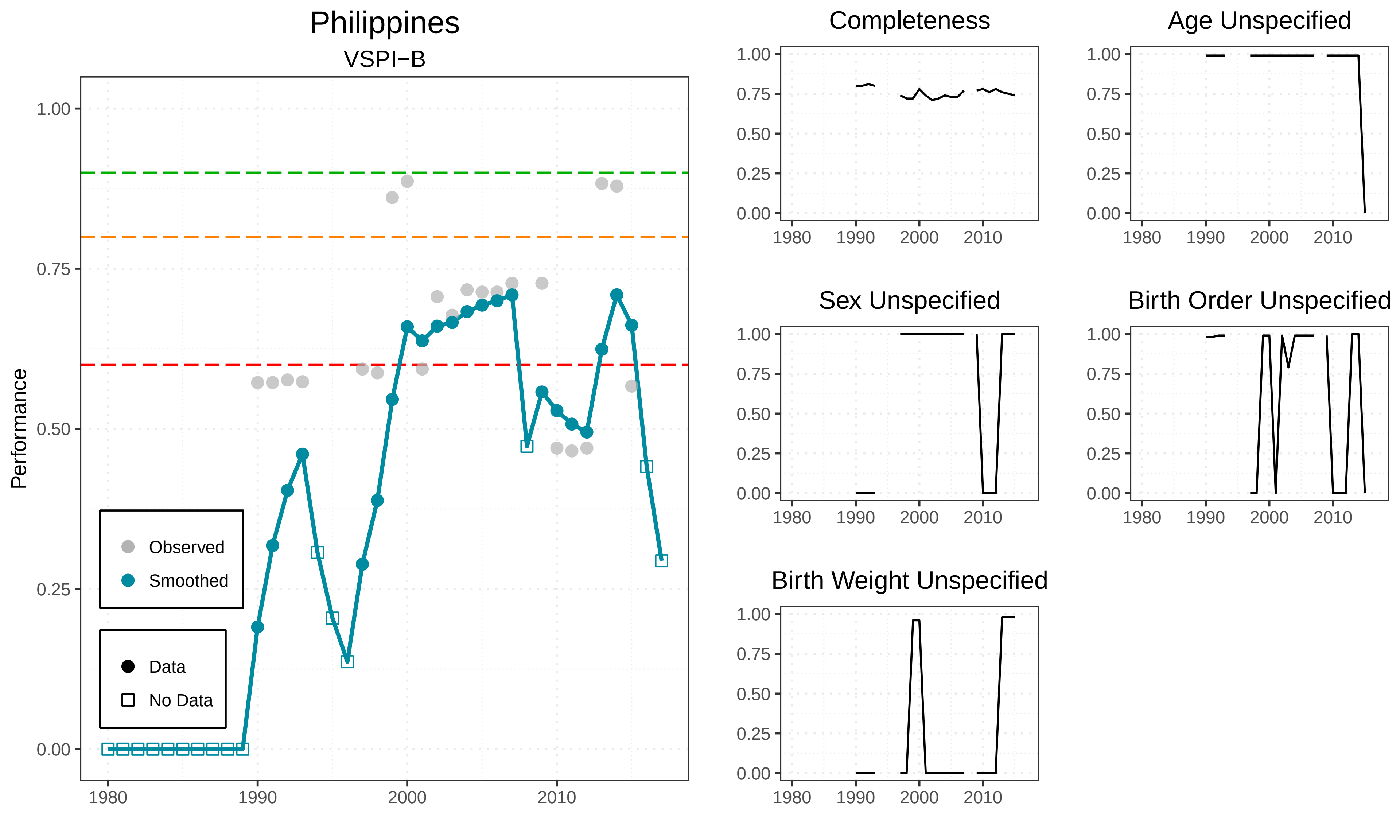

Philippines
VSPI−B
Performance
1.00
0.75
0.50
0.25
0.00
1980
1990
2000
2010
Observed
Smoothed
Data
No Data
Completeness
Age Unspecified
Sex Unspecified
Birth Order Unspecified
Birth Weight Unspecified

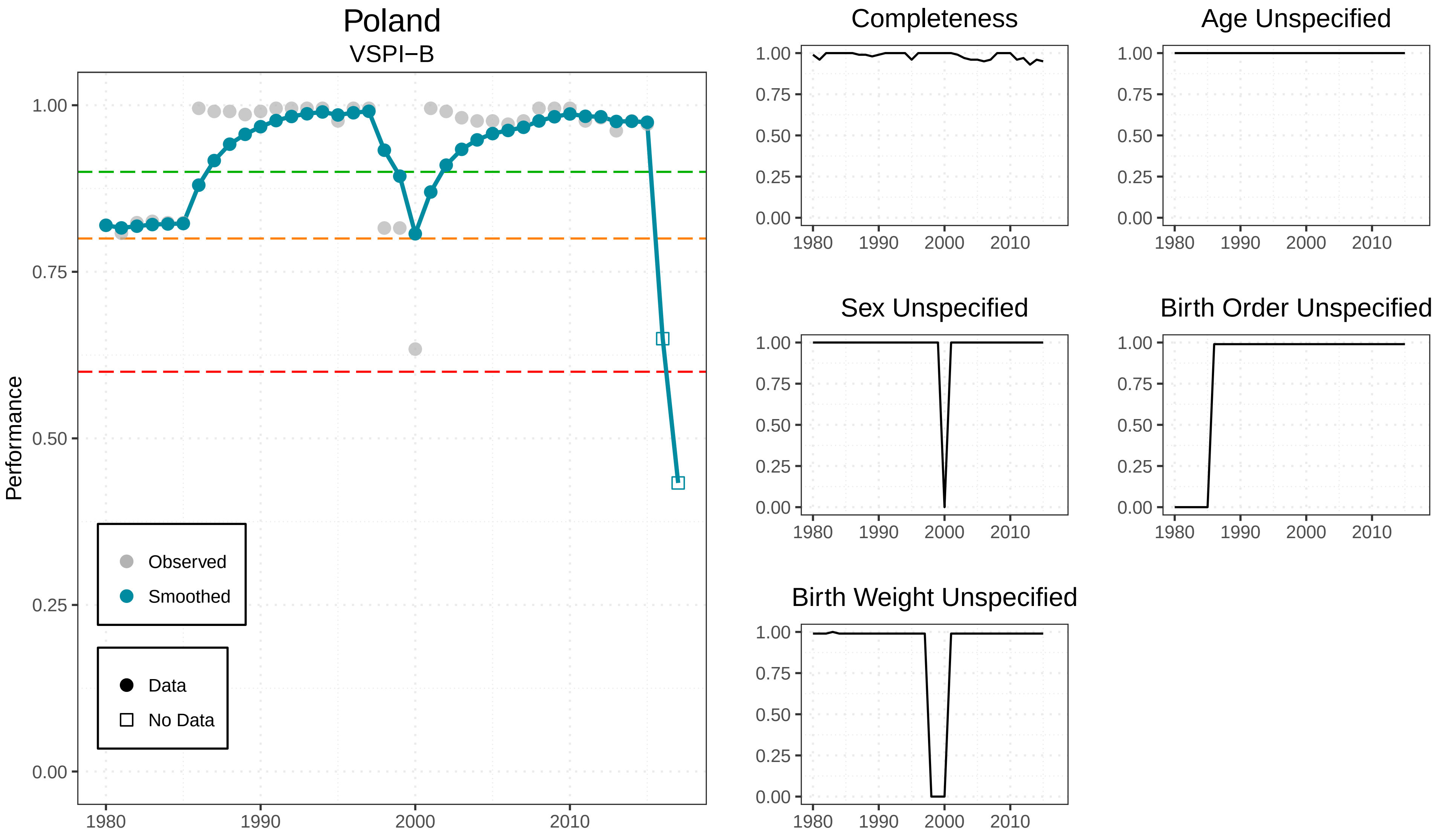

Poland
VSPI−B
Performance
1.00
0.75
0.50
0.25
0.00
1980
1990
2000
2010
Observed
Smoothed
Data
No Data
Completeness
Age Unspecified
Sex Unspecified
Birth Order Unspecified
Birth Weight Unspecified

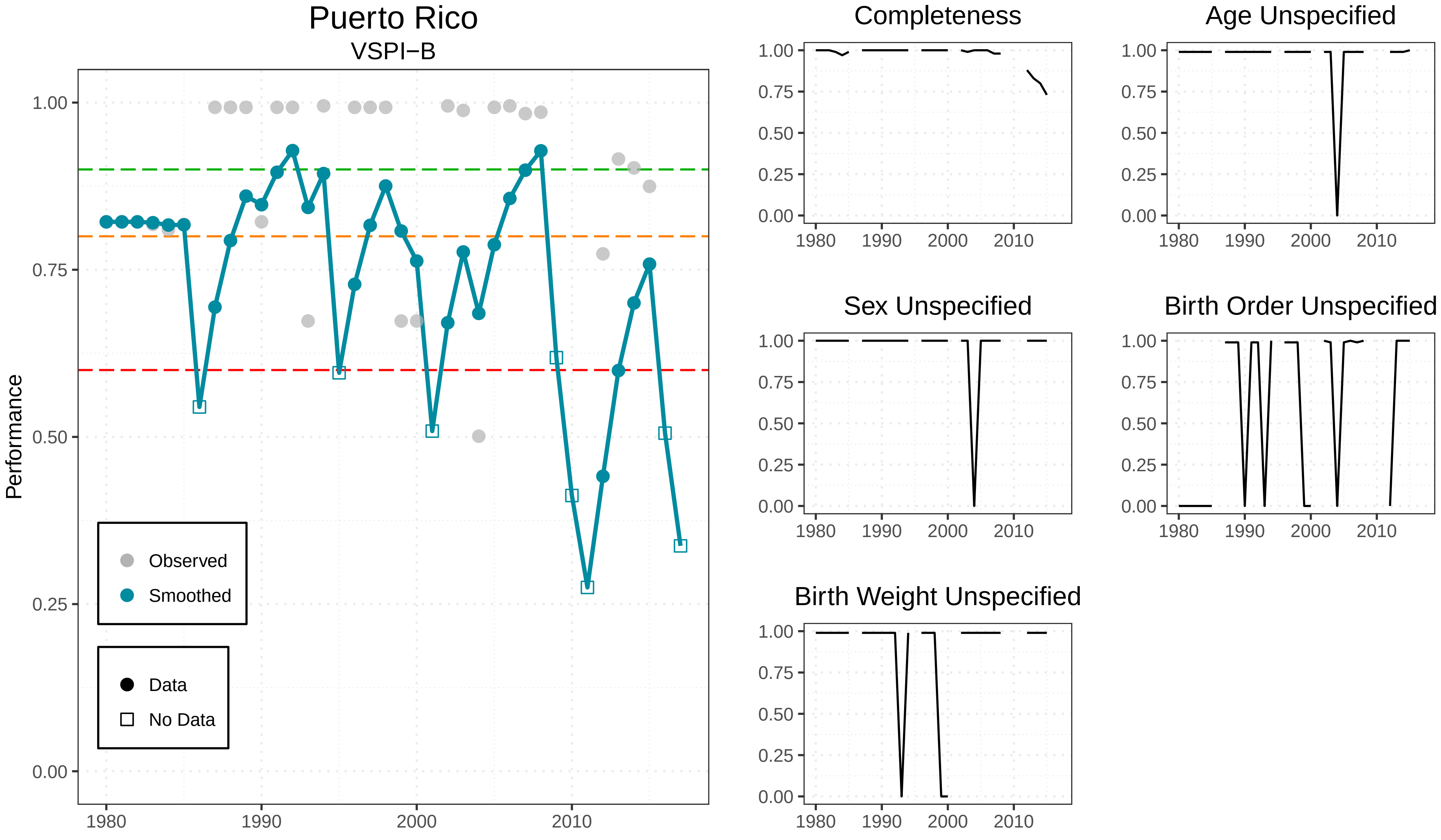
Puerto Rico
VSPI−B
Performance
1.00
0.75
0.50
0.25
0.00
1980
1990
2000
2010
Observed
Smoothed
Data
No Data
Completeness
Age Unspecified
Sex Unspecified
Birth Order Unspecified
Birth Weight Unspecified

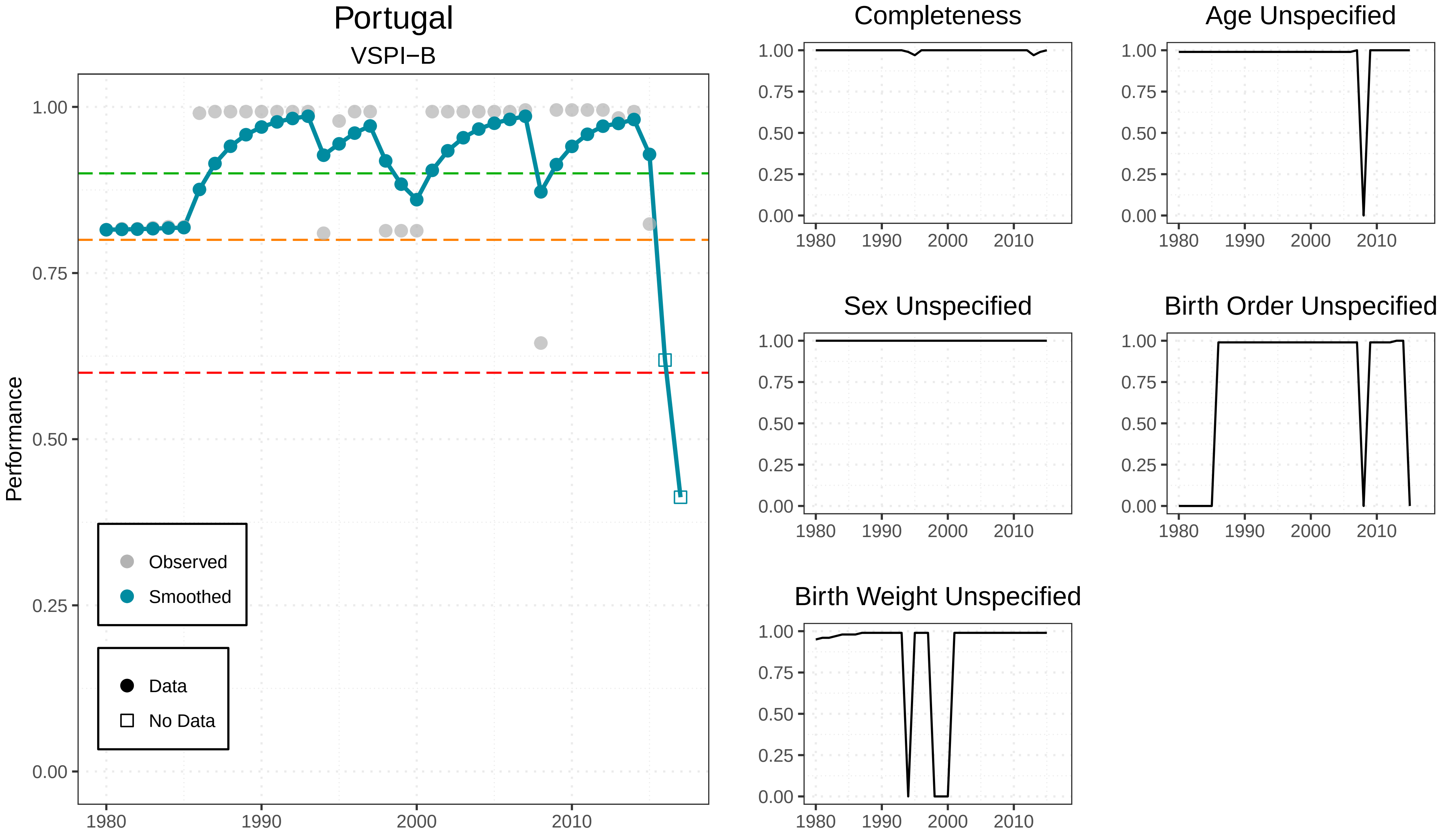

Portugal
VSPI−B
Performance
1.00
0.75
0.50
0.25
0.00
1980
1990
2000
2010
Observed
Smoothed
Data
No Data
Completeness
Age Unspecified
Sex Unspecified
Birth Order Unspecified
Birth Weight Unspecified

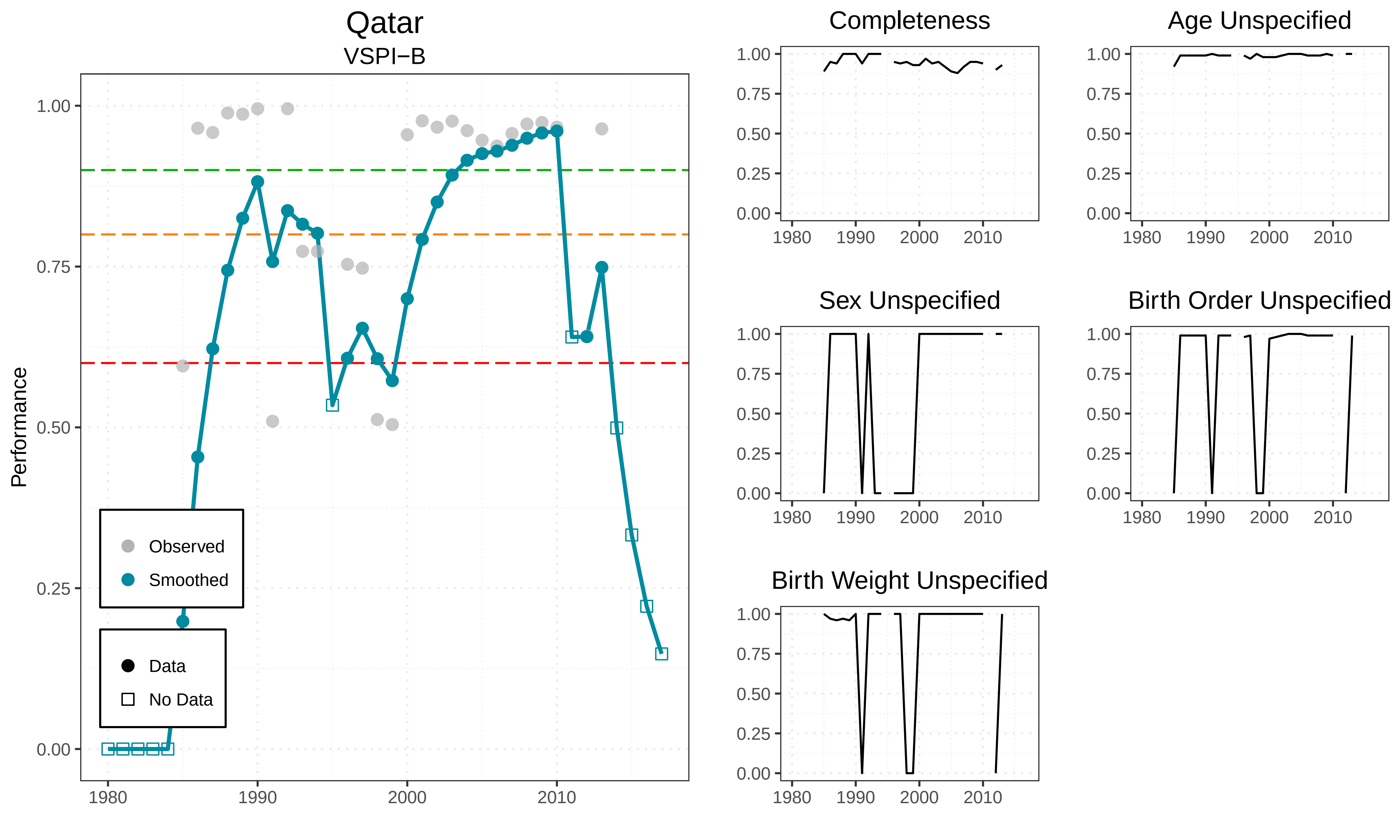

Qatar
VSPI−B
Performance
Observed
Smoothed
Data
No Data
Completeness
Age Unspecified
Sex Unspecified
Birth Order Unspecified
Birth Weight Unspecified

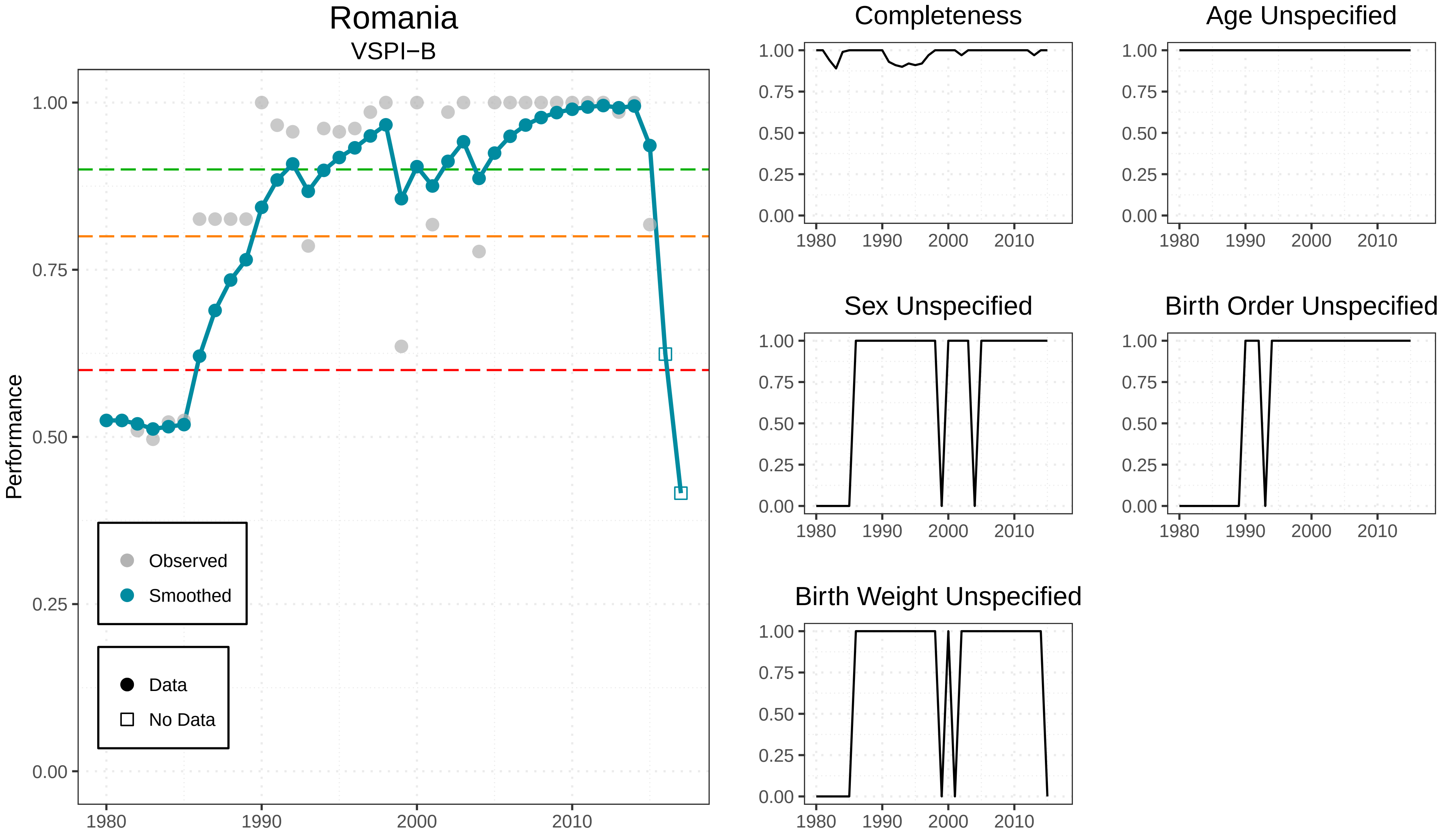

Romania
VSPI−B
Performance
1.00
0.75
0.50
0.25
0.00
1980
1990
2000
2010
Observed
Smoothed
Data
No Data
Completeness
Age Unspecified
Sex Unspecified
Birth Order Unspecified
Birth Weight Unspecified

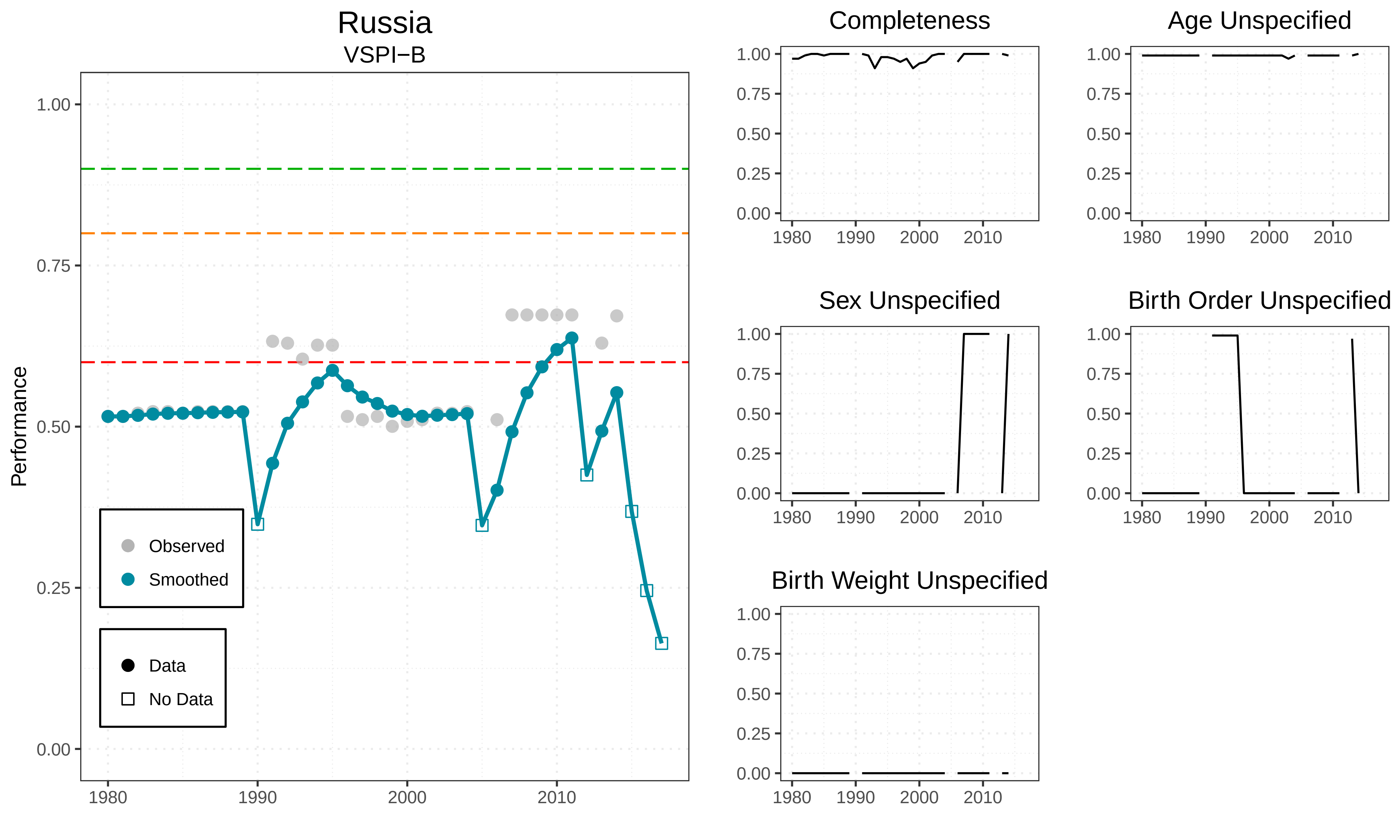
Russia
VSPI−B
Performance
1.00
0.75
0.50
0.25
0.00
1980
1990
2000
2010
Observed
Smoothed
Data
No Data
Completeness
Age Unspecified
Sex Unspecified
Birth Order Unspecified
Birth Weight Unspecified

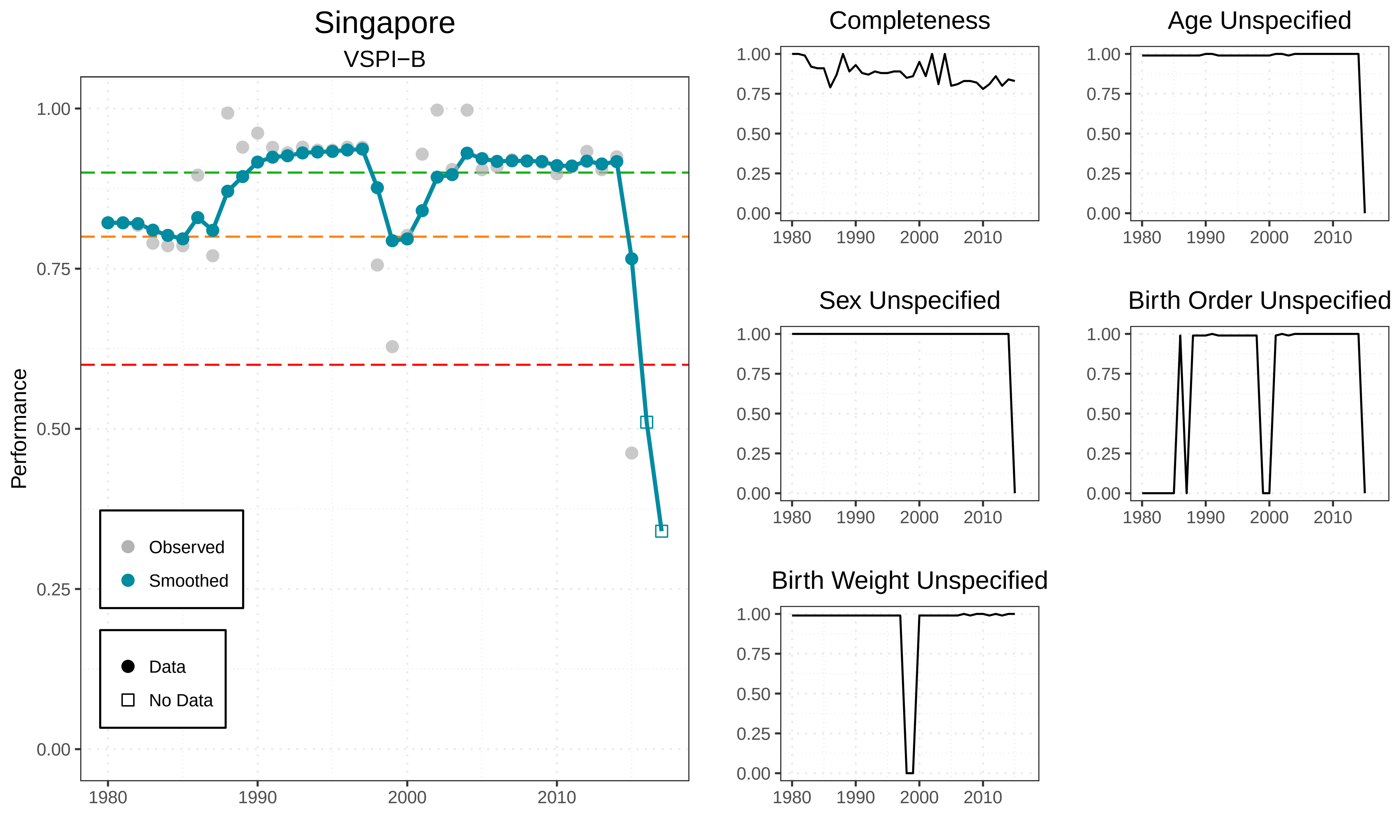

Singapore
VSPI−B
Performance
1.00
0.75
0.50
0.25
0.00
1980
1990
2000
2010
Observed
Smoothed
Data
No Data
Completeness
Age Unspecified
Sex Unspecified
Birth Order Unspecified
Birth Weight Unspecified

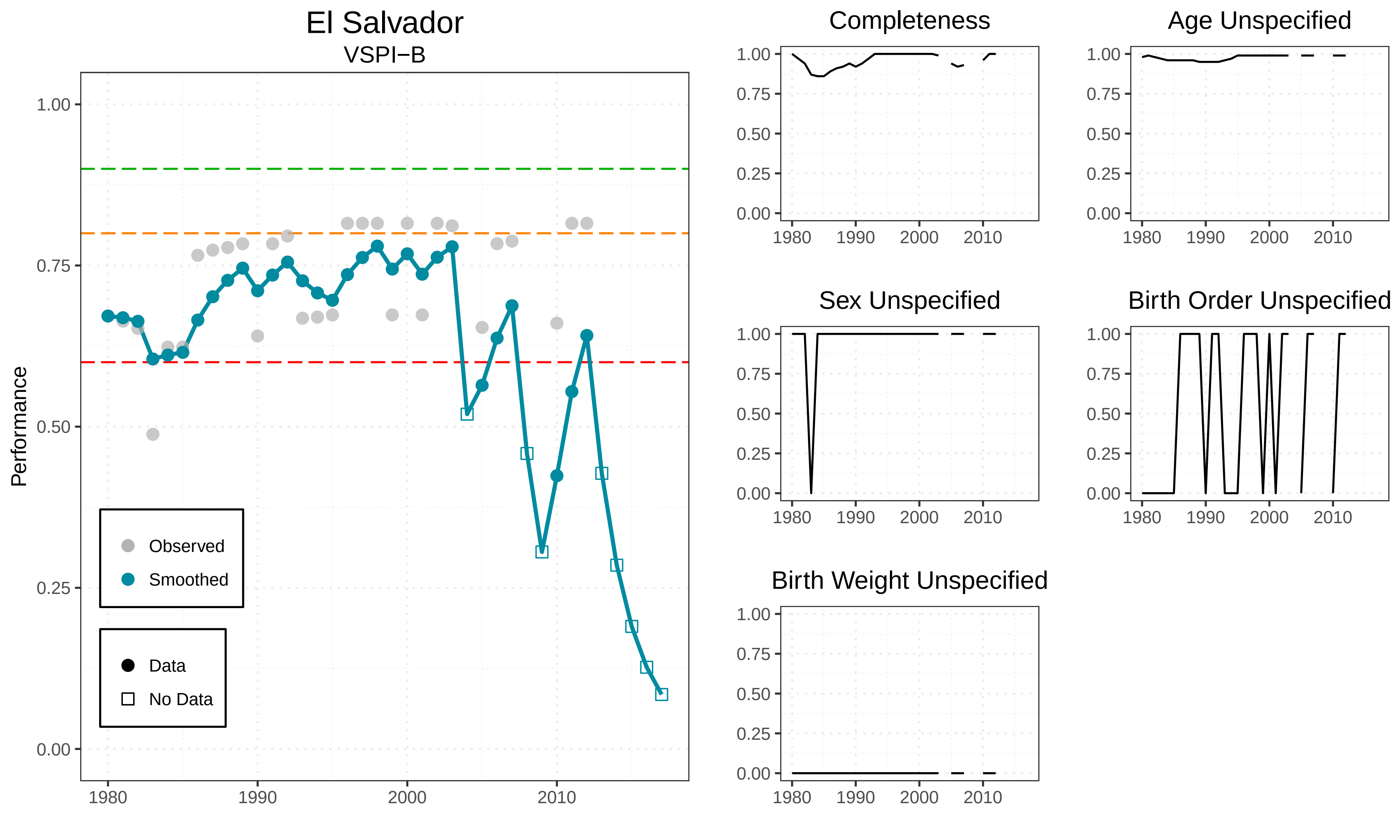

El Salvador
VSPI−B
Performance
1.00
0.75
0.50
0.25
0.00
1980
1990
2000
2010
Observed
Smoothed
Data
No Data
Completeness
Age Unspecified
Sex Unspecified
Birth Order Unspecified
Birth Weight Unspecified

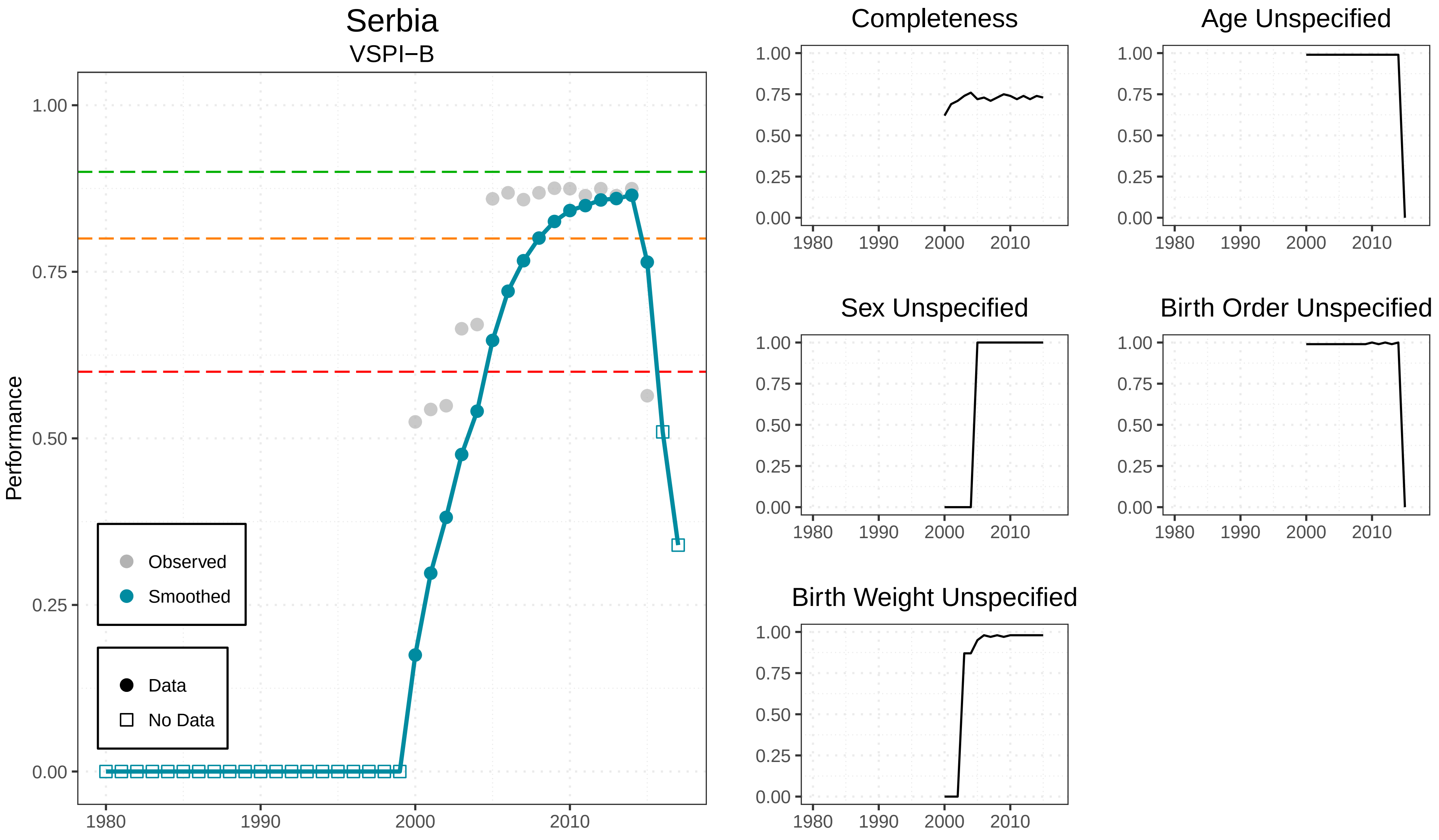

Serbia
VSPI−B
Performance
1.00
0.75
0.50
0.25
0.00
1980
1990
2000
2010
Observed
Smoothed
Data
No Data
Completeness
Age Unspecified
Sex Unspecified
Birth Order Unspecified
Birth Weight Unspecified

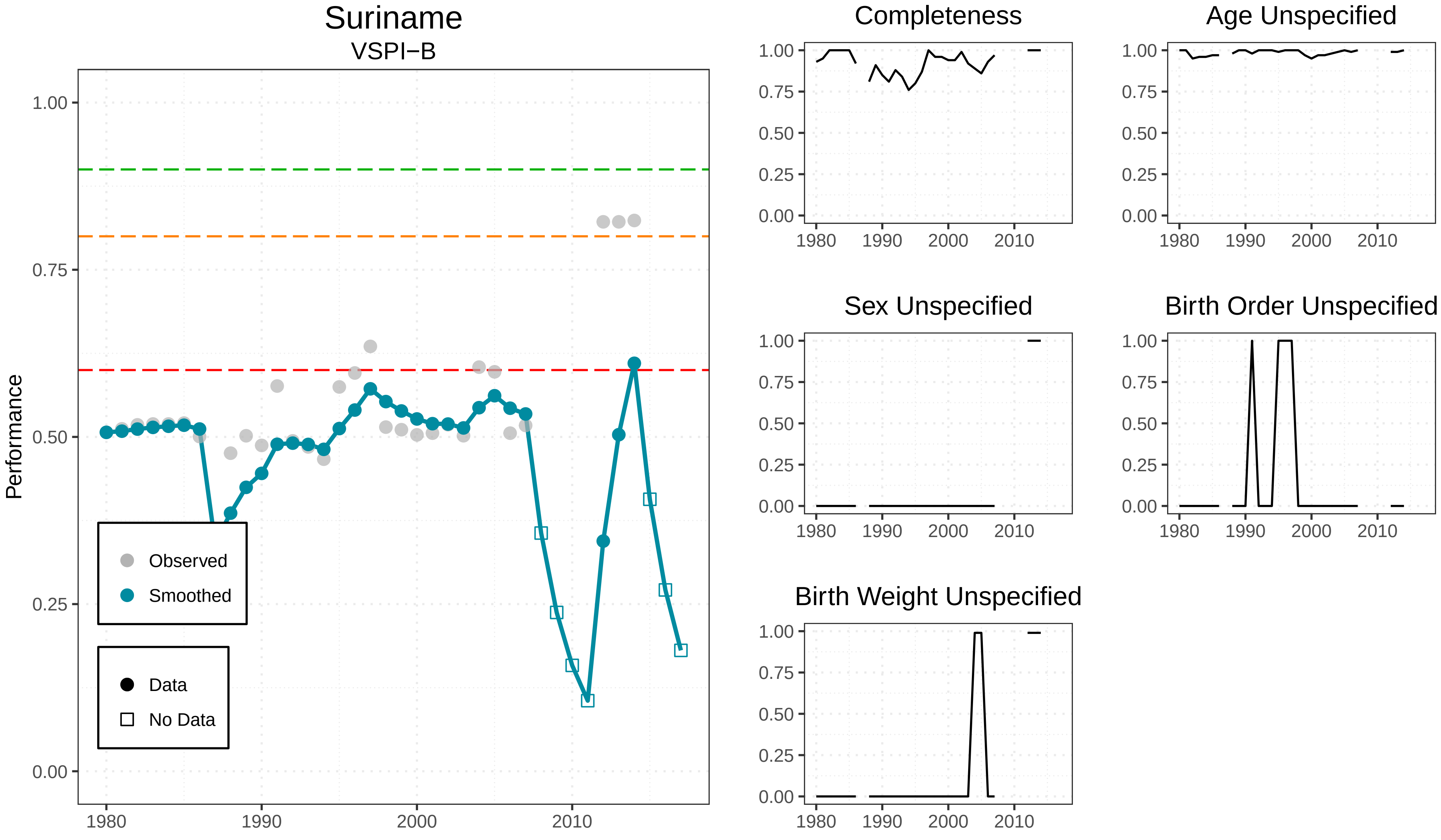

Suriname
VSPI−B
Performance
Observed
Smoothed
Data
No Data
Completeness
Age Unspecified
Sex Unspecified
Birth Order Unspecified
Birth Weight Unspecified

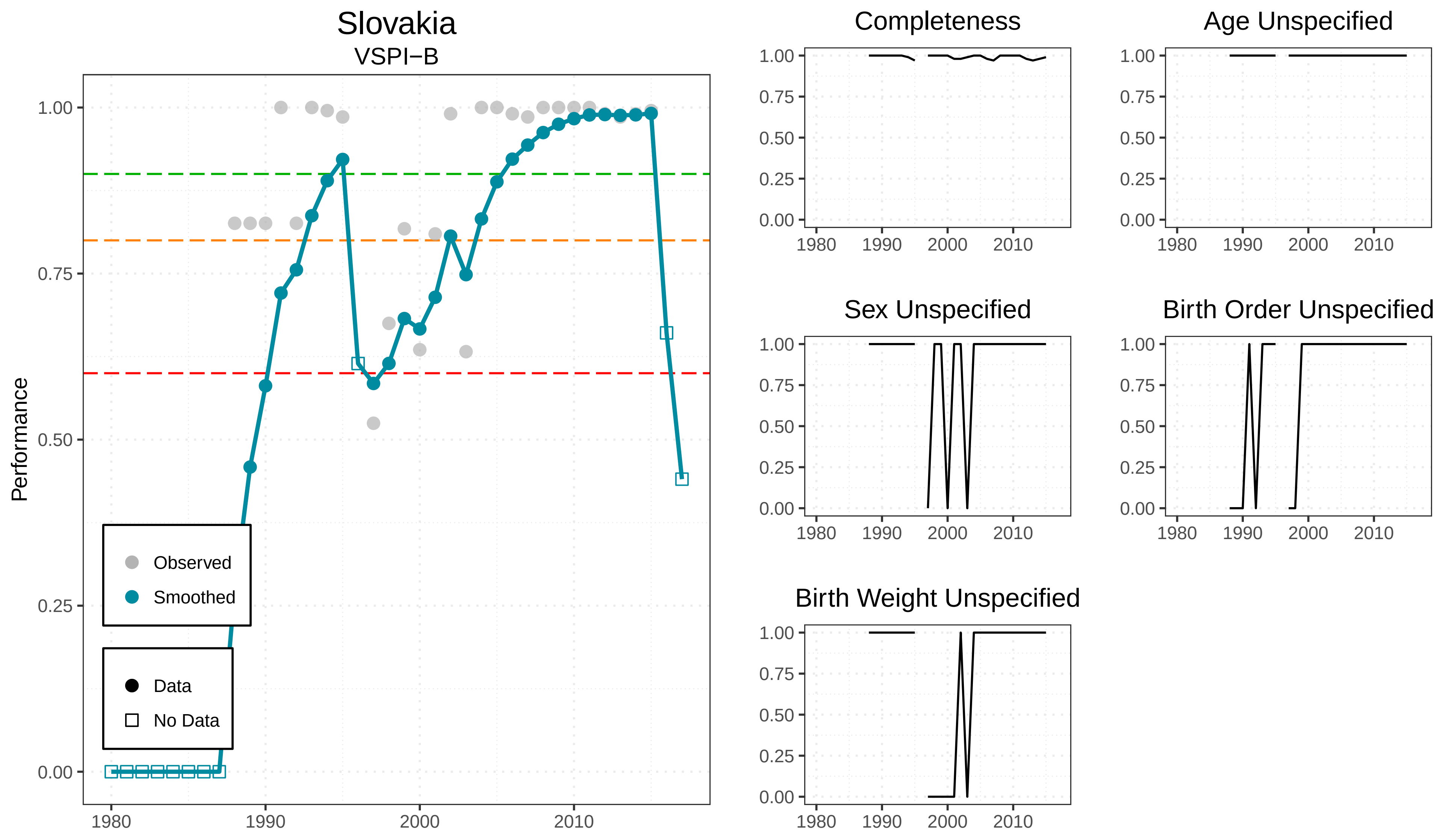
Slovakia
VSPI−B
Performance
1.00
0.75
0.50
0.25
0.00
1980
1990
2000
2010
Observed
Smoothed
Data
No Data
Completeness
Age Unspecified
Sex Unspecified
Birth Order Unspecified
Birth Weight Unspecified

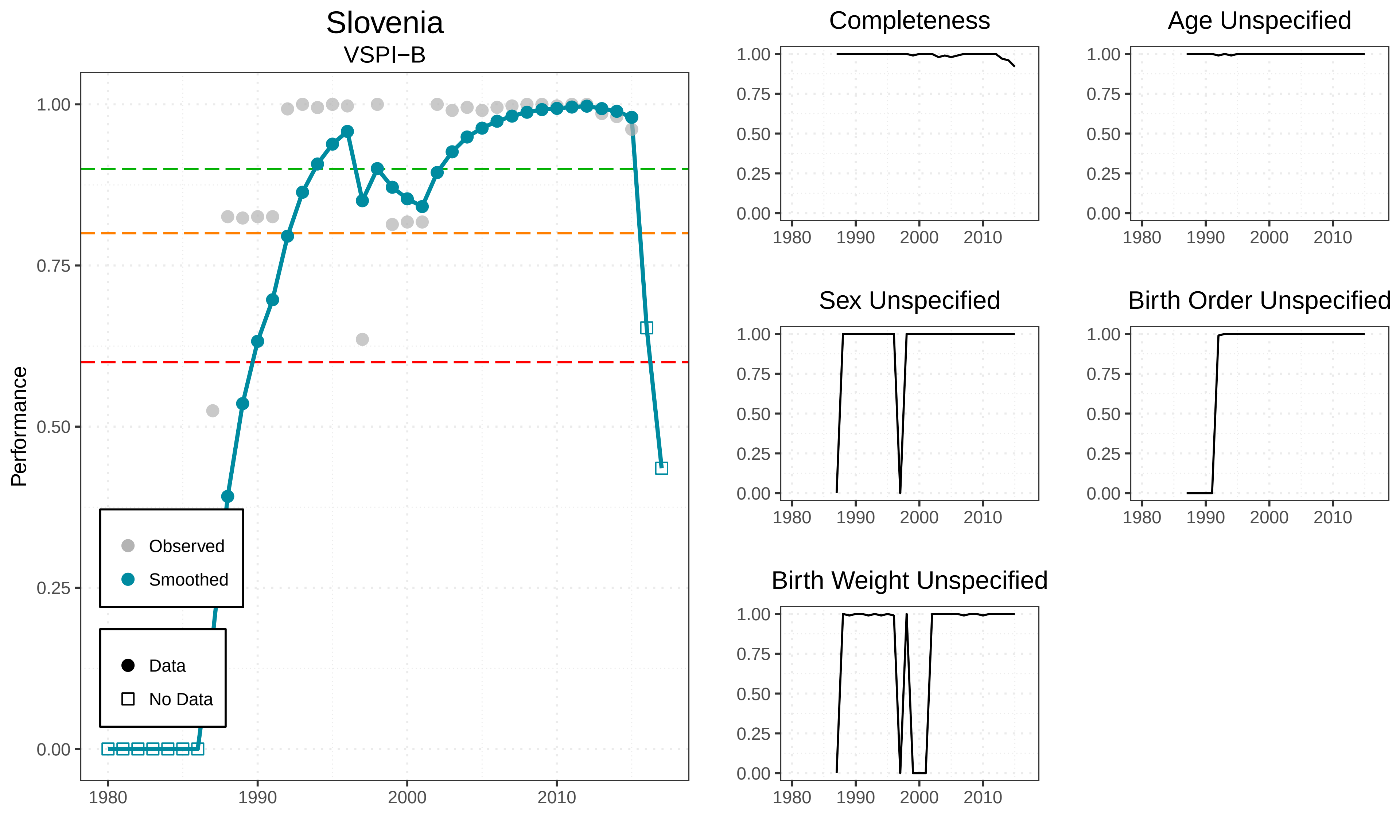

Slovenia
VSPI−B
Performance
1.00
0.75
0.50
0.25
0.00
1980
1990
2000
2010
Observed
Smoothed
Data
No Data
Completeness
Age Unspecified
Sex Unspecified
Birth Order Unspecified
Birth Weight Unspecified

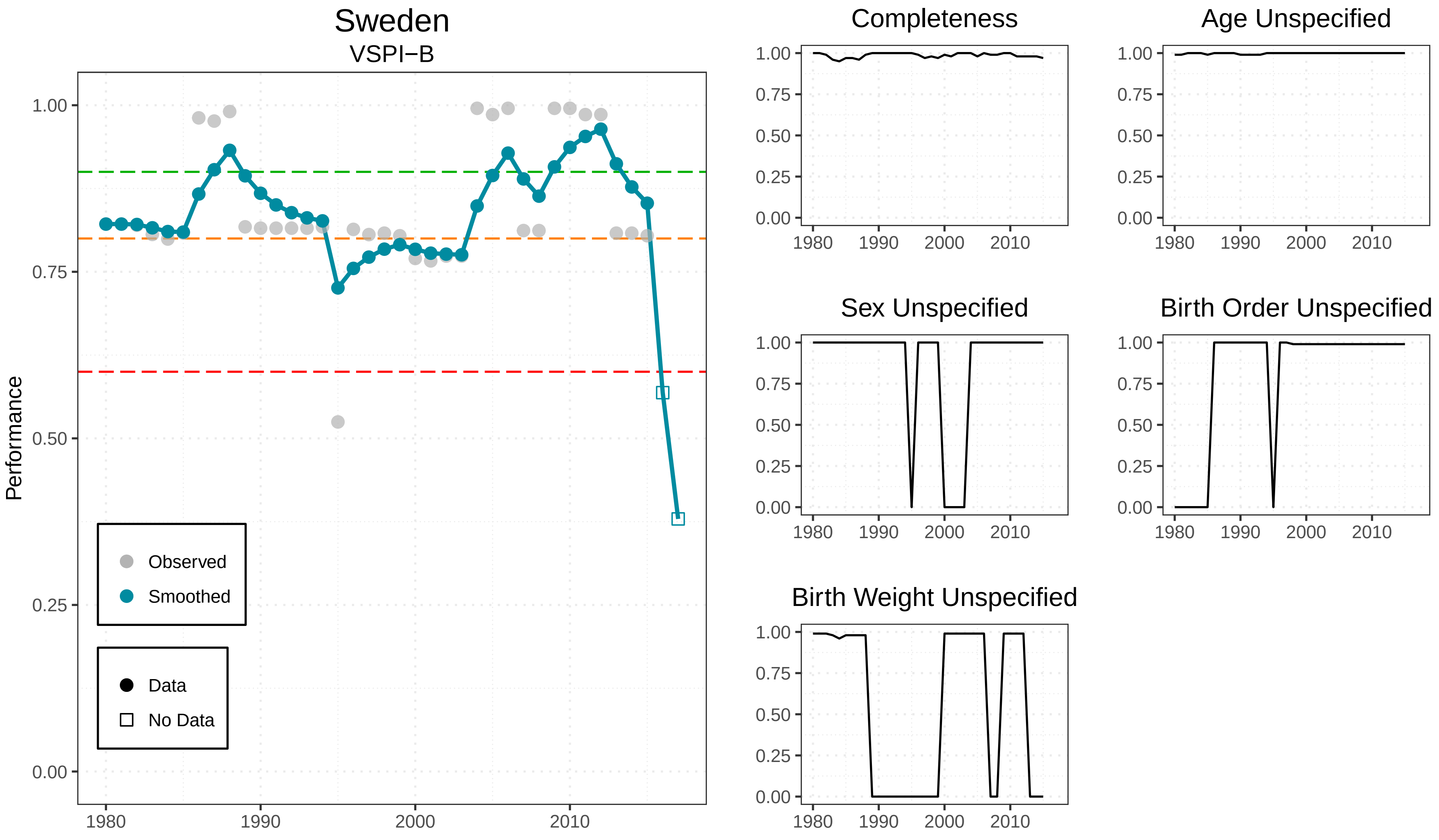

Sweden
VSPI−B
Performance
1.00
0.75
0.50
0.25
0.00
1980
1990
2000
2010
Observed
Smoothed
Data
No Data
Completeness
Age Unspecified
Sex Unspecified
Birth Order Unspecified
Birth Weight Unspecified

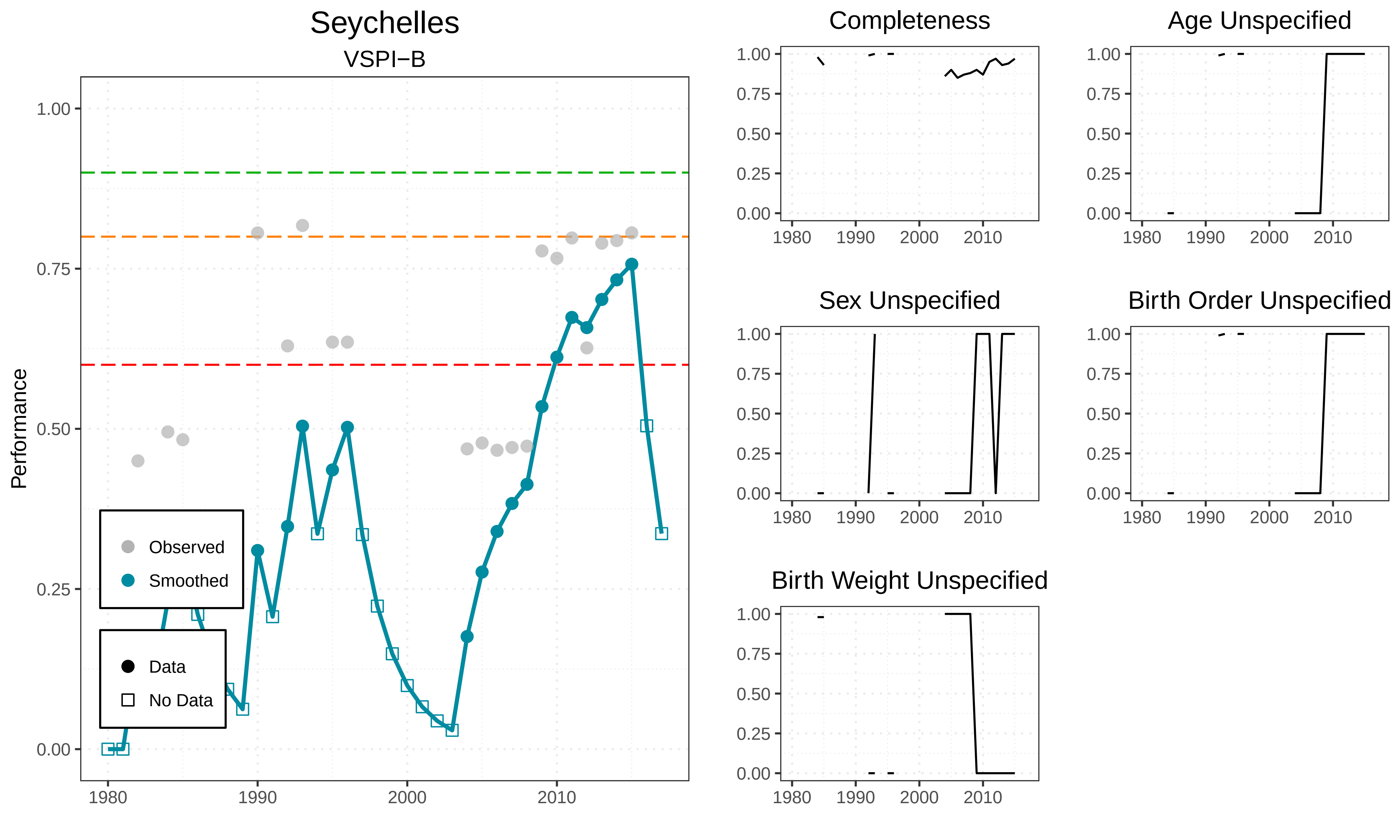
Seychelles
VSPI−B
Performance
Observed
Smoothed
Data
No Data
Completeness
Age Unspecified
Sex Unspecified
Birth Order Unspecified
Birth Weight Unspecified

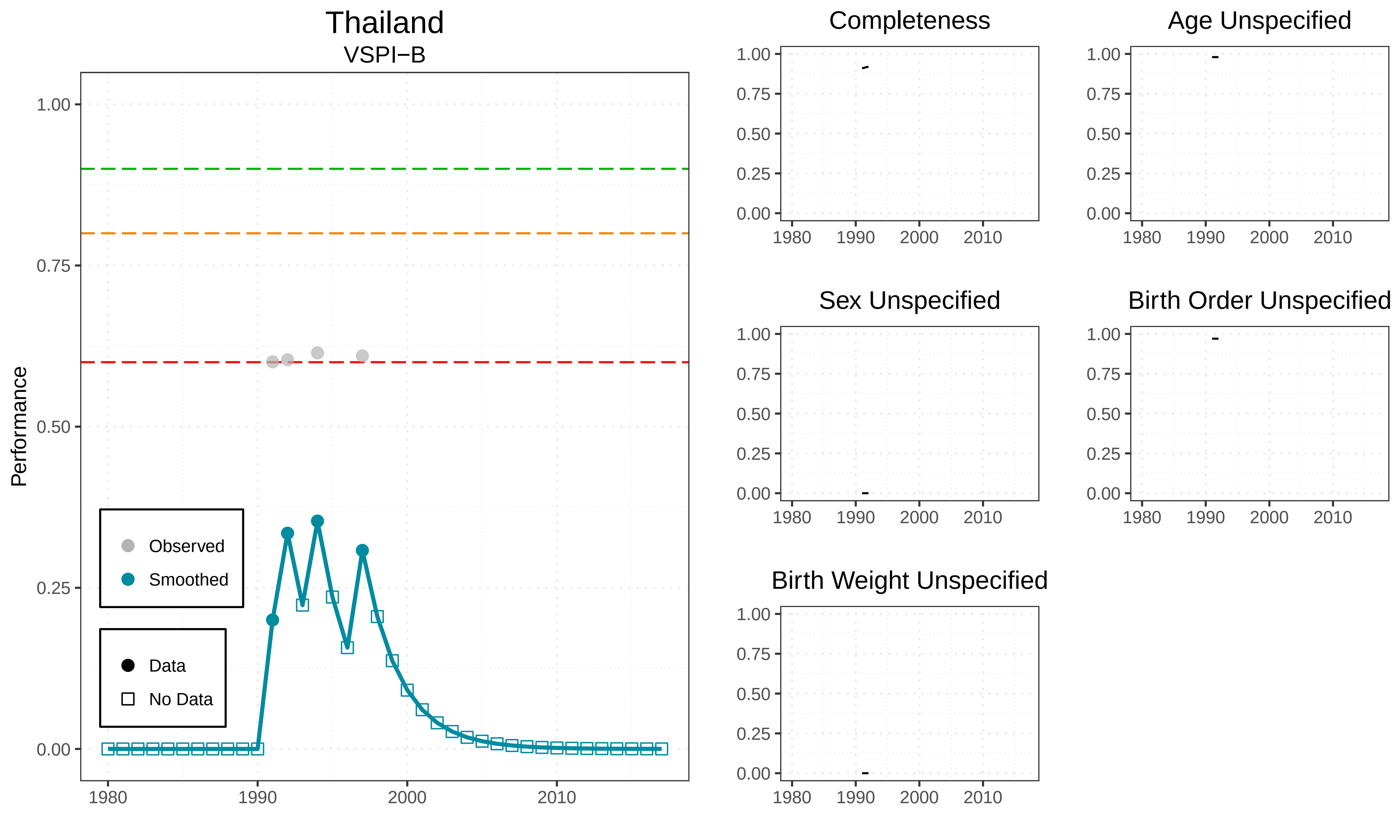

Thailand
VSPI−B
Performance
1.00
0.75
0.50
0.25
0.00
1980
1990
2000
2010
Observed
Smoothed
Data
No Data
Completeness
Age Unspecified
Sex Unspecified
Birth Order Unspecified
Birth Weight Unspecified

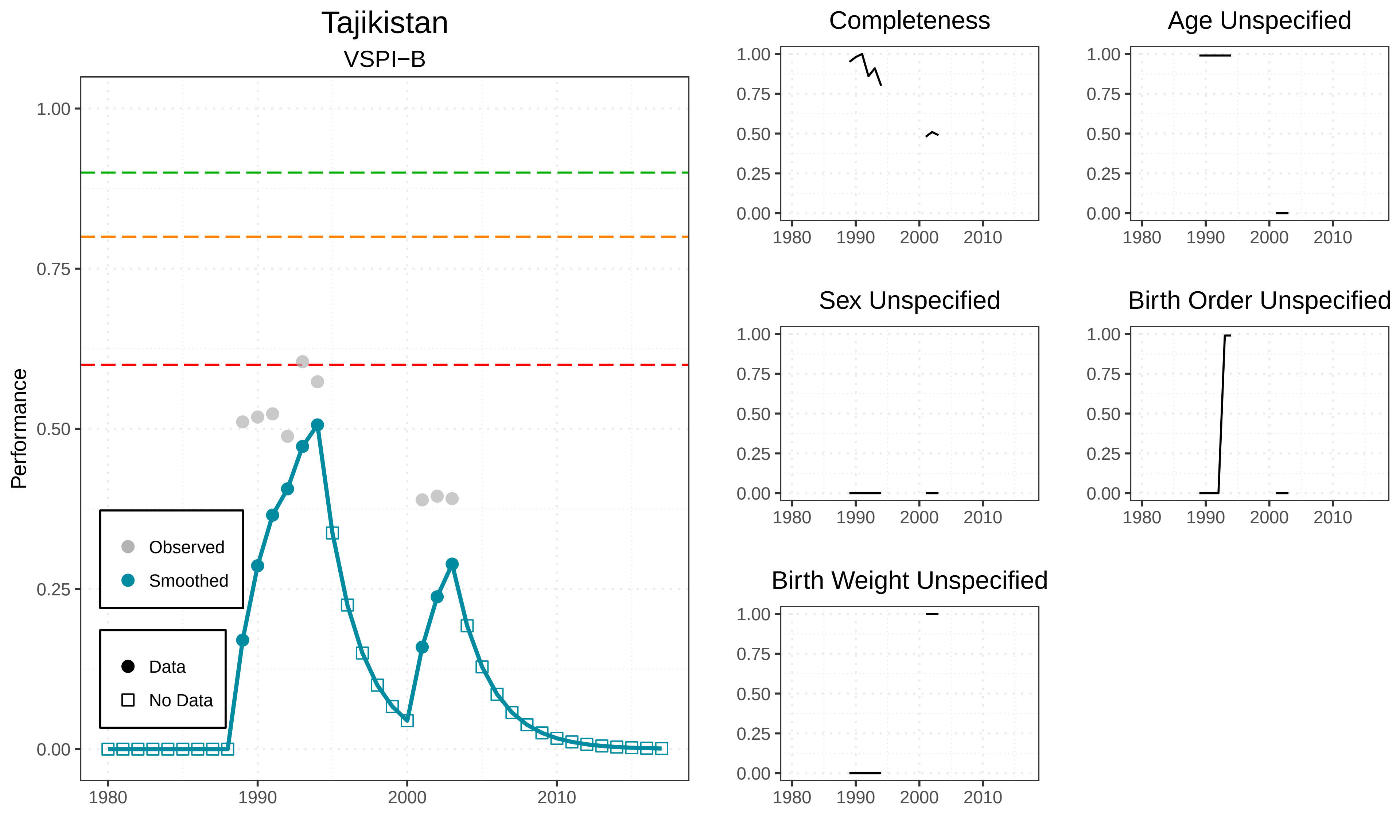

Tajikistan
VSPI−B
Performance
1.00
0.75
0.50
0.25
0.00
1980
1990
2000
2010
Observed
Smoothed
Data
No Data
Completeness
Age Unspecified
Sex Unspecified
Birth Order Unspecified
Birth Weight Unspecified

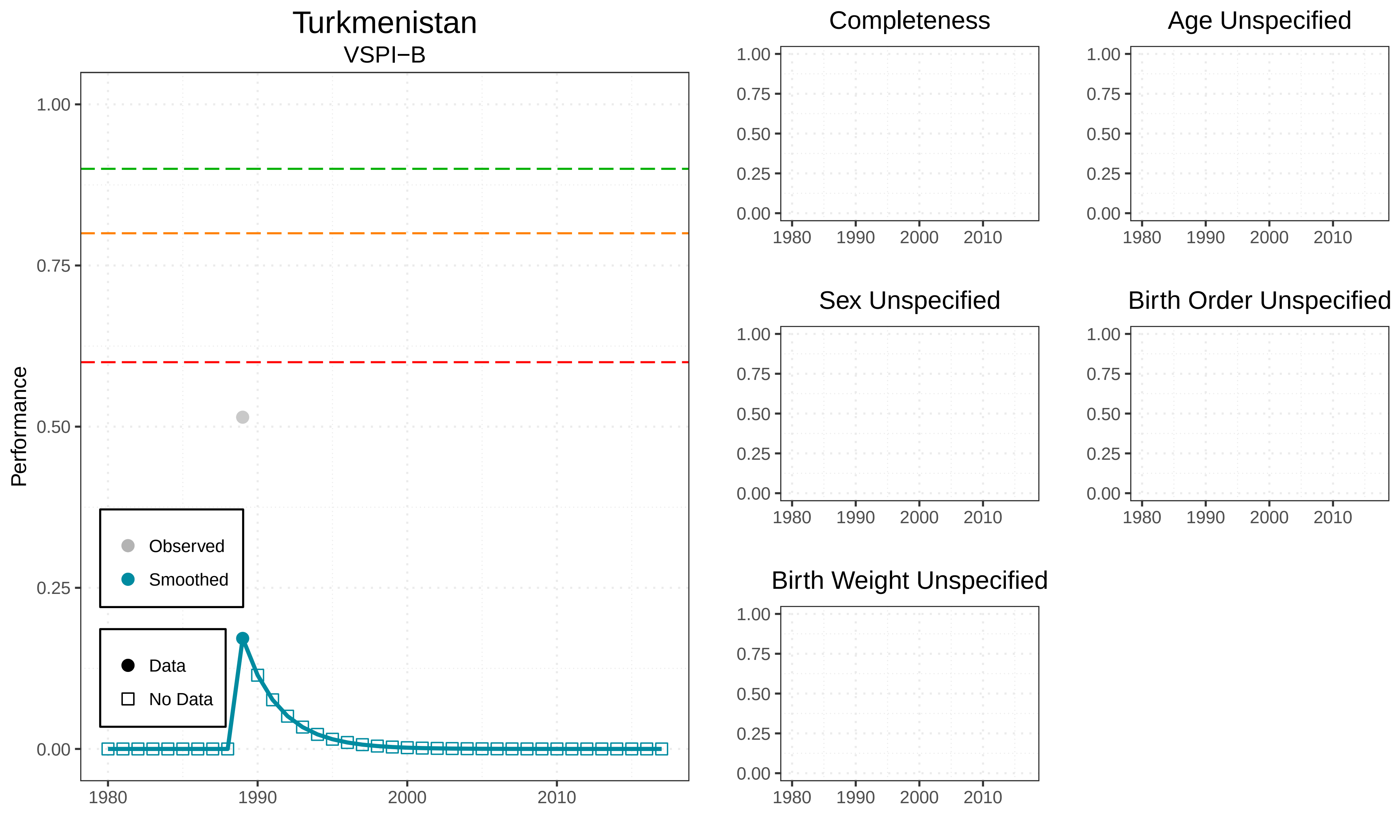
Turkmenistan
VSPI−B
Performance
1.00
0.75
0.50
0.25
0.00
1980
1990
2000
2010
Observed
Smoothed
Data
No Data
Completeness
Age Unspecified
Sex Unspecified
Birth Order Unspecified
Birth Weight Unspecified

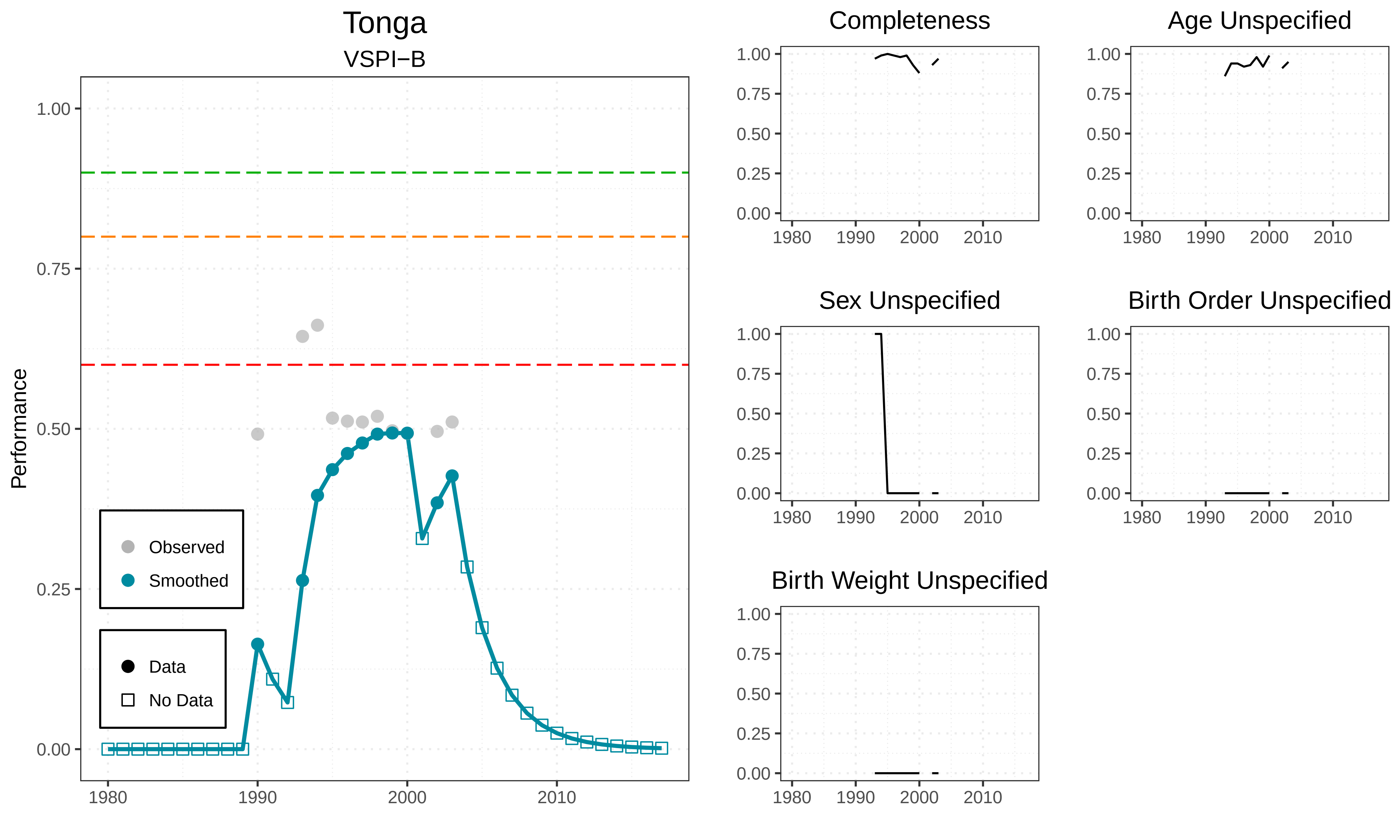

Tonga
VSPI−B
Performance
1.00
0.75
0.50
0.25
0.00
1980
1990
2000
2010
Observed
Smoothed
Data
No Data
Completeness
Age Unspecified
Sex Unspecified
Birth Order Unspecified
Birth Weight Unspecified

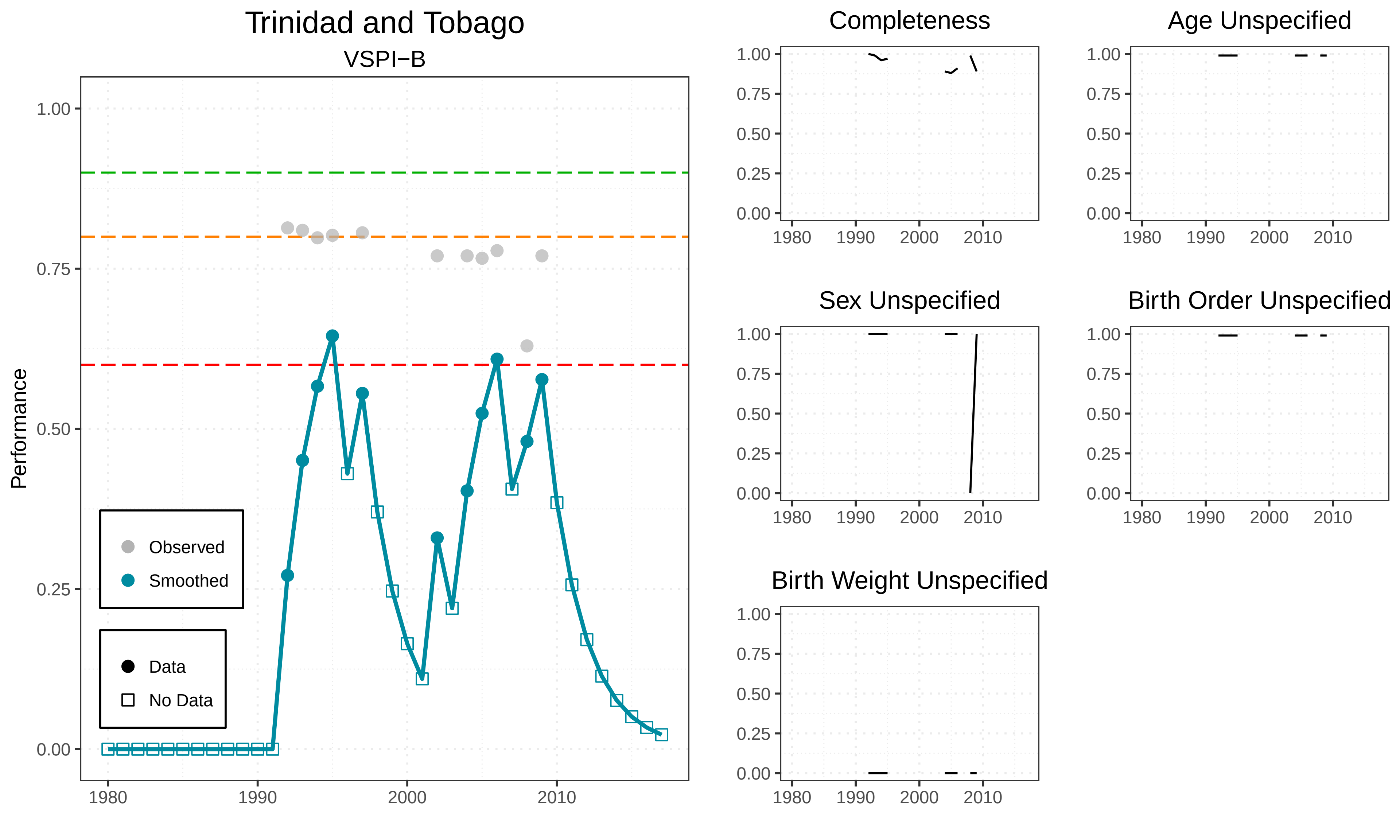

Trinidad and Tobago
VSPI−B
Performance
Observed
Smoothed
Data
No Data
Completeness
Age Unspecified
Sex Unspecified
Birth Order Unspecified
Birth Weight Unspecified

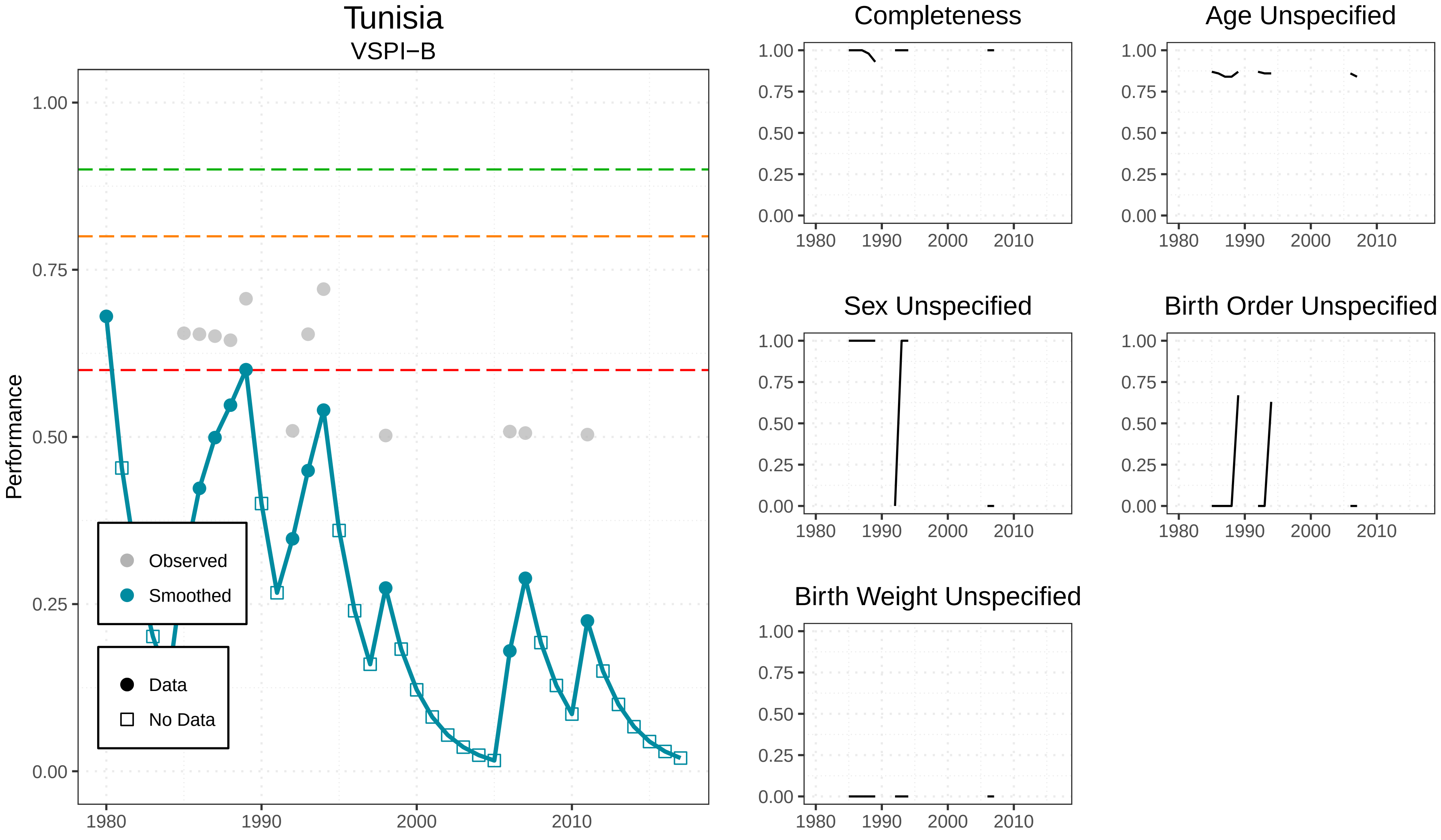

Tunisia
VSPI−B
Performance
1.00
0.75
0.50
0.25
0.00
1980
1990
2000
2010
Observed
Smoothed
Data
No Data
Completeness
Age Unspecified
Sex Unspecified
Birth Order Unspecified
Birth Weight Unspecified

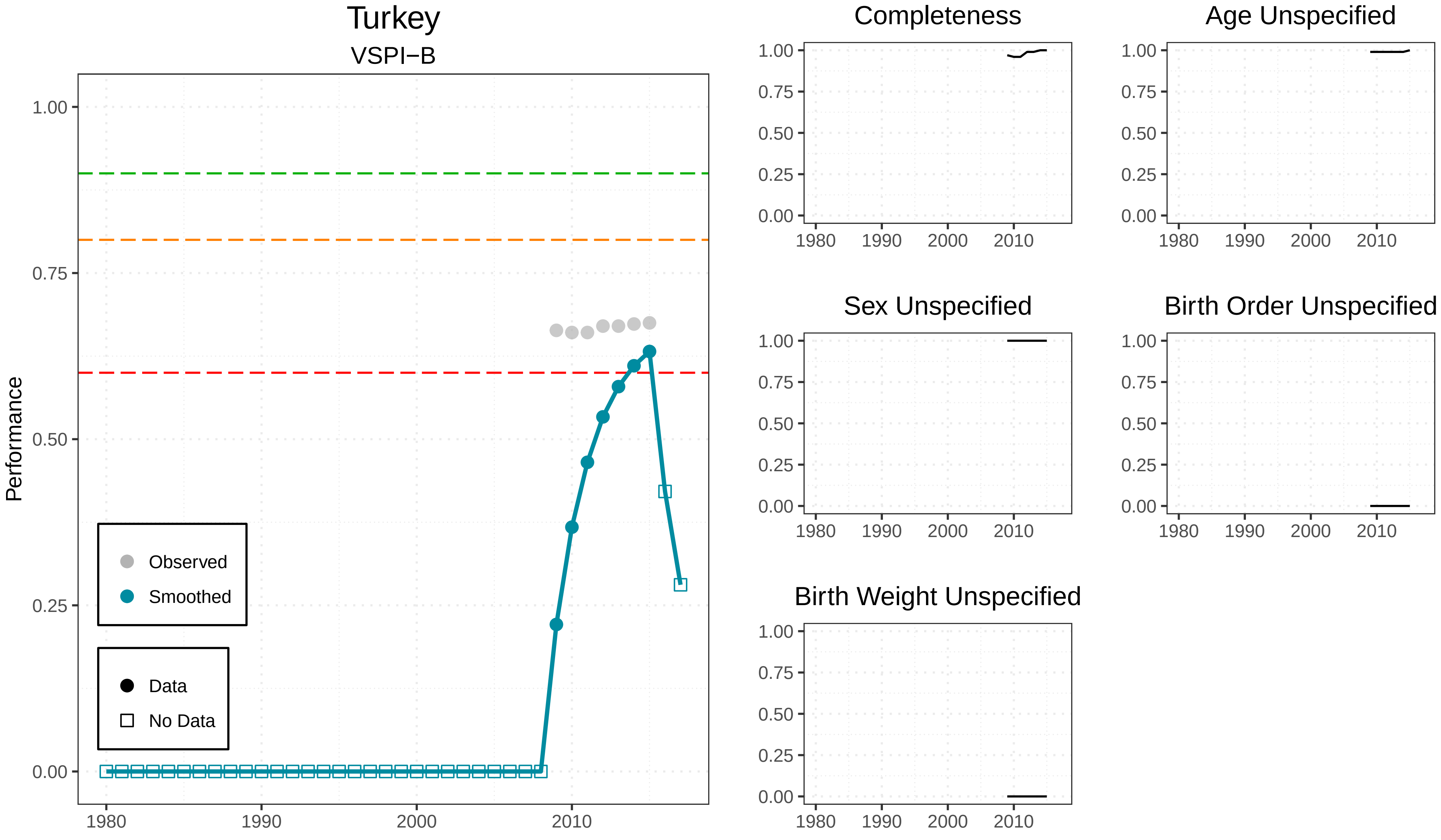

Turkey
VSPI−B
Performance
1.00
0.75
0.50
0.25
0.00
1980
1990
2000
2010
Observed
Smoothed
Data
No Data
Completeness
Age Unspecified
Sex Unspecified
Birth Order Unspecified
Birth Weight Unspecified

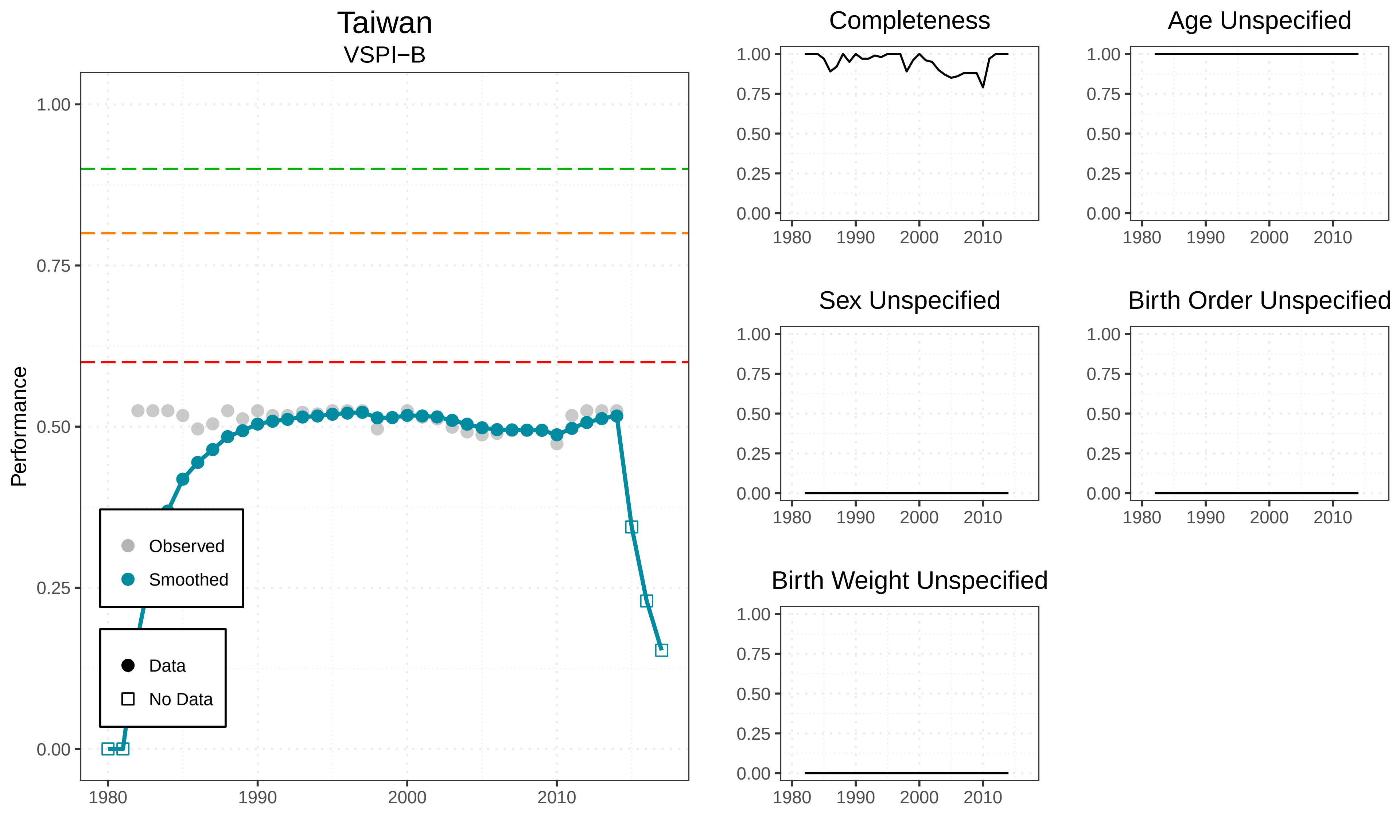

Taiwan
VSPI−B
Performance
Observed
Smoothed
Data
No Data
1.00
0.75
0.50
0.25
0.00
1980
1990
2000
2010
Completeness
Age Unspecified
Sex Unspecified
Birth Order Unspecified
Birth Weight Unspecified

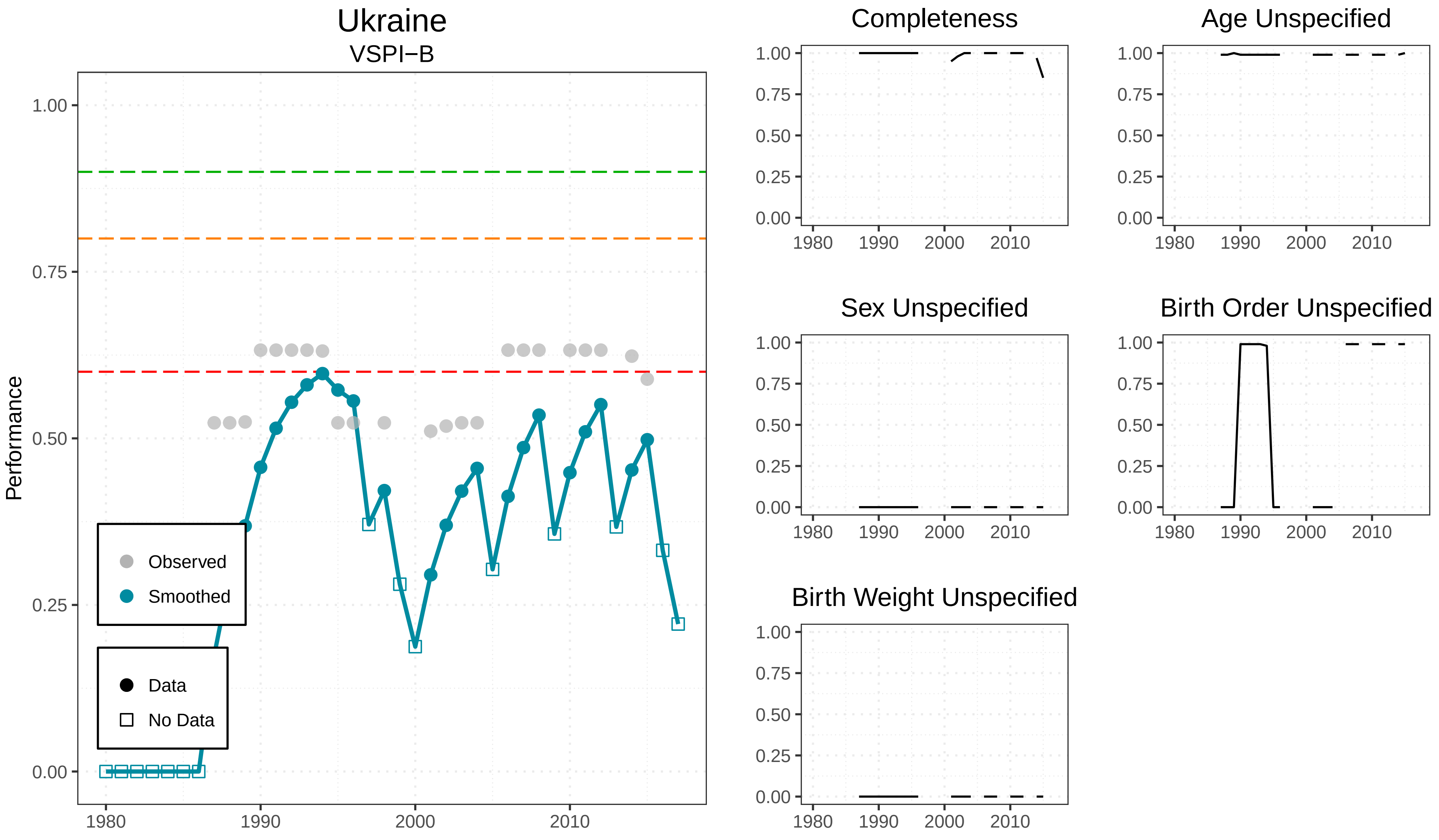

Ukraine
VSPI−B
Performance
1.00
0.75
0.50
0.25
0.00
1980
1990
2000
2010
Observed
Smoothed
Data
No Data
Completeness
Age Unspecified
Sex Unspecified
Birth Order Unspecified
Birth Weight Unspecified

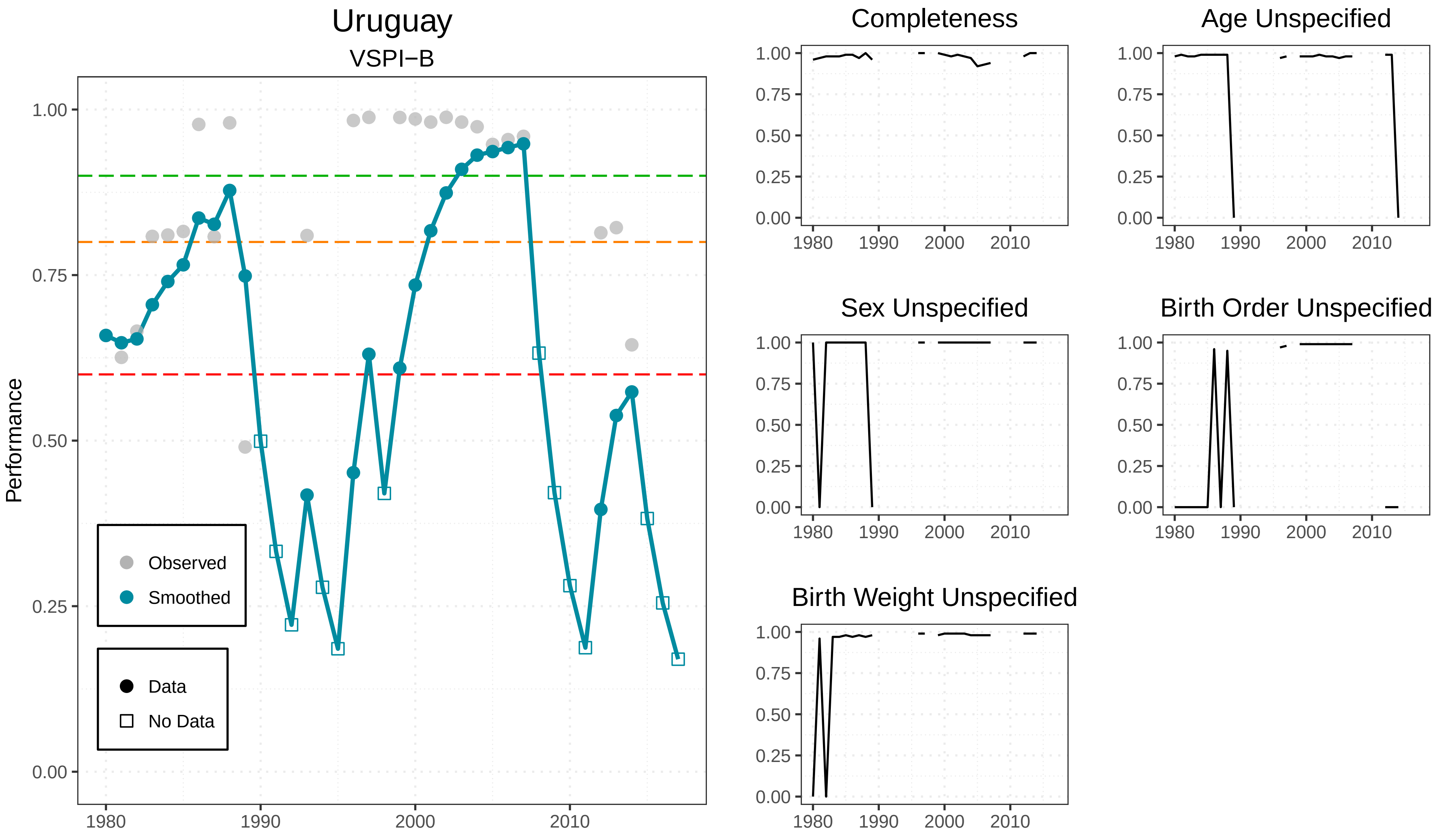

Uruguay
VSPI−B
Performance
1.00
0.75
0.50
0.25
0.00
1980
1990
2000
2010
Observed
Smoothed
Data
No Data
Completeness
Age Unspecified
Sex Unspecified
Birth Order Unspecified
Birth Weight Unspecified

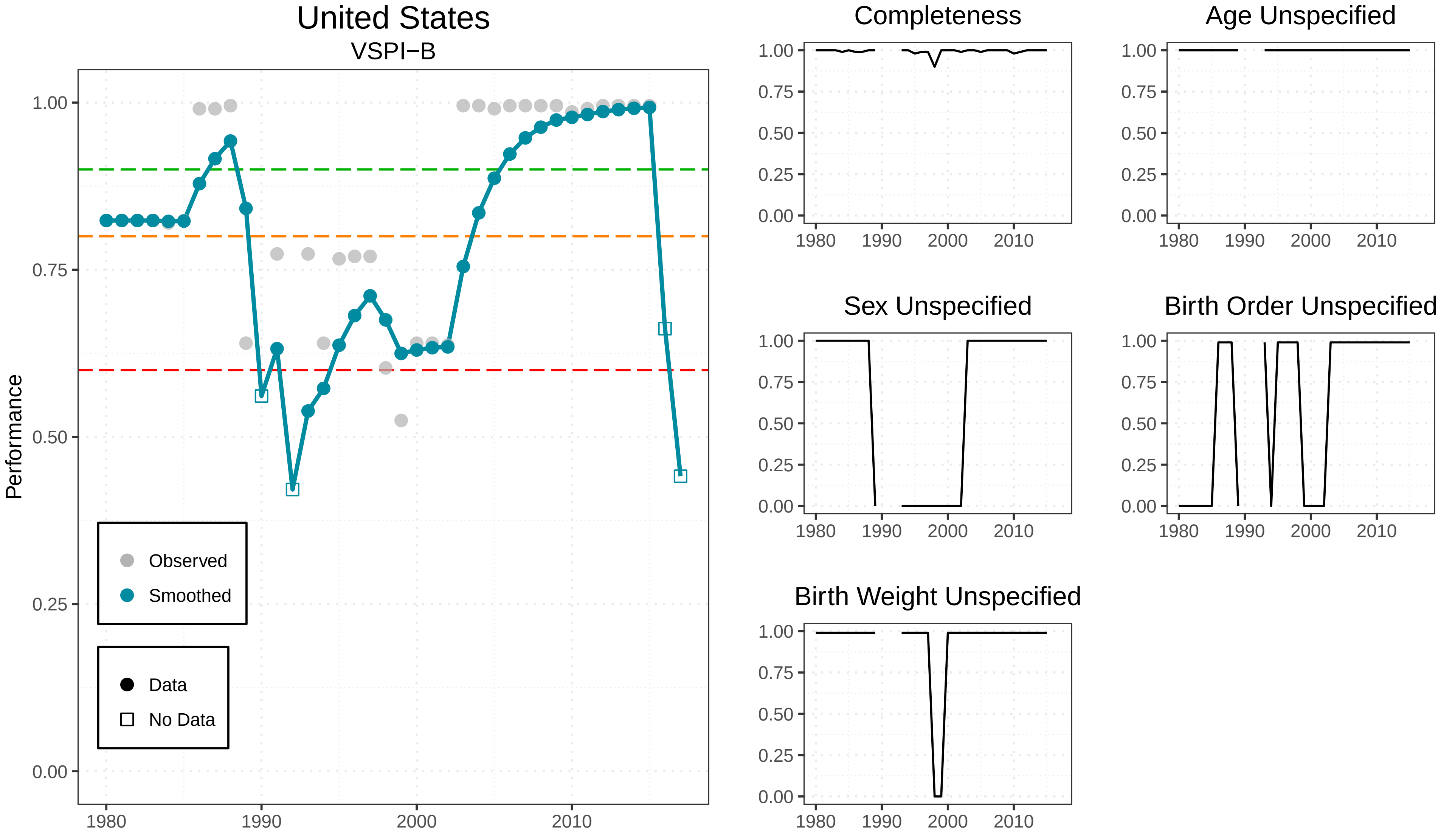

United States
VSPI−B
Performance
1.00
0.75
0.50
0.25
0.00
1980
1990
2000
2010
Observed
Smoothed
Data
No Data
Completeness
Age Unspecified
Sex Unspecified
Birth Order Unspecified
Birth Weight Unspecified

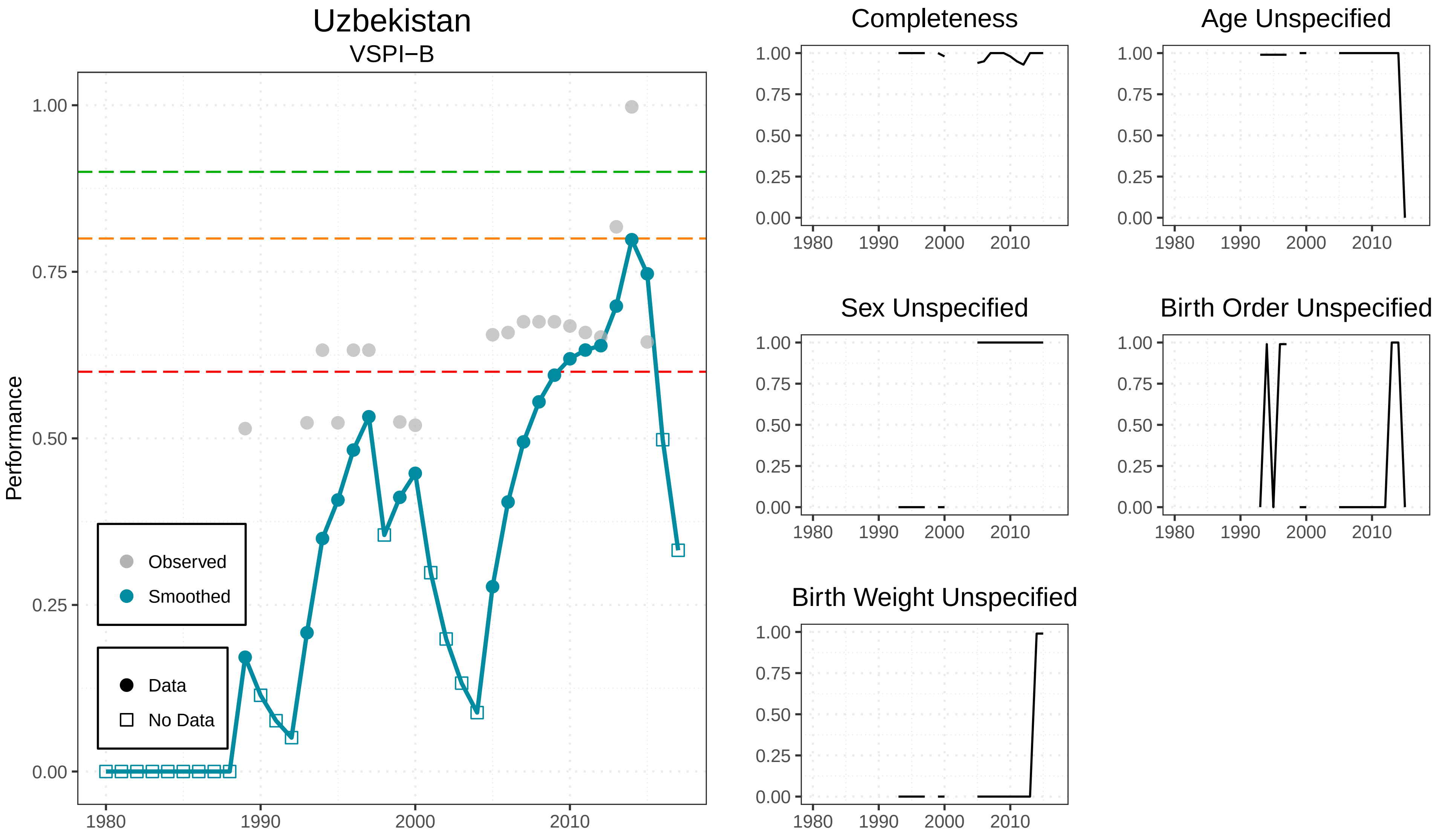

Uzbekistan
VSPI−B
Performance
1.00
0.75
0.50
0.25
0.00
1980
1990
2000
2010
Observed
Smoothed
Data
No Data
Completeness
Age Unspecified
Sex Unspecified
Birth Order Unspecified
Birth Weight Unspecified

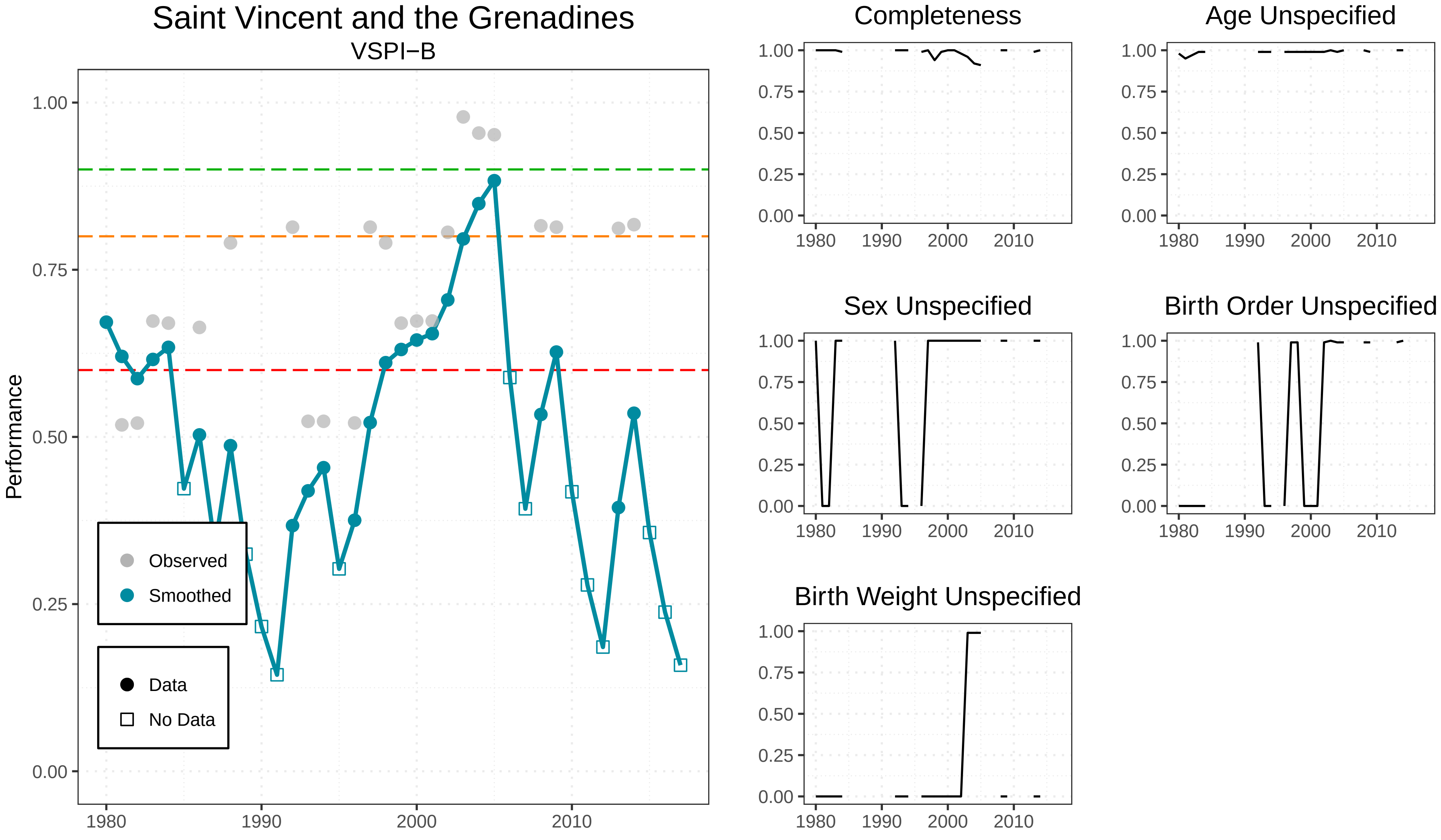

Saint Vincent and the Grenadines
VSPI−B
Performance
1.00
0.75
0.50
0.25
0.00
1980
1990
2000
2010
Observed
Smoothed
Data
No Data
Completeness
Age Unspecified
Sex Unspecified
Birth Order Unspecified
Birth Weight Unspecified

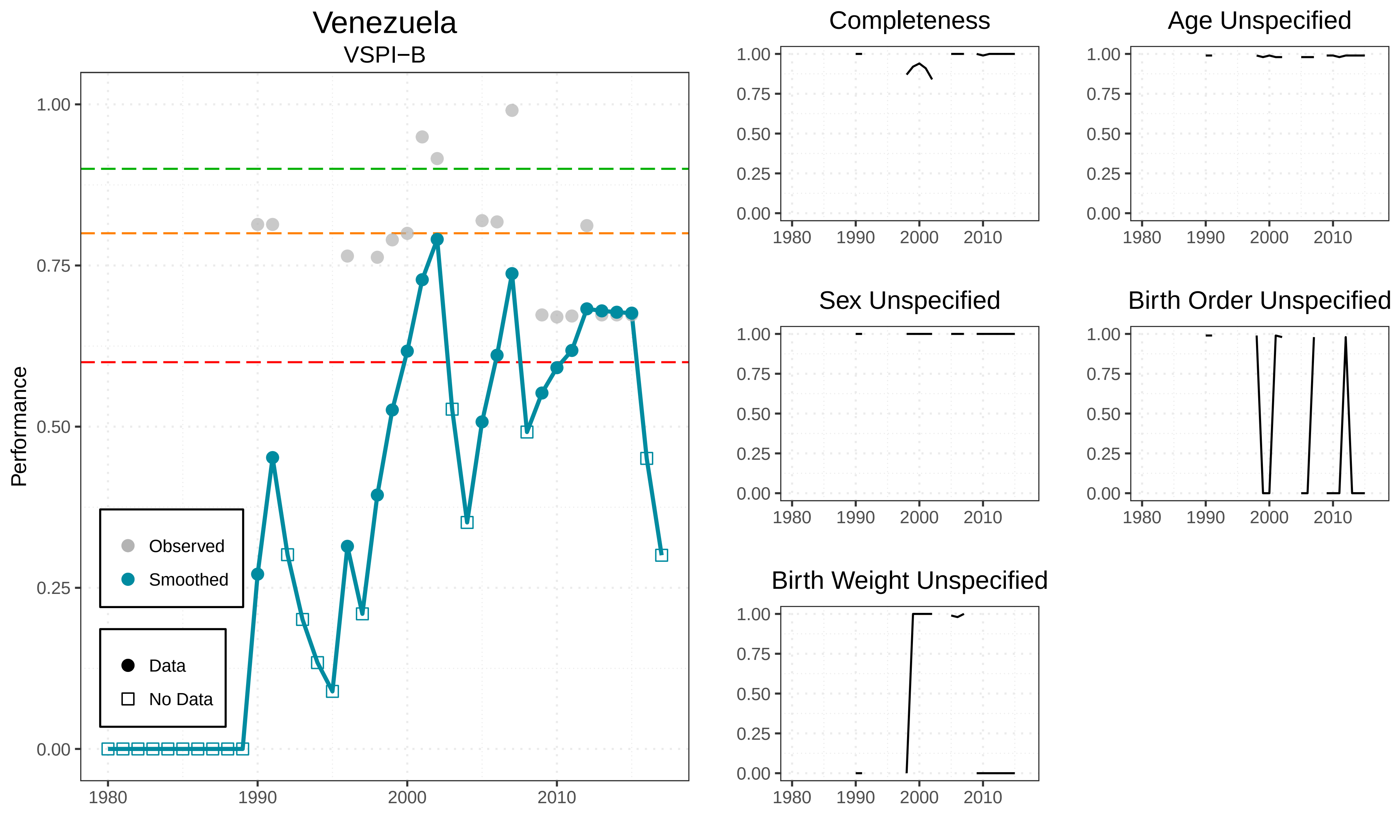

Venezuela
VSPI−B
Performance
1.00
0.75
0.50
0.25
0.00
1980
1990
2000
2010
Observed
Smoothed
Data
No Data
Completeness
Age Unspecified
Sex Unspecified
Birth Order Unspecified
Birth Weight Unspecified

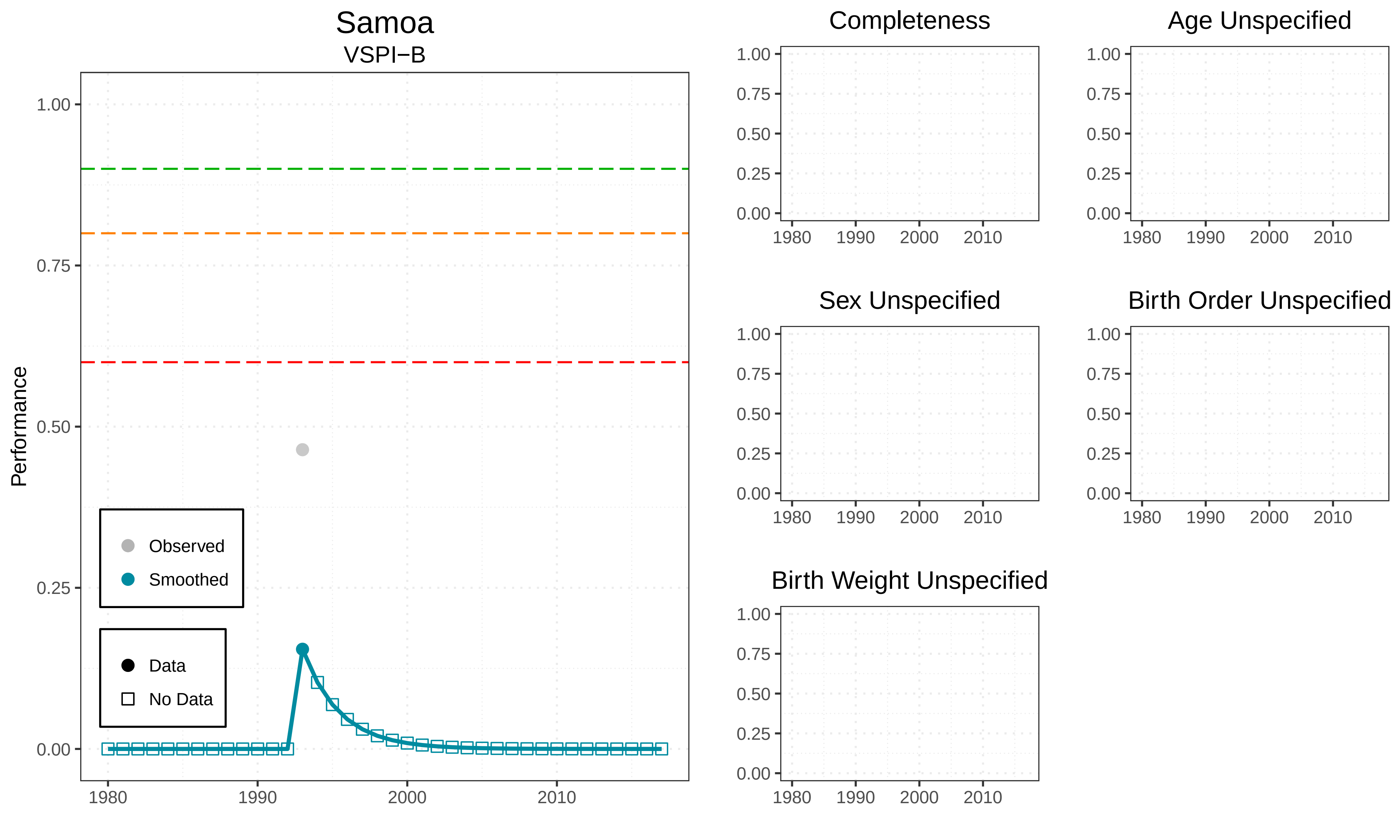
Samoa
VSPI−B
Performance
Observed
Smoothed
Data
No Data
1.00
0.75
0.50
0.25
0.00
1980
1990
2000
2010
Completeness
Age Unspecified
Sex Unspecified
Birth Order Unspecified
Birth Weight Unspecified

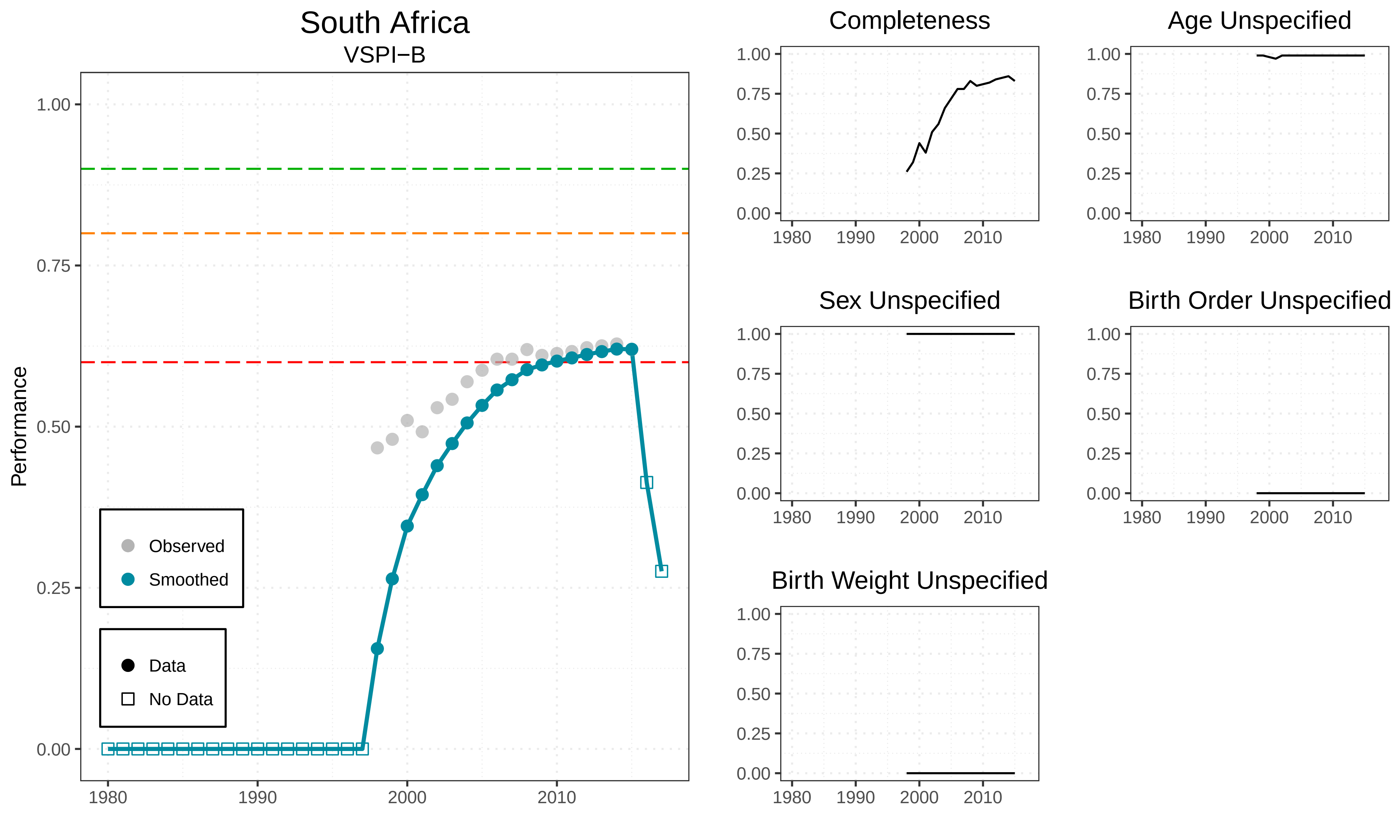
South Africa
VSPI−B
Performance
1.00
0.75
0.50
0.25
0.00
1980
1990
2000
2010
Observed
Smoothed
Data
No Data
Completeness
Age Unspecified
Sex Unspecified
Birth Order Unspecified
Birth Weight Unspecified
