# Supplementary material for: How useful are registered birth statistics for health and social policy? A global systematic assessment of the availability and quality of birth registration data
Source: Popul Health Metr. 2018 Dec 27;16:21. doi: 10.1186/s12963-018-0180-6 (PMC6307230; doi:10.1186/s12963-018-0180-6)
Supplement: Supplementary file 3 — Simulated Age-Sex-Parity-Birthweight Fraction Accuracy Associated with Each Indicator. A figure displaying the results of the simulation procedure. The lines demonstrate the accuracy of simulated data in terms of the fraction of births in each birth group, as compared to the underlying population. Each line represents a different component of the VSPI-B at different simulated levels of that component. (PDF 7 kb) [file 12963_2018_180_MOESM3_ESM.pdf]

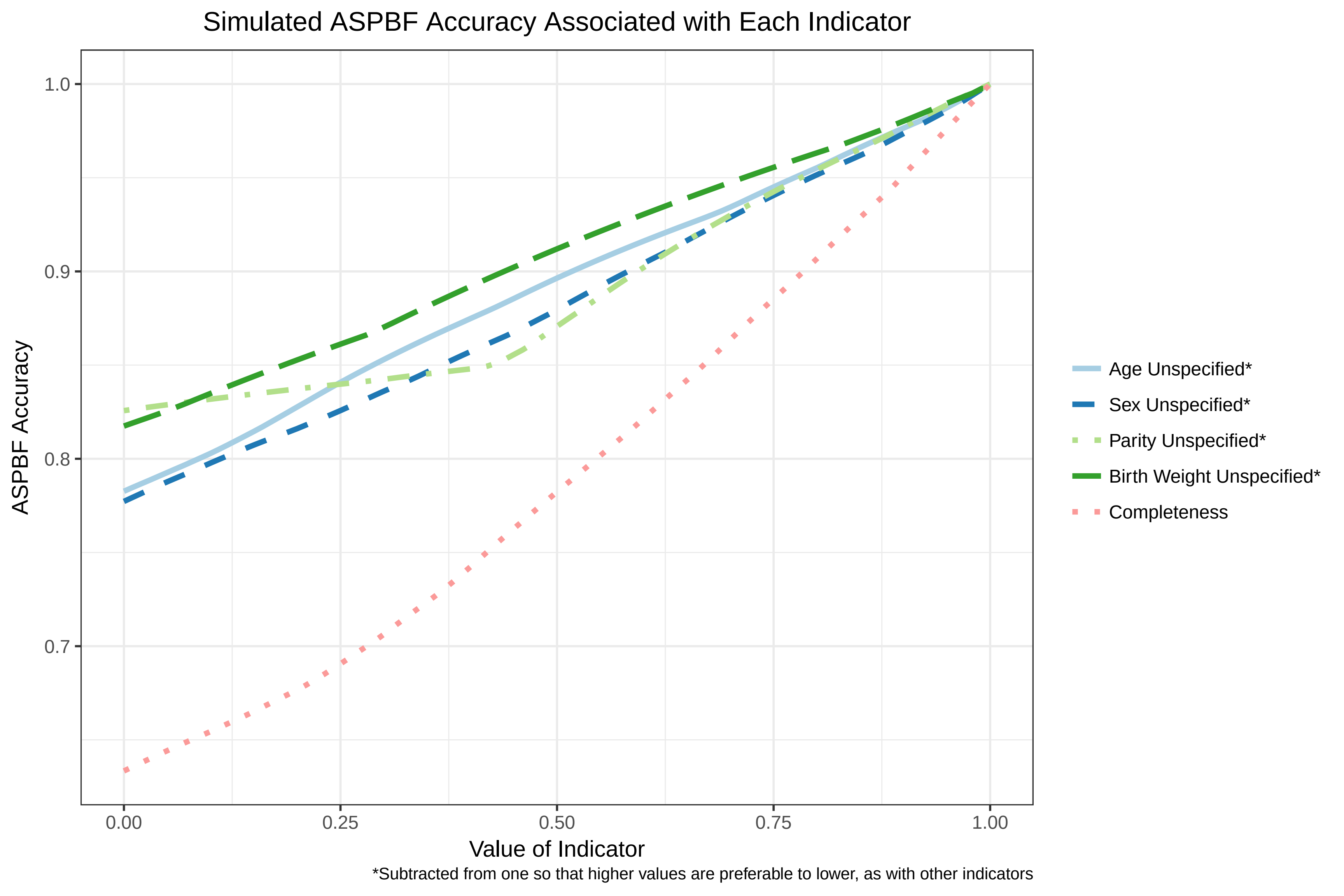
Simulated ASPBF Accuracy Associated with Each Indicator
1.0
0.9
0.8
0.7
ASPBF Accuracy
0.00
0.25
0.50
0.75
1.00
Value of Indicator
Age Unspecified*
Sex Unspecified*
Parity Unspecified*
Birth Weight Unspecified*
Completeness
*Subtracted from one so that higher values are preferable to lower, as with other indicators
